# Precipitation is the main axis of tropical plant phylogenetic turnover across space and time

**DOI:** 10.1126/sciadv.ade4954

**Published:** 2023-02-17

**Authors:** Jens J. Ringelberg, Erik J. M. Koenen, Benjamin Sauter, Anahita Aebli, Juliana G. Rando, João R. Iganci, Luciano P. de Queiroz, Daniel J. Murphy, Myriam Gaudeul, Anne Bruneau, Melissa Luckow, Gwilym P. Lewis, Joseph T. Miller, Marcelo F. Simon, Lucas S. B. Jordão, Matías Morales, C. Donovan Bailey, Madhugiri Nageswara-Rao, James A. Nicholls, Oriane Loiseau, R. Toby Pennington, Kyle G. Dexter, Niklaus E. Zimmermann, Colin E. Hughes

**Affiliations:** ^1^ Department of Systematic and Evolutionary Botany, University of Zurich, Zollikerstrasse 107, CH 8008 Zurich, Switzerland.; ^2^ Programa de Pós Graduação em Ciências Ambientais, Centro das Ciências Biológicas e da Saúde, Universidade Federal do Oeste da Bahia, Rua Prof. José Seabra de Lemos, 316, Bairro Recanto dos Pássaros, 47808-021 Barreiras-BA, Brazil.; ^3^ Instituto de Biologia, Universidade Federal de Pelotas, Campus Universitário Capão do Leão, Travessa André Dreyfus s/n, 96010-900 Capão do Leão-RS, Brazil.; ^4^ Programa de Pós-Graduação em Botânica, Universidade Federal do Rio Grande do Sul, Avenida Bento Gonçalves, 9500, 91501-970 Porto Alegre-RS, Brazil.; ^5^ Departamento Ciências Biológicas, Universidade Estadual de Feira de Santana, Avenida Transnordestina s/n, Novo Horizonte, 44036-900 Feira de Santana-BA, Brazil.; ^6^ Royal Botanic Gardens Victoria, Birdwood Ave., Melbourne, VIC 3004, Australia.; ^7^ School of Biological, Earth and Environmental Sciences, Faculty of Science, University of New South Wales, Sydney, NSW 2052, Australia.; ^8^ School of BioSciences, University of Melbourne, Melbourne, VIC 3010, Australia.; ^9^ Institut de Systématique, Evolution, Biodiversité (ISYEB), MNHN-CNRS-SU-EPHE-UA, 57 rue Cuvier, CP 39, 75231 Paris, Cedex 05, France.; ^10^ Institut de Recherche en Biologie Végétale and Département de Sciences Biologiques, Université de Montréal, 4101 Sherbrooke St E, Montreal, QC H1X 2B2, Canada.; ^11^ School of Integrative Plant Science, Plant Biology Section, Cornell University, 215 Garden Avenue, Roberts Hall 260, Ithaca, NY 14853, USA.; ^12^ Accelerated Taxonomy Department, Royal Botanic Gardens, Kew, Richmond, Surrey TW9 3AE, UK.; ^13^ Global Biodiversity Information Facility, Universitetsparken 15, DK-2100 Copenhagen Ø, Denmark.; ^14^ Embrapa Recursos Genéticos e Biotecnologia, 70770-901 Brasília-DF, Brazil.; ^15^ Programa de Pós-Graduação em Botânica, Instituto de Pesquisas Jardim Botânico do Rio de Janeiro, 22460-030 Rua Pacheco Leão-RJ, Brazil.; ^16^ Instituto de Recursos Biológicos, CIRN-CNIA, Instituto Nacional de Tecnología Agropecuaria (INTA), Hurlingham 1686, Buenos Aires, Argentina.; ^17^ Consejo Nacional de Investigaciones Científicas y Técnicas (CONICET), C1425FQB Ciudad Autónoma de Buenos Aires, Argentina.; ^18^ Facultad de Agronomía y Ciencias Agroalimentarias, Universidad de Morón, B1708JPD Morón, Buenos Aires, Argentina.; ^19^ Department of Biology, New Mexico State University, Las Cruces, NM 88001, USA.; ^20^ United States Department of Agriculture - Agricultural Research Service, Subtropical Horticulture Research Station, 13601 Old Cutler Road, Miami, FL 33158, USA.; ^21^ Australian National Insect Collection, CSIRO, Clunies Ross Street, Acton, ACT 2601, Australia.; ^22^ School of Geosciences, University of Edinburgh, Old College, South Bridge, Edinburgh EH8 9YL, UK.; ^23^ Department of Geography, University of Exeter, Laver Building, North Park Road, Exeter EX4 4QE, UK.; ^24^ Tropical Diversity Section, Royal Botanic Garden Edinburgh, Edinburgh EH3 5LR, UK.; ^25^ Department of Environmental System Science, ETH Zürich, Universitätstrasse 16, 8092 Zürich, Switzerland.; ^26^ Swiss Federal Institute for Forest, Snow and Landscape Research WSL, Zürcherstrasse 111, 8903 Birmensdorf, Switzerland.

## Abstract

Early natural historians—Comte de Buffon, von Humboldt, and De Candolle—established environment and geography as two principal axes determining the distribution of groups of organisms, laying the foundations for biogeography over the subsequent 200 years, yet the relative importance of these two axes remains unresolved. Leveraging phylogenomic and global species distribution data for Mimosoid legumes, a pantropical plant clade of c. 3500 species, we show that the water availability gradient from deserts to rain forests dictates turnover of lineages within continents across the tropics. We demonstrate that 95% of speciation occurs within a precipitation niche, showing profound phylogenetic niche conservatism, and that lineage turnover boundaries coincide with isohyets of precipitation. We reveal similar patterns on different continents, implying that evolution and dispersal follow universal processes.

## Teaser

Water availability dictates patterns of global phylogenetic beta diversity in tropical plants.

## INTRODUCTION

Ever since natural historians first observed that groups of organisms are geographically restricted (*1*–*3*)—palms to the tropics, pines to north temperate climates, extant lemurs to Madagascar, and hummingbirds to the Americas—understanding the factors that shape turnover of evolutionary lineages has been a central question in biogeography and macroevolution (*4*–*6*). While the importance of geography and environment as the main axes determining the distribution of lineages, i.e., phylogenetic turnover or beta diversity, was already established >200 years ago (*1*–*3*), their relative contributions are still keenly debated (*6*–*10*). The key factors determining turnover, phylogenetic niche conservatism (PNC) and geographic dispersal limitation (DL) (*5*, *8*), have both spatial and temporal dimensions, reflecting the spatial distribution of lineages and the evolutionary time scales over which barriers to dispersal, whether environmental (i.e., PNC) or spatial (i.e., DL), are overcome (*8*, *11*). To understand the contemporary spatial structure of diversity, turnover across space and time needs to be considered together (*6*, *8*, *11*, *12*), but this is rarely achieved in empirical studies.

For most terrestrial organisms, oceans and areas experiencing frost pose major barriers to dispersal and adaptation, resulting in high phylogenetic turnover among continents (*13*, *14*) and across the tropical-temperate divide, manifest in tropical niche conservatism (*5*, *15*, *16*). Precipitation appears to be a major axis of tropical turnover for some lineages (*5*, *17*) but not others (*8*), and the relative importance of PNC versus DL within continents is disputed (*5*, *8*). Whether phylogenetic turnover shows congruent patterns, driven by similar processes on different continents, remains unclear. Likewise, uncertainty remains about temporal turnover of lineages and the influence of PNC and DL through time (*4*, *10*, *18*). While important breakthroughs have been made in recent years [e.g., (*5*, *8*, *13*–*15*, *17*–*19*)], the lack of any more general understanding about spatial and temporal turnover is aggravated by (i) reports of lineage- and region-specific idiosyncrasies of PNC and phylogenetic turnover (*13*, *19*), which likely result from the fact that most clade-based studies have focused on relatively small clades [e.g., (*20*–*22*)]; (ii) primary focus on spatial rather than temporal turnover (*8*, *13*); (iii) strong focus on understanding the latitudinal diversity gradient (*23*), rather than turnover longitudinally across continents within latitudinal zones, where water availability gradients are just as prominent as latitudinal temperature gradients; and (iv) lack of high-resolution species occurrence data and robust phylogenies for species-rich clades.

This lack of understanding applies especially to turnover of tropical plant lineages, because almost all studies of tropical plant species turnover are geographically restricted and/or lack precise species distribution data or phylogenies (*5*, *8*). This gap in the tropics is critical given that plants are the primary producers and structural elements of terrestrial ecosystems (*23*), and the tropics are the most species-rich region of the planet (*5*, *23*). To gain a more general understanding of adaptation and turnover of woody plants across the tropics, standardized analyses of species-rich and geographically and ecologically widespread tropical lineages across continents are needed to disentangle regional and taxon effects on the drivers of phylogenetic turnover across space and time.

**Fig. 1.**
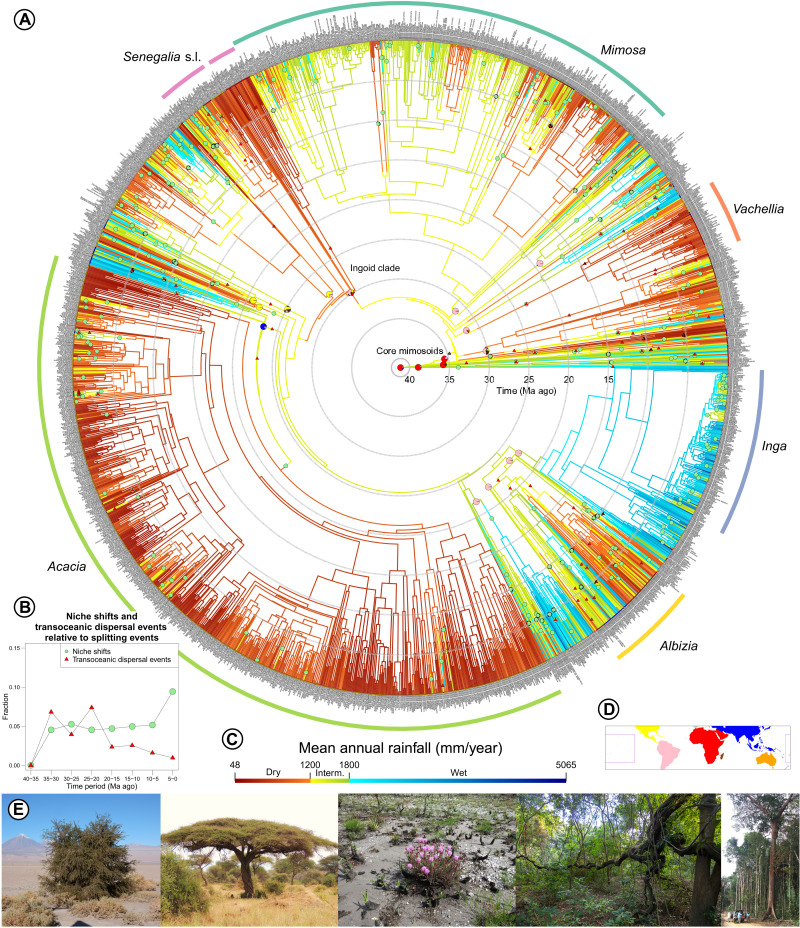
Mimosoid evolution and diversity across precipitation gradients. (**A**) Phylogeny of Mimosoid legumes showing the evolution of precipitation niches and transcontinental dispersal events through time. Branch colors correspond to mean annual precipitation (MAP) estimates [see (**C**) for scale]. Pie charts at tips and nodes of named clades [sensu (*24*)] represent observed and estimated spatial distributions [based on area definitions in (**D**)]. Ancestral niches and areas were estimated using a complete metachronogram for Caesalpinioideae, including non-Mimosoid Caesalpinioideae taxa, but only the Mimosoid clade is shown here. Green circles on branches indicate shifts between precipitation categories [following (*17*)] that encompass a difference of at least 250-mm MAP; red triangles indicate postulated transcontinental dispersals according to the best-supported model. The six most species-rich genera are labeled. (**B**) Fractions of niche shifts and transcontinental dispersal events, averaged across multiple optimizations, relative to total phylogenetic splits plotted through time for 5-Ma bins. (**E**) Mimosoid growth form diversity across the tropical precipitation gradient, from deserts with <50-mm MAP (left) through savannas to rain forests with >5000-mm MAP (right). See the Supplementary Results for species names and photographers. See fig. S51 for more information.

We investigate phylogenetic turnover through the past 45 million years (Ma) across the global lowland tropics using the Mimosoid clade of legumes. Mimosoids, originating in the Eocene (*24*), are functionally diverse, comprise c. 3500 species of trees, shrubs, geoxyles, and lianas, and make up ecologically important elements of all major lowland tropical biomes (Fig. 1E), including deserts, seasonally dry forests, savannas, and rain forests (*24*–*26*). Across tropical biomes and continents, Mimosoids constitute 5 to 17% of all Angiosperm species (see Methods and Supplementary Results) and include numerous iconic tropical tree radiations, such as wattles (*Acacia*) with >1000 species across Australia, umbrella thorn “Acacias” (*Senegalia* and *Vachellia*) with >150 species dominating African savannas, and ice-cream bean trees (*Inga*), a model system for Amazonian rain forest diversification with c. 300 species (*25*). The pantropical ubiquity of Mimosoids across all lowland tropical environments, mirroring that of arborescent flowering plants as a whole, makes this an ideal clade for elucidating general patterns of phylogenetic turnover for plants. Furthermore, quantifying PNC and DL across a clade of several thousand species avoids the pitfalls surrounding the potential nonrepresentativity of results when studying small clades.

We deploy large, high-resolution, global-scale taxonomic, phylogenomic, and geographic distribution datasets based on expert-curated plant material collected during targeted fieldwork in key tropical biodiversity hotspots over the past two decades, and leaf tissue and species occurrence data from specimens sampled across the world’s herbaria. Using these newly generated phylogenomic, taxonomic, and occurrence datasets, we assess (i) whether turnover is primarily structured by DL or PNC, and which environmental factors determine PNC; (ii) what levels of PNC versus niche shifting and DL versus long-distance dispersal through time characterize a species-rich clade that has fully colonized the lowland tropics; (iii) whether patterns and drivers of turnover are similar across continents, implying general processes shaping turnover, or whether phylogenetic turnover is primarily driven by taxon and area-specific idiosyncrasies; and (iv) whether the tempo of lineage diversification through time is associated with global Cenozoic climate change.

## RESULTS AND DISCUSSION

### Phylogeny and distribution of Mimosoids

We generated a robust, time-calibrated backbone phylogeny based on sequences of 997 nuclear genes (*24*) for 99 of 100 Mimosoid genera and 420 species (figs. S1 to S33). We combined this well-resolved backbone with 15 species-level phylogenies derived from diverse DNA sequence data types to generate a species-level metachronogram for 1860 species (58% of Mimosoids) (Fig. 1 and fig. S34). Using a newly compiled taxonomic checklist, we assembled 424,333 quality-controlled species occurrence records (fig. S35) to derive climate niches for 93% of Mimosoid species. We show that Mimosoid species span a 100-fold range in mean annual precipitation (MAP) from <50 mm in Somalia (*Vachellia qandalensis*), the Atacama Desert of Chile (*Strombocarpa tamarugo*), and the Namib Desert (*Senegalia montis-usti*) to >5000 mm in the hyper-wet rain forests of the Colombian Chocó (*Zygia dissitiflora*) and the Indian Western Ghats (*Archidendron monadelphum* var. *gracile*) (Fig. 1).

### DL or PNC?

**Fig. 2.**
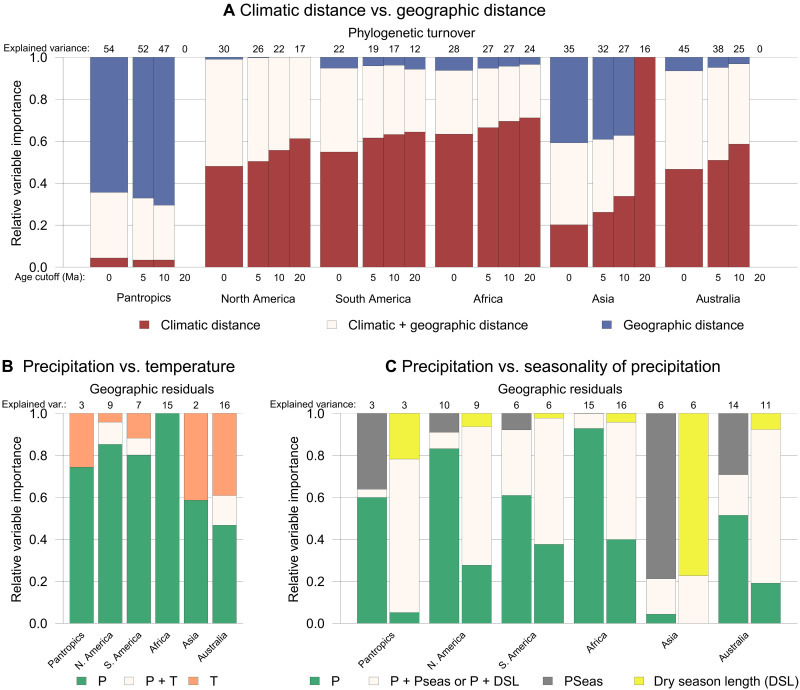
Drivers of phylogenetic turnover of Mimosoid legumes across the global lowland tropics. Bars show relative fractions of phylogenetic turnover explained by predictors (rescaled to add up to one). Numbers above bars are absolute explained percentages of turnover (tables S12 and S20). (**A**) Phylogenetic turnover explained by climatic distance (maroon), geographic distance (blue), or their interaction (cream). Turnover is assessed across four depths in the phylogeny: with the full metachronogram (age cutoff of 0) and with all clades younger than 5, 10, and 20 Ma collapsed. Note that it was not possible to fit a model to the phylogeny collapsed at 20 Ma for the pantropical and Australian models. (**B**) Phylogenetic turnover explained by MAP (green) and/or annual mean temperature (orange). Turnover is expressed as phylogenetic turnover not explained by geographic distance (“geographic residuals”). (**C**) Geographic residuals of phylogenetic turnover explained by MAP (green) and/or precipitation seasonality (gray; left) or dry season length (DSL) (i.e., the number of consecutive months with precipitation < 100 mm/month; yellow; right). See fig. S45 for results obtained with an alternative, genus-level Mimosoid phylogeny. P, MAP; T, annual mean temperature; Pseas, precipitation seasonality.

Pantropically, geographic distance explains a much greater fraction of Mimosoid phylogenetic turnover than climatic distance (Fig. 2 and fig. S45), in line with DL caused by oceanic barriers as the most important factor shaping the global distribution of lineages (*13*, *14*). Nevertheless, despite high DL, transoceanic dispersal has been important in shaping the distribution of Mimosoids: Transcontinentally distributed Mimosoid clades are common, including five pantropical genera (*Entada*, *Vachellia*, *Neptunia*, *Senegalia*, and *Parkia*). Overall, 60 (±27) transoceanic disjunctions were inferred across the phylogeny (Fig. 1 and fig. S47). Given the Early to Mid-Eocene crown age of Mimosoids (Fig. 1), these disjunctions and the strong geographical structuring of global Mimosoid phylogenetic turnover are explained not by vicariance (*27*) but by stochastic sweepstakes long-distance dispersal (*28*) followed by in situ diversification within continents, against a backdrop of DL. The percentage of phylogenetic nodes associated with transoceanic dispersal, a measure of DL, varies from 6.5% (±3.4%) between 20 and 35 Ma ago to 2% (±0.9%) in the past 20 Ma (Fig. 1 and table S24), suggesting that an early burst of Mimosoid dispersal established their pantropical distribution.

Contrary to the high phylogenetic turnover among continents, within four of the five continents spatial distance plays a minor role in explaining turnover (Fig. 2A). The exception is within Asia, likely because the greater climatic homogeneity and numerous island archipelagos across Southeast Asia favor DL-driven turnover. On all other continents, climatic distance has a much larger effect than spatial distance (Fig. 2A), suggesting relatively infrequent adaptation to different climatic conditions compared to within-continent dispersal. In other words, for Mimosoids within continents, it has been “easier [for lineages] to move than to evolve” (*11*), implying strong PNC (*5*, *8*) and little DL. Although taxonomic and phylogenetic turnover are strongly correlated (tables S12 and S13), our results reflect the turnover not just of species but also lineages (tables S12 to S15) (*19*). The trend on every continent toward a higher contribution of climatic distance compared to geographic distance in explaining turnover at deeper evolutionary levels (Fig. 2A) shows that the impact of climatic distance is not due just to species and shallow lineage turnover (Fig. 2A) (*29*). This suggests that, in the past, climatic tolerances evolved even less frequently relative to lineage diversification and within-continent dispersal (*8*), although uncertainty increases for reconstructions further back in time. Furthermore, even when covariation between climatic and geographic distance is accounted for by analyzing the residuals of a linear regression of phylogenetic turnover with geographic distance (“geographic residuals”) (*30*, *31*), climatic distance still explains a considerable fraction of phylogenetic turnover (Fig. 2, B and C, and table S20). This contrasts with the geographic residuals of global mammalian phylogenetic turnover, which cannot be explained by climatic distance (*30*), indicating a stronger impact of climate on the distributions of vascular plants than mammals. Nevertheless, despite this dominant role of climate in dictating turnover, climate and geography alone are not sufficient to explain all Mimosoid phylogenetic turnover (Fig. 2 and table S20), as found in other plant clades (*7*, *8*). Other factors such as fire, soil, and herbivory also likely shape distribution patterns (*20*, *32*).

**Fig. 3.**
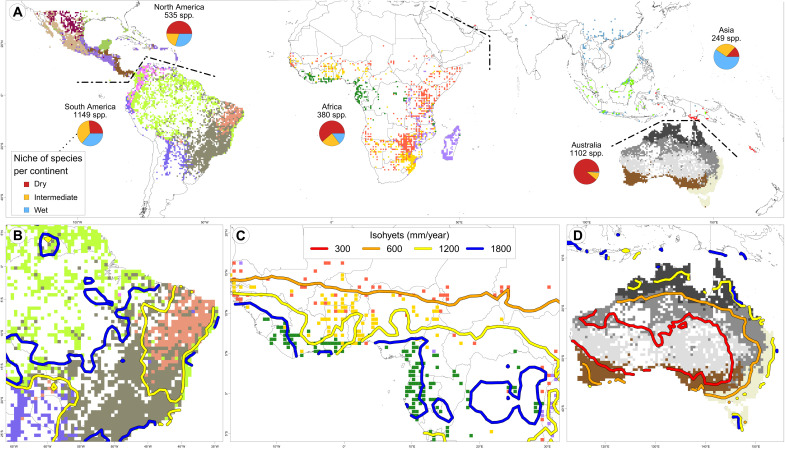
Phyloregionalization of Mimosoids. (**A**) Phylogenetic regionalization of Mimosoid legumes within continents and correspondence between phyloregion boundaries and isohyets in (**B**) eastern South America, (**C**) West Africa, and (**D**) Australia. Colored map cells denote phyloregions but are not indicative of phylogenetic proximity between regions; dashed lines indicate borders between continents and regionalizations. Pie charts (A) show niche distributions of species (categories follow Fig. 1). See Figs. S36 to S41 and S44 for results of pantropical clustering, different numbers of phyloregions, and clusters obtained using ancient turnover, geographic residuals, and a genus-level Mimosoid phylogeny.

Drivers of turnover are similar across continents: Compared to precipitation, temperature explains only a small fraction of phylogenetic turnover (Fig. 2B), which is likely because Mimosoids are largely confined to <2500m elevation in the tropics. Whether total annual precipitation or precipitation seasonality is more important in driving turnover is less clear and dependent on how seasonality is quantified (Fig. 2C and table S20). This underlines the wide distribution of Mimosoids across gradients of both precipitation and precipitation seasonality (*33*), as well as the importance of investigating multiple indices of environmental parameters. Although the distribution of Mimosoids across precipitation niches varies across continents, reflecting differences in available niche space (Fig. 3), our results (Fig. 2) strongly suggest that the evolution and dispersal of Mimosoids across continents followed universal processes and drivers associated with water availability.

Two additional lines of evidence suggest that the evolution and distribution of Mimosoids are structured by precipitation. First, we observe high phylogenetic signal of MAP and dry season length (DSL) [Pagel’s lambda (*34*) of 0.88 and 0.84, respectively, with 1 being the maximum], both considerably higher than the phylogenetic signal of MAP measured across 1100 lowland tropical tree genera in South America (i.e., lambda of 0.5) (*17*). Second, spatial clustering of phylogenetic turnover via phyloregionalization analyses (*29*), estimated independently of climate, reveals that, on most continents, higher-level clusters, representing the deepest phylogenetic divergences between areas, involve phyloregions characterized by large water availability differences (Fig. 3 and figs. S36 to S44). Many lower-level clusters, i.e., geographic areas that are less phylogenetically divergent, are also characterized by different precipitation regimes (fig. S43) and their boundaries broadly coincide with isohyets of precipitation and major vegetation types (Fig. 3). For example, in eastern South America, phyloregions defined solely by Mimosoid turnover closely match the seasonally dry Caatinga, Cerrado (savanna), Chaco dry woodland, and Amazonian and Mata Atlântica tropical rain forests (Fig. 3) (*35*), supporting regional analyses (*33*). Nevertheless, biomes per se provide a poor explanation of phylogenetic turnover at continental or transcontinental scales, likely caused by relatively high turnover between geographically disjunct areas of the same biome (tables S22 and S23). For instance, the Brazilian Caatinga, Colombian dry inter-Andean valleys, and north coastal Venezuela are considered part of the same seasonally dry tropical forest biome (*20*, *35*), and while the phyloregionalization separates these areas from adjacent wetter regions, they do not group together in the same phyloregion (Fig. 3). The correspondence between isohyets and phyloregional boundaries is not perfect nor equally compelling in all regions (fig. S42), again indicating the importance of other factors shaping vegetation patterns (*32*). Nevertheless, the overall similarity between precipitation regimes and phyloregions is notable, providing additional evidence that precipitation is the main driver of evolutionary turnover of plant lineages within the tropics.

### PNC through time

The prevalence of PNC related to precipitation demonstrates that precipitation gradients strongly influence the evolution and distribution of diversity within continents (*5*, *15*, *36*), yet Mimosoids were able to overcome adaptive barriers associated with water availability to occupy all tropical lowland climates and diversify as ubiquitous components of dry and wet biomes (Figs. 1 and 3) (*24*–*26*, *33*), showing considerable climatic niche evolution. This apparent paradox between high PNC and niche evolvability suggests that it is the balance between PNC and niche evolution that is central to explain turnover. We show that throughout the evolutionary history of Mimosoids c. 5% of speciation events involved a shift in precipitation regime, regardless of whether MAP (Fig. 1) or DSL is used (fig. S46). This rate remained constant (Fig. 1), until apparently increasing in the past 5 Ma, although this may be due to undersampling of taxa within precipitation regimes toward the tips of the phylogeny. Thus, while many more species resulted from diversification within a precipitation niche (i.e., 95%) than via adaptation across precipitation regimes (i.e., 5%), we document 262 shifts in precipitation niche over the past 45 Ma. Many of these shifts spawned species-rich clades confined to wet or dry niches: For example, rapid diversification of the c. 385 species in the Inga clade (*Inga* and closely allied genera) followed a shift into wet neotropical rain forests c. 17 Ma ago; similarly, *Acacia* with >1000 species largely in dry areas of Australia followed dispersal and a niche shift to drier climates c. 23 Ma ago (Fig. 1). Several monospecific or species-poor evolutionarily-isolated Mimosoid lineages [“depauperons” sensu (*37*)], such as the monospecific genera *Xerocladia* and *Mezcala* confined to arid climates, or *Cedrelinga* and *Wallaceodendron* confined to rain forests, have likely persisted for many millions of years in restricted rainfall niches, also providing additional evidence for PNC, although other depauperon lineages appear to be ecologically more widespread. The Mimosoid-wide 5% average of niche shifts masks considerable heterogeneity in niche specificity within subclades, with some genera like *Mimosa* and *Albizia* more labile than others (Fig. 1), highlighting the importance of phylogenetic scale for assessing PNC (*38*, *39*).

Lack of adaptation to different temperature regimes contrasts with findings for precipitation. No diverse extratropical radiations are evident in Mimosoids, and only 7.2% of Mimosoids occur outside the tropics (i.e., just 270 taxa in 30 genera have more than half their occurrences in areas that experience frost in an average year). Using this very strict definition of the tropics that excludes many subtropical areas, e.g., parts of central Mexico (*40*), and therefore likely inflates the numbers of extratropical species and transitions, across Mimosoids we document just 43 evolutionary transitions out of the tropics to geographically juxtaposed frost-prone temperate zones (fig. S50), indicating strong tropical niche conservatism (*5*, *16*). This suggests that the adaptive barrier posed by frost is just as formidable or potentially even stronger than that posed by transoceanic dispersal barriers, providing further evidence of the importance of PNC and the environment in dictating the turnover of clades. As in many other pantropical arborescent plant lineages (e.g., Burseraceae, Malpighiaceae, and Meliaceae), strong tropical PNC in Mimosoids is likely, in part, because they never evolved annual life cycle facilitating adaptation to temperate climates (*41*).

Just how prevalent PNC is across the tree of life and how it influences diversity patterns are heavily debated (*5*, *8*, *11*, *38*). An important factor that influences assessment of PNC is phylogenetic scale (*38*, *39*), yet almost all estimates of PNC are from studies of small clades or geographically confined floras or are based on poorly resolved phylogenies (*5*, *20*, *21*, *36*). Here, in a large pantropical clade of c. 3500 species, we show that the evolution and distribution of Mimosoids were profoundly shaped by PNC, demonstrating that conservatism does structure larger, older clades that are ecologically diverse, even when their constituent lineages vary substantially in how niche-conserved they are (Fig. 1). Our 5% estimate for niche shifts through time closely matches estimates from continental floras, such as 4% shifts in the Southern Hemisphere (*21*) and 7% in tropical Africa (*36*). Theoretical models of evolutionary diversification suggest that intermediate levels of PNC and niche evolution result in the highest species richness (*18*, *42*). Under these models, overly high PNC limits geographic distributions, resulting in high extinction and/or limited speciation; conversely, a too high prevalence of niche evolution results in smaller numbers of more widespread species (*18*, *42*). We suggest that our observed 20:1 PNC-to–niche evolution ratio, i.e., repeated individual niche colonizations followed by in situ diversification of niche-conserved and often species-rich clades, ultimately resulting in c. 3500 species across the global tropics, provides a compelling empirical estimate of optimal PNC, corroborating theoretical model predictions (*18*, *42*) and other empirical observations (*21*, *36*). Thus, while potent examples of PNC or niche evolution have been derived from numerous studies of small clades (*20*–*22*, *43*), PNC-to–niche evolution ratios need to be assessed across species-rich, ecologically diverse clades to test the ubiquity of this 20:1 ratio, as we do here for Mimosoids.

### Lineage diversification dynamics through time

Quantifying turnover of lineages through time and assessing whether, like spatial turnover, episodes of extinction and speciation are also intimately linked to climate are more challenging than quantifying spatial turnover because of the difficulties of estimating extinction rates. To account for these difficulties, we estimated speciation rates through time in Mimosoids across various levels of temporal turnover implied by a wide range of fixed extinction rates. These analyses show that, while the center of gravity of speciation moves from the tips to deeper in the phylogeny under increasing extinction rates (fig. S48), there is considerable diversification rate heterogeneity among lineages and through time across all extinction rates (fig. S49). Notably, across almost all potential extinction rates, we find a marked increase in speciation rate in the Late Eocene or at the Eocene-Oligocene transition (c. 34 Ma ago; fig. S49). This acceleration in speciation coincides with the transition from “Warmhouse” to “Coolhouse” conditions, the most prominent Cenozoic climate transition that coincided with the formation of ice sheets in Antarctica (*44*). This acceleration in speciation also coincides with the evolutionary transition from typically large tree and liana genera of African and Asian humid forests to a series of dry-adapted lineages in the core Mimosoid clade, originating in the Oligocene (34 to 23 Ma ago; Fig. 1) and distributed across the tropics as a whole. This prominent transition from slower to faster diversification thus appears to be associated with environmental change, specifically with cooling and global expansion of dry habitats in the Oligocene and Miocene (*45*). Elevated net diversification rates were sustained through this Coolhouse period of global aridification (*45*) until the Middle to Late Miocene (c. 13 to 8 Ma ago; fig. S49), notably by a set of arid radiations, especially of *Acacia* with >1000 species in Australia, and diversification along the mainly dry-adapted backbones of the core Mimosoid and ingoid clades (Fig. 1 and fig. S48). More recent Mimosoid evolutionary radiations are ecologically diverse and geographically scattered, including both wet forest lineages such as *Archidendron* (Southeast Asian rain forests) and the Jupunba and Inga clades (both neotropical and predominantly in Amazonian rain forests) (Fig. 1 and fig. S48), as well as the mostly arid synchronous radiations of the three largest clades of Madagascan Mimosoids in the past c. 15 to 10 Ma (Fig. 1 and fig. S31).

Radiations in the Mimosoid clade, characterized by nodes showing elevated gene tree conflict and very short phylogenetic branches (fig. S31), are thus temporally and geographically scattered and episodically nested across the phylogeny (fig. S48). They include more ancient episodes of apparently near-simultaneous lineage diversification [e.g., the ingoid and *Archidendron* clades with putative hard polytomies (*24*, *26*)], as well as more recent nested Late Miocene and Pliocene Madagascan and Amazonian radiations (fig. S31). This pattern of episodic radiations is potentially consistent with a model of episodic species turnover (*4*), as suggested by the high diversity of non-Mimosoid Caesalpinioid fossil legumes in the Eocene of North America (*46*) followed in the Oligocene by fossil assemblages rich in core Mimosoid lineages from Puebla in Mexico (*47*, *48*). The regionally heterogeneous opportunities provided by global climate change (*10*) and long-distance dispersal, in combination with the ability of Mimosoids to diversify within and across precipitation niches (Fig. 1), have repeatedly generated phylogenetically and geographically scattered rapid radiations across water availability gradients, ultimately leading to the current pantropical prevalence of Mimosoids.

The prevalence of PNC suggests that the world is crossed by sets of major environmental adaptive barriers [e.g., to drought (*20*), frost (*5*), or salinity (*49*)] that can be just as formidable as geographic dispersal barriers. In some cases, these adaptive barriers are apparently even more difficult for plant lineages to surmount than transcontinental oceanic dispersal barriers, further emphasizing the central importance of PNC at continental and transcontinental scales. Understanding these barriers and their relative permeabilities is important not only to understand the distribution of lineages across the globe, as we show here, but also to examine the role of plant functional traits and their evolution in shaping diversity patterns (*26*, *50*) and to define the broad framework and macroevolutionary context for understanding the fundamental population-level processes underpinning evolutionary adaptation.

## MATERIALS AND METHODS

Unless stated otherwise, all data handling and analyses were performed in R (*51*).

### Phylogenetic inference

To construct a phylogeny that is robustly supported and densely sampled in terms of taxa, we combined a newly generated Hybseq backbone phylogeny with a set of 15 newly generated, taxonomically enhanced, or previously published species-level phylogenies for particular subclades. The backbone phylogeny was constructed from DNA sequence data generated via targeted enrichment of 997 nuclear genes selected specifically for phylogenomics of Mimosoid legumes [Mimobaits (*24*), https://github.com/erikkoenen/mimobaits]. For the backbone, data were generated for 420 species representing 158 of the 163 genera in subfamily Caesalpinioideae, which contains the Mimosoid clade, and sampling taxa spanning the root nodes of each of the species-level subclades. This large phylogenomic dataset ensures that the backbone phylogeny is as robustly supported as possible and facilitates high-precision time calibration using a subset of informative and clock-like genes. The 15 species-level phylogenies (hereafter referred to as subtrees) were constructed using a range of DNA sequence data types (Hybseq, RADseq, and traditional Sanger-sequenced loci), either newly generated or from published data, and appropriately rooted. Densely sampled ultrametric subtrees for these 15 subclades were grafted onto the time-constrained backbone phylogeny. We refer to the resulting time-calibrated phylogeny as a metachronogram, based on the earlier idea of meta-trees (*52*, *53*), as implemented by Spriggs *et al*. (*54*) to build a phylogeny for grasses. One potential limitation of the metachronogram approach is that combining different data types and different clock models for the backbone and subtrees could affect subsequent diversification rate analyses, but these impacts are likely to be minor and manifest only at finer scales. This potential limitation is outweighed by several important advantages, i.e., that diverse data types can be combined to build a single time-calibrated phylogeny, thereby tapping into the full wealth of available DNA sequence data in a flexible way to maximize the number of taxa that can be sampled with molecular data. The metachronogram also provides a computationally tractable way to build a large phylogeny with many taxa.

#### Phylogenomic backbone

##### Taxon sampling, hybrid capture, and sequencing

We extracted DNA from herbarium specimens and silica-dried leaf samples of 284 taxa (table S1) using the DNeasy Plant Mini Kit (Qiagen, Venlo, The Netherlands). DNA integrity and concentration of a subset of extractions from older herbarium specimens were checked on a 4200 TapeStation System using D5000 ScreenTape (Agilent Technologies, Santa Clara, USA). Library preparation, hybrid capture, enrichment, and sequencing were performed by Arbor Biosciences (previously Mycroarray, Ann Arbor, USA). For the capture, a custom bait set of 1 Mb, targeting 997 nuclear genes, was used. This bait set was specifically designed for phylogenomic analyses of the Mimosoid clade of subfamily Caesalpinioideae [sensu (*55*)] of the legumes by Koenen *et al*. (*24*). To generate the bait set, a custom pipeline was used to select putatively single-copy genes from transcriptomes of four genera (*Albizia julibrissin*, *Entada abyssinica*, *Microlobius foetidus*, and three species of *Inga*) spanning the Mimosoid clade (*24*). During the capture reactions, 25 libraries were pooled on the basis of approximate evolutionary distances between taxa. Sequencing was performed on Illumina HiSeq 4000, resulting in 150–base pair (bp) paired-end reads. In addition to these newly generated sequences, we also used the raw reads of the same genes from 122 Caesalpinioideae taxa previously generated by Koenen *et al*. (*24*) and 18 *Inga* species generated by Nicholls *et al*. (*56*). Together, this resulted in a dataset of 424 taxa, covering 161 of the 163 genera of subfamily Caesalpinioideae, including 99 of 100 Mimosoid genera (table S1). The two missing genera are *Stenodrepanum*, the monospecific sister genus of *Hoffmannseggia* (*57*), and the Mimosoid *Microlobius*, which is also monospecific and closely related to *Gwilymia* and *Stryphnodendron* (*58*). Last, sequences for as many of the 997 genes as possible were extracted from published genomes of the five taxa from the legume subfamilies Cercidoideae and Papilionoideae (table S2) using BLAST (*59*) and BLAT (*60*) to serve as outgroups. Full details about the data cleaning, target assembly, and orthology assessment are presented in the Supplementary Methods.

##### Phylogenetic analyses

We applied coalescent and concatenation approaches to infer several species trees using all three sets of gene alignments (see Supplementary Methods). For the coalescent approach (fig. S3), all sequences shorter than 300 bp and at the same time shorter than half the total alignment length of the nucleotide alignments were trimmed. Using these cleaned alignments, final gene trees were inferred using RaxML with the GTRGAMMA model and 200 rapid bootstrap replicates. On the basis of Koenen *et al.* (*24*) and visual inspection of the resulting gene trees, all trees with a root-to-tip length variance over 0.009 were excluded from the dataset to remove outlier gene trees that may be affected by alignment or orthology inference issues. Following Mirarab (*61*), branches with bootstrap support lower than 10% were collapsed. Gene trees were used to infer three separate species trees with ASTRAL-III 5.7.3 (fig. S3) (*62*): one using just the single-copy genes, one using the single-copy genes plus the multicopy genes treated as single-copy (i.e., without paralogs), and one using the single-copy genes plus the multicopy genes with paralogs. The maximum likelihood and Bayesian approaches used to generate species trees based on concatenated gene alignments are described in the Supplementary Methods, as well as the method to infer a chloroplast phylogeny.

##### Assessing gene tree conflict and topological congruence

Conflict among single-copy gene trees was assessed in several ways. Absolute numbers of the single-copy gene trees (with a root-to-tip length variance < 0.009) supporting and conflicting each bipartition in the single-copy genes ASTRAL species tree were calculated using PhyParts (*63*). PhyParts was also used to calculate bipartition-based Internode Certainty All (ICA) values on the same tree. For both analyses, only gene tree nodes with >50% bootstrap support were considered, following Smith *et al.* (*63*). Because bipartition-based calculations of support and conflict can be affected by missing taxa in gene trees (*64*), quartet-based extended quadripartition internode certainty values were also calculated using QuartetScores (*64*).

Congruence between trees was expressed in Robinson-Foulds distance (*65*) calculated with phangorn’s (*66*) “treedist” function. Topological comparisons of all tree pair combinations were made using phytools’ (*67*) “cophylo” function.

ASTRAL’s polytomy test (*62*) was used to assess the probability and locations of potential polytomies on the ASTRAL species tree based on the single-copy genes with a root-to-tip length variance < 0.009. Rogue taxa were identified by using RogueNaRok (*68*) to compare all six sets of nuclear RaxML bootstrap replicate trees to three reference trees (the relevant RaxML best tree, a strict consensus tree, and a majority rule consensus tree) with three dropset sizes (from one to three; analyses were repeated with each dropset size until no additional rogue taxa were identified), resulting in 54 unique RogueNaRok analyses.

#### Time calibration

The ASTRAL single-copy gene topology of the Caesalpinioideae Hybseq backbone phylogeny was time-scaled in BEAST v. 1.8.4 (*69*, *70*), i.e., the topology was constrained, while BEAST was used to calculate the branch lengths, using a fixed local clock model with six local clocks to account for discrete branch length variation observed across the tree. The fixed local clock is a good compromise between relaxing the clock to account for rate heterogeneity and having the data influence the branch lengths and divergence times. In other words, we are not “over-relaxing” the clock such that branch lengths and divergence times are mostly estimated from the priors rather than the molecular data (*71*, *72*). From the Hybseq dataset, 100 gene alignments were selected using SortaDate (*73*) as follows: (i) All genes with less bipartition correspondence to the ASTRAL topology than the median were discarded; (ii) all genes with total tree length shorter than the median were discarded; and (iii) the 100 most clock-like genes, i.e., those with the least root-to-tip length variance, were selected. These 100 genes were concatenated and analyzed in a single partition with a GTR + GAMMA model. To calibrate the tree, seven fossil calibrations were used with minimum ages set as listed in table S3, using uniform priors and maximum ages set at 66 Ma ago. In addition, the root node (i.e., the crown node of the Leguminosae) was calibrated using a normal prior at 66 Ma ago, with a SD of 0.1, as this was shown to be the likely approximate crown age of the family by Koenen *et al*. (*74*). The fossils and their minimum age calibrations are discussed in detail by Lavin *et al*. (*27*), Bruneau *et al*. (*75*), Simon *et al*. (*76*) (Supplementary Materials), and Koenen *et al.* (*74*) (Supplementary Appendix).

The analyses were carried out using two chains of 50 million generations each, from which subsequently 10,000 trees of each chain were sampled and used to estimate 95% highest posterior density (HPD) age intervals. A further subsample of 1000 trees was used as the backbone set to create a set of 1000 metachronograms, in combination with 1000 post–burn-in trees of each subtree.

#### Metachronogram

As indicated above, to generate a densely sampled time-calibrated phylogeny, we grafted a set of densely sampled subtrees, i.e., phylogenies of subclades and genera, onto the Hybseq time-calibrated backbone phylogeny described above. Because the backbone tree and the grafted subtrees were derived from data sets that used different markers, both the backbone and the subtrees were estimated as ultrametric trees to make the branch lengths compatible. The subtrees were then rescaled according to the corresponding node in the time-calibrated backbone onto which it was subsequently inserted. A Python script to carry out this procedure is available at https://github.com/erikkoenen/metachronogram and can be used to produce similar metachronograms for other clades where a backbone and subtree phylogenies with (potentially) nonoverlapping markers are available. Full details about the data, samples and methods used to generate the subtrees are presented in the Supplementary Methods.

### Taxonomic checklist and species occurrence dataset

#### Mimosoid clade

To assemble an accurate quality-controlled species occurrence dataset, we first compiled a comprehensive taxonomic checklist of accepted names with partial synonymy for all species and infraspecific taxa in the Mimosoid clade. For each genus or clade, the most recent taxonomic monograph or revision (when available) was used, and more recently described taxa were added using the International Plant Names Index (www.ipni.org). This checklist was used to download species occurrence records from the Global Biodiversity Facility (GBIF; www.gbif.org), the Latin American Seasonally Dry Tropical Forest Floristic Network (DryFlor; www.dryflor.info), and the Southwestern Environmental Information Network (http://swbiodiversity.org/seinet), as well as various other taxon- or region-specific sources. Extensive data cleaning was performed: We updated synonymous names using the checklist and removed records not based on vouchered herbarium specimens (except for DryFlor records based on plot data or checklists), records located in the sea or on country or major area centroids, cultivated records, and records located outside native distribution ranges as delimited in the primary taxonomic literature.

For some taxa, precompiled occurrence datasets were used. For example, for the vast majority of the largest Mimosoid genus, the predominantly Australian genus *Acacia* that comprises c. 1000 species, we did not assemble a custom checklist and occurrence data but instead relied on the occurrence dataset of González-Orozco *et al*. (*77*) with minor updates [see Appendix 1 (data S1) for details]. Literature used to assemble the taxonomic checklist and perform quality control of the occurrence data, as well as GBIF DOIs, taxon-specific notes, and other sources of occurrence data, are in Appendix 1 (data S1).

#### Non-Mimosoid Caesalpinioideae

For the optimizations of climate and geography across the metachronogram, the 78 outgroup taxa across Caesalpinioideae outside the Mimosoid clade in the phylogenomic backbone were included to mitigate against the tendency to reconstruct intermediate trait values across deeper ancestral nodes in quantitative trait optimizations and hence improve the accuracy of ancestral area and climate reconstructions across the early nodes of the Mimosoid clade. We assembled an occurrence dataset for the 78 non-Mimosoid Caesalpinioideae taxa present in the phylogeny, using published (*20*, *78*) and newly assembled occurrence data in the same way as for the Mimosoid clade (see above). For each outgroup taxon, literature, GBIF DOIs, data sources, and notes are in Appendix 1 (data S1).

#### Abundance of Mimosoids across biomes

We estimated the fraction of Mimosoids across biomes and continents using several datasets.

The fraction of Mimosoid species among all Amazonian tree species was estimated in two ways: using appendix S1 of ter Steege *et al.* (*79*) and dataset S01 of Cardoso *et al.* (*80*). For the fraction of Mimosoid species among neotropical dry forest trees, the occurrence dataset of DryFlor (downloaded from www.dryflor.info/data/datasets) (*81*) was used. The fraction of Mimosoid species among African savanna trees was assessed using appendix S1 of Fayolle *et al.* (*82*). Last, the fraction of Mimosoid species among all native Australian Angiosperms was calculated using a species checklist from the Australasian Virtual Herbarium (https://avh.chah.org.au/).

### Phylogenetic turnover across space

#### Phylogenies

Phylogenetic turnover was calculated using the maximum clade credibility (MCC) tree of the Mimosoid metachronogram. All non-Mimosoid taxa, multiple occurrences of the same taxon, and taxa lacking occurrence data were removed, leaving a total of 1940 unique taxa, including infraspecific taxa.

To test the robustness of our results using a phylogeny with almost all species of Mimosoids, we repeated the spatial turnover analyses using a genus-level Mimosoid phylogeny. The ultrametric Caesalpinioideae phylogeny was used as a backbone topology, from which all non-Mimosoid taxa were removed and Mimosoid species added as genus-level polytomies, resulting in an ultrametric Mimosoid phylogeny with 3165 species, i.e., almost all Mimosoid species (a few missing species could not be placed with confidence due to generic nonmonophyly). Because of the dense taxon sampling in the backbone phylogeny, most genus-level polytomies could simply be added on the crown node of the genus. However, some smaller genera are represented by just a single tip in the backbone, and for those, the genus-level polytomy was added halfway along the terminal branch of the genus. The genus-level phylogeny does not include infraspecific taxa, and for analyses using this tree, occurrence data for infraspecific taxa were amalgamated into their inclusive species. Thus, compared to the metachronogram, this genus-level tree used for the robustness test has a higher number of species but lacks phylogenetic resolution within genera.

#### Phylogenetic turnover

Phylogenetic turnover across the global tropics was quantified using the phylogenetic version of Simpson’s pairwise dissimilarity index (*83*–*85*). Also known as (phylo)beta-sim or βsim (*83*, *86*), Simpson’s dissimilarity index quantifies the “true” turnover component of Sørensen’s dissimilarity index (*83*, *84*) and has the important advantage of not being influenced by differences in species richness between sites (*83*, *84*, *86*, *87*).

The “phylo.beta.pair” function of the betapart R package (*88*) was used to calculate pairwise phylogenetic turnover between all half-by-half degree longitude/latitude grid cells with at least three (metachronogram) or five (genus-level tree) taxa located at the most 33° latitude north or south of the equator, i.e., restricting assessment of phylogenetic turnover patterns to the global tropics and subtropics. Temperate regions were excluded because Mimosoids show high tropical niche conservatism (*25*): Only 31 taxa (i.e., 0.83% of all taxa in the occurrence dataset), belonging to *Neltuma* (13 taxa), *Mimosa* (six taxa), *Desmanthus* (three taxa), *Strombocarpa*, *Albizia*, *Prosopidastrum*, *Senegalia* (each two taxa), and *Calliandra* (one taxon), have over half their occurrence points north or south of 33° latitude in North or South America, Africa, the Middle East, or Asia, and these climatic and geographic outliers are likely to strongly bias explanations of patterns of phylogenetic turnover in these regions. The only exception to this is Australia, where 288 taxa of *Acacia* and one taxon of *Paraserianthes* (i.e., 7.70% of all Australian taxa) have over half of their occurrence points south of 33° latitude, and phylogenetic turnover was therefore quantified across all of Australia, including Tasmania.

#### Predictor variables

Geographic (great circle) distances between centers of grid cells were calculated using the “RdistEarth” function of the fields package (*89*), or internally in the “gdm” function of the gdm package (*90*).

Climatic distances between grid cells were calculated using all 19 Bioclim variables of CHELSA (*91*), cloud cover, and the intra-annual SD of cloud cover downloaded from EarthEnv.org (*92*), plus DSL, i.e., the number of consecutive months with rainfall <100 mm/month (*20*). Predictor variables were aggregated to a half degree resolution using the “aggregate” function of the raster package (*93*).

#### Explaining phylogenetic turnover

Generalized dissimilarity modeling (GDM) provides a powerful technique to analyze and explain patterns of spatial turnover (*94*, *95*). It offers several advantages over other approaches, including fitting nonlinear relationships between turnover and predictor variables (*94*, *95*), which provides a more realistic way to assess complex biological patterns than strictly linear relationships (*12*). GDM also allows precise quantification of the effect of each individual predictor variable on turnover patterns (*13*). GDMs were run using the “gdm” function of the gdm package (*90*) with default settings.

#### Hypothesis testing

Hypotheses were tested using variation partitioning (*7*, *8*, *13*), whereby, to tease apart the unique and combined effects of hypothetical predictors A and B, we compared the effects of GDMs run with predictors A and B together, just with A, and just with B.

To tease apart the influence of DL and PNC, we quantified the fraction of global phylogenetic turnover explained by geographic distance and by climatic predictors. This was done on a pantropical scale (excluding temperate areas) and at the level of individual continents: North America (including Mesoamerica and the Caribbean), South America, Africa (including Madagascar and Arabia), Asia, and Australia. Exploratory analyses show that using other definitions of continents, e.g., Africa without Madagascar and Arabia, and Australia without its temperate regions, does not have a large impact on the results (results not shown).

Next, we ran a simple linear regression of phylogenetic turnover with geographic distance. The residuals of this model, hereafter referred to as geographic residuals, represent the fraction of phylogenetic turnover that is not explained by spatial distance (*30*, *31*). Using these geographic residuals ensures that we are not mistakenly assigning environmental explanations to phylogenetic turnover patterns that are actually driven purely by spatial distance (*96*). Geographic residuals were used for two additional rounds of variation partitioning, at both pantropical level and at the level of the five individual continents: quantifying the fraction of phylogenetic turnover explained by MAP (Bio12) and annual mean temperature (Bio1), and quantifying the fraction explained by MAP (Bio12) and seasonality of precipitation, quantified either as CHELSA’s Bio15 (“precipitation seasonality”), EarthEnv.org’s intra-annual SD of cloud cover, or DSL.

#### Ancient turnover and PBD_dev_

Although our aim is to assess turnover of lineages rather than turnover of taxa, taxonomic turnover [calculated using betapart’s (*88*) “beta.pair” function] and phylogenetic turnover are strongly correlated in Mimosoids (tables S12 and S13) and other taxonomic groups (*7*, *30*, *84*). To overcome this issue, we used two approaches.

First, we followed Daru *et al*. (*29*) and McFadden *et al*. (*97*) and calculated phylogenetic turnover at deeper levels in the phylogeny by collapsing all branches younger than a certain threshold, using the scripts provided by Daru *et al*. (*29*). We calculated ancient turnover at three thresholds: 5, 10, and 20 Ma ago.

Second, we used the PBD_dev_ metric of Peixoto *et al*. (*19*), which “measures the importance of phylogenetic beta diversity after factoring out taxonomic beta diversity.” PBD_dev_ is calculated as (taxonomic beta diversity − phylogenetic beta diversity)/taxonomic beta diversity (*19*). Here, we use the reverse of PBD_dev_ (i.e., −PBD_dev_), which is positively correlated with phylogenetic beta diversity.

Both ancient turnover and PBD_dev_ were used to perform variation partitioning to assess the influence of PNC and DL (see above) across all continents and using both phylogenies. For both measures, the correlation coefficients with taxonomic and phylogenetic beta diversity are reported (tables S12 to S15).

#### Biomes

The role of biomes in explaining phylogenetic turnover was examined by using variation partitioning to compare GDMs based on geographic and climatic distance and biome dissimilarity with GDMs based on just biome dissimilarity. For this test, grid cell pairs in the same biome were assigned a distance of zero, and grid cell pairs in different biomes a distance of one (*95*). Three biome schemes were used: the 14 biomes of Olson *et al*. (*98*), the 24 biomes of Higgins *et al*. (*99*), and a scheme recognizing three major tropical lowland biomes: tropical rain forest, savanna, and dry tropical forests/succulent biome (*20*, *100*, *101*). For the latter, three individual biome maps [i.e., tropical rain forest: Corlett and Primack (*102*); savanna: Lehmann *et al*. (*103*); and succulent biome: Ringelberg *et al*. (*20*)] were superimposed. The savanna and succulent biome maps overlap in several regions, and these overlapping regions were delimited as a fourth, intermediate biome (distance set to 0.5). Cells not covered by any biome map were excluded from the analyses. We assessed the effect of all biomes across all continents and using both phylogenies.

#### Phylogenetic regionalization

Phyloregionalization or bioregionalization analysis provides a way to visualize and map patterns of spatial phylogenetic turnover purely based on the phylogeny and occurrence dataset, independent from climatic or other types of data (*77*, *86*, *104*). We identified the best clustering algorithm for phyloregionalization by correlating cophenetic distance matrices with the corresponding phylogenetic distance matrices (*86*, *87*, *105*). However, as has been reported before (*87*, *105*), in some continents the best-scoring algorithm, unweighted pair group method with arithmetic mean (UPGMA), yielded highly unbalanced results, resulting in one very large phyloregion consisting of almost all grid cells, and multiple smaller phyloregions of just one or very few cells. We therefore selected the Ward clustering algorithm instead (*87*). Phyloregionalization was performed using the “hclust” function of the stats package (*51*), which partitioned grid cells into between two and eight clusters based on their phylogenetic beta diversity. Clustering was performed at pantropical and continent levels, using full phylogenetic turnover, ancient turnover, and geographic residuals.

To investigate whether the resulting clusters are climatically different from each other, we tested whether the MAP (Bio12), precipitation seasonality (Bio15), and DSL values of all cells making up a cluster are significantly different from the climatic values of other clusters. This was done using the Wilcoxon rank sum test [“wilcox.test” function of the stats package (*51*)] for comparisons of two clusters and the Kruskal-Wallis rank sum test (“kruskal.test” of the stats package) for comparisons of more than two clusters. In case of a significant outcome of the Kruskal-Wallis test, Dunn’s multiple comparison test with Bonferroni adjustment for multiple comparisons [using the “dunnTest” function of the FSA package (*106*), which relies on the dunn.test package (*107*)] was used to identify the number of climatically distinct clusters.

### Optimizations of climate and geography

#### Optimization of precipitation

Precipitation was optimized as a continuous variable across the MCC tree of the metachronogram using the “contMap” function of the phytools package (*67*). Two independent optimizations were performed, using the median values per species of MAP (Bio12) and DSL (the number of consecutive months with rainfall < 100 mm). To increase the accuracy of the optimization especially at deeper levels in the Mimosoid tree, data for the 78 non-Mimosoid Caesalpinioideae outgroup taxa in the phylogeny were included in the analyses. While the outgroup sampling is less dense than sampling within the Mimosoid clade, the 78 outgroup taxa provide a reasonable representation of the climatic and geographic distributions of the non-Mimosoid Caesalpinioideae and include taxa belonging to 59 of the 63 non-Mimosoid Caesalpinioideae genera.

Following the optimization, nodes and tips outside the Mimosoid clade were removed from the tree. Nodes were then divided into three rainfall categories following Neves *et al.* (*17*): dry (<1200 mm/year), wet (>1800 mm/year), and intermediate (between 1200 and 1800 mm/year). These categories were used to calculate the number, location, and age of niche shifts, defined as changes in rainfall regime (i.e., between dry, wet, and intermediate) that involve a change in MAP of at least 250 mm, optimized on a single branch. Niche shifts are estimated to have taken place at the midpoint of the ages of the parent and child nodes.

For the optimization of the DSL, nodes were also divided into three regimes: dry (>8 months), wet (<4 months), and intermediate (between 4 and 8 months). Niche shifts were defined as changes in rainfall regime that involve a change in DSL of at least 1 month.

Rainfall category thresholds correspond to the climatic space distinguishing lineages with wet and dry affinities. Wet lineages are hypothesized to not occur below 1200 MAP and dry lineages not above 1800 MAP (*17*). Dry tropical forest/succulent biome vegetation predominantly receives <1200 MAP (*20*, *33*, *101*) and is absent from regions with >1800 MAP (*101*), whereas 1800 (to 2000) MAP is the lower bound of tropical rain forest vegetation (*17*, *108*). Similarly, tropical rain forests experience fewer than 4 months with <100-mm rainfall per year (*108*), whereas more than half of all areas of succulent biome have a DSL of at least 8 months (*20*).

#### Phylogenetic signal

Phylogenetic signal, expressed as Pagel’s lambda (*34*), was calculated using the “phylosig” function in the phytools package (*67*), the MCC tree of the metachronogram, and median values of all Mimosoid species of MAP (Bio12) and DSL. Significance was assessed using a hypothesis test (option “test” of “phylosig”).

#### Tropical-temperate transitions

All Mimosoid and non-Mimosoid Caesalpinioid taxa in the metachronogram were scored as either tropical or temperate using definition 4 of the tropics of Feeley and Stroud (*40*), i.e., “all areas where temperatures do not go below freezing in a typical year.” This is a strict definition of the tropics, which excludes many subtropical areas in, e.g., mid-elevation areas of central Mexico that experience only mild frost (*40*), and therefore likely inflates the numbers of temperate taxa and tropical-temperate transitions found. Applying such a strict definition of the tropics, thereby estimating the maximum number of possible tropical to temperate transitions, provides a rigorous basis for comparison with the frequency of transcontinental dispersal. Temperature niche was optimized as a binary trait (i.e., tropical or temperate) across the full phylogeny, including non-Mimosoid Caesalpinioideae outgroups, using the “make.simmap” function in the phytools package (*67*), with 100 independent simulations and an all rates different (ARD) model. After removing the non-Mimosoid taxa from the output, transitions were identified as branches that connect two nodes with conflicting states in at least half of the simulations.

#### Optimization of geography

Geography was optimized across the MCC tree of the metachronogram, including non-Mimosoid Caesalpinioideae outgroups (see above), using BioGeoBEARS (*109*). To do so, all taxa in the metachronogram were assigned to one or more of eight areas using their distribution data: North America, South America, Africa, Madagascar, Asia, Australia, Oceania, and the European Mediterranean.

The BioGeoBEARS R package (*109*–*111*) was used to fit six different models: DEC, DEC+J, DIVALIKE, DIVALIKE+J, BAYAREALIKE, and BAYAREALIKE+J. After removing nodes representing non-Mimosoid Caesalpinioideae outgroup taxa from the BioGeoBEARS output of each model, the most probable ancestral range of each internal node was determined as the combination of all the most probable areas that together have a probability >50%. On the basis of this ancestral range reconstruction, the number, location, and age of transoceanic dispersal events were estimated using two different definitions of transoceanic dispersal events.

First, we consider all dispersal events between the Americas (i.e., North and South America treated as a single region), Africa, Madagascar, Asia, Australia, Oceania, and the Mediterranean as transoceanic. Second, we combine these seven areas into three, considering only dispersal events between the Americas, Africa-Madagascar-Asia-Australia-Mediterranean, and Oceania as a stricter definition of transoceanic dispersal. The results were summarized across all six models and both definitions of transoceanic dispersal using Akaike Information Criterion (AIC)-weighted model averaging.

### Lineage diversification dynamics through time

To explore scenarios of lineage turnover through time in relation to Cenozoic climate cooling that led to an increase in dry habitats across the planet (*45*), we ran a set of analyses with BAMM (Bayesian Analysis of Macroevolutionary Mixtures) (*112*), using the MCC tree of the metachronogram set with the outgroup removed except for *Erythrophleum* and *Pachyelasma* (the sister group of the Mimosoid clade) to include the stem lineage of the Mimosoid clade. This method has been criticized (*113*, *114*), but the author of the program has responded to these criticisms (*115*, *116*). More generally, any diversification rate estimation method suffers from a lack of power to estimate extinction rates (*117*), meaning that speciation rates are not identifiable from phylogenies (*118*). To account for this limitation, we ran analyses across a wide range of fixed extinction rates to assess how speciation rates would vary through time under various levels of turnover while making use of the powerful way in which BAMM can take unsampled diversity into account by assigning sampling fractions to genera or clades, which we estimated on the basis of our Mimosoid checklist and taxonomic expertise. Priors were set using the setBAMMpriors option of the BAMMtools R package (*119*), and extinction rates were fixed across different analyses at 0.05 lineages per million years and ranging from 0.5 to 3.5 with intervals of 0.5. Speciation rates and rate shifts (or, more accurately, shifts to different speciation rate regimes as the model also includes time-variable speciation rates) were left as free parameters to be estimated during the analysis. BAMM was then run for 10 million generations while saving parameters every 1000 generations. Phylorate plots and rate-through-time plots were drawn using the BAMMtools R package (*119*). The table with sampling fractions is available in data S1.

## Supplementary Materials

### This PDF file includes:

Supplementary Methods and Results Tables S1 to S24 Figs. S1 to S51 Data S1 References
