# Supplementary material for: Precipitation is the main axis of tropical plant phylogenetic turnover across space and time
Source: Sci Adv. 2023 Feb 17;9(7):eade4954. doi: 10.1126/sciadv.ade4954 (PMC10957106; doi:10.1126/sciadv.ade4954)
Supplement: Supplementary file 1 — Supplementary Methods and Results Tables S1 to S24 Figs. S1 to S51 Data S1 References [file sciadv.ade4954_sm.pdf]

Science Advances
AAAS

Supplementary Materials for

Precipitation is the main axis of tropical plant phylogenetic turnover across space and time

Jens J. Ringelberg *et al.*

Corresponding author: Jens J. Ringelberg, jens.ringelberg@gmail.com

Sci. Adv. **9**, eade4954 (2023)
DOI: 10.1126/sciadv.ade4954

The PDF file includes:

Supplementary Text
Tables S1 to S24
Figs. S1 to S51
References

Other Supplementary Material for this manuscript includes the following:

Data S1

1. Supplementary methods

1.1 Phylogenetic inference

1.1.1. Phylogenomic backbone

Data cleaning and target assembly

Figure S1 presents an overview of this step. Duplicated reads were removed with FastUniq 1.1 (*120*). Reads were filtered with Trimmomatic 0.36 (*121*) to remove adapter sequences, leading and trailing bases with a quality score < 20, sliding windows of five bp with an average quality score < 20, and reads shorter than 51 bp. Finally, we used HybPiper 1.3.1 (*122*) to generate sequences for the 997 target genes. HybPiper uses BWA (*123*) to map the cleaned paired and unpaired reads to their targets (i.e., the DNA sequences of the bait set used for the capture reaction), SPAdes (*124*) for *de novo* assembly of mapped reads into contigs, and Exonerate (*125*) to extract coding sequences of the target genes. We ran HybPiper using default settings, except that the coverage cut-off of SPAdes was lowered to two, which increased the number and length of recovered genes for several samples with degraded DNA derived from old herbarium specimens. HybPiper yields two types of output. The main output consists of one DNA sequence for each taxon/gene combination. In addition, in cases where multiple sequences are recovered for particular taxon/gene combinations, these extra, potentially paralogous sequences are also stored.

Trimming outlier sequences and orthology assessment

Figure S2 presents an overview of this step. To remove outlier sequences and assess orthology of potentially paralogous sequences of genes recovered by HybPiper we used slightly edited versions of steps five and six of the Yang and Smith (*126*) pipeline. We divided the sequences of the 997 genes into two groups: single-copy and multi-copy genes. The multi-copy genes are those genes for which HybPiper recovered potentially paralogous sequences in at least 5% of the taxa; genes for which fewer or no potential paralogs were found were presumed to be single-copy. We applied Yang and Smith's (*126*) pipeline in different ways to these two subgroups (Figure S2).

To trim outlier sequences from the single-copy genes, we first split the sequences for each gene into two roughly equally-sized groups of taxa: a subclade of the Ingoid clade (*24*, *26*) here referred to as the 'core ingoid' (CI) clade, i.e. all taxa descended from the most recent common ancestor of *Zapoteca* and *Zygia*, and the 'early-branching Caesalpinioideae' (EBC), i.e. all taxa not in the CI clade. Splitting the data in this way was done because, based on Koenen et al. (*24*) and preliminary analyses of our data, branch lengths in the CI clade tend to be considerably shorter than those outside this clade across the EBC. Removal of outlier sequences based on branch lengths therefore requires different settings and is more accurate if the CI and EBC taxa are analysed separately. For each group, codon alignments were produced with MACSE 2.01 (*127*), all sites with a column occupancy < 0.3 were trimmed using pxclsq (*128*), and gene trees inferred with RaxML 8.2.12 (*129*) using the GTRCAT model. Yang and Smith's (*126*) trim_tips.py script was used to trim outlier long tips with a relative cut-off of 0.1 and absolute cut-offs of 0.15 for the CI sequences and 0.3 for the EBC sequences. An additional round of trimming outlier taxa was performed using TreeShrink 1.3.3 (*130*) with a quantile of 0.1. Finally, long internal branches were cut using Yang and Smith's (*126*) cut_long_internal_branches.py script with a cut-off of 0.3 and only keeping clusters with at least 22 taxa. At this point, the CI and EBC sequences were reunited into a single dataset

for a second round of alignment with MACSE, tree-building with RaxML, removal of outlier long tips with trim_tips.py (with relative and absolute cut-offs of 0.1 and 0.3, respectively), and further removal of outliers using TreeShrink. Finally, outgroup sequences (if available) were added to the codon alignments with MACSE's enrichAlignment option, and codons with > 95% missing data were removed with BMGE 1.1 (*131*), resulting in final codon alignments with outgroups (if available) for all single-copy genes.

The multi-copy genes were analysed in two different ways, resulting in two different sets of final alignments for the same multi-copy genes (Figure S2). For the first set, potentially paralogous sequences were not included, and the multi-copy genes were analysed in exactly the same way as the single-copy genes (described above), resulting in codon alignments with outgroups (if available).

For the second set, potentially paralogous sequences were included, and a full orthology assessment following Yang and Smith (*126*) was performed (Figure S2). For this, the sequences of each gene were first split between the CI and the EBC. Then sequences for each of these two groups were aligned using MACSE, sites with < 0.3 column occupancy trimmed using pxclsq, gene trees inferred using RaxML, and outlier tips removed using Yang and Smith's (*126*) trim_tips.py (with absolute cut-offs of 0.15 and 0.3 for the CI and EBC, respectively) and TreeShrink (quantile 0.1). At this point, Yang and Smith's (*126*) mask_tips_by_taxonID_transcripts.py script was used to mask mono- and paraphyletic tips, after which internal branches > 0.3 were cut with cut_long_internal_branches.py, only keeping clusters with at least 22 taxa. This round of aligning, tree building, and trimming outliers was repeated for the CI and EBC separately, after which sequences of these two groups of taxa were reunited into a single dataset, for an additional round of aligning, tree building, and trimming outliers. Before trimming outliers in this last round, all sequences flagged as potential paralogs by HybPiper for eight putative recent polyploids were removed. These putative polyploids, *Vachellia erioloba*, *V. farnesiana*, *V. nilotica*, *Sympetalandra schmutzii*, *S. unijugum*, *Mimosa tricephala*, *Schleinitzia insularum*, and *Leucaena trichandra*, were identified based on total numbers of potential paralogs recovered by HybPiper and analysis of preliminary gene trees, as well as, in some cases, earlier reports of polyploidy (*132*, *133*). Potential paralogs were removed because presence of these homeologous polyploid sequences would negatively affect the next step of orthology assessment following the maximum inclusion method (*126*) with the prune_paralogs_MI.py script, using relative and absolute cut-offs of 0.1 and 0.3 respectively, and only keeping clusters with at least 22 taxa. The resulting ortholog clusters were subjected to one final round of aligning with MACSE, trimming codons with > 95% missing data with BMGE, tree inference with RaxML, and removing outliers with trim_tips.py (relative and absolute cut-off 0.1 and 0.3) and TreeShrink (quantile 0.1), resulting in trimmed, final ortholog alignments.

At this point three groups of gene alignments were available for phylogenetic analyses (second-bottom row in Figure S2): the single-copy gene alignments (with outgroups, if available), alignments of the multi-copy genes treated as single-copy (with outgroups, if available), and ortholog alignments resulting from full orthology assessment of the multi-copy genes (without outgroups).

Phylogenetic analyses

In addition to the multi-species coalescent approach to generating a species tree (described in the main text), we also used both maximum likelihood and Bayesian approaches to infer species trees based on gene alignments concatenated with pxcat (*128*). For the maximum likelihood analyses (Figure S4), we excluded alignments of loci of gene trees with a root-to-tip length variance higher than 0.009, and trimmed codons with > 90% missing data using BMGE. We produced six concatenated alignments: i.e. separate nucleotide and amino acid alignments of just the single-copy gene loci, of all gene loci including

paralogs, and of all gene loci excluding paralogs. Six species trees were inferred with RaxML, using 200 rapid bootstrap replicates with the GTRCAT model (*134*) for the nucleotide alignments and the PROTGAMMALG4X model (*135*) for the amino acid alignments, two complex substitution models that allow for rate heterogeneity across sites. Finally, we used a gene jackknifing approach (following Koenen et al. (*24*)) to infer a Bayesian species tree (Figure S5). After excluding loci of gene trees with a root-to-tip length variance > 0.009 and trimming codons with > 90% missing data, we divided the amino acid single-copy gene alignments over 16 roughly equally sized concatenated alignments of circa 19,700 sites each. This gene jackknifing procedure was repeated four times, resulting in 48 unique jackknifes. These jackknifes were then used as input to PhyloBayes-MPI (*136*) using the CATGTR model (*137*, *138*) and 1000 cycles, the first 500 of which were discarded as burn-in. Convergence of the jackknife-runs was checked with the R (*51*) package CODA (*139*). Finally, all trees resulting from the 48 unique jackknifes were summarized as a majority-rule consensus tree.

Chloroplast phylogeny

A chloroplast phylogeny was inferred following the approach of Koenen et al. (*24*). Off-target reads were extracted using BLAST and three Caesalpinioideae reference chloroplast genomes: *Inga leiocalycina* ((*140*); GenBank accession KT428296), *Leucaena trichandra* ((*140*); GenBank accession KT428297), and *Erythrophleum fordii* ((*141*); GenBank accession MG644609). Coding sequences for each plastid gene were extracted using a custom Python script (*24*), aligned with MACSE, and concatenated with pxcat. The concatenated alignment contains 72 plastid genes, because two genes (*accD* and *clpP*) were removed following Koenen et al. (*24*). Finally, all taxa with > 95% missing data were trimmed from the alignment, and a species tree was inferred with RaxML using the GTRCAT model and 200 rapid bootstrap replicates.

1.1.2 Metachronogram

Here we present a clade-by-clade description of the analyses to infer the subtrees. All analyses were carried out in MrBayes v. 3.2.1 (*142*) or BEAST v. 1.8.4 (*69*, *70*), using a GTR+GAMMA model, but with different clock models as specified below. For all analyses, convergence was assessed using Tracer v. 1.7 (*143*) and appropriate amounts of burn-in were discarded from the posterior samples. The subtree alignments and MrBayes execution files or BEAST xml files are available at https://github.com/erikkoenen/metachronogram, as well as example tree files that include 100 posterior trees for each subclade and the backbone.

Acacia

The *Acacia* subtrees were estimated from an alignment of 717 *Acacia* species and 7 outgroup taxa, using the nuclear internal and external transcribed spacers (*ITS* and *ETS*), and chloroplast markers *matK*, *psbA*, and *trnL,* with a total aligned length of 6,407 nucleotide positions. This data set builds on that of Mishler et al. (*144*), but includes sequences for 210 *Acacia* species newly generated here, representing the largest phylogeny of the genus to date. GenBank accession numbers for newly generated sequences are in Table S4; for the other sequences see Mishler et al. (*144*). The analysis was carried out in BEAST, using the GTR+GAMMA model, with separate partitions for the nuclear and chloroplast data and an uncorrelated relaxed clock (lognormal). Four separate chains were run for 100 million generations each, after which the first 50 million generations were discarded as burn-in and 1,000 post-burn-in trees

extracted. The outgroup taxa were pruned prior to grafting the trees onto the *Acacia* crown node in the backbone phylogeny.

Albizia clade

For the Albizia clade sensu Koenen et al. (*24*) we used 500 targeted exons for phylogenetic inference. These exons were selected by identifying all exons longer than than 500bp (including the flanking untranslated regions), and using the Yang & Smith (*126*) pipeline for orthology assessment, from an unpublished hybrid capture data set for the same Mimobaits gene set and probes used to construct the backbone tree. European Nucleotide Archive (ENA) accession numbers for these new data are included in Table S5. All accessions of the Albizia clade that were used in the backbone phylogeny are also included here. A total of 79 accessions of *Albizia,* six accessions of *Enterolobium*, a single accession of *Boliviadendron bolivianum* and seven outgroup accessions were sampled, for a total aligned length of 71,328 nucleotide positions. Subsequently, 32 informative and clock-like genes were selected using the same SortaDate procedure described above for the backbone phylogeny. The ultrametric trees were inferred with MrBayes using an independent gamma rates (IGR) clock model with a uniform branch length prior, running for 50 million generations. The outgroup was pruned prior to grafting the posterior trees onto the most recent common ancestor (MRCA) node of *Albizia* + *Enterolobium* + *Boliviadendron bolivianum* (i.e., the Albizia clade sensu Koenen et al. (*24*)) on the backbone phylogeny.

Calliandra

For *Calliandra* we used the *ITS* + *trnL-F* alignment of Souza et al. (*145*) which includes 90 species of *Calliandra* plus 40 outgroup accessions, with a total aligned length of 2,170 nucleotide positions. Analyses were carried out using the same settings in MrBayes as for the Albizia clade, but running only 30 million generations as this was sufficient to reach convergence due to the shorter alignment length. The outgroup was pruned in the posterior trees prior to grafting these on the crown node of *Calliandra* on the backbone phylogeny.

Dichrostachys group

For *Alantsilodendron, Dichrostachys and Gagnebina* (i.e. the informal Dichrostachys group of Hughes et al. (*146*), excluding *Calliandropsis* for which the accession in the backbone was retained), we used a subset of a newly generated nextRAD RADseq data set, including six accessions of *Alantsilodendron*, 17 of *Dichrostachys*, and five of *Gagnebina*, for a total aligned length of 34,030 nucleotide positions. Sample information and European Nucleotide Archive accession codes are in Table S6. The loci to be included were selected based on a minimum taxon occupancy of 75%. The analysis was carried out in MrBayes using the same settings as for the Albizia clade. An outgroup was not included but the subtrees were inferred with a constraint where the non-Madagascan species of *Dichrostachys* were enforced as the sister clade to the clade including the Madagascan species of *Dichrostachys* and the other two genera to make sure the trees were properly rooted. The subtrees were then grafted onto the MRCA node of the three genera on the backbone phylogeny.

Inga

We used a new Hybseq dataset that builds on the data presented in Nicholls et al. (*56*) for the genus *Inga,* with additional taxon sampling to include a total of 164 accessions of the genus (including morphospecies and/or cryptic taxa that have not yet been formally described), with a total aligned length of 134,648 nucleotide positions. Bait design, capture, and bioinformatics all follow Nicholls et al. (*56*). GenBank accession codes of all *Inga* samples are in Table S7. Accessions of *Zygia inundata* and *Z. sabatieri*, that are the sister taxa of *Inga*, from the backbone, were included in the subtree analysis to improve estimation of the branch length of the stem lineage and thus of the crown age of *Inga*. Analyses were carried out in BEAST using a fixed local clock model with a separate partition for a large clade

within *Inga* which has discreetly higher substitution rates than the remainder of the genus. Three chains of each 50 million generations were run and the postburn-in posterior samples of these were combined. After pruning the single outgroup accession (*Zygia* sp. voucher Coley & Kursar *Tip917*), the subtrees were grafted onto the MRCA node of the *Inga*, *Zygia inundata*, and *Z. sabatieri* clade in the backbone phylogeny.

Leucaena group

Subtrees for the clade composed of *Desmanthus, Kanaloa, Leucaena, Mezcala*, and *Schleinitzia* (i.e., the informal Leucaena group of Hughes et al. (*146*)), were inferred from an alignment of 72 chloroplast genes extracted from off-target reads from the hybrid capture accessions used to build the backbone phylogeny, plus a new set of *Leucaena* transcriptomes (Table S8) and the *Desmanthus illinoensis* transcriptome of Cannon et al. (*147*). This dataset includes a total of four *Desmanthus*, one *Kanaloa*, one *Mezcala*, 24 *Leucaena* and three *Schleinitzia* accessions, for a total aligned length of 53,788 nucleotide positions. The accessions of *Lemurodendron capuronensis* and *Neptunia oleracea* from the backbone phylogeny were used as the outgroup. The analysis was run in MrBayes with the same settings as for the Albizia clade, and after pruning the outgroup, the subtrees were grafted onto the MRCA of the five genera in the backbone phylogeny.

Mimosa

For the *Mimosa* subtrees we used an alignment of the chloroplast marker *trnD-trnT* that builds on the data sets of Simon et al. (*148*), Vasconcelos et al. (*149*), and Morales et al. (*150*), plus 49 newly generated sequences, giving a total of 428 accessions from the genus plus 13 outgroup accessions, with a total aligned length of 2,704 nucleotide positions. GenBank accession numbers of the newly sequenced accessions are included in Table S9. The analysis was run in BEAST using the same settings as for the *Acacia* subtree analysis and after pruning the outgroup the subtrees were grafted onto the crown node of the genus in the backbone phylogeny.

Madagascan *Mimosa*

A separate set of posterior subtrees was inferred for the Old World clade of *Mimosa*, which is mostly confined to Madagascar and nested within *Mimosa* (*148*), from a RADseq data set similar to that of *Alantsilodendron*, *Dichrostachys* and *Gagnebina*. Sample information and European Nucleotide Archive accession codes are in Table S10. Since the Madagascan *Mimosa* data set has more missing data across the RAD loci, we used a cut-off of at least 40% taxon occupancy per locus to have sufficient total aligned length after concatenation. The alignment includes 29 accessions, with a total aligned length of 41,729 nucleotide positions. No outgroup was included, but instead the analysis was run with a constrained topology in which the non-Madagascan species were enforced as the sister clade to the clade of Madagascan species (previously shown to be monophyletic by Simon et al. (*148*)) to ensure correct rooting of the subtrees. The analysis was run in MrBayes using the same settings as for the Albizia clade and the subtrees were grafted onto the MRCA node of the Old World clade of *Mimosa* in the *trnD-trnT Mimosa* subtrees described above.

Parkia clade

To generate an alignment for the Parkia clade sensu Koenen et al. (*24*), we used the *Anadenanthera*, *Parkia* and *Vachellia* sequence data of Boatwright et al. (*151*) and added additional sequences from GenBank (see Table S11), including those of Comben et al. (*152*) for Australian *Vachellia*. In addition, sequences for *Albizia kalkora, Chloroleucon mangense, Lysiloma tergeminum, Neptunia monosperma* and *Piptadenia stipulacea* from Boatwright et al. (*151*) and *Senegalia senegal* of Terra et al. (*153*) were included as outgroups. The alignment includes two species of *Anadenanthera,* 17 species of *Parkia* and 57 species of *Vachellia* and has a total aligned length of 4,444 nucleotide positions. The analysis was run

in MrBayes using a strict clock, because the IGR clock model caused the long stem branches of the three genera in the Parkia clade to be substantially shortened, and inferred unrealistically high substitution rates for these. The chain was run for 30 million generations. After pruning the outgroup the subtrees were grafted onto the crown node of the Parkia clade in the backbone phylogeny.

Piptadenia

We extracted the 13 accessions of *Piptadenia* from the alignment of Simon et al. (*154*), and *Mimosa myriadenia* to serve as the outgroup. These data include three chloroplast markers (*matK*/*trnK*, *trnD-trnT* and *trnL-trnF*) and nuclear *ITS*, with a total aligned length of 6,426 nucleotide positions when concatenated. The analysis was run with MrBayes using the same settings as for the Albizia clade, but running only 30 million generations. The outgroup was pruned and subtrees were grafted on the crown node of the genus in the backbone phylogeny.

Senegalia

We assembled an alignment of 70 *Senegalia* accessions plus *Parasenegalia vogeliana* as an outgroup by combining the data sets of Boatwright et al. (*151*) and Terra et al. (*153*) for the three chloroplast loci *trnK*/*matK*, *trnL-trnF* and *psbA-trnH*, with a total aligned length of 4,079. The analysis was run in MrBayes using the same settings as for the Albizia clade, but running only 30 million generations. Because *Senegalia* is non-monophyletic under its current circumscription (*24*, *26*, *155*), subtrees corresponding to the two separate clades of the genus were extracted and grafted separately onto their respective crown nodes in the backbone phylogeny.

Stryphnodendron clade

For the Stryphnodendron clade, we extracted sequence data for one accession of *Microlobius foetidus*, one accession of *Naiadendron*, four accessions each of *Parapiptadenia*, *Pityrocarpa*, and *Marlimorimia*, seven of *Gwilymia*, and 15 of *Stryphnodendron* from the alignment of Simon et al. (*154*). *Inga edulis, Mimosa myriadenia, Piptadenia robusta, Lachesiodendron viridiflora* and *Senegalia nigrescens* were used as the outgroup. These data comprise three chloroplast markers (*matK*/*trnK*, *trnD-trnT*, and *trnL-trnF*) and nuclear *ITS*, with a total aligned length of 6,426 nucleotide positions when concatenated. The analysis was run with MrBayes using the same settings as for the Albizia clade, but running only 30 million generations. The outgroup was pruned and subtrees were grafted on the MRCA node of *Parapiptadenia* and *Stryphnodendron* (i.e. the crown node of the Stryphnodendron clade sensu Koenen et al. (*24*)) in the backbone phylogeny. Note that the taxonomy of the Stryphnodendron clade has recently been revised (*58*, *156*).

Zapoteca clade

For the Zapoteca clade, sequence data for chloroplast locus *trnL-trnF* and nuclear *ETS* and *ITS* of Ferm (*157*) and Souza et al. (*158*) were combined to include *Faidherbia albida*, *Sanjappa cynometroides*, two species of *Thailentadopsis*, six species of *Viguieranthus* and 27 species and subspecies of *Zapoteca*, with a total aligned length of 2,759 nucleotide positions. Sequence data from Ferm (*157*) for *Calliandra dysantha, C. surinamensis, Havardia mexicana, H. pallens, Pithecellobium diversifolium* and *P. dulce* were included as the outgroup. The analysis was run in MrBayes using the same settings as for the Albizia clade, but running 30 million generations. After pruning the outgroup, the subtrees were grafted onto the crown node of the Zapoteca clade in the backbone phylogeny.

Zygia

For *Zygia*, we used data of Ferm et al. (*159*) to include 41 species and varieties of *Zygia* sensu stricto, and eight outgroup species (including *Z. inundata, Z. ocumarensis*, and *Z. sabatieri* that were shown by Ferm et al. (*159*) to be more closely related to *Inga* and *Macrosamanea*), with a total aligned length of

3,228 nucleotide positions. The analysis was run in MrBayes using the same settings as for the Albizia clade, but running only 30 million generations. After pruning the outgroup, the subtrees were grafted onto the crown node of *Zygia* in the backbone phylogeny.

2. Supplementary results

Only results not included in the main text or in main or supplementary figures or tables are presented here.

2.1 Phylogenetic inference

2.1.1 Phylogenomic backbone

Excluded taxa

Four accessions were excluded from the final phylogenetic analyses: *Calliandra umbrosa*, *Vouacapoua americana*, *Pterogyne nitens*, and one of the accessions of *Albizia subdimidiata* var. *subdimidiata*. The sequencing of *C. umbrosa* was unsuccessful, generating no reads that could be used in the analyses, probably because of low DNA quality and yield (circa 0.01 µg, the lowest yield of all samples) from poor quality herbarium material. While the sequencing of *V. americana* was more successful, only a few genes could be recovered for this taxon, and the vast majority of these were removed as outliers during data cleaning, leaving too few genes to be confident about the phylogenetic placement of this taxon. *Pterogyne nitens* and one of the accessions of *Albizia subdimidiata* var. *subdimidiata* were placed in the species trees, but their placements were highly unexpected, and inspection of gene trees and alignments heavily implies sequences of these two taxa were affected by contamination. They were therefore removed from all analyses. (Note that two accessions of *Albizia subdimidiata* var. *subdimidiata* were sampled, and that one of these, *Ferreira 210* (K), is still included in the phylogeny.)

Additionally, in every phylogeny *Hultholia mimosoides* was found to be nested within *Entada*, a placement that is clearly incorrect, as previous studies have placed *Hultholia* in the distantly-related Caesalpinia group (*57*, *78*). Although *H. mimosoides* should clearly not be placed in the Mimosoid clade, its highly divided bipinnate leaves are nevertheless Mimosoid-like (hence its specific epithet), and its geographic distribution overlaps with those of several *Entada* species (i.e., *E. glandulosa*, *E. phaseoloides*, *E. reticulata*, and *E. rheedii*). We therefore conclude that sample or identification mix-up has resulted in sequencing DNA from an unidentified *Entada* species, instead of *H. mimosoides*, and have renamed this sample '*Entada* sp. van Beusekom et al. 4706'.

With exclusion or renaming of these five taxa, samples of 420 ingroup taxa belonging to 158 of 163 Caesalpinioideae genera are included in the final phylogenies, alongside five outgroup taxa. This means that only five Caesalpinioideae genera were not sampled: *Stenodrepanum* and *Microlobius* (not sequenced); *Hultholia*, *Vouacapoua*, and *Pterogyne* (excluded from phylogenetic inference).

Sequencing results and data quality

Sequencing, data cleaning, and target assembly results per accession are presented in Table S1.

Fractions of duplicated reads, as determined by FastUniq (*120*), were moderately high, probably reflecting the large number of samples derived from old herbarium specimens with partially degraded DNA (Figure S6): TapeStation analyses showed that many DNA extractions yielded highly-fragmented DNA (average fragment sizes of circa 1000 bp) in low quantities (often < 1 µg) (results not shown).

Fractions of reads filtered by Trimmomatic (*121*), in contrast, were low (Figure S7). The target capture was successful, as shown by the high percentages of reads on target (Figure S8) and the high numbers of genes that were recovered with at least 75% of the target length (Figure S9) by HybPiper (*122*), thereby confirming that herbarium specimens can be rich sources of DNA for hybrid capture (*160*–*162*). The capture was less successful with lower percentage of reads on target in most samples from Koenen et al. (*24*) in part due to the considerably larger bait set used for the hybrid capture reaction in that study. For most samples, additional, potentially paralogous sequences were recovered for only a small number of genes, but in a small minority of samples substantially higher numbers of genes appear to be duplicated, suggesting that these taxa are likely polyploids (Figure S10).

Of the 997 target genes, 167 were treated as multi-copy (i.e., potentially paralogous sequences were present in > 5% of taxa), and the remaining 830 genes were deemed to be single-copy (Figure S2). Outgroup sequences were obtained for 918 genes (Table S2, Figure S2). Alignment lengths varied between 117 and 7,752 nucleotide sites, with a median length of 843 (Figure S11). Numbers of taxa (including outgroups) per alignment varied between 18 and 426 (Figure S12). The much lower number of taxa in the alignments of the multi-copy genes with paralogs (median number of taxa: 42) compared to alignments of the single-copy genes (median: 409) and multi-copy genes without paralogs (median: 412), is caused by the orthology inferences for the former, which cut up larger gene trees into smaller, orthologous clusters (*126*).

Phylogenetics

Only a handful of gene trees had relatively high root-to-tip variances (i.e., > 0.009; Figure S13), namely nine out of 830 single-copy gene trees, three out of 169 multi-copy gene trees without paralogs, and two out of 667 multi-copy gene trees with paralogs. These were excluded from further analyses. Concatenated alignments of the remaining loci were large, with many informative sites and relatively few gaps (Table S16, Figure S4).

A total of ten nuclear species trees were produced: three made with ASTRAL (Figure S3: Figures S14 – S16), six with RAxML (Figure S4: Figures S17 – S22), and one with PhyloBayes (Figure S5: Figure S23). All nuclear trees are robustly supported and largely congruent with each other (Figures S24 – S27), as evidenced by high topological similarities between trees (Table S17, Figure S28). The chloroplast phylogeny (Figure S29) has a considerable number of missing taxa (383 taxa, versus 427 in the nuclear phylogenies) and shows greater conflict with the nuclear trees than there is among the different nuclear estimates (Figure S28).

Gene tree conflict is ubiquitous across the Caesalpinioideae phylogeny: not a single bipartition in the single-copy ASTRAL phylogeny is supported by all 821 single-copy gene trees (Figures S30 and S31). Approximately one third of the nodes in the species tree are supported by at least half of all gene trees, and over half of species tree nodes are supported by at least a quarter of the gene trees. In contrast, for half of the species tree bipartitions there are more conflicting than supporting gene trees. In general there are many different conflicting topologies among the gene trees for each node, rather than a few abundant ones, indicating that gene tree conflict is mainly caused by lack of signal rather than common alternative topologies (*24*). Lack of signal in the gene trees is also directly evident given that for circa 20% of species tree nodes the majority of gene trees do not provide any information, be it in support or conflict. Some parts of the tree are especially prone to gene tree conflict, including along the ingoid backbone, the Madagascan clades in *Albizia* and *Dichrostachys* – *Alantsilodendron*, the Australasian

clade comprising *Acacia*, *Archidendron*, and several smaller genera, and across the recently diversified largely Amazonian rain forest genera *Macrosamanea*, *Zygia*, and *Inga*. Gene tree conflict is also indicated by the two types of internode certainty values, ICA and EQP-IC, which are strongly correlated with each other (Pearson's correlation coefficient 0.85), although on average the quartet-based EQP-IC scores are lower than the bipartition-based ICA values (Figure S32), as found by Zhou et al. (*64*).

ASTRAL's polytomy test shows the locations of several potential hard polytomies in the ASTRAL single-copy phylogeny (Figure S33), which closely coincide with nodes and clades associated with considerable gene tree conflict (Figures S30 – S32). These polytomies fall into two categories. The first category relates to the placements of difficult-to-place individual taxa, such as *Cylicodiscus gabunensis*, *Parasenegalia vogeliana*, and *Cedrelinga cateniformis*, while the second category comprises polytomies at the base of or within clades that most likely represent rapid radiations, such as along the ingoid backbone, in the Madagascan clades of *Albizia* and *Dichrostachys – Alantsilodendron*, within the Australasian clade, and within and among the recently diversified rain forest genera *Macrosamanea*, *Zygia*, and *Inga*. Several taxa in these clades are often identified by RogueNaRok as rogue taxa (Table S18), based on their variable placements in the RAxML bootstrap trees, indicating the difficulty, or perhaps impossibility, of resolving and representing the relationships resulting from rapid radiations as fully bifurcating topologies.

In the rest of this study, the single-copy ASTRAL phylogeny (Figure S14) is used as the 'reference' Caesalpinioideae phylogeny for several reasons. First, given the much larger size of the nuclear dataset based on large numbers of independent loci, and the high fraction of missing data and taxa in the chloroplast alignment, as well as the low bootstrap support values and short branches across parts of the plastid phylogeny, the nuclear trees provide a more reliable estimate of the species tree than the chloroplast phylogeny. Second, by selecting a phylogeny based solely on single-copy genes, the effects of the (absence of) orthology assessment, although minor, do not have to be considered. Furthermore, whether expressed in absolute gene numbers (Figure S30) or bipartition- or quartet-based internode certainty values (Figure S32), there is extensive conflict among individual gene trees, which violates the central assumption of the concatenation model (*163*). Finally, the multi-species coalescent model has been shown to consistently outperform the concatenation model on a range of phylogenomic datasets (*163*), which strongly suggests that the ASTRAL trees provide the most accurate approximation of the Caesalpinioideae species tree.

The eleven nuclear and plastid Caesalpinioideae phylogenies are available as online supplementary data for this article.

Note that the taxonomic consequences of the new Caesalpinioideae phylogeny are discussed elsewhere (*26*, *58*, *170–175*, *155*, *156*, *164–169*).

2.1.2 Time calibration

The time-calibrated Caesalpinioideae phylogeny is shown in Figure S31.

A time-calibrated tree file and the BEAST xml file, which includes the alignment of the 100 clock-like genes used for dating, are available as online supplementary data for this article.

2.1.3 Metachronogram

The Mimosoid clade in the maximum clade credibility (MCC) tree of the metachronogram is shown in Figure 1. Figure S34 depicts the same tree, with all added subtrees labelled and highlighted.

The 1000 metachronograms and the MCC tree with age intervals and posterior probabilities are available as online supplementary data for this article, as well as a table linking taxon names in the metachronogram with those in the occurrence dataset. Scripts for metachronogram analyses and the alignments of the subtrees are available on https://github.com/erikkoenen/metachronogram.

2.2 Taxonomic checklist and occurrence dataset

Mimosoid clade

Our taxonomic checklist for the Mimosoid clade (Appendix 2 (Data S1)) contains 3,464 accepted species and 671 infraspecific taxa (Table S19). 6,408 recent synonyms likely to have been used on herbarium specimen determinations are associated with these taxa.

Using the taxonomic checklist, we assembled 424,333 quality-controlled occurrence records representing all 100 Mimosoid genera, 3,232 species, and 514 infraspecific taxa, i.e. 93% of species and 91% of all taxa in the Mimosoid clade (Table S19), with a mean of 113 and a median of 33 records per taxon. Taxon richness quantified as the number of taxa per half degree longitude/latitude grid cell (Figure S35) shows that the Mimosoid clade is most diverse in the Neotropics and Australia. However, generic richness (Figure S35) shows that this is strongly affected by the hyperdiverse genus *Acacia*, as large parts of Australia are characterised by high numbers of *Acacia* taxa but very few other Mimosoid genera. Furthermore, under-representation of some African regions in online data repositories (*176*) also introduces sampling bias, revealed by the many African cells with low taxon richness but relatively high genus richness (Figure S35). In Asia, the low taxon and genus richness is attributable to the relatively low number of Asian Mimosoid lineages and the limited availability of data, especially from the Indian subcontinent.

Figure 1e shows Mimosoid growth form variation across the tropical precipitation gradient. From left to right, the following species are shown:

- *Strombocarpa tamarugo*, a small tree in the hyper arid Pampa del Tamarugal (northern Chile). Photographer: Oliver Whaley;
- *Vachellia tortilis*, a typical ‘umbrella thorn Acacia’ of African savannas, Serengeti National Park (Tanzania). Photographer: Robur.q (https://commons.wikimedia.org/wiki/File:Vachellia_(ex_Acacia)_tortilis.jpg, last visited on 31/05/2022);
- *Mimosa pumilio*, a geoxyle in the Brazilian Cerrado. Photographer: Marcelo F. Simon;
- *Entada rheedei*, a liana in humid tropical forest, Mozambique. Photographer: Bart T. Wursten (www.mozambiqueflora.com) (*177*);
- *Parkia pendula*, a giant tropical rainforest emergent tree in Brazil. Photographer: Marcelo F. Simon.
-

Non-Mimosoid Caesalpinioideae

The occurrence dataset of the 78 non-Mimosoid Caesalpinioideae outgroups contains 18,346 records, with a mean of 238 and a median of 109 records per taxon.

All occurrence data are available as online supplementary data for this article.

Abundance of Mimosoids across biomes

Mimosoids represent 5.4% of all tree species in the Amazon according to ter Steege et al. (*79*) and 4.5% following Cardoso et al. (*80*), 7.7% of all tree species in Neotropical dry forests (*81*), 17.3% of all African savanna tree species (*82*), and 5.4% of all native Australian Angiosperm species.

2.3 Phylogenetic turnover across space

Robustness tests

The GDMs, variation partitioning and phyloregionalization results obtained using the metachronogram and the genus-level tree used as a robustness test are quantitatively and qualitatively highly similar. The main metachronogram results are presented Figures 2 and 3 and Tables S12 and S20. Figures S36 – S41 show metachronogram phyloregionalization results per region with different numbers of clusters, as well as those based on the geographic residuals of phylogenetic turnover and ancient turnover. Figure S42 presents the metachronogram phyloregionalization results with a global distribution of isohyets. The climatic distinctiveness of the metachronogram phyloregions is shown in Figure S43.

Phyloregionalization and variation partitioning results obtained using the genus-level Mimosoid phylogeny are presented in Figures S44 and S45 and Tables S13 and S21.

For all phyloregionalization figures, the underlying map is the 'countriesHigh' world map of the rworldextra package (*178*).

Ancient turnover and PBD_{dev} are both more strongly correlated with phylogenetic beta diversity than with taxonomic beta diversity (Tables S12-S15). Variation partitioning results obtained using these metrics (Tables S12-S15) yield similar results to analyses using full phylogenetic turnover, indicating that our results do not just reflect the turnover of species but also that of lineages.

Biomes were found to explain very little to no additional phylogenetic turnover beyond climatic and geographic distance (Tables S22-S23).

2.4 Optimisations of climate and geography

Optimisation of precipitation

Estimated precipitation niche shifts through time are highly similar regardless of whether mean annual precipitation (Figure 1) or dry season length (Figure S46) are used to calculate ancestral niches. Figure 1b shows the fraction of precipitation niche shifts per speciation event per five million years averaged

across both optimisations, whereas the inset in Figure S46 shows the same just based on shifts in dry season length niches.

Optimisation of geography

The DEC+J model was recovered as the best-fitting model for the data. Fractions of dispersal events through time (Figure 1b, Table S24) were averaged across all twelve estimates (i.e., six models, two definitions of trans-oceanic dispersal), using AIC-weighted model averaging. Dispersal events mapped onto the metachronogram (Figure 1a) are those inferred by the DEC+J model with seven regions (i.e., the Americas, Africa, Madagascar, Asia, Australia, Oceania, and the Mediterranean). Ancestral states across Caesalpinioideae, based on the DEC+J model, and inferred trans-oceanic dispersal events in the Mimosoid clade, based on the model with seven regions, are shown in Figure S47.

2.5 Lineage diversification dynamics through time

The BAMM results are presented in Figures S48 and S49. Figure S48 shows the distribution of speciation rates across the metachronogram under different extinction scenarios. The top subfigure in Figure S49 shows the median speciation rate across the phylogeny in 100 time bins under the same extinction scenarios. Paleotemperatures in the middle figure are based on Zachos et al. (*179*), and were obtained using RPANDA (*180*). The phenogram was plotted using the 'phenogram' function in phytools (*67*).

An important limitation of the BAMM approach used here is that extinction rates are held constant through time. Under a model of episodic turnover, which we hypothesize (see main text), extinction rates are also likely to vary substantially through time, with relatively high levels of extinction likely during times of rapid historical climate change, and in particular, at the Eocene-Oligocene transition.

3. Note on supplementary data

Supplementary Data S1 on Zenodo (https://doi.org/10.5281/zenodo.7568716) contains the following files:

- Appendix 1: Information about the Mimosoid checklist and occurrence dataset;
- Appendix 2: Taxonomic checklist of the Mimosoid clade;
- a table with the sampling fractions used in the BAMM analysis;
- the xml file (including alignment of 100 clock-like genes) used for the BEAST dating analysis of the phylogenomic backbone, the resulting tree file with age ranges per node, and a table with up-to-date names for each taxon included in these analyses;
- the 1000 inferred metachronograms, and the MCC tree with age intervals and posterior probabilities;
- occurrence datasets of all Mimosoids and of the non-Mimosoid Caesalpinioideae present in the phylogenomic backbone;
- the eleven inferred phylogenomic backbones of Caesalpinioideae;
- and a table to match taxon names in the metachronogram and the occurrence datasets.

4. Supplementary tables

Table S1. Names and voucher details for species and accessions sampled in the Caesalpinioideae phylogenomic backbone, European Nucleotide Archive (ENA) accession codes, and the sequencing and target assembly results, including numbers of sequence reads on target, genes recovered, and paralog warnings for each accession.

Taxon	Voucher	ENA accession number	Source	Fraction of duplicated reads	Fraction of filtered reads	Reads left	Reads mapped	Genes with sequences	Genes recovered with 75% of target length	Potential paralogs
Abarema cochliacarpos	de Queiroz 15538 (HUEFS)	ERS4812838	Koenen et al. (*24*)	0.552	0.07	8499987	3150000 (37.1%)	971	853	15
Abarema diamantina	Guerra 148 (ICN)	ERS11697078	This study	0.194	0.005	6944231	5312876 (76.5%)	992	966	54
Acacia alata var. *biglandulosa*	Murphy 464 (MELU)	ERS11697096	This study	0.142	0.005	3811009	3043513 (79.9%)	992	961	35
Acacia ampliceps	Murphy 323 (MELU)	ERS11697097	This study	0.146	0.006	6059169	4736028 (78.2%)	994	970	39
Acacia auriculiformis	Brown 154 (MEL)	ERS11697098	This study	0.151	0.006	5079017	4191953 (82.5%)	994	964	38
Acacia ausfeldii	Karunajeewa 1149 (MEL)	ERS11697099	This study	0.13	0.007	5487545	4044020 (73.7%)	994	972	44
Acacia colei var. *colei*	Murphy 326 (MELU)	ERS11697100	This study	0.158	0.006	6192508	4808345 (77.6%)	993	939	46
Acacia deanei subsp. *paucijuga*	Murphy 599 (MELU)	ERS11697101	This study	0.148	0.006	7159214	5527391 (77.2%)	992	966	43
Acacia longifolia	Koenen 182 (Z)	ERS4812840	Koenen et al. (*24*)	0.012	0.018	3705539	115743 (3.1%)	772	565	0
Acacia lycopodiifolia	Murphy 339 (MELU)	ERS11697102	This study	0.149	0.006	2250348	1830789 (81.4%)	993	966	19
Acacia montana	Murphy 672 (MELU)	ERS11697103	This study	0.152	0.006	5357202	4096344 (76.5%)	992	966	43
Acacia murrayana	Ariati 110 (MELU)	ERS11697104	This study	0.148	0.005	5154241	3942099 (76.5%)	992	960	48
Acacia oswaldii	Murphy 573 (MELU)	ERS11697105	This study	0.153	0.005	5809958	4471048 (77%)	992	965	47
Acacia platycarpa	Murphy 327 (MELU)	ERS11697106	This study	0.152	0.005	4125748	3097650 (75.1%)	992	969	38
Acacia pycnantha	Murphy 670 (MELU)	ERS11697107	This study	0.17	0.006	7377943	5368248 (72.8%)	991	963	46
Acacia pyrifolia	Murphy 337 (MELU)	ERS11697108	This study	0.244	0.007	2763767	2293777 (83%)	993	953	22
Acacia rostellifera	Murphy 466 (MELU)	ERS11697109	This study	0.19	0.005	8302200	6384932 (76.9%)	993	976	42
Acacia sibirica	Murphy 486 (MEL)	ERS11697110	This study	0.159	0.005	6046537	4625699 (76.5%)	988	954	55
Acacia triptera	Karunajeewa 1446 (MEL)	ERS11697111	This study	0.165	0.006	7506934	5657563 (75.4%)	992	969	49

Acacia tumida	Murphy 306 (MELU)	ERS11697112	This study	0.194	0.007	2415907	2004246 (83%)	991	949	26
Acacia vernici-flua	Karunajeewa 1012 (MEL)	ERS11697113	This study	0.137	0.006	3949459	3006398 (76.1%)	992	972	33
Acacia victo-riae	Ariati 260 (MELU)	ERS11697114	This study	0.155	0.006	6876195	5437933 (79.1%)	994	969	45
Acaciella vil-losa	Hughes 2635 (FHO)	ERS4812841	Koenen et al. (*24*)	0.037	0.02	2911372	74570 (2.6%)	598	314	0
Acrocarpus fraxinifolius	Manos 1416 (DUKE)	ERS11697115	This study	0.236	0.008	5998945	3865675 (64.4%)	955	746	89
Adenanthera pavonina	Ambriansyah and Arifin AA295 (K)	ERS4812842	Koenen et al. (*24*)	0.016	0.018	4242045	164600 (3.9%)	882	588	12
Adenopodia patens	Sandoval MS343 (K)	ERS4812843	Koenen et al. (*24*)	0.043	0.022	6434933	443279 (6.9%)	968	778	8
Adenopodia scelerata	Jongkind 10602 (WAG)	ERS4812844	Koenen et al. (*24*)	0.047	0.008	9208280	707958 (7.7%)	981	874	16
Afrocalliandra gilbertii	Gillett and Hemming 24799 (PRE)	ERS11697116	This study	0.202	0.006	4355148	3628668 (83.3%)	990	941	29
Afrocalliandra redacta	Germishuizen 5585 (PRE)	ERS11697117	This study	0.248	0.006	7960105	6666175 (83.7%)	992	947	27
Alantsiloden-dron glomera-tum	Koenen 257 (G, K)	ERS11697118	This study	0.139	0.006	4313074	3157671 (73.2%)	992	946	47
Alantsiloden-dron pilosum	Koenen 203 (Z)	ERS4812845	Koenen et al. (*24*)	0.023	0.015	3141103	202237 (6.4%)	928	721	2
Alantsiloden-dron villosum	Koenen 409 (G, K)	ERS11697119	This study	0.156	0.005	3698603	2946722 (79.7%)	992	945	30
Albizia adian-thifolia	Wieringa 6278 (WAG)	ERS4812846	Koenen et al. (*24*)	0.029	0.008	9142035	461499 (5%)	973	834	10
Albizia anthel-mintica	Maurin 363 (JRAU)	ERS4812848	Koenen et al. (*24*)	0.028	0.01	5165679	327512 (6.3%)	970	831	8
Albizia ataka-taka	Koenen 229 (Z)	ERS4812849	Koenen et al. (*24*)	0.647	0.089	12500723	4350341 (34.8%)	972	807	14
Albizia auris-parsa	Koenen 230 (Z)	ERS4812850	Koenen et al. (*24*)	0.031	0.01	14432059	1021898 (7.1%)	986	898	18
Albizia bernieri	Koenen 354 (Z)	ERS4812851	Koenen et al. (*24*)	0.016	0.016	3429090	142276 (4.1%)	879	687	8
Albizia boivinii	Koenen 270 (Z)	ERS4812852	Koenen et al. (*24*)	0.028	0.013	3362669	234729 (7%)	955	784	14
Albizia brevi-folia	Maurin 826 (JRAU)	ERS4812853	Koenen et al. (*24*)	0.023	0.02	2433285	156865 (6.4%)	906	662	5
Albizia ferrug-inea	Jongkind 10762 (WAG)	ERS4812857	Koenen et al. (*24*)	0.033	0.008	6199048	343428 (5.5%)	965	839	10
Albizia grandi-bracteata	Koenen 159 (WAG)	ERS4812858	Koenen et al. (*24*)	0.72	0.095	7971575	2894494 (36.3%)	969	791	12
Albizia maha-lao	Koenen 216 (Z)	ERS4812860	Koenen et al. (*24*)	0.711	0.096	15008953	6126074 (40.8%)	972	802	12
Albizia masiko-rorum	Koenen 237 (Z)	ERS4812861	Koenen et al. (*24*)	0.032	0.011	11494741	723554 (6.3%)	988	895	15

Albizia obbiadensis	Thulin 4163 (UPS)	ERS4812862	Koenen et al. (*24*)	0.031	0.012	4969768	331864 (6.7%)	966	838	10
Albizia polyphylla	Koenen 256 (Z)	ERS4812864	Koenen et al. (*24*)	0.029	0.008	2976472	209635 (7%)	945	769	15
Albizia retusa	Hyland 2732 (L)	ERS4812865	Koenen et al. (*24*)	0.032	0.011	10800530	702560 (6.5%)	981	864	10
Albizia sahafariensis	Koenen 405 (Z)	ERS4812866	Koenen et al. (*24*)	0.031	0.018	11553872	734373 (6.4%)	983	885	16
Albizia saponaria	Jobson 1041 (BH)	ERS4812867	Koenen et al. (*24*)	0.7	0.095	8862638	3243698 (36.6%)	972	803	15
Albizia umbellata	Jobson 1037 (BH)	ERS4812882	Koenen et al. (*24*)	0.536	0.067	9829794	4228923 (43%)	971	854	20
Albizia umbellata subsp. *umbellata*	Larsen 33736 (K)	ERS11697169	This study	0.243	0.01	2937052	2603852 (88.7%)	982	789	93
Albizia versicolor	Maurin 560 (JRAU)	ERS4812868	Koenen et al. (*24*)	0.719	0.086	13969835	5950552 (42.6%)	969	806	9
Albizia viridis	Du Puy M251 (K)	ERS4812869	Koenen et al. (*24*)	0.029	0.013	6447985	426750 (6.6%)	973	836	15
Albizia zygia	Wieringa 5915 (WAG)	ERS4812870	Koenen et al. (*24*)	0.03	0.008	7296600	381783 (5.2%)	969	820	9
Amblygonocarpus andongensis	Sokpon 1451 (WAG)	ERS4812871	Koenen et al. (*24*)	0.02	0.016	4673226	157163 (3.4%)	864	527	12
Anadenanthera colubrina	de Queiroz 15685 (HUEFS)	ERS4812872	Koenen et al. (*24*)	0.024	0.012	3882704	231474 (6%)	943	755	7
Anonychium africanum	Essou 2110 (WAG)	ERS4812931	Koenen et al. (*24*)	0.033	0.009	10475738	879557 (8.4%)	985	854	15
Arapatiella psilophylla	de Lima et al. 7906 (RB)	ERS11697134	This study	0.168	0.006	4701923	3236767 (68.8%)	985	861	70
Archidendron bubalinum	Latiff and Zainudin ALM3503 (L)	ERS11697135	This study	0.226	0.005	4606643	3935161 (85.4%)	994	964	22
Archidendron clypearia	Wieringa 1849 (WAG)	ERS11697136	This study	0.197	0.006	8533396	7182846 (84.2%)	994	968	32
Archidendron ellipticum subsp. *ellipticum*	Kalat ARK 42 (L)	ERS11697137	This study	0.191	0.006	6306349	5430400 (86.1%)	994	967	29
Archidendron grandiflorum	Clarkson 6233 (L)	ERS11697138	This study	0.178	0.008	7556417	5729504 (75.8%)	995	968	43
Archidendron jiringa	Annable 3321 (NY)	ERS11697139	This study	0.195	0.006	4588076	3900989 (85%)	995	970	25
Archidendron kanisii	Ford and Holmes AF3669 (L)	ERS11697140	This study	0.185	0.006	5970058	4833236 (81%)	994	970	40
Archidendron lucidum	Wang and Lin 2534 (L)	ERS4812873	Koenen et al. (*24*)	0.014	0.01	5795037	316866 (5.5%)	971	824	9
Archidendron ptenopum	Takeuchi and Ama 15334 (L)	ERS11697141	This study	0.149	0.006	4613690	3511372 (76.1%)	993	968	46
Archidendron quocense	Newman 2094 (E)	ERS4812874	Koenen et al. (*24*)	0.726	0.092	11417868	4456246 (39%)	970	806	9
Archidendron triplinervium	Church et al. 1171 (L)	ERS11697142	This study	0.218	0.006	5563658	4815056 (86.5%)	994	973	18

Archidendropsis granulosa	McKee 38353 (L)	ERS4812875	Koenen et al. (*24*)	0.021	0.009	12082020	831488 (6.9%)	987	910	12
Arcoa gonavensis	Gardner and Knees 7026 (E)	ERS11697144	This study	0.182	0.007	9685695	6011856 (62.1%)	939	662	212
Arquita trichocarpa	Gagnon 2018 (MT)	ERS11697145	This study	0.247	0.007	3176819	1627215 (51.2%)	953	677	35
Aubrevillea kerstingii	Nimba Botanic Team JR957 (WAG)	ERS4812876	Koenen et al. (*24*)	0.015	0.016	5685788	231500 (4.1%)	926	708	10
Balsamocarpon brevifolium	Eggli and Leuenberger 2986 (Z)	ERS11697148	This study	0.254	0.009	4616413	1847991 (40%)	955	732	54
Batesia floribunda	Grenand 3032 (CAY)	ERS11697149	This study	0.233	0.006	492546	291743 (59.2%)	934	649	27
Biancaea decapetala	Hughes 2227 (FHO)	ERS11697150	This study	0.276	0.007	6890313	4093095 (59.4%)	964	767	42
Blanchetiodendron blanchetii	de Queiroz 15616 (HUEFS)	ERS4812879	Koenen et al. (*24*)	0.029	0.008	6107868	314085 (5.1%)	970	828	10
Boliviadendron bolivianum	Hughes 2608 (FHO)	ERS4812907	Koenen et al. (*24*)	0.023	0.009	7312893	334766 (4.6%)	972	841	13
Burkea africana	Smith 6 (WAG)	ERS11697151	This study	0.233	0.006	2546859	1774273 (69.7%)	983	853	47
Bussea perrieri	Randrianasolo 527 (WAG)	ERS11697152	This study	0.173	0.005	3607830	2443747 (67.7%)	983	834	52
Caesalpinia cassioides	Pennington 789 (K)	ERS11697153	This study	0.248	0.006	5725554	3462449 (60.5%)	971	749	41
Calliandra bella	de Queiroz 15696 (HUEFS)	ERS11697156	This study	0.214	0.007	8940737	7202271 (80.6%)	992	944	37
Calliandra haematocephala	Hughes 2604 (FHO)	ERS11697157	This study	0.199	0.007	5482960	4308847 (78.6%)	986	945	38
Calliandra haematomma	Kass 2008 1 (BH)	ERS11697158	This study	0.212	0.006	6493219	5542035 (85.4%)	990	940	32
Calliandra hygrophila	de Queiroz 15542 (HUEFS)	ERS4812880	Koenen et al. (*24*)	0.039	0.014	3725309	211230 (5.7%)	919	717	7
Calliandra parviflora	Wood 26606 (K)	ERS11697159	This study	0.18	0.007	7464597	6167415 (82.6%)	984	943	38
Calliandra sessilis	de Queiroz 15608 (HUEFS)	ERS11697160	This study	0.204	0.006	5678535	4695359 (82.7%)	988	931	39
Calliandra sp. nov.	Poilane 9150 (P)	ERS11697161	This study	0.232	0.006	2706841	2297453 (84.9%)	991	943	29
Calliandra viscidula	de Queiroz 15541 (HUEFS)	ERS11697162	This study	0.209	0.006	6337439	5203387 (82.1%)	988	950	27
Calliandropsis nervosa	Hughes 1784 (K)	ERS11697163	This study	0.214	0.006	6190848	4841250 (78.2%)	992	959	39
Calpocalyx dinklagei	Wieringa 6094 (WAG)	ERS4812881	Koenen et al. (*24*)	0.023	0.013	10150267	312067 (3.1%)	936	692	10
Calpocalyx heitzii	van Valkenburg 2877 (WAG)	ERS11697164	This study	0.162	0.006	2555176	1877054 (73.5%)	989	889	33
Campsiandra comosa	Iganci 856 (RB)	ERS11697165	This study	0.18	0.008	8560245	6097949 (71.2%)	986	908	94
Cassia cowanii var. *guianensis*	Redden 3277 (US)	ERS11697166	This study	0.182	0.007	7359511	4902385 (66.6%)	973	795	46

Cedrelinga cateniformis	Pennington 17761 (K)	ERS4812883	Koenen et al. (*24*)	0.02	0.019	3588744	223591 (6.2%)	950	768	6
Cenostigma pluviosum var. *maraniona*	Hughes 3105 (MT)	ERS11697170	This study	0.257	0.007	8289046	4853867 (58.6%)	970	782	62
Ceratonia siliqua	Wieringa 3477 (WAG)	ERS11697171	This study	0.224	0.006	4232224	2964154 (70%)	966	722	63
Chamaecrista adiantifolia	Iganci 861 (RB)	ERS11697172	This study	0.146	0.007	2899104	1813687 (62.6%)	969	777	55
Chamaecrista lineata	Bradley 32006 (US)	ERS11697173	This study	0.19	0.006	4065468	2504843 (61.6%)	947	671	27
Chamaecrista ramosa	Lewis 3845 (K)	ERS11697174	This study	0.198	0.005	4926849	3116425 (63.3%)	945	696	36
Chamaecrista viscosa	Wood 26658 (K)	ERS11697175	This study	0.195	0.006	4228490	2512358 (59.4%)	945	702	37
Chidlowia san-guinea	Wieringa 4338 (WAG)	ERS4812884	Koenen et al. (*24*)	0.02	0.015	8052913	247743 (3.1%)	923	664	11
Chloroleucon mangense var. *mangense*	Lozano 1166 (K)	ERS11697176	This study	0.134	0.005	3472053	2708124 (78%)	994	971	24
Chloroleucon tenuiflorum	de Queiroz 15514 (HUEFS)	ERS4812885	Koenen et al. (*24*)	0.018	0.01	6789783	419135 (6.2%)	973	868	15
Cojoba arborea	Simon 1545 (CEN)	ERS4812886	Koenen et al. (*24*)	0.047	0.008	9087281	556186 (6.1%)	982	871	15
Cojoba filipes	Colella 1331 (NY)	ERS11697177	This study	0.278	0.006	5763912	4870940 (84.5%)	994	960	26
Cojoba rufescens	Castroviejo 14683 (K)	ERS11697178	This study	0.206	0.005	4589082	3566079 (77.7%)	994	967	42
Cojoba zanonii	Zanoni 36337 (NY)	ERS11697179	This study	0.186	0.007	3584825	3121762 (87.1%)	993	962	21
Colvillea race-mosa	Bruneau 1403 (MT)	ERS11697180	This study	0.19	0.005	5746997	3834447 (66.7%)	980	884	65
Conzattia mul-tiflora	Hughes 2071 (FHO)	ERS11697181	This study	0.186	0.005	4260881	2903786 (68.1%)	977	869	48
Cordeauxia edulis	Kuchar 17803 (K)	ERS11697182	This study	0.164	0.007	2922067	1681248 (57.5%)	967	761	44
Coulteria platy-loba	MacQueen 178 (FHO)	ERS11697154	This study	0.26	0.008	7199957	4181262 (58.1%)	965	738	152
Cylicodiscus gabunensis	Sosef 645A (WAG)	ERS4812887	Koenen et al. (*24*)	0.037	0.009	6104369	404128 (6.6%)	964	770	15
Delonix decaryi	Koenen 238 (G, K)	ERS11697183	This study	0.178	0.006	4250257	2742084 (64.5%)	977	872	67
Delonix edule	Willing s.n. (K)	ERS11697227	This study	0.19	0.006	4981753	3367148 (67.6%)	979	864	50
Denisophytum madagascar-iense	Bruneau et al. 1348 (MT)	ERS11697184	This study	0.217	0.01	2930272	1392506 (47.5%)	959	715	33
Desmanthus acuminatus	Hughes 2314 (FHO)	ERS11697185	This study	0.14	0.005	3176892	2421869 (76.2%)	995	963	35
Desmanthus leptophyllus	Hughes 2035 (FHO)	ERS4812888	Koenen et al. (*24*)	0.019	0.018	4309291	193040 (4.5%)	917	686	7

Desmanthus virgatus	Wood 26551 (K)	ERS11697187	This study	0.148	0.005	5846308	4447399 (76.1%)	992	965	51
Dichrostachys cinerea	Maurin 256 (JRAU)	ERS4812889	Koenen et al. (*24*)	0.03	0.018	4143707	247851 (6%)	942	720	3
Dichrostachys myriophylla	Koenen 301 (G, K)	ERS11697188	This study	0.145	0.005	3010514	2241701 (74.5%)	995	959	37
Dichrostachys paucifoliolata	Luckow 4157 (BH)	ERS11697189	This study	0.157	0.006	7700124	5511552 (71.6%)	993	958	51
Dichrostachys richardiana	Koenen 282 (G, K)	ERS11697190	This study	0.127	0.006	3899707	3113125 (79.8%)	992	955	32
Dichrostachys tenuifolia	Labat 3579 (P)	ERS11697191	This study	0.141	0.006	3335610	2699005 (80.9%)	993	943	25
Dichrostachys unijuga	Koenen 242 (G, K)	ERS11697192	This study	0.145	0.006	3262061	2495849 (76.5%)	993	940	36
Dimorphandra davisii	Luckow 4593 (BH)	ERS11697193	This study	0.174	0.005	3658031	2579975 (70.5%)	986	870	64
Dimorphandra gardneriana	Hughes 2409 (FHO)	ERS11697194	This study	0.228	0.006	4727541	3206179 (67.8%)	987	881	75
Dimorphandra macrostachya	Iganci 877 (RB)	ERS4812890	Koenen et al. (*24*)	0.014	0.017	6153872	144945 (2.4%)	823	586	19
Dinizia jueira-na-facao	Folli 4889 (HUE-FS, K)	ERS11697195	This study	0.167	0.009	3254995	2291449 (70.4%)	986	887	66
Diptychandra aurantiaca	Wood 26513 (K)	ERS4812891	Koenen et al. (*24*)	0.011	0.015	7891660	67216 (0.9%)	463	262	2
Ebenopsis confinis	Hughes 1539 (FHO)	ERS4812892	Koenen et al. (*24*)	0.042	0.019	4878310	253534 (5.2%)	953	736	9
Entada afri-cana	van der Maesen 7144 (WAG)	ERS11697197	This study	0.185	0.006	6429832	4515988 (70.2%)	988	925	38
Entada are-naria	Bamps 8098 (WAG)	ERS11697198	This study	0.192	0.007	4085220	3196694 (78.3%)	986	893	27
Entada burkei	van der Bank 15 (JRAU)	ERS11697196	This study	0.236	0.006	4778607	3486450 (73%)	994	928	41
Entada el-ephantina	Komape, Mabe, and Siebert 198 (JRAU)	ERS4812893	Koenen et al. (*24*)	0.02	0.008	6908210	277014 (4%)	926	734	14
Entada pervillei	Koenen 302 (G, K)	ERS11697199	This study	0.216	0.006	5885323	4655217 (79.1%)	992	927	36
Entada polys-tachya	Amaral Santos 3326 (CEN)	ERS11697200	This study	0.181	0.005	5920321	4327808 (73.1%)	986	908	32
Entada rheedei	Koenen 496 (Z)	ERS4812894	Koenen et al. (*24*)	0.016	0.008	2451784	103654 (4.2%)	808	615	5
Entada sp.	van Beusekom et al. 4706	ERS11697222	This study	0.406	0.037	209536	90355 (43.1%)	794	472	7
Entada tuber-osa	Koenen 417 (G, K)	ERS11697201	This study	0.183	0.005	3895325	2740535 (70.4%)	987	917	40
Enterolobium barinense	Blanco 157 (NY)	ERS11697202	This study	0.297	0.01	5647074	4652940 (82.4%)	992	970	31
Enterolobium barnebianum	Villa 1775 (K)	ERS11697203	This study	0.223	0.006	8653164	7178035 (83%)	993	956	48
Enterolobium contortisiliq-uum	de Queiroz 15579 (HUEFS)	ERS4812895	Koenen et al. (*24*)	0.013	0.017	8146204	642461 (7.9%)	928	702	11

Enterolobium cyclocarpum	MacQueen and Styles 75 (K)	ERS11697204	This study	0.225	0.007	9560067	8015575 (83.8%)	994	975	34
Enterolobium gummiferum	Harley 28284 (K)	ERS11697205	This study	0.247	0.006	9400120	7936398 (84.4%)	994	974	28
Enterolobium maximum	Nascimento 34 (K)	ERS11697206	This study	0.234	0.006	7827154	6486086 (82.9%)	994	973	36
Erythrophleum ivorense	Wieringa 5487 (WAG)	ERS4812896	Koenen et al. (*24*)	0.023	0.007	10744241	337474 (3.1%)	942	711	24
Erythrophleum teysmannii	Smitinand 10468 (K)	ERS11697209	This study	0.175	0.007	2684620	1818272 (67.7%)	987	890	50
Erythrostemon coluteifolius	Gagnon 207 (MT)	ERS11697210	This study	0.268	0.007	2617653	1142755 (43.7%)	949	689	33
Erythrostemon mexicanus	Gagnon 2010 015 (MT)	ERS11697211	This study	0.251	0.009	3472543	1723736 (49.6%)	956	672	22
Faidherbia albida	Maurin 3495 (JRAU)	ERS4812897	Koenen et al. (*24*)	0.044	0.011	5705125	315581 (5.5%)	963	793	9
Falcataria moluccana	Ambri and Arifin W826A (K)	ERS4812898	Koenen et al. (*24*)	0.032	0.015	6552373	420103 (6.4%)	976	829	10
Fillaeopsis discophora	Wieringa 5498 (WAG)	ERS4812899	Koenen et al. (*24*)	0.014	0.025	2028878	61595 (3%)	487	279	0
Gagnebina commersoni-ana	Koenen 374 (G, K)	ERS11697212	This study	0.153	0.005	4924185	3728358 (75.7%)	993	959	37
Gelrebia ros-trata	6th International Legume Confer-ence 5 (JRAU)	ERS11697213	This study	0.266	0.01	3977542	1979853 (49.8%)	942	638	24
Gleditsia chin-ensis	Koenen 604 (Z)	ERS11697214	This study	0.103	0.009	1852772	1015332 (54.8%)	956	723	74
Gretheria cam-pylacantha	Hughes 1404 (K)	ERS11697218	This study	0.186	0.006	5858307	5056883 (86.3%)	996	945	28
Guilandina bonduc	Herendeen and Mbago 9 XII 97 3 (US)	ERS11697215	This study	0.256	0.008	3088619	1411360 (45.7%)	962	763	34
Gwilymia paniculata	Simon 1058 (CEN)	ERS11697313	This study	0.154	0.006	5781510	4469062 (77.3%)	993	972	42
Gymnocladus dioicus	Jongkind and Wieringa 4426 (WAG)	ERS11697216	This study	0.185	0.005	4530437	2849766 (62.9%)	963	782	96
Haematoxylum brasiletto	Gagnon 2010 013 (MT)	ERS11697217	This study	0.343	0.008	7115301	4248340 (59.7%)	959	710	40
Havardia pal-lens	Hughes 2138 (FHO)	ERS4812900	Koenen et al. (*24*)	0.042	0.012	5849724	298968 (5.1%)	967	809	13
Heliodendron xanthoxylon	Hyland 9229 (L)	ERS11697143	This study	0.141	0.005	4667843	3839349 (82.3%)	993	966	30
Hererolandia pearsonii	Kolberg and Loots HK 1399 (K)	ERS11697219	This study	0.258	0.006	9555894	5524893 (57.8%)	957	791	55
Hesperalbizzia occidentalis	Hughes 1296 (FHO)	ERS4812901	Koenen et al. (*24*)	0.022	0.006	5078817	321941 (6.3%)	964	824	7
Heteroflorum sclerocarpum	Hughes 1849 (FHO)	ERS11697220	This study	0.228	0.007	4860825	3374699 (69.4%)	978	834	42

Hoffmannseg-gia arequip-ensis	Hughes 2342 (FHO)	ERS11697221	This study	0.241	0.008	5218642	3131359 (60%)	942	640	16
Hydrochorea corymbosa (1)	Bonadeu 655 (RB)	ERS4812902	Koenen et al. (*24*)	0.705	0.091	8772810	2969459 (33.8%)	970	799	12
Hydrochorea corymbosa (2)	Iganci 862 (RB)	ERS4812903	Koenen et al. (*24*)	0.537	0.071	7443935	3123121 (42%)	971	850	18
Hydrochorea elegans	Iganci 870 (RB)	ERS11697146	This study	0.215	0.006	7937229	5995772 (75.5%)	995	955	46
Hydrochorea gonggrijpii	Tillett 45696 (K)	ERS11697223	This study	0.22	0.006	5304614	4552594 (85.8%)	994	960	26
Hydrochorea leucocalyx	Aguilar 1939 (NY)	ERS11697147	This study	0.22	0.006	5789748	4546951 (78.5%)	994	970	47
Hydrochorea obliquifoliolata	Wieringa 6519 (WAG)	ERS4812863	Koenen et al. (*24*)	0.559	0.063	4785416	2047233 (42.8%)	972	840	14
Hydrochorea panurensis	Morim 563 (RB)	ERS11697224	This study	0.222	0.006	8308280	6365865 (76.6%)	994	965	50
Hydrochorea pedicellaris	de Queiroz 15529 (HUEFS)	ERS4812877	Koenen et al. (*24*)	0.503	0.07	11239481	3679071 (32.7%)	974	859	20
Hydrochorea rhombifolia	Deighton 3618 (K)	ERS11697168	This study	0.216	0.007	3323568	2721152 (81.9%)	995	969	33
Hydrochorea uaupensis	Morim 577 (RB)	ERS4812878	Koenen et al. (*24*)	0.541	0.064	7968724	3021322 (37.9%)	971	849	19
Indopiptadenia oudhensis	Adhikari, Pouls-en, and Parmar BAG31 (E)	ERS11697225	This study	0.165	0.006	4741919	3591620 (75.7%)	943	678	295
Inga alata	Coley and Kursar TAKPDC1673	SAMEA3283847	Nicholls et al. (*56*)	0.07	0.008	1310260	586851 (44.8%)	962	827	23
Inga alba	Coley and Kursar TAKPDC1677 (UT)	ERR776844	Koenen et al. (*24*)	0.062	0.009	1496500	613302 (41%)	969	822	27
Inga auristellae	Coley and Kursar TAKPDC1681 (UT)	SAMEA3283845	Nicholls et al. (*56*)	0.06	0.01	1369861	593606 (43.3%)	966	821	31
Inga bourgonii	Coley and Kursar TAKPDC1688 (UT)	SAMEA3283822	Nicholls et al. (*56*)	0.055	0.01	1214463	543496 (44.8%)	970	823	27
Inga brevipes	Coley and Kursar TAKPDC1694 (UT)	SAMEA3283838	Nicholls et al. (*56*)	0.011	0.011	1275553	527411 (41.3%)	966	832	32
Inga cin-namomea	Dexter 465 (E, MOL)	SAMEA3283820	Nicholls et al. (*56*)	0.069	0.009	1462414	626418 (42.8%)	968	822	33
Inga cylindrica	Coley and Kursar TAKPDC1713 (UT)	SAMEA3283801	Nicholls et al. (*56*)	0.067	0.009	1398145	617850 (44.2%)	966	815	25
Inga edulis	Coley and Kursar TAKPDC1719 (UT)	ERR776838	Koenen et al. (*24*)	0.058	0.011	1427222	617452 (43.3%)	965	819	25
Inga hetero-phylla	Dexter 345 (E, MOL)	SAMEA3283844	Nicholls et al. (*56*)	0.075	0.011	1217628	536299 (44%)	967	813	25
Inga huberi	Coley and Kursar TAKPDC1755 (UT)	ERR776810	Koenen et al. (*24*)	0.058	0.011	1350368	593530 (44%)	968	812	24

Inga laurina	Dexter 398 (E)	ERR776816	Koenen et al. (*24*)	0.078	0.009	1381539	571714 (41.4%)	970	813	28
Inga leiocalycina	Dexter 355 (E, MOL)	SAMEA3283810	Nicholls et al. (*56*)	0.073	0.008	1325708	566598 (42.7%)	966	817	27
Inga longiflora	Coley and Kursar TAKPDC1788 (UT)	SAMEA3283836	Nicholls et al. (*56*)	0.066	0.009	1253600	543896 (43.4%)	967	812	27
Inga marginata	BCI 8582	SAMEA3283827	Nicholls et al. (*56*)	0.017	0.008	968533	394059 (40.7%)	964	844	30
Inga nouragensis	Coley and Kursar TAKPDC1819 (UT)	SAMEA3283830	Nicholls et al. (*56*)	0.062	0.011	1273976	555161 (43.6%)	969	831	26
Inga pezizifera	BCI 8577	SAMEA3283806	Nicholls et al. (*56*)	0.017	0.007	790785	325289 (41.1%)	961	832	25
Inga punctata	BCI 8580	SAMEA3283828	Nicholls et al. (*56*)	0.016	0.009	1008652	387572 (38.4%)	969	838	28
Inga ruiziana	BCI 8589	SAMEA3283824	Nicholls et al. (*56*)	0.008	0.008	749402	243746 (32.5%)	961	826	24
Inga sapindoides	BCI 97	SAMEA3283803	Nicholls et al. (*56*)	0.072	0.008	1369321	514025 (37.5%)	965	808	29
Inga setosa	Dexter 343 (E, MOL)	SAMEA3283850	Nicholls et al. (*56*)	0.055	0.011	1109681	479709 (43.2%)	969	825	18
Inga stipularis	Coley and Kursar TAKPDC1856 (UT)	ERR776821	Koenen et al. (*24*)	0.067	0.008	1497618	629753 (42.1%)	965	834	28
Inga tenuistipula	Dexter 110 (E)	ERR776831	Koenen et al. (*24*)	0.071	0.008	1246734	543681 (43.6%)	967	822	29
Inga thibaudiana	Coley and Kursar TAKPDC1859 (UT)	SAMEA3283785	Nicholls et al. (*56*)	0.064	0.01	1302945	552562 (42.4%)	966	816	31
Inga umbellifera	BCI 103	SAMEA3283799	Nicholls et al. (*56*)	0.011	0.01	1355642	546555 (40.3%)	964	841	32
Jacqueshuberia brevipes	Redden 1240 (US)	ERS11697226	This study	0.231	0.005	5380639	3913315 (72.7%)	980	861	58
Jupunba abbottii	Zanoni 21220 (NY)	ERS11697071	This study	0.196	0.008	3747723	3197746 (85.3%)	995	968	31
Jupunba aspleniifolia	Ekman 6383 (NY)	ERS11697072	This study	0.442	0.009	959289	796982 (83.1%)	994	954	24
Jupunba barbouriana	Iganci 847 (RB)	ERS11697073	This study	0.233	0.006	7284640	5844453 (80.2%)	993	962	38
Jupunba brachystachya	de Lima 7438 (RB)	ERS11697074	This study	0.19	0.006	5455545	4232261 (77.6%)	994	973	46
Jupunba cochleata	Bonadeu 673 (RB)	ERS11697076	This study	0.18	0.006	5895755	4445955 (75.4%)	994	969	46
Jupunba commutata	Maguire 46145 (NY)	ERS11697077	This study	0.237	0.006	5221538	4211275 (80.7%)	994	968	41
Jupunba filamentosa	de Lima 7487 (RB)	ERS11697079	This study	0.188	0.006	5318746	4121885 (77.5%)	993	965	50
Jupunba floribunda	Iganci 883 (RB)	ERS11697080	This study	0.156	0.005	5803035	4578418 (78.9%)	994	966	61
Jupunba idiopoda	Quesada 1718 (NY)	ERS11697081	This study	0.195	0.005	3232056	2713116 (83.9%)	993	961	28

Jupunba laeta	Mori 25147 (NY)	ERS11697083	This study	0.268	0.007	1665213	1237759 (74.3%)	994	965	24
Jupunba langsdorffii	Ribeiro 728 (RB)	ERS11697084	This study	0.178	0.006	5605517	4246609 (75.8%)	992	971	45
Jupunba leucophylla	Iganci 839 (RB)	ERS11697086	This study	0.24	0.006	10089905	7755339 (76.9%)	993	959	62
Jupunba longipedunculata	Cardona 2682 (NY)	ERS11697088	This study	0.295	0.006	3933788	3191531 (81.1%)	994	961	30
Jupunba macradenia	Lourteig 3021 (NY)	ERS11697089	This study	0.337	0.007	3391013	2796383 (82.5%)	994	966	32
Jupunba microcalyx	Iganci 855 (RB)	ERS11697090	This study	0.187	0.006	7478422	5711833 (76.4%)	994	962	51
Jupunba nipensis	Mayo 19662 (NY)	ERS11697091	This study	0.424	0.008	810725	605585 (74.7%)	993	958	21
Jupunba oppositifolia	Liegier 16014 (NY)	ERS11697092	This study	0.302	0.007	3447350	2868985 (83.2%)	994	965	35
Jupunba oxyphyllidia	Yonker 6157 (NY)	ERS11697093	This study	0.236	0.006	4429470	3679176 (83.1%)	993	967	30
Jupunba rhombea	Iganci 261 (RB)	ERS11697087	This study	0.188	0.006	5403648	4148748 (76.8%)	993	970	47
Jupunba trapezifolia var. *micradenia*	Simon 1600 (CEN)	ERS4812839	Koenen et al. (*24*)	0.54	0.066	6111172	2117308 (34.6%)	968	832	18
Jupunba villosa	Borges 423 (RB)	ERS11697095	This study	0.194	0.007	6341126	4848824 (76.5%)	995	968	43
Kanaloa kahoolawensis	Lorence 7380 (PTBG)	ERS4812904	Koenen et al. (*24*)	0.036	0.008	11308250	1138422 (10.1%)	983	926	20
Lachesiodendron viridiflorum	de Queiroz 15614 (HUEFS)	ERS4812905	Koenen et al. (*24*)	0.04	0.009	16885363	1108078 (6.6%)	985	933	26
Lemurodendron capuronii	Koenen 435 (Z)	ERS4812906	Koenen et al. (*24*)	0.029	0.013	6336745	487016 (7.7%)	955	631	103
Leucaena trichandra	Hughes 1128 (FHO)	ERS11697228	This study	0.171	0.005	7893619	5938340 (75.2%)	949	816	462
Leucochloron limae	Chase 8250 (K)	ERS4812908	Koenen et al. (*24*)	0.019	0.01	7205109	443002 (6.1%)	976	848	13
Libidibia glabrata	Lewis and Lozano 3073 (K)	ERS11697229	This study	0.244	0.008	4178019	2495534 (59.7%)	958	696	21
Lophocarpinia aculeatifolia	Vogt 1321 (G)	ERS11697230	This study	0.246	0.008	7044959	3807972 (54.1%)	951	721	43
Lysiloma candidum	Marazzi 300 (ASU)	ERS4812909	Koenen et al. (*24*)	0.005	0.019	1872260	35152 (1.9%)	154	93	0
Lysiloma latisiliquum	Pennington 9197 (K)	ERS11697231	This study	0.104	0.008	4349268	3444588 (79.2%)	995	972	35
Macrosamanea amplissima	Bonadeu 663 (RB)	ERS4812910	Koenen et al. (*24*)	0.017	0.007	2228110	101748 (4.6%)	758	584	3
Macrosamanea discolor	Wurdack 42732 (K)	ERS11697232	This study	0.15	0.008	2575768	2112820 (82%)	992	943	32
Macrosamanea duckei	Simon 1646 (CEN)	ERS11697233	This study	0.179	0.005	3977417	3260309 (82%)	990	948	34

Macrosamanea kegelii	Prevost 1721 (NY)	ERS11697234	This study	0.218	0.006	2335405	1871666 (80.1%)	990	954	25
Macrosamanea prancei	da Silva 183 (NY)	ERS11697235	This study	0.229	0.007	3118916	2600083 (83.4%)	989	934	24
Macrosamanea simabifolia	Iganci 881 (RB)	ERS11697236	This study	0.181	0.006	6849903	4840869 (70.7%)	994	960	64
Macrosamanea spruceana	Steyermark 87793 (NY)	ERS11697237	This study	0.435	0.01	2262225	1781741 (78.8%)	991	940	31
Mariosousa sericea	Chase 18949 (K)	ERS4812911	Koenen et al. (*24*)	0.037	0.007	7488756	682808 (9.1%)	982	853	14
Marlimorimia contorta	de Queiroz 15582 (HUEFS)	ERS4812933	Koenen et al. (*24*)	0.048	0.008	6924713	330101 (4.8%)	962	821	22
Marlimorimia psilostachya	Simon 1245 (CEN)	ERS11697284	This study	0.147	0.006	2749436	1962195 (71.4%)	993	963	38
Melanoxylon brauna	Lopes and Andrade 113 (K)	ERS11697238	This study	0.188	0.006	4898735	3379585 (69%)	978	801	56
Mezcala balsensis	Hughes 1825 (FHO)	ERS11697186	This study	0.136	0.006	5883758	4369334 (74.3%)	993	965	42
Mezoneuron kauaiensis	Lorence and Wagner 8904 (PTBG)	ERS11697239	This study	0.27	0.008	5356803	3183152 (59.4%)	962	749	37
Mimosa ceratonia	de Queiroz 15484 (HUEFS)	ERS11697240	This study	0.14	0.005	2841672	2277796 (80.2%)	988	923	28
Mimosa dolens	Simon 879 (FHO)	ERS11697241	This study	0.143	0.006	4467084	3367839 (75.4%)	956	683	148
Mimosa grandidieri	Koenen 207 (Z)	ERS4812912	Koenen et al. (*24*)	0.051	0.008	7052923	400295 (5.7%)	963	792	12
Mimosa hexandra	Wood 26499 (K)	ERS11697242	This study	0.138	0.007	3213510	2288855 (71.2%)	989	911	42
Mimosa hondurana	Simon 858 (MEXU)	ERS11697243	This study	0.139	0.007	3583743	2590167 (72.3%)	983	929	36
Mimosa invisa	Simon 715 (FHO)	ERS11697244	This study	0.144	0.006	4871784	3636864 (74.7%)	985	919	25
Mimosa myriadenia	Iganci 835 (RB)	ERS11697245	This study	0.148	0.006	4099090	3053023 (74.5%)	991	958	27
Mimosa pigra	Simon 820 (MEXU)	ERS11697246	This study	0.159	0.007	7042106	4955742 (70.4%)	985	840	49
Mimosa pudica	Simon 1480 (CEN)	ERS11697247	This study	0.182	0.005	7218521	5309829 (73.6%)	971	813	30
Mimosa revoluta	Hughes 3051 (Z)	ERS11697248	This study	0.142	0.005	3058656	2380707 (77.8%)	991	957	29
Mimosa rubicaulis subsp. *himalayana*	Thomas SM 24 1 (K)	ERS11697249	This study	0.165	0.006	5237948	3929708 (75%)	988	932	28
Mimosa speciosissima	Simon 753 (FHO)	ERS11697250	This study	0.148	0.006	3608125	2665822 (73.9%)	978	889	23
Mimosa tenuiflora	de Queiroz 15498 (HUEFS)	ERS4812913	Koenen et al. (*24*)	0.051	0.008	5629617	250289 (4.4%)	927	733	10
Mimosa tequilana	Simon 813 (MEXU)	ERS11697251	This study	0.137	0.005	5364099	3924410 (73.2%)	979	882	33

Mimosa tricephala	Simon 849 (MEXU)	ERS11697252	This study	0.139	0.006	5195320	3729774 (71.8%)	946	719	223
Mimozyganthus carinatus	Hughes 2476 (FHO)	ERS4812914	Koenen et al. (*24*)	0.037	0.01	7315427	404905 (5.5%)	962	805	14
Moldenhawera floribunda	Klitgaard 30 (K)	ERS11697253	This study	0.171	0.007	2169956	1202944 (55.4%)	979	818	40
Mora gonggrijpii	Breteler 13792 (WAG)	ERS11697254	This study	0.205	0.008	7112635	4980946 (70%)	984	874	85
Moullava spicata	Gillis 9504 (MO)	ERS11697255	This study	0.412	0.018	198073	95544 (48.2%)	813	324	6
Naiadendron duckeanum	Simon 1606 (CEN)	ERS11697312	This study	0.146	0.006	3948999	3097143 (78.4%)	991	966	38
Neltuma argentina	Guaglianone 1338 (NY)	ERS11697276	This study	0.174	0.007	4752670	4103193 (86.3%)	995	958	21
Neltuma juliflora	Hughes 1703 (FHO)	ERS11697280	This study	0.164	0.009	4009818	3161949 (78.9%)	992	957	21
Neltuma kuntzei	Hughes 2458 (FHO)	ERS11697281	This study	0.165	0.006	8818267	6735181 (76.4%)	992	970	48
Neltuma laevigata	Hughes 2058 (FHO)	ERS4812932	Koenen et al. (*24*)	0.02	0.019	3056948	113584 (3.7%)	772	590	7
Neltuma ruscifolia	Fortunato 6773 (NY)	ERS11697282	This study	0.175	0.015	930099	732360 (78.7%)	992	906	15
Neptunia oleracea	Koenen 283 (Z)	ERS4812915	Koenen et al. (*24*)	0.029	0.032	8812926	529920 (6%)	977	795	13
Newtonia hildebrandtii	Maurin 2457 (JRAU)	ERS4812916	Koenen et al. (*24*)	0.039	0.008	7884530	415593 (5.3%)	966	761	19
Osodendron altissimum	Jongkind 10709 (WAG)	ERS4812847	Koenen et al. (*24*)	0.526	0.066	9157157	3852321 (42.1%)	973	856	19
Osodendron dinklagei	Jongkind 7359 (WAG)	ERS4812855	Koenen et al. (*24*)	0.01	0.017	1903228	75207 (4%)	581	441	3
Osodendron leptophyllum (1)	Brummitt 14035 (K)	ERS11697292	This study	0.168	0.006	5071059	4377041 (86.3%)	994	968	28
Osodendron leptophyllum (2)	Breteler 507 (WAG)	ERS11697167	This study	0.217	0.006	7065837	5992229 (84.8%)	995	967	34
Pachyelasma tessmannii	Wieringa 5229 (WAG)	ERS4812917	Koenen et al. (*24*)	0.021	0.009	10879659	415320 (3.8%)	959	757	25
Painteria elachistophylla	Guzman Cruz UG2169 (K)	ERS11697256	This study	0.2	0.006	4999242	3952300 (79.1%)	993	957	36
Parapiptadenia excelsa	Hughes 2451 (FHO)	ERS11697258	This study	0.135	0.005	2284574	1771891 (77.6%)	993	953	32
Parapiptadenia zehntneri	de Queiroz 15692 (HUEFS)	ERS4812918	Koenen et al. (*24*)	0.032	0.017	3906807	170055 (4.4%)	898	701	7
Pararchidendron pruinosum	Jobson 1039 (BH)	ERS4812919	Koenen et al. (*24*)	0.018	0.007	7208030	382671 (5.3%)	969	840	11
Parasenegalia visco	Fortunato 7645 (SI)	ERS11697302	This study	0.14	0.006	7345791	5884473 (80.1%)	993	971	38
Parasenegalia vogeliana	Zanoni 34986 (NY)	ERS11697126	This study	0.365	0.008	1201177	943730 (78.6%)	993	896	30

Paraserianthes lophantha subsp. *lophantha*	van Slageren and Newton MSRN648 (K)	ERS4812920	Koenen et al. (*24*)	0.017	0.008	5986133	302423 (5.1%)	964	827	9
Parkia bahiae	de Queiroz 15699 (HUEFS)	ERS11697259	This study	0.153	0.005	7977371	6329950 (79.3%)	993	970	48
Parkia bicolor	Wieringa 6265 (WAG)	ERS11697260	This study	0.132	0.006	4451222	3569411 (80.2%)	992	969	39
Parkia igneiflora	Iganci 885 (RB)	ERS11697261	This study	0.148	0.005	4751363	3705148 (78%)	991	966	47
Parkia panurensis	Iganci 842 (RB)	ERS4812921	Koenen et al. (*24*)	0.024	0.019	2295962	125886 (5.5%)	837	582	4
Parkia pendula	Simon 1194 (CEN)	ERS11697262	This study	0.179	0.006	5659537	4390458 (77.6%)	993	964	44
Parkia timoriana	Murphy 351 (NY)	ERS11697263	This study	0.144	0.007	4739305	3697930 (78%)	989	967	41
Parkia ulei	Poncy s.n. (K)	ERS11697264	This study	0.156	0.006	6025015	4186376 (69.5%)	994	974	59
Parkinsonia andicola	Hughes 2619 (FHO)	ERS11697265	This study	0.176	0.006	4517120	2911215 (64.4%)	984	885	70
Paubrasilia echinata	Filgueiras 3391 (NY)	ERS11697266	This study	0.242	0.007	3580881	1992192 (55.6%)	973	768	32
Peltophorum africanum	Koenen 601 (Z)	ERS4812922	Koenen et al. (*24*)	0.009	0.026	2589158	45554 (1.8%)	368	142	2
Peltophorum dubium	Hughes 2436 (FHO)	ERS11697267	This study	0.182	0.006	7352587	5207658 (70.8%)	984	861	57
Pentaclethra macroloba	Boyle 6720 (K)	ERS11697268	This study	0.153	0.006	3213908	2491507 (77.5%)	989	903	29
Pentaclethra macrophylla	Galeuchet & Balthazar 10 (Z)	ERS4812923	Koenen et al. (*24*)	0.024	0.008	16663175	524022 (3.1%)	968	780	16
Piptadenia buchtienii	Hughes 2427 (FHO)	ERS11697269	This study	0.135	0.006	4154987	3328448 (80.1%)	993	967	38
Piptadenia robusta	Luckow 4633 (BH)	ERS4812924	Koenen et al. (*24*)	0.038	0.011	3153147	216876 (6.9%)	926	687	22
Piptadenia uaupensis	Mori & Ishikawa 20836 (K)	ERS11697270	This study	0.133	0.006	4852695	4048317 (83.4%)	990	966	39
Piptadeniastrum africanum	Koenen 152 (WAG)	ERS4812925	Koenen et al. (*24*)	0.018	0.008	8360522	385173 (4.6%)	953	757	15
Piptadeniopsis lomentifera	Luckow 4505 (BH)	ERS4812926	Koenen et al. (*24*)	0.026	0.007	5891872	427637 (7.3%)	973	812	9
Pithecellobium dulce	Marazzi 309 (ASU)	ERS4812927	Koenen et al. (*24*)	0.036	0.007	6013147	372328 (6.2%)	974	835	14
Pithecellobium excelsum	Hughes 3101 (Z)	ERS11697271	This study	0.187	0.006	4722927	3840361 (81.3%)	994	969	36
Pithecellobium hymenaeifolium	Arvigo 216 (NY)	ERS11697272	This study	0.216	0.005	5649519	4686247 (82.9%)	993	968	33
Pithecellobium keyense	Chase 8958 (K)	ERS11697273	This study	0.217	0.005	6251735	4912041 (78.6%)	994	968	39
Pithecellobium macrandrium	Aguilar 1894 (NY)	ERS11697274	This study	0.214	0.006	4881101	3956361 (81.1%)	993	962	40

Pityrocarpa moniliformis	Wood 26516 (K)	ERS4812928	Koenen et al. (*24*)	0.041	0.014	5395524	221123 (4.1%)	929	753	16
Pityrocarpa schumanniana	de Lima 7903 (RB)	ERS11697285	This study	0.134	0.006	2846698	2021242 (71%)	994	960	45
Plathymenia reticulata	de Queiroz 15688 (HUEFS)	ERS4812929	Koenen et al. (*24*)	0.009	0.019	2196912	116395 (5.3%)	822	563	5
Pomaria jamesii	Gagnon 2010 020 (MT)	ERS11697275	This study	0.228	0.007	2454719	1057749 (43.1%)	944	622	21
Prosopidas-trum globosum	Luckow s.n. (BH)	ERS4812930	Koenen et al. (*24*)	0.033	0.018	5321583	337387 (6.3%)	962	775	7
Prosopis ciner-aria	Hafisullah and Dilawar 266 (Z)	ERS11697277	This study	0.314	0.04	101697	65927 (64.8%)	697	467	4
Prosopis farcta	Kerimov 31 (NY)	ERS11697278	This study	0.243	0.006	3041167	2258136 (74.3%)	992	955	33
Pseudalbizzia adinocephala (1)	Hughes 1435 (K)	ERS11697133	This study	0.211	0.006	3486392	2827768 (81.1%)	995	977	24
Pseudalbizzia adinocephala (2)	Hughes 1070 (FHO)	ERS11697120	This study	0.344	0.007	4675412	3431491 (73.4%)	995	980	42
Pseudalbizzia berteroana	Jiménez Rodríguez 1107 (NY)	ERS11697121	This study	0.251	0.006	4737523	3786287 (79.9%)	993	977	29
Pseudalbizzia burkartiana	Stival-Santos 678 (RB)	ERS4812854	Koenen et al. (*24*)	0.031	0.014	5440692	242245 (4.5%)	956	792	7
Pseudalbizzia coripatensis	Hughes 2433 (FHO)	ERS11697123	This study	0.258	0.006	7841663	6094428 (77.7%)	993	975	41
Pseudalbizzia decandra	Vilhena 231 (NY)	ERS11697124	This study	0.377	0.01	830632	644911 (77.6%)	994	957	12
Pseudalbizzia edwallii	Dalmaso 272 (RB)	ERS4812856	Koenen et al. (*24*)	0.02	0.008	3116764	180288 (5.8%)	921	764	8
Pseudalbizzia glabripetala	Lewis 1652 (K)	ERS11697125	This study	0.562	0.011	1176536	881434 (74.9%)	990	905	32
Pseudalbizzia inundata	Wood 26530 (K)	ERS4812859	Koenen et al. (*24*)	0.725	0.096	7376881	2521660 (34.2%)	968	785	7
Pseudalbizzia multiflora	Hughes 3090 (Z)	ERS11697127	This study	0.225	0.006	4764898	3772512 (79.2%)	994	968	28
Pseudalbizzia niopoides	Simon 1601 (CEN)	ERS11697128	This study	0.218	0.006	5693507	4724671 (83%)	995	972	22
Pseudalbizzia sinaloensis	Hughes 1576 (K)	ERS11697130	This study	0.222	0.007	3073383	2471008 (80.4%)	993	976	23
Pseudalbizzia subdimidiata var. *minor*	Gorts 341 (K)	ERS11697131	This study	0.527	0.012	649626	455201 (70.1%)	993	966	19
Pseudalbizzia subdimidiata var. *subdimid-iata*	Ferreira 210 (K)	ERS11697129	This study	0.259	0.005	4907457	4054305 (82.6%)	993	966	24
Pseudalbizzia tomentosa	Hughes 648 (K)	ERS11697132	This study	0.237	0.006	3848755	3084913 (80.2%)	994	972	23
Pseudoprosop-is euryphylla	Lock & Frison 88/83 (K)	ERS11697286	This study	0.146	0.007	2291993	1765731 (77%)	987	858	28

Pseudoprosopis gilletii	Wieringa 6021 (WAG)	ERS4812934	Koenen et al. (*24*)	0.018	0.007	5604090	159884 (2.9%)	857	573	11
Pseudoprosopis sericea	Jongkind 9513 (WAG)	ERS11697287	This study	0.153	0.006	3850946	2800400 (72.7%)	988	904	48
Pseudosamanea carbonaria	Daza 16353 (K)	ERS11697122	This study	0.233	0.006	10728752	8913272 (83.1%)	994	977	35
Pseudosamanea cubana	Leon 12095 (NY)	ERS11697288	This study	0.161	0.007	3885869	3042116 (78.3%)	995	977	33
Pseudosamanea guachapele	Hughes 1198 (FHO)	ERS4812935	Koenen et al. (*24*)	0.021	0.014	6673993	473494 (7.1%)	982	858	12
Pseudosenegalia feddeana	Atahuachi 1146 (FHO, LPB)	ERS11697289	This study	0.171	0.005	6105338	5021078 (82.2%)	991	944	69
Pterolobium stellatum	Herendeen and Mbago 17 XII 97 9 (F)	ERS11697290	This study	0.242	0.007	3887117	2366370 (60.9%)	959	737	28
Punjuba callejasii	Daly 5935 (NY)	ERS11697075	This study	0.235	0.008	1185683	780532 (65.8%)	993	961	20
Punjuba killipii	Palodorios 6252 (NY)	ERS11697082	This study	0.227	0.006	2140489	1842980 (86.1%)	991	957	23
Punjuba lehmannii	Escobar 7465 (NY)	ERS11697085	This study	0.169	0.007	3964907	3155020 (79.6%)	993	972	47
Punjuba racemiflora	Jimenez and Soares 3626 (USJ)	ERS11697094	This study	0.177	0.005	5467696	4445341 (81.3%)	994	976	40
Recordoxylon speciosum	Redden 5983 (US)	ERS11697291	This study	0.17	0.007	3672456	2416333 (65.8%)	970	806	54
Ricoa leptophylla	Tenorio and Manriquez 4067 (MEXU)	ERS11697257	This study	0.217	0.006	7125144	5829138 (81.8%)	995	971	28
Robrichia oldemanii	Bonadeu 706 (NY)	ERS11697207	This study	0.159	0.005	6805644	5463859 (80.3%)	993	963	46
Robrichia schomburgkii	Maguire 56534 (K)	ERS11697208	This study	0.133	0.008	2398649	2094588 (87.3%)	992	951	19
Samanea saman	Hughes 421 (FHO)	ERS4812936	Koenen et al. (*24*)	0.015	0.008	3134366	217362 (6.9%)	942	800	9
Sanjappa cynometroides	Krishnaraj 71501 (TBGT)	ERS11697293	This study	0.187	0.007	1909678	1523421 (79.8%)	994	930	30
Schizolobium parahyba	Klitgaard 694 (K)	ERS11697294	This study	0.165	0.005	2957135	1938266 (65.5%)	961	710	241
Schleinitzia insularum	Rinehart 17441 (K)	ERS11697295	This study	0.148	0.006	7684552	5971235 (77.7%)	967	779	370
Schleinitzia megaladenia	Ramos & Edaño 46708 (P)	ERS11697296	This study	0.228	0.008	1463032	1247392 (85.3%)	986	746	104
Schleinitzia novoguineensis	Chaplin 57 84 (FHO)	ERS4812937	Koenen et al. (*24*)	0.037	0.008	15322018	1503504 (9.8%)	979	829	41
Senegalia ataxacantha	Jongkind 10603 (WAG)	ERS4812938	Koenen et al. (*24*)	0.054	0.007	10724616	680898 (6.3%)	982	857	10
Senegalia bahiensis	de Queiroz 15499 (HUEFS)	ERS11697298	This study	0.158	0.006	5216922	4016660 (77%)	989	960	41

Senegalia borneensis	Ambriansyah AA1679 (L)	ERS11697299	This study	0.145	0.006	3048927	2292401 (75.2%)	991	961	48
Senegalia nigrescens	Barnes 536 (FHO)	ERS11697300	This study	0.146	0.005	8049990	6425427 (79.8%)	994	952	42
Senegalia pentagona	Jongkind 10670 (WAG)	ERS11697301	This study	0.146	0.006	3503774	2761310 (78.8%)	990	956	40
Senegalia sakalava	Koenen 215 (Z)	ERS4812939	Koenen et al. (*24*)	0.042	0.022	10377918	492909 (4.7%)	972	789	9
Senna cushina	Hughes 3121 (Z)	ERS11697303	This study	0.188	0.006	4109680	2554233 (62.2%)	969	795	52
Senna lasseigniana	Hughes 3086 (Z)	ERS11697304	This study	0.184	0.007	3435905	2083375 (60.6%)	962	771	33
Senna leandrii	Koenen 245 (G, K)	ERS11697305	This study	0.174	0.006	7120815	4396488 (61.7%)	967	823	67
Senna mollissima	Hughes 3150 (Z)	ERS11697306	This study	0.169	0.006	4492751	2659553 (59.2%)	965	786	48
Senna rugosa	de Queiroz 15592 (HUEFS)	ERS11697307	This study	0.186	0.005	4630639	2893029 (62.5%)	963	746	29
Senna velutina	Wood 26598 (K)	ERS11697308	This study	0.21	0.006	4123567	2664458 (64.6%)	965	767	43
Serianthes calycina	Barrabé 1158 (NOU)	ERS11697309	This study	0.14	0.006	3888534	2912175 (74.9%)	994	973	41
Serianthes nelsonii	Moore 1241 (L)	ERS4812940	Koenen et al. (*24*)	0.016	0.01	6673348	332881 (5%)	968	826	8
Sphinga acatlensis	Hughes 2112 (FHO)	ERS4812941	Koenen et al. (*24*)	0.02	0.011	8449227	450528 (5.3%)	976	849	11
Stachyothyrsus staudtii	van Andel 4054 (WAG)	ERS11697310	This study	0.2	0.006	7505359	5218629 (69.5%)	988	884	82
Strombocarpa ferox	Hughes 2618 (FHO)	ERS11697279	This study	0.163	0.005	5970858	4786921 (80.2%)	993	962	27
Strombocarpa strombulifera	Kiesling 4828 (NY)	ERS11697283	This study	0.147	0.006	3704512	3164148 (85.4%)	993	955	18
Stryphnodendron adstringens	de Queiroz 15580 (HUEFS)	ERS11697311	This study	0.157	0.005	4934723	3893905 (78.9%)	995	971	36
Stryphnodendron pulcherrimum	de Queiroz 15482 (HUEFS)	ERS4812942	Koenen et al. (*24*)	0.038	0.007	10894656	789387 (7.2%)	983	887	22
Stuhlmannia moavi	Keraudren and Aymonin 25628 (MO, P)	ERS11697314	This study	0.195	0.006	2677061	1587577 (59.3%)	971	774	44
Sympetalandra schmutzii	Schmutz 3764 (MO)	ERS11697315	This study	0.265	0.008	4370229	3347867 (76.6%)	948	614	224
Sympetalandra unijuga	Sidiyasa 1320 (K)	ERS11697316	This study	0.184	0.006	4942770	3832570 (77.5%)	935	676	286
Tachigali bracteolata	Mori 24793 (NY)	ERS11697317	This study	0.171	0.006	3385924	2220995 (65.6%)	984	851	77
Tachigali guianensis	Mori 22791 (NY)	ERS11697297	This study	0.193	0.006	4060573	2758090 (67.9%)	985	857	64
Tachigali odoratissima	Morim 562 (RB)	ERS4812943	Koenen et al. (*24*)	0.012	0.015	7331095	127595 (1.7%)	777	513	11

Tachigali paniculata	Henkel 657 (NY)	ERS11697318	This study	0.166	0.006	3664973	2347363 (64%)	984	845	68
Tachigali vasquezii	Neill 13998 (MO)	ERS11697319	This study	0.162	0.006	2660201	1794909 (67.5%)	984	840	47
Tara spinosa	Eastwood 36 (FHO)	ERS11697320	This study	0.26	0.009	4787915	2560721 (53.5%)	967	761	40
Tetrapleura tetraptera	Koenen 155 (WAG)	ERS4812944	Koenen et al. (*24*)	0.017	0.01	3936371	156722 (4%)	873	612	13
Tetrapterocarpon geayi	Koenen 231 (G, K)	ERS11697321	This study	0.227	0.006	6655137	4025160 (60.5%)	942	672	63
Thailentadopsis nitida	Kostermans 28234 (K)	ERS11697322	This study	0.23	0.005	3128413	2452697 (78.4%)	992	964	42
Thailentadopsis tenuis	Larsen & Larsen 33960 (K)	ERS11697323	This study	0.175	0.006	2236040	1901846 (85.1%)	992	955	20
Ticanto crista	Herendeen 1 V 99 3 (US)	ERS11697155	This study	0.285	0.007	6059665	3807786 (62.8%)	962	718	31
Umtiza listeriana	6th International Legume Conference 10 (JRAU)	ERS11697324	This study	0.208	0.006	5975233	3930911 (65.8%)	967	776	98
Vachellia erioloba	living collection Botanical Garden Zurich	ERS11697325	This study	0.153	0.006	10546334	7761717 (73.6%)	958	789	368
Vachellia farnesiana	living collection Botanical Garden Zurich	ERS11697326	This study	0.137	0.006	6609686	4552453 (68.9%)	962	836	432
Vachellia nilotica	Eastwood 117 (FHO)	ERS11697327	This study	0.136	0.007	7162697	5462646 (76.3%)	932	774	362
Vachellia tortilis	Koenen 603 (Z)	ERS4812945	Koenen et al. (*24*)	0.025	0.02	4704801	268630 (5.7%)	944	689	7
Vachellia viguieri	Koenen 199 (Z)	ERS4812946	Koenen et al. (*24*)	0.037	0.013	4330329	321296 (7.4%)	938	727	25
Viguieranthus glaber	Koenen 325 (Z)	ERS4812947	Koenen et al. (*24*)	0.047	0.013	9401416	671920 (7.1%)	986	837	13
Wallaceodendron celebicum	Flynn 7173 (NY)	ERS11697328	This study	0.158	0.005	6008113	4567544 (76%)	994	973	33
Xerocladia viridiramis	Kolberg and Tholkes HK2493 (WIND)	ERS11697329	This study	0.2	0.007	10389113	8119361 (78.2%)	994	959	40
Xylia evansii	Jongkind 9064 (WAG)	ERS11697330	This study	0.176	0.005	6704436	4864344 (72.6%)	989	914	56
Xylia hoffmannii	Koenen 402 (Z)	ERS4812948	Koenen et al. (*24*)	0.022	0.022	6632000	276998 (4.2%)	922	678	13
Xylia torreana	Maurin et al. RBN171 (JRAU)	ERS11697331	This study	0.172	0.006	2829838	2042888 (72.2%)	989	899	37
Zapoteca aculeata	Delinks 332 (NY)	ERS11697332	This study	0.228	0.007	2357164	1747778 (74.1%)	989	944	35
Zapoteca amazonica	Graham 270 (K)	ERS11697333	This study	0.291	0.009	292718	59749 (20.4%)	572	283	1
Zapoteca caracasana	Hughes 3071 (Z)	ERS4812949	Koenen et al. (*24*)	0.046	0.013	4703875	149537 (3.2%)	861	622	6
Zapoteca nervosa	Garcia 665 (NY)	ERS11697334	This study	0.317	0.009	3264117	2740207 (83.9%)	992	950	19

Zuccagnia punctata	Fortunato 5545 (MO, BAB)	ERS11697335	This study	0.301	0.009	3141469	1138914 (36.3%)	948	696	33
Zygia ampla	Prance 26527 (NY)	ERS11697336	This study	0.159	0.007	5076411	4296146 (84.6%)	993	948	34
Zygia basijuga	Wurdack 1937 (NY)	ERS11697337	This study	0.15	0.006	3283801	2665921 (81.2%)	992	958	33
Zygia bisingula	Ortega Mendoza 2561 (NY)	ERS11697338	This study	0.163	0.006	7127321	5671760 (79.6%)	994	956	43
Zygia cataractae	Bonadeu 647 (RB)	ERS11697339	This study	0.183	0.006	7213847	5544614 (76.9%)	994	950	53
Zygia claviflora	Iganci 841 (RB)	ERS4812950	Koenen et al. (*24*)	0.014	0.012	2246421	94113 (4.2%)	719	516	2
Zygia conzattii	Ortega 596 (K)	ERS11697340	This study	0.136	0.008	2174933	1843911 (84.8%)	991	944	26
Zygia inaequalis	Iganci 832 (RB)	ERS4812951	Koenen et al. (*24*)	0.012	0.015	5158351	250135 (4.8%)	956	771	7
Zygia inundata	Poncy 361 (NY)	ERS11697341	This study	0.168	0.006	6246417	5038331 (80.7%)	994	967	33
Zygia latifolia var. *communis*	Simon 1649 (CEN)	ERS11697342	This study	0.197	0.005	11242303	8987934 (79.9%)	993	945	50
Zygia longifolia	MacQueen 609 (K)	ERS11697343	This study	0.172	0.007	4463055	3773313 (84.5%)	990	943	29
Zygia morongii	Krapovickas 23604 (NY)	ERS11697344	This study	0.152	0.008	3929083	3227141 (82.1%)	993	922	37
Zygia oboliningoides	Krukoff 10969 (NY)	ERS11697345	This study	0.191	0.007	4781761	3877913 (81.1%)	992	950	30
Zygia ocumarensis	Stergios 14780 (NY)	ERS11697346	This study	0.158	0.006	4060642	3247793 (80%)	991	961	31
Zygia racemosa	Simon 1658 (CEN)	ERS4812952	Koenen et al. (*24*)	0.02	0.007	10085857	397461 (3.9%)	976	830	10
Zygia ramiflora	de Lima 2751 (NY)	ERS11697347	This study	0.388	0.011	257984	173996 (67.4%)	964	847	8
Zygia rhytidocarpa	Yuncker 8553 (NY)	ERS11697348	This study	0.205	0.005	7756608	6172803 (79.6%)	993	937	39
Zygia sabatieri	Sabatier 4838 (K)	ERS11697349	This study	0.204	0.007	5239416	4461118 (85.1%)	992	959	31
Zygia sp.	Coley and Kursar Tip917 (UT)	ERR776824	Koenen et al. (*24*)	0.07	0.008	1229023	508986 (41.4%)	960	802	36
Zygia unifoliolata	Wendt 4388 (K)	ERS11697350	This study	0.163	0.005	5591357	4408016 (78.8%)	993	950	50

Table S2. Outgroup species with numbers of genes matching the target genes used here which were recovered by BLAST and BLAT from genome sequences.

Taxon	Reference	BLAST	BLAT	Total
Arachis ipaensis	(*181*)	451	195	646
Cercis canadensis	(*182*)	694	167	861
Glycine max	(*183*)	623	135	758
Medicago truncultata	(*184*)	488	158	646
Nissolia schotii	(*185*)	527	191	718

Total unique genes	918

Table S3. Identities, ages, and placements of fossil constraints used for time-calibrating the Hybseq backbone phylogeny. SG = Stem Group; MRCA = Most Recent Common Ancestor

Node	Fossil taxon	Min age (Ma ago)	Reference
Caesalpinioideae SG	Unnamed bipinnate leaves	58	(*186, 187*)
Arcoa SG	*Arcoa*	48.5	(*188, 189*)
Senna/*Cassia* split	*Senna* fruits	37.8	(46)
Dinizia/Mimosoid clade MRCA	*Eumimosoidea plumosa* flowers, leaves and fruits	37.8	(190)
Vachellia/ingoid clade split	Flattened 16-celled polyads similar to *Vachellia, Albizia* and other ingoids	33.9	(191)
Acacia SG	*Acacia* polyads	23	(192)
Calliandra SG	*Calliandra* polyads	16	(193)

Table S4. GenBank accession numbers of new *Acacia* samples included in the metachronogram.

Species	Voucher	Isolate	*ETS*	*ITS*	*matK*	*psbA*	*trnL*
Acacia acellerata	CANB 0439085	JM2617				OQ096851	
Acacia aculeiformis	CANB 00495815	JM2620	OQ061170			OQ096852	
Acacia alleniana	ATSC 12075	JM1786	KC807379	KC955261	KC957120	KC955779	
Acacia ammophila	CBG 8801375	JM2934	KC807449	KC955351	KC957177	KC955942	KF048186
Acacia amoena	nindethana NS-7731	JM3553	KC807718		KC957393	KC956212	KF048399
Acacia anarthros	CANB 267736	JM2658				OQ096853	
Acacia aptaneura	B.R. Maslin 9275	JM9275	KC283908	KC200618	KC421945	KC283994	KC957947
Acacia awestoniana	CANB 00439392	JM2797				OQ096854	
Acacia barancana	M.D. Barrett 2941	JM3530	KC807705	KC955613	KC957379	KC956198	KF048387
Acacia barbinervis subsp. *barbinervis*	CANB 347200	JM2839		OQ099810			
Acacia bartleana	CANB 00585265	JM2837			KC421796	KC284049	
Acacia blaxellii	CBG 7908439	JM2848			OQ096834	OQ096855	
Acacia botrydion	CANB 310780	JM3154			OQ096835	OQ096856	
Acacia brachycarpa	CBG 7703159	JM2843			KC421798	KC284073	KC957802
Acacia brumalis	Kings Park BG 20041137	JM3557	KC807721	KC955627	KC957397	KC956215	
Acacia brunioides subsp. *brunioides*	CANB 00591436	JM2868			KC421808	KC284085	KC957817
Acacia burbidgeae	CANB 438914	JM2888	KC807415		KC957148	KC955909	KF048155
Acacia bynoeana	CANB 584837	JM2887	KC807414		KC957147	KC955908	KF048154
Acacia caerulescens	CANB 690511	JM2886	KC807413			KC955907	KF048153
Acacia caesaneura	B.R. Maslin 9367	JM9367		KC200680	KC421953	KC284088	KC957955
Acacia caesariata	CANB 439671	JM2885	KC807412	KC955325			
Acacia calcarata	CBG 68130	JM2883	OQ061173				
Acacia caleyi	CBG 8506136	JM2882	KC807410	KC955323		KC955905	
Acacia calyculata	A. Carroll 44	JM3866	OQ061171	OQ099811	OQ096836	OQ096857	OQ096885
Acacia cambagei	CANB 751003s2	JM3468	KC807659	KC955567	KC957348	KC956162	
Acacia carens	CANB 00586466	JM2876		OQ099812			
Acacia caroleae	Clarke 42ca (ironstone)	JM2067	KC283387	KC200696	KC421350	OQ096858	KC958138
Acacia cataractae	CANB 398079	JM2898	KC807423	KC955333		KC955917	KF048161
Acacia centrinervia	CBG 9709800	JM2893	KC807420	KC955330	KC957150	KC955914	KF048158
Acacia cerastes	CANB 586470	JM2892	KC807419	KC955329		KC955913	
Acacia chalkeri	CANB 611470.1	JM2891	KC807418	KC955328		KC955912	KF048157
Acacia chamaeleon	CANB 713022	JM2890	KC807417	KC955327	KC957149	KC955911	
Acacia chapmanii subsp. *chapmanii*	CANB 508614	JM3489	KC807672	KC955582	KF048718		KF048363
Acacia chippendalei	CANB 6362431	JM3488		KC955581	KF048717		KF048362
Acacia chrysocephala	CANB 615701	JM827		AF487760	AF523157		
Acacia chrysotricha	MELU 102548	JM2188			KC421400	KC284135	KC958196
Acacia cincinnata	D. Seigler 13864	JM1778	KC807378	KC955260	KC957119	KC955778	
Acacia cochlocarpa subsp. *velutinosa*	CANB 701493	JM3297	KC807558	KC955456	KC957269	KC956066	KF048264
Acacia convallium	CANB 587073	JM3302	KC807563	KC955461	KC957273	KC956070	KF048267
Acacia costiniana	ANBG 8100260	JM3609	KC807765		KC957445	KC956264	KF048442
Acacia covenyi	CANB 931401	JM2811		KC200732	KF048687	KC284169	KC957779
Acacia crassistipula	CANB 708338	JM3304	KC807564	KC955462	KC957274	KC956071	KF048268
Acacia crassuloides	CANB 496021	JM3308			KC957275	KC956073	

Acacia cretata	CANB 615670s2	JM1770	KC807377	KC955259	KC957118	KC955777	
Acacia crispula	CBG 8310256	JM3311	KC807566		KC957277	KC956074	
Acacia cummingiana	CANB 439560	JM3128		OQ099813		OQ096859	
Acacia dacrydioides	R.L. Barrett 6924	JM3538	KC807710	KC955618	KC957384	KC956203	KF048392
Acacia daviesioides	B.R. Maslin 5060	JM3650		KC955676	KC957458	KC956275	
Acacia delicatula	CANB 604250	JM3315	KC807568	KC955465	KC957279	KC956076	KF048270
Acacia deltoidea subsp. *deltoidea*	R.L. Barrett 6464	JM3519	KC807695	KC955603	KC957371	KC956188	KF048379
Acacia dermatophylla	CBG 8310145	JM3255	KC807539	KC955434		KC956039	
Acacia diaphyllodinea	CANB 439668	JM3252		KC955432		KF048578	
Acacia dilatata	CANB 315433.1	JM3248	KC807538	KC955431		KC956037	
Acacia diminuta	CANB 64868	JM3247		KC955430		KC956036	
Acacia disticha	CANB 472294	JM3246		KC955429		KC956035	KF048250
Acacia drewiana subsp. *drewiana*	CANB 579561	JM3243		KC955428		KC956034	
Acacia durabilis	CANB 301181	JM3240	KC807537	KC955427			
Acacia echinuliflora	CANB 277505	JM3232	KC807532	KC955420		KC956028	KF048248
Acacia effusifolia	CANB 472282	JM3416	KC807621	KC955526	KC957326	KC956127	KF048329
Acacia ericksoniae	CBG 8504995	JM3230	KF048832	KF048807	KF048698	KF048576	KF048247
Acacia erioclada	CANB 22503	JM3220	KC807525	KC955413	KC957247	KC956022	OQ096886
Acacia fasciculifera	CANB 615692	JM305		AF487769	AF274154		
Acacia filamentosa	CANB 604247	JM3419	KC807623	KC955528	KF048709		KF048331
Acacia flavescens	ATSC 13870	JM3612	KC807768		KC957447	KC956266	KF048445
Acacia flavipila var. *flavipila*	CANB 439690	JM3832	KC807881		KC957560	KC956389	KF048533
Acacia floribunda	CANB 804537	JM3636	OQ061182	OQ099823	OQ096839	OQ096882	OQ096891
Acacia floydii	A. Carroll N9 (69)	JM3773	KC807846	KC955732	KC957522	KC956348	KF048512
Acacia frigescens	ATSC 18071	JM3640	KC807776	KC955668	KC957449	KC956268	KF048448
Acacia froggattii	R. L. Barrett 6854	JM3515	KC807692		KC957369	KC956186	KF048377
Acacia fuscaneura	B.R. Maslin 9778	JM9778	KC283924	KC200827	KC421965	KC284272	KC957968
Acacia gemina	nindethana NS-5738	JM3560	KC807724	KC955630	KC957400	KC956218	
Acacia glaucocaesia	Leg. ign. 19.2	JM2859		KC200838	KC421806	KC284285	KC957815
Acacia gordonii	Mount Annan BG<br>884056	JM2138				OQ096860	
Acacia gracilenta	CANB 295365	JM3420	KC807624	KC955529		KC956128	KF048332
Acacia helicophylla	Alice Springs Desert<br>Park D 184073	JM3601	KC807759	KC955658	KC957438	KC956257	KF048436
Acacia hemignosta	M.D. Barrett 3164	JM3520	KC807696	KC955604	KC957372	KC956189	KF048380
Acacia humifusa	A. Carroll 47	JM3867	OQ061181	OQ099814	OQ096837		OQ096887
Acacia hylaoneura	A. Carroll 10	JM3744	KC807838	KC955724	KC957514	KC956340	KF048505
Acacia imitans	CANB 301185	JM3168	KC283866	KC200871	KC421898	KC284327	KC957902
Acacia incognita	CANB 702993	JM3425	KC807628	KC955532	KC957327	KC956132	KF048335
Acacia incurva	CANB 615644	JM873			AF523146		
Acacia incurvaneura	B.R. Maslin 9646	JM9646		KC200876	KC421962	KC284336	KC957965
Acacia ingrata	CANB 318837	JM3165			OQ096838	OQ096861	
Acacia inophloia	ATSC 17662	JM3588	KC807747	KC955647	KC957425	KC956244	KF048423
Acacia intricata	nindethana NS-17023	JM3571		KC955636	KC957410	KC956229	
Acacia jasperensis	CANB 492906	JM3428	KC807630	KC955535	KC957328	KC956135	KF048338
Acacia kelleri	M.D. Barrett 3103	JM3536	KC807708	KC955616	KC957382	KC956201	KF048390

Acacia kenneallyi	RL. Barrett 6543	JM3517	KC807694		KC957370	KC956187	
Acacia kettlewelliae	CANB 381592	JM3434	KC807635	KC955540	KC957330	KC956140	KF048342
Acacia kimberleyensis	R.L. Barrett 6700	JM3537	KC807709	KC955617	KC957383	KC956202	KF048391
Acacia kochii	CBG 9515260	JM3293			KC957268	KC956065	
Acacia lacertensis	CANB 352537.2	JM3291	OQ061172			OQ096862	
Acacia lachnophylla	CBG 9105475	JM3286	KC807555	KC955453	KC957266	KC956062	KF048263
Acacia lasiocarpa var. *lasiocarpa*	R.S. Cowan s.n. Seabird	JM3655	KC807788	KC955678	KC957462	KC956279	KF048461
Acacia latericola	Leg. Ign. s.n. 02	JM157		AF487774	AF523144		
Acacia latifolia	CANB 599309	JM3437	KC807636	KC955542	KC957332	KC956142	
Acacia latior	CANB 702995	JM3283	KC807552	KC955450	KC957263	KC956059	KF048261
Acacia leeuweniana	CANB 683104	JM3281	KC807550	KC955448	KC957261	KC956057	KF048259
Acacia leichhardtii	ATSC 16655	JM1986	KC283347	KC200907	KC421277	KC284361	KC958084
Acacia leiophylla	ANBG 7800105	JM3652	KC807785	KC955677	KC957460	KC956277	KF048458
Acacia lentiginea	R.L. Barrett 6534	JM3525	KC807701	KC955609	KC957377	KC956194	KF048384
Acacia leprosa var. *graveolens*	CANB 489990	JM3822	KC807876	KC955755	KC957556	KC956383	KF048531
Acacia leptoclada	DM 1	DM1		OQ099815			
Acacia leptostachya	nindethana NS-11917	JM3586	KC807745	KC955646	KC957423	KC956242	KF048421
Acacia limbata	ATSC 14659	JM3507		KC955598	KF048725		KF048373
Acacia linarioides	CANB 438790	JM3439	KC807638	KC955544		KC956144	KF048344
Acacia linophylla	Kings Park BG 19810051 item 339	JM1866	KC283285	KC200923	KC421217	KC284381	KC958002
Acacia littorea	nindethana NS-02217	JM3564	KC807728	KC955633		KC956222	KF048405
Acacia lucasii	CBG 9409629	JM3277	KC807548	KC955446	KC957259	KC956055	KF048257
Acacia lullfitziorum	CANB 577283	JM3441	OQ061174	OQ099816			
Acacia luteola	nindethana NS-1281	JM2100		OQ099817			
Acacia macraneura	B.R. Maslin 9178	JM9178		KC200936	KC421942	KC284403	KC957944
Acacia maidenii	CANB 712416	JM3004	KC807500	KC955385	KC957220	KC955994	KF048219
Acacia matthewii	Mount Annan BG 970294	JM2121				OQ096863	
Acacia megalantha	Alice Springs Desert Park 09 D 192656	JM3585	KC807744	KC955645	KC957422	KC956241	KF048420
Acacia merrallii	ANBG 7900559	JM3607	KC807764		KF048729		
Acacia merrickiae	ANBG 9401340	JM448			AF523150		
Acacia microcalyx	CANB 687948	JM3325		KC955471	KC957282	KC956080	KF048275
Acacia microneura	CANB 439391	JM3275				OQ096864	
Acacia mimica var. *mimica*	CANB 439682	JM3273	KC807545	KC955445		KC956052	
Acacia moirii subsp. *dasycarpa*	nindethana NS-824	JM2101	OQ061176			OQ096865	
Acacia mooreana	CANB 32368.7	JM3270		KC955444	KC957257	KC956050	KF048255
Acacia mulganeura	B.R. Maslin 9276	JM9276		KC200961	KC421946	KC284445	KC957948
Acacia multistipulosa	CANB 295343	JM3267		KC955443		KC956049	
Acacia neriifolia	Clarke 8n	JM2033	JF420291	OQ099818	KC421320	JF420181	KC958108
Acacia nitidula	CANB 429573	JM3265		KC955442		KC956048	
Acacia nodiflora var. *ferox*	nindethana NS-1450	JM3583	KC807742	KC955643	KC957420	KC956239	KF048418
Acacia octonervia	CBG 7908580	JM3264	KC807544	KC955441		KC956047	
Acacia ophiolithica	CANB 472156	JM3262				OQ096866	
Acacia oraria	A. Carroll 66	JM3722	KC807823		KC957501	KC956327	
Acacia orbifolia	CBG 8905404	JM3261		KC955439	KC957256	KC956045	KF048254

Acacia papulosa	CANB 472197	JM3323		KC955470	KC957281	KC956079	
Acacia peregrinalis	ATSC 17560	JM2366	KC283528	KC201007	KC421458	KC284512	KC958335
Acacia phaeocalyx	CBG 7902255	JM3322		KC955469	KC957280	KC956078	KF048274
Acacia pharangites	CANB 310707	JM3320		OQ099819			OQ096888
Acacia piligera	Mount Annan BG 20051482	JM2120	OQ061175				
Acacia pinguiculosa subsp. *pinguiculosa*	CANB 744592.1	JM3318		KC955466	KF048699		KF048271
Acacia plicata	CANB 550916	JM3219				OQ096867	
Acacia polystachya	CANB 507596	JM3327	KC807571	KC955473	KC957283	KC956081	KF048277
Acacia pteraneura	Leg. Ign. s.n. 38	JM3066	KC283804	KC201025	KC421828	KC284543	KC957838
Acacia ptychoclada	nindethana NS-17618	JM3645	KC807779		KC957454	KC956271	KF048453
Acacia pubescens	Mount Annan BG 884038s2	JM2275					OQ096889
Acacia pubirhachis	CANB 262921	JM3331	KC807574	KC955476	KF048701		KF048280
Acacia quadrimarginea	nindethana NS-3525s2	JM3570	KC807733		KC957409	KC956228	KF048410
Acacia quadrisulcata	nindethana NS-3525	JM3562	KC807726		KC957402	KC956220	
Acacia quinquenervia	CANB 300024	JM3334	KC807575	KC955477		OQ096868	
Acacia resinicostata	Mount Annan BG 961744	JM2147	OQ061177	OQ099820			
Acacia resinimarginea	CANB 708341	JM2924	KC807441	KC955343	KC957169	KC955934	KF048178
Acacia resinosa	CANB 531092	JM3335		OQ099821			OQ096890
Acacia restiacea	Kings Park BG 19850433 item 383	JM3582			KC957419	KC956238	KF048417
Acacia riceana	J.T. Miller 2001	JM3794		KC955742	KC957537	KC956363	KF048520
Acacia rossei	CANB 615569	JM209		AF487756	AF274162		
Acacia rothii	ATSC 13875	JM3604	KC807761	KC955660	KC957441	KC956260	KF048439
Acacia rotundifolia	nindethana NS-10659	JM3600	KC807758	KC955657	KC957437	KC956256	KF048435
Acacia saxatilis	CANB 438983	JM3209	OQ061178			OQ096869	
Acacia sclerosperma	MEL 252971	JM3697		OQ099822			
Acacia semirigida	ATSC 15852	JM3608			KC957444	KC956263	
Acacia sericata	CANB 586837	JM3339	KC807576	KC955478	KC957285	KC956083	KF048281
Acacia sericocarpa	CANB 438984	JM3344	KC807579	KC955480	KF048702		KF048284
Acacia sessilis	nindethana NS-5430	JM3599	KC807757		KC957436	KC956255	KF048434
Acacia setulifera	CANB 712946	JM3804	KC807860		KC957540	KC956366	
Acacia shirleyi	A. Carroll s.n. (mesa hitop)	JM3748	KC807841	KC955727	KC957517	KC956343	KF048508
Acacia signata	nindethana NS-no number (JMseed 1677)	JM3556	KC807720	KC955626	KC957396	KC956214	KF048401
Acacia sp.	A. Carroll 33	JM3721	OQ061179	OQ099824	OQ096840	OQ096870	OQ096892
Acacia sp.	A. Carroll s.n. (White Mountain NP)	JM3730	OQ061180		OQ096841	OQ096871	
Acacia sp.	A. Carroll 75	JM3757	OQ061183		OQ096842	OQ096872	OQ096893
Acacia sp.	A. Carroll N10 (70)	JM3768	OQ061185	OQ099825	OQ096843	OQ096873	OQ096894
Acacia sp.	A. Carroll 15	JM3775	OQ061186		OQ096844	OQ096874	
Acacia sp.	A. Carroll 45	JM3864	OQ061184	OQ099826	OQ096845	OQ096875	OQ096895
Acacia sp. HK4	HK 4	JM3118		OQ099828	OQ096846	OQ096876	OQ096897
Acacia sp. HK5	HK 5	JM3119		OQ099829	OQ096847	OQ096877	

Acacia sp. jancada	Mount Annan BG 842532	JM2304	OQ061189	OQ099830		OQ096878	OQ096898
Acacia sp. larigerava	Mount Annan BG 842732	JM2298	OQ061190	OQ099831		OQ096879	OQ096899
Acacia sp. maranoensis	Mount Annan BG 961757	JM2152			OQ096848	OQ096880	OQ096900
Acacia sp. nov. aff. barrettiorum	R.L. Barrett 6460	JM3518	OQ061188	OQ099832	OQ096849	OQ096881	OQ096901
Acacia sp. pubescens	Mount Annan BG 884038	JM2251	OQ061187	OQ099827		OQ096850	OQ096896
Acacia sp. Urandangie (L.Pedley 2025) Qld Herbarium	Alice Springs Desert Park DEA 12064 OG A 112424	JM3587	KC807746		KC957424	KC956243	KF048422
Acacia speckii	CANB 472143	JM3346	JM3346	KC955481	KC957287	KC956085	KF048285
Acacia spinosissima	CBG 8905438	JM3349		KC955483	KC957289	KC956087	OQ096902
Acacia spondylophylla	CANB 615683	JM934			AF523140		
Acacia startii	MEL 719650	JM3702		KC955702	KC957493	KC956316	KF048486
Acacia steedmanii	nindethana NS-no number (JMseed 1683)	JM3563	KC807727	KC955632	KC957403	KC956221	
Acacia stellaticeps	CANB 355149	JM3784	KC807851	KC955737	KC957529	KC956355	KF048514
Acacia stenophylla	MEL 2054404	JM3704	KC807815	KC955703	KF048741	OQ096883	OQ096903
Acacia subflexuosa	nindethana NS-7306	JM3597	KC807755	KC955655	KC957434	KC956253	KF048432
Acacia sublanata	CANB 527697	JM3353	KC807583	KC955486	KC957291	KC956089	KF048289
Acacia subporosa	CANB 209876	JM3783	KC807850	KC955736	KC957528	KC956354	
Acacia subracemosa	Herb. Gardens S.N.	JM3567			KC957406	KC956225	
Acacia subternata	CANB 439055	JM3806	KC807862	KC955747	KC957542	KC956368	KF048525
Acacia sulcata	nindethana NS-no number (JMseed 1690)	JM3598	KC807756	KC955656	KC957435	KC956254	KF048433
Acacia teretifolia	nindethana NS-no number (JMseed 1693)	JM3594	KC807752	KC955652	KC957431	KC956250	KF048429
Acacia trigonophylla	nindethana NS-10636	JM3593	KC807751	KC955651	KC957430	KC956249	KF048428
Acacia trinalis	CANB 471649	JM3226	KC807529	KC955417	KC957250	KC956025	KF048244
Acacia trinervata	nindethana NS-17619	JM3568	KC807731		KC957407	KC956226	KF048408
Acacia trineura	nindethana NS-03492	JM3590	KC807748	KC955649	KC957427	KC956246	KF048425
Acacia triptycha	nindethana NS-3669	JM3581	KC807741		KF048728		
Acacia ulicifolia var. *brownii*	nindethana NS-1782	JM3579	KC807740	KC955641	KC957417	KC956236	KF048415
Acacia ulicina	nindethana NS-no number (JMseed 1705)	JM3580		KC955642	KC957418	KC956237	KF048416
Acacia uliginosa	CANB 301069	JM3224	KC807528	KC955416			
Acacia uncifera	A. Carroll 22	JM3732	KC807829		KC957507	KC956333	KF048498
Acacia uncinella	nindethana NS-3606	JM3591	KC807749		KC957428	KC956247	KF048426
Acacia unguicula	CANB 439675	JM3223	KC807527	KC955415	KC957249	KC956024	KF048243
Acacia urophylla	nindethana NS-11638	JM3576	KC807737		KC957414	KC956233	KF048412
Acacia veronica	CBG 9408974	JM3780	KC807849	KC955735	KC957525	KC956351	
Acacia verticillata subsp. *verticillata*	CANB 476919	JM2950	KC807458	KC955356	KC957186	KC955951	KF048193
Acacia vincentii	R.L. Barrett 6080	JM3522	KC807698	KC955606	KC957374	KC956191	KF048382
Acacia vittata	CANB 510610	JM3221				OQ096884	
Acacia wardellii	CANB 383563	JM3355	KC807584	KC955487			
Acacia wickhamii	nindethana NS-12780	JM3578	KC807739	KC955640	KC957416	KC956235	KF048414
Acacia williamsonii	nindethana NS-7534	JM3577	KC807738	KC955639	KC957415	KC956234	KF048413

Table S5. New *Albizia* samples in the metachronogram, including European Nucleotide Archive (ENA) accession numbers.

Species name	Voucher	Herbarium	ENA accession code
Albizia acle	Villavicencio 27204	P	ERR9867593
Albizia amara subsp. *amara*	Semsei 3274	PRE	ERR9894926
Albizia amara subsp. *sericocephala*	Maurin 3368	JRAU	ERR9867635
Albizia androyensis	Randrianaivo et al. 1742	P	ERR9868582
Albizia antunesiana	Tinley 1454	PRE	ERR9867652
Albizia arenicola	Du Puy M296	K	ERR9870734
Albizia attopeuensis	Maxwell 92-747	P	ERR9867594
Albizia balabaka	Du Puy M123	K	ERR9870735
Albizia boinensis	Du Puy M523	WAG	ERR9868583
Albizia canescens	Forster PIF9580	L	ERR9870053
Albizia chevalieri	Okafov & Daramota 54622	FHO	ERR9867642
Albizia chinensis	But 79-100A	K	ERR9870736
Albizia commiphoroides	Phillipson 2781	P	ERR9868584
Albizia coriaria	Jongkind 2482	WAG	ERR9867655
Albizia corniculata	Shiu Ying Hu 10383	K	ERR9867601
Albizia crassiramea	Parnell 95-401	K	ERR9867602
Albizia divaricata	Randrianaivo 485	P	ERR9870728
Albizia duclouxii	Ducloux 6112	P	ERR9867598
Albizia evansii	Correia & Marques 843	PRE	ERR9867637
Albizia forbesii	Maurin 0228	JRAU	ERR9867656
Albizia gillardinii	Deschamps 256	K	ERR9868579
Albizia glaberrima	Maurin 2605	JRAU	ERR9867657
Albizia guillainii	Veillon 8151	NOU	ERR9867600
Albizia gummifera	Tawakali & Kaunda 436	PRE	ERR9867658
Albizia harveyi	Maurin 0773	JRAU	ERR9867659
Albizia isenbergiana	Verdcourt 3237B	K	ERR9868580
Albizia jaubertiana	Service Forestier Madagascar & Capuron 24619 sf	P	ERR9870729
Albizia julibrissin	Fumihiro Konta 18313	P	ERR9867634
Albizia kalkora	Xiao Bai-Zhong	K	ERR9870760
Albizia laurentii	Breteler 15764	WAG	ERR9867666
Albizia lebbeck	Nee 28682	NY	ERR9869143
Albizia lebbekoides	Larsen 31706	K	ERR9870761
Albizia mainaea	Du Puy M682	K	ERR9868581
Albizia malacophylla	de Wilde & de Wilde-Duyfjes 8944	PRE	ERR9867667
Albizia morombensis	Phillipson & Manjakahery 5985	P	ERR9870732
Albizia myriophylla	Larsen 33455	P	ERR9870769
Albizia numidarum	Labat 3552	K	ERR9870762
Albizia odorata	Du Puy M335	K	ERR9870763
Albizia odoratissima	Chow 80078	K	ERR9867604
Albizia pedicellata	Ambriansyah AA702	K	ERR9867610
Albizia perrieri	Nusbaumer LN 1876	K	ERR9870767
Albizia petersiana	Greenway & Kanuri 15362	PRE	ERR9867671
Albizia philippinensis	Ahern’s collector 215	P	ERR9867595

Albizia procera	Matthew RHT 63670	K	ERR9867611
Albizia rosulata	Ariffin Kalat BRUN 17375	K	ERR9867612
Albizia rufa	Zollinger 80	P	ERR9867599
Albizia salomonensis	Whitmore BSIP 4424	K	ERR9867613
Albizia schimperiana	Van Wyk BSA 2748	PRE	ERR9867677
Albizia sherriffii	Grierson 4314	K	ERR9867633
Albizia sp.	Koenen 387	G	ERR9895978
Albizia suluensis	Maurin 1810	JRAU	ERR9868575
Albizia tanganyicensis	Maurin 1972	JRAU	ERR9868576
Albizia tomentella	Leach & Dunlop 3801	L	ERR9867596
Albizia tulearensis	Service Forestier Madagascar & Capuron 29107 sf	P	ERR9870727
Albizia vaughanii	Vaughan 10763	K	ERR9870768
Albizia verrucosa	Service Forestier Madagascar & Capuron 24093 sf	P	ERR9870731
Albizia vialeana	Larsen 32204	P	ERR9867597

Table S6. European Nucleotide Archive (ENA) accession numbers of Dichrostachys group samples in the metachronogram.

**Species**	**Voucher**	**ENA accession code**
Alantsilodendron brevipes	Luckow 4324 (BH)	ERS13672742
Alantsilodendron glomeratum	Koenen 257 (G, K)	ERS13672731
Alantsilodendron humbertii	Allorge 2603 (P)	ERS13672713
Alantsilodendron mahafalense	Labat 3630 (P)	ERS13672714
Alantsilodendron pilosum	Koenen 203 (G, K)	ERS13672715
Alantsilodendron villosum	Koenen 409 (G, K)	ERS13672719
Dichrostachys akataensis	Koenen 410 (G, K)	ERS13672721
Dichrostachys arborescens	Labat 3553 (P)	ERS13672720
Dichrostachys bernieriana	Koenen 414 (G, K)	ERS13672722
Dichrostachys cinerea	Maurin 256 (JRAU)	ERS13672748
Dichrostachys dehiscens	Miller 1609613 (E)	ERS13672725
Dichrostachys dumetaria	Du Puy M130 (P)	ERS13672726
Dichrostachys kirkii	Thulin 4581 (UPS)	ERS13672727
Dichrostachys myriophylla	Koenen 301 (G, K)	ERS13672735
Dichrostachys paucifoliolata	Luckow 4157 (BH)	ERS13672729
Dichrostachys richardiana	Koenen 282 (G, K)	ERS13672741
Dichrostachys santapaui	Sebastine & Ramamurthy 13612 (K)	ERS13672732
Dichrostachys scottiana	Phillipson & Milijaana 3539 (G)	ERS13672733
Dichrostachys sp. 2 EK-2014	Koenen 360 (G, K)	ERS13672716
Dichrostachys sp. 3 EK-2014	Capuron 29131 sf (P)	ERS13672737
Dichrostachys tenuifolia	Labat 3579 (P)	ERS13672739
Dichrostachys unijuga	Koenen 242 (G, K)	ERS13672740
Dichrostachys venosa	Luckow 4339 (BH)	ERS13672744
Gagnebina bakoliae	Luckow 4225 (BH)	ERS13672746
Gagnebina bernieriana	Luckow 4243 (BH)	ERS13672750
Gagnebina calcicola	Luckow 4441 (BH)	ERS13672717
Gagnebina commersoniana	Koenen 224 (G, K)	ERS13672747
Gagnebina pterocarpa	Luckow s.n. (BH)	ERS13672718

Table S7. GenBank project and accession numbers of *Inga* samples included in the metachronogram.

Species	Collector	Herbarium	GenBank project: accession code
Inga acreana	Kursar, T.A.	CAY	PRJNA683762:SRR13356793
Inga acrocephala	Coley, P.D. & Kursar, T.A.	Coley/Kursar Lab, Univ. of Utah	PRJNA683762:SRR13356713
Inga acuminata	Coley, P.D. & Kursar, T.A.	STRI	PRJEB8722:ERR776788
Inga adenophylla	Dexter, K.G., Marcelo Pe-a, J.L., Pare-des, E. & Daza Yomona, A.	E, MOL	PRJNA683762:SRR13347369
Inga affinis	Dexter, K.G. & Villaroel, D.	E, USZ, RB, HUEFS	PRJNA683762:SRR13347358
Inga alata	Kursar, T.A.	CAY	PRJNA683762:SRR13386451
Inga alba	Coley, P.D. & Kursar, T.A.	Coley/Kursar Lab, Univ. of Utah	PRJEB8722:ERR776844
Inga allenii	Pennington, T.D.	K	PRJNA683762:SRR13347400
Inga aptera	Neves, D.M. et al.	E, K, HUEFS, BHCB, UESC	PRJNA683762:SRR13386462
Inga aria	Dexter, K.G.		PRJNA683762:SRR13386420
Inga auristellae	Coley, P.D. & Kursar, T.A.	Coley/Kursar Lab, Univ. of Utah	PRJEB8722:ERR776820
Inga barbata	Batista, M.		PRJNA683762:SRR13386385
Inga barbata.aff	Dexter, K.G.	DUKE	PRJNA683762:SRR13386413
Inga bicoloriflora	Coley, P.D. & Kursar, T.A.	INPA	PRJNA683762:SRR13241602
Inga blanchetiana	Vasconcelos, G.		PRJNA683762:SRR13386439
Inga bourgonii	Kursar, T.A.	CAY	PRJNA683762:SRR13386454
Inga brachyrhachis	Dexter, K.G.	DUKE, K	PRJNA683762:SRR13241580
Inga brachyrhachis.aff	Kursar, T.A.	living herbarium	PRJNA683762:SRR13356711
Inga brachystachys	Kursar, T.A.	CAY	PRJEB8722:ERR776795
Inga brachystachys.aff	Coley, P.D. & Kursar, T.A.	Coley/Kursar Lab, Univ. of Utah	PRJNA683762:SRR13347330
Inga brevialata	Neves, D.M.	E, K, HUEFS, BHCB, GOELDI	PRJNA683762:SRR13347365
Inga brevipes	Kursar, T.A.	CAY	PRJEB8722:ERR776779
Inga capitata type12	Endara, M.J.		PRJNA683762:SRR13356771
Inga capitata type33	Kursar, T.A.	living herbarium	PRJNA683762:SRR13356715
Inga capitata type35	Coley, P.D. & Kursar, T.A.	CAY	PRJEB8722:ERR776828
Inga capitata.aff	Neves, D.M. et al.	E, K, HUEFS, BHCB, UESC	PRJNA683762:SRR13347377
Inga cayennensis	Kursar, T.A.	Coley/Kursar Lab, Univ. of Utah	PRJNA683762:SRR13356797
Inga chartacea	Dexter, K.G.	DUKE	PRJNA683762:SRR13347356
Inga chocoensis	Pennington, T.D.	K	PRJNA683762:SRR13347397
Inga chrysantha	Dexter, K.G.	DUKE, K	PRJNA683762:SRR13241668
Inga ciliata	Batista, M.		PRJNA683762:SRR13386386
Inga cinnamomea	Coley, P.D. & Kursar, T.A.	Coley/Kursar Lab, Univ. of Utah	PRJNA683762:SRR13356796
Inga cocleensis	Coley, P.D. & Kursar, T.A.	STRI	PRJEB8722:ERR776800
Inga coleyana	Coley, P.D. & Kursar, T.A.	living herbarium	PRJNA683762:SRR13241644
Inga congesta	Garcia, F.C.P.		PRJNA683762:SRR13386456
Inga cordatoalata	Dexter, K.G.	DUKE, K	PRJNA683762:SRR13241666
Inga cordistipula	Fernandes, J.M.		PRJNA683762:SRR13386432
Inga coruscans	Kursar, T.A. & Coley, P.D.	MOL	PRJNA683762:SRR13241641

Inga cylindrica	Kursar, T.A. & Coley, P.D.	MOL	PRJNA683762:SRR13347314
Inga cylindrica.aff	Batista, M.		PRJNA683762:SRR13386384
Inga densiflora	Dexter, K.G.	DUKE, K	PRJNA683762:SRR13347343
Inga edulis	Garcia, F.C.P.	HRCB, K	PRJNA683762:SRR13386460
Inga exfoliata	Neves, D.M. et al.	E, K, HUEFS, BHCB	PRJNA683762:SRR13386411
Inga fastuosa	Coley, P.D. & Kursar, T.A.	Coley/Kursar Lab, Univ. of Utah	PRJNA683762:SRR13356746
Inga feuillei	Pennington, R.T.	K	PRJNA683762:SRR13386383
Inga flagelliformis	Coley, P.D. & Kursar, T.A.	Coley/Kursar Lab, Univ. of Utah	PRJEB8722:ERR776787
Inga fosteriana	Dexter, K.G.		PRJNA683762:SRR13241599
Inga globularis	Neves, D.M. et al.	E, K, HUEFS, BHCB	PRJNA683762:SRR13386378
Inga glomeriflora	Dexter, K.G. & Souza, P.	PPBIO, E, RB	PRJNA683762:SRR13347360
Inga goldmanii	Coley, P.D. & Kursar, T.A.	STRI	PRJEB8722:ERR776791
Inga golfodulcensis	Pennington, T.D. et al.	K	PRJNA683762:SRR13347401
Inga gracilifolia	Coley, P.D. & Kursar, T.A.	CAY	PRJEB8722:ERR776807
Inga grandiflora	Coley, P.D. & Kursar, T.A.	Coley/Kursar Lab, Univ. of Utah	PRJEB8722:ERR776819
Inga heterophylla	Dexter, K.G.		PRJNA683762:SRR13356785
Inga hispida	Fernandes, J.M.		PRJNA683762:SRR13386430
Inga huberi	Coley, P.D. & Kursar, T.A.	Coley/Kursar Lab, Univ. of Utah	PRJEB8722:ERR776810
Inga ilta	Pennington, T.D.	K	PRJNA683762:SRR13347394
Inga ingoides	Dexter, K.G.	DUKE	PRJNA683762:SRR13386426
Inga jenmani	Coley, P.D. & Kursar, T.A.	Coley/Kursar Lab, Univ. of Utah	PRJNA683762:SRR13241674
Inga jinicuil	Pennington, T.D.	K	PRJNA683762:SRR13347402
Inga klugii	Dexter, K.G.	DUKE, K	PRJNA683762:SRR13241654
Inga kursarii	Coley, P.D. & Kursar, T.A.	living herbarium	PRJNA683762:SRR13356729
Inga lanceifolia	Garcia, F.C.P. et al.		PRJNA683762:SRR13386458
Inga lateriflora	Kursar, T.A.	Coley/Kursar Lab, Univ. of Utah	PRJNA683762:SRR13356791
Inga laurina	Dexter, K.G.	DUKE, K	PRJEB8722:ERR776816
Inga laurina.aff	Dexter, K.G.	DUKE	PRJNA683762:SRR13347354
Inga leiocalycina	Dexter, K.G.	DUKE, K	PRJEB8722:ERR776815
Inga leiocalycina.aff	Coley, P.D. & Kursar, T.A.	living herbarium	PRJNA683762:SRR13356732
Inga lenticellata	Kulkamp, J.		PRJNA683762:SRR13386437
Inga lentiscifolia	Neves, D.M. et al.	E, K, HUEFS, BHCB	PRJNA683762:SRR13386465
Inga leptantha	Fernandes, J.M.		PRJNA683762:SRR13386435
Inga lineata	Dexter, K.G.	DUKE, K	PRJNA683762:SRR13347374
Inga litoralis	Pennington, T.D.	K	PRJNA683762:SRR13347399
Inga lomatophylla	Kursar, T.A.	CAY	PRJNA683762:SRR13356724
Inga longiflora	Coley, P.D. & Kursar, T.A.	Coley/Kursar Lab, Univ. of Utah	PRJEB8722:ERR776847
Inga longipedunculata	Coley, P.D. & Kursar, T.A.	Coley/Kursar Lab, Univ. of Utah	PRJNA683762:SRR13356801
Inga longipes	Dexter, K.G.	DUKE, K	PRJNA683762:SRR13347395
Inga loubryana	Coley, P.D. & Kursar, T.A.	CAY	PRJEB8722:ERR776786

Inga luschnathiana	Fernandes, J.M.		PRJNA683762:SRR13386428
Inga macrophylla	Dexter, K.G.	DUKE, K, MOL	PRJNA683762:SRR13347353
Inga marginata	Coley, P.D. & Kursar, T.A.	STRI	PRJEB8722:ERR776829
Inga maritima	Garcia, F.C.P.		PRJNA683762:SRR13386457
Inga megaphylla	Dexter, K.G.	DUKE, K	PRJNA683762:SRR13347355
Inga melinonis	Coley, P.D. & Kursar, T.A.	Coley/Kursar Lab, Univ. of Utah	PRJNA683762:SRR13356735
Inga microcoma	Coley, P.D. & Kursar, T.A.	living herbarium	PRJNA683762:SRR13241645
Inga multinervis	Kursar, T.A.	living herbarium	PRJNA683762:SRR13356751
Inga nobilis	Dexter, K.G.	DUKE	PRJNA683762:SRR13241579
Inga nouragensis	Coley, P.D. & Kursar, T.A.	Coley/Kursar Lab, Univ. of Utah	PRJEB8722:ERR776839
Inga obidensis	Dexter, K.G. & Correa, C.	PPBIO, E, RB, HUEFS	PRJNA683762:SRR13347363
Inga oerstediana	Pennington, T.D. et al.	K	PRJNA683762:SRR13347403
Inga oerstediana.aff	Pennington, T.D.	K	PRJNA683762:SRR13356764
Inga ornata	Pennington, T.D.	K	PRJNA683762:SRR13356762
Inga paraensis	Coley, P.D. & Kursar, T.A.	Coley/Kursar Lab, Univ. of Utah	PRJNA683762:SRR13356792
Inga pauciflora	Kursar, T.A. & Coley, P.D.	Coley/Kursar Lab, Univ. of Utah	PRJNA683762:SRR13356766
Inga pezizifera	Kursar, T.A.	Coley/Kursar Lab, Univ. of Utah	PRJNA683762:SRR13356779
Inga pezizifera.aff	Coley, P.D. & Kursar, T.A.	STRI	PRJEB8722:ERR776830
Inga pilosula	Dexter, K.G. & Villaroel, D.	E, USZ, RB, HUEFS, K	PRJNA683762:SRR13347357
Inga pitmanii	Dexter, K.G.	DUKE, K	PRJNA683762:SRR13386418
Inga pitmanii.aff	Dexter, K.G.	DUKE	PRJNA683762:SRR13347373
Inga platyptera	Siqueira, L.C.		PRJNA683762:SRR13386398
Inga poeppigiana	Coley, P.D. & Kursar, T.A.	Coley/Kursar Lab, Univ. of Utah	PRJNA683762:SRR13356799
Inga porcata	Dexter, K.G.	DUKE, K, MOL	PRJNA683762:SRR13241660
Inga pruriens	Dexter, K.G.	DUKE	PRJNA683762:SRR13347367
Inga psittacorum	Dexter, K.G.	DUKE, K	PRJNA683762:SRR13347321
Inga punctata	Coley, P.D. & Kursar, T.A.	STRI	PRJEB8722:ERR776794
Inga retinocarpa	Coley, P.D. & Kursar, T.A.	Coley/Kursar Lab, Univ. of Utah	PRJEB8722:ERR776833
Inga rhynchocalyx	Coley, P.D. & Kursar, T.A.	INPA	PRJNA683762:SRR13386393
Inga rubiginosa	Coley, P.D. & Kursar, T.A.	Coley/Kursar Lab, Univ. of Utah	PRJNA683762:SRR13356757
Inga ruiziana	Coley, P.D. & Kursar, T.A.	STRI	PRJEB8722:ERR776818
Inga saltensis	Pennington, T.D.	K	PRJNA683762:SRR13356760
Inga sapindoides	Kursar, T.A.	living herbarium	PRJNA683762:SRR13356719
Inga schinifolia	Siqueira, L.C.		PRJNA683762:SRR13386396
Inga sellowiana	Baitelo, J.B.		PRJNA683762:SRR13386438
Inga sertulifera	Dexter, K.G.	DUKE, K	PRJNA683762:SRR13241655
Inga sessilis	Siqueira, L.C.		PRJNA683762:SRR13386399
Inga setosa	Dexter, K.G.	DUKE, K	PRJEB8722:ERR776814
Inga sp. LA17	Dexter, K.G.	DUKE, K	PRJNA683762:SRR13241656
Inga sp. LA41	Dexter, K.G.	DUKE, K	PRJNA683762:SRR13241665

Inga sp. LA48	Dexter, K.G.		PRJNA683762:SRR13241586
Inga sp. M18	Dexter, K.G.	DUKE, K	PRJNA683762:SRR13241670
Inga sp. M25	Coley, P.D. & Kursar, T.A.	INPA	PRJNA683762:SRR13241581
Inga sp. M27	Dexter, K.G. & Souza, P.	PPBIO, E, RB, HUEFS	PRJNA683762:SRR13347361
Inga sp. M30	Coley, P.D. & Kursar, T.A.	INPA	PRJNA683762:SRR13241583
Inga sp. M34	Coley, P.D. & Kursar, T.A.	INPA	PRJNA683762:SRR13241609
Inga sp. M71	Coley, P.D. & Kursar, T.A.	INPA	PRJNA683762:SRR13241614
Inga sp. T18	Coley, P.D. & Kursar, T.A.	living herbarium	PRJNA683762:SRR13356754
Inga sp. T21	Kursar, T.A.	living herbarium	PRJNA683762:SRR13347381
Inga sp. T50	Coley, P.D. & Kursar, T.A.	living herbarium	PRJNA683762:SRR13347388
Inga sp. T51	Coley, P.D. & Kursar, T.A.	living herbarium	PRJNA683762:SRR13241646
Inga sp. T82	Coley, P.D. & Kursar, T.A.	living herbarium	PRJNA683762:SRR13356733
Inga sp. T85	Coley, P.D. & Kursar, T.A.	living herbarium	PRJNA683762:SRR13241648
Inga sp. T88	Coley, P.D. & Kursar, T.A.	living herbarium	PRJNA683762:SRR13347391
Inga spectabilis	Coley, P.D. & Kursar, T.A.	STRI	PRJEB8722:ERR776808
Inga splendens	Coley, P.D. & Kursar, T.A.	Coley/Kursar Lab, Univ. of Utah	PRJNA683762:SRR13356795
Inga steinbachii	Dexter, K.G.	DUKE, K, MOL	PRJNA683762:SRR13241627
Inga stenoptera	Dexter, K.G.	DUKE, K	PRJNA683762:SRR13386415
Inga stipulacea	Dexter, K.G.	DUKE, K, MOL	PRJNA683762:SRR13347310
Inga stipularis	Coley, P.D. & Kursar, T.A.	Coley/Kursar Lab, Univ. of Utah	PRJEB8722:ERR776821
Inga striata	Kursar, T.A.	Coley/Kursar Lab, Univ. of Utah	PRJNA683762:SRR13356802
Inga suaveolens	Dexter, K.G.	DUKE, K	PRJNA683762:SRR13241647
Inga subcapitata	Dexter, K.G.	DUKE, K	PRJNA683762:SRR13241590
Inga suberosa	Coley, P.D. & Kursar, T.A.	INPA	PRJNA683762:SRR13241620
Inga subnuda	Fernandes, J.M.		PRJNA683762:SRR13386427
Inga tenuicalyx	Dexter, K.G., Linares Palomino, R., Marcelo Pe-a, J.L., Daza Yomona, A. & Pennington, R.T.	E, MOL	PRJNA683762:SRR13347370
Inga tenuis	Garcia, F.C.P.		PRJNA683762:SRR13386459
Inga tenuistipula	Dexter, K.G.	DUKE, K	PRJEB8722:ERR776831
Inga tessmannii	Castro, W.		PRJNA683762:SRR13347317
Inga thibaudiana	Kursar, T.A. & Coley, P.D.	STRI	PRJEB8722:ERR776796
Inga tocacheana	Dexter, K.G.	DUKE	PRJNA683762:SRR13241652
Inga tomentosa	Dexter, K.G.	DUKE	PRJNA683762:SRR13241616
Inga tonduzii	Pennington, T.D.	K	PRJNA683762:SRR13347398
Inga tripa	Vasconcelos, G.		PRJNA683762:SRR13386441
Inga ulei	Coley, P.D. & Kursar, T.A.	INPA	PRJNA683762:SRR13241619
Inga umbellifera	Dexter, K.G.	DUKE, MOL	PRJEB8722:ERR776782
Inga umbellifera.aff	Kursar, T.A.	Coley/Kursar Lab, Univ. of Utah	PRJEB8722:ERR776781
Inga umbratica	Dexter, K.G.	DUKE	PRJNA683762:SRR13241653
Inga velutina	Coley, P.D. & Kursar, T.A.	living herbarium	PRJNA683762:SRR13347383
Inga velutina.aff	Pennington, T.D.	K	PRJNA683762:SRR13356761
Inga venusta	Dexter, K.G.	DUKE, K	PRJNA683762:SRR13241592
Inga vera	Coley, P.D. & Kursar, T.A.	STRI	PRJEB8722:ERR776817

Inga virgultosa	Coley, P.D. & Kursar, T.A.	Coley/Kursar Lab, Univ. of Utah	PRJNA683762:SRR13356798
Inga vismiifolia	Dexter, K.G.	DUKE, K	PRJNA683762:SRR13386409
Inga vulpina	Siqueira, L.C.		PRJNA683762:SRR13386402
Inga yasuniana	Dexter, K.G.	DUKE, K	PRJNA683762:SRR13347384

Table S8. GenBank accession numbers of *Leucaena* samples included in the metachronogram.

Sample	GenBank accession number	Voucher
Leucaena collinsii	SRX2719653	OXF Forestry Seed Bank seedlot 51/88/01
Leucaena confertiflora subsp. *adenotheloidia*	SRX2719652	OXF Forestry Seed Bank seedlot 87/94/01
Leucaena cruziana	SRX2719651	OXF Forestry Seed Bank seedlot 43/85/16
Leucaena cuspidata	SRX2719650	OXF Forestry Seed Bank seedlot 83/94
Leucaena diversifolia	SRX2719649	OXF Forestry Seed Bank seedlot 82/92/02
Leucaena esculenta	SRX2719648	OXF Forestry Seed Bank seedlot 47/87/01
Leucaena greggii	SRX2719647	OXF Forestry Seed Bank seedlot 19/86/01
Leucaena involucrata	SRX2719646	OXF Forestry Seed Bank seedlot 87/92
Leucaena lanceolata	SRX2719645	OXF Forestry Seed Bank seedlot 44/85/02
Leucaena lempirana	SRX2719644	OXF Forestry Seed Bank seedlot 6/91/01
Leucaena leucocephala subsp. *leucocephala*	SRX2719642	OXF Forestry Seed Bank seedlot 80/92/02
Leucaena macrophylla subsp. *macrophylla*	SRX2719640	OXF Forestry Seed Bank seedlot 55/88
Leucaena magnifica	SRX2719639	OXF Forestry Seed Bank seedlot 58/88/01
Leucaena matudae	SRX2719638	OXF Forestry Seed Bank seedlot 49/87/02
Leucaena multicapitula	SRX2719637	OXF Forestry Seed Bank seedlot 81/87
Leucaena pallida	SRX2719636	OXF Forestry Seed Bank seedlot 78/92/02
Leucaena pueblana	SRX2719610	Hughes 2610 (FHO)
Leucaena pulverulenta	SRX2719635	OXF Forestry Seed Bank seedlot 84/87/05
Leucaena retusa	SRX2719634	OXF Forestry Seed Bank seedlot 23/86/01
Leucaena salvadorensis	SRX2719633	OXF Forestry Seed Bank seedlot 7/91/26
Leucaena shannonii	SRX2719632	OXF Forestry Seed Bank seedlot 22/83/01
Leucaena trichandra	SRX2719631	OXF Forestry Seed Bank seedlot 4/91/15
Leucaena trichodes	SRX2719630	OXF Forestry Seed Bank seedlot 61/88
Leucaena zacapana	SRX2719629	OXF Forestry Seed Bank seedlot 57/88/01

Table S9. GenBank accession numbers of new non-Madagascan *Mimosa* samples included in the metachronogram.

Name in metachronogram	Species name	Voucher	Herbarium	GenBank accession code
Mimosa_aff_custodis_QUIR6	*Mimosa* sp.	Morales 1629	BAB	ON530940
Mimosa_aff_pigra_MFS3066	*Mimosa pigra*	Simon 3066	CEN	ON530935
Mimosa_afranioi_ACS6669	*Mimosa afranioi*	Sevilha 6669	MG	OM891221
Mimosa_burchellii_MFS2826	*Mimosa burchellii*	Simon 2826	MA	OM891222
Mimosa_burkartii_BK1	*Mimosa burkartii*	Fortunato 12720	BAB	ON530912
Mimosa_callydryas_MFS3568	*Mimosa callidryas*	Simon 3568	CEN	ON530936
Mimosa_campicola_LSBJ354	*Mimosa campicola*	Jordão 354	BA	OM891223
Mimosa_crumenarioides_LSBJ215	*Mimosa crumenarioides*	Jordão 215	BA	OM891224
Mimosa_demissa_MM5128	*Mimosa demissa*	Mendoza 5128	CEN	ON530932
Mimosa_distans_MFS2462	*Mimosa distans*	Simon 2462	CEN	ON530924
Mimosa_diversipila_MM2026_DE	*Mimosa diversipila*	Morales 2026	BAB	ON530911
Mimosa_dolens_foliolosa_DE13	*Mimosa dolens* var. *foliolosa*	Morales 1162	BAB	ON530920
Mimosa_dolens_rudis_BOTU3	*Mimosa dolens* var. *rudis*	Morales 1616	BAB	ON530919
Mimosa_dryandroides_LJ427	*Mimosa dryandroides*	Jordão 427	RB	ON530922
Mimosa_emaensis_LSBJ424	*Mimosa emaensis*	Jordão 424	GO	OM891226
Mimosa_falcipinna_MM4980	*Mimosa falcipinna*	Mendoza 4980	CEN	ON530925
Mimosa_filiformis_LSBJ303	*Mimosa gracilis* subsp. *fili-formis*	Jordão 303	MG	OM891227
Mimosa_flabellifolia_LSBJ439	*Mimosa flabellifolia*	Jordão 439	TO	OM891228
Mimosa_flavocaesia_MFS3193	*Mimosa flavocaesia*	Simon 3193	CEN	ON530933
Mimosa_gracilis_brevissima_MFS2606	*Mimosa gracilis* subsp. *bre-vissima*	Simon 2606	GO	OM891230
Mimosa_gracilis_gracilis_LSBJ300	*Mimosa gracilis* subsp. *gracilis*	Jordão 300	MG	OM891231
Mimosa_hatschbachii_EL4109	*Mimosa hatschbachii*	Lozano 4109	MBM	ON530915
Mimosa_intricata_INT8	*Mimosa intricata*	Fortunato 12798	BAB	ON530914
Mimosa_longipes_MFS2467	*Mimosa longipes*	Simon 2467	CEN	ON530930
Mimosa_macrocephala_MFS2475	*Mimosa macrocephala*	Simon 2475	CEN	ON530926
Mimosa_maracayuensis_LSBJ410	*Mimosa maracayuensis*	Jordão 410	MS	OM891232
Mimosa_micropteris_MM2124	*Mimosa micropteris*	Morales 2124	BAB	ON530938
Mimosa_multiceps_LSBJ419	*Mimosa multiceps*	Jordão 419	MS	OM891233
Mimosa_osmarii_LSBJ279	*Mimosa osmarii*	Jordão 279	MG	OM891234
Mimosa_pabstiana_ACS6809	*Mimosa pabstiana*	Sevilha 6809	MG	MT044447
Mimosa_palmetorum_DMN1581	*Mimosa palmetorum*	Neves 1581	BA	OM891235
Mimosa_paraibana_MFS2813	*Mimosa paraibana*	Simon 2813	MA	MT044446
Mimosa_phyllodinea_LSBJ394	*Mimosa phyllodinea*	Jordão 394	GO	OM891237
Mimosa_pinetorum_LSBJ243	*Mimosa pinetorum*	Jordão 243	BA	OM891238
Mimosa_rava_MFS2658	*Mimosa rava*	Simon 2658	CEN	ON530928
Mimosa_reduviosa_TBC3846	*Mimosa reduviosa*	Cavalcanti 3846	CEN	ON530941
Mimosa_regnellii_MM2118	*Mimosa regnellii*	Morales 2118	BAB	ON530937
Mimosa_regnellii_supersetosa_ MM2130	*Mimosa regnellii* var. *super-setosa*	Morales 2130	BAB	ON530939
Mimosa_selloi_LSBJ430	*Mimosa selloi*	Jordão 430	SP	OM891239
Mimosa_serpens_EDL2678	*Mimosa serpens*	Lozano 2678	PR	OM891240
Mimosa_sobralii_A17	*Mimosa sobralii*	Morales 1060d	BAB	ON530913

Mimosa_sp_nov_pachyc_MFS3060	*Mimosa* sp.	Simon 3060	CEN	ON530927
Mimosa_sp_nov_polycepha_MFS3061	*Mimosa pseudoracemosa*	Simon 3061	CEN	ON530934
Mimosa_sp_nov_TBC3839	*Mimosa* sp.	Cavalcanti 3839	CEN	ON530918
Mimosa_sp_stipel_PSE4	*Mimosa* sp.	Fortunato 12835	BAB	ON530917
Mimosa_subenervis_LSBJ336	*Mimosa subenervis*	Jordão 336	BA	OM891241
Mimosa_suburbana_LSBJ393	*Mimosa suburbana*	Jordão 393	DF	OM891242
Mimosa_supravisa_LSBJ442	*Mimosa supravisa*	Jordão 442	GO	OM891243
Mimosa_thomista_MB2768	*Mimosa thomista*	Brotto 2768	MBM	ON530923

Table S10. European Nucleotide Archive (ENA) accession numbers of Madagascan *Mimosa* samples in the metachronogram.

**Species**	**Voucher**	**ENA accession code**
Mimosa andringitrensis	Humbert 13493 (P)	ERS13672761
Mimosa capuronii	Service Forestier Madagascar SF 23276 (P)	ERS13672768
Mimosa dumetaria	Service Forestier Madagascar SF 13846 (P)	ERS13672767
Mimosa grandidieri	Koenen 228 (G, K)	ERS13672770
Mimosa haavoa	Service Forestier Madagascar SF 7235 (P)	ERS13672756
Mimosa hafomantsina	Lewis 2138 (K)	ERS13672759
Mimosa hildebrandtii	Peltier 5890 (P)	ERS13672752
Mimosa himalayana	Panigrahi 2990 (P)	ERS13672783
Mimosa ikondensis	Humbert 19886 (P)	ERS13672785
Mimosa kitrokala	Humbert 20763 (P)	ERS13672789
Mimosa latispinosa	Koenen 269 (G, K)	ERS13672791
Mimosa levenensis	Koenen 355 (G, K)	ERS13672779
Mimosa mahilakensis	Service Forestier Madagascar SF 24596 (P)	ERS13672758
Mimosa manomboensis	Labat 3628 (P)	ERS13672760
Mimosa menabeensis	Service Forestier 12565 (P)	ERS13672755
Mimosa mossambicensis	Brummitt 8896 (K)	ERS13672784
Mimosa nossibiensis	Du Puy M899 (K)	ERS13672780
Mimosa onilahensis	DuPuy M434 (P)	ERS13672787
Mimosa planitei	Nusbaumer 1875 (G)	ERS13672776
Mimosa psoralea	Rabevohitra 2088 (P)	ERS13672772
Mimosa rokatavensis	Reserves Naturelles Madagascar RN 5023 (P)	ERS13672781
Mimosa rubicaulis	Williams 8191 (K)	ERS13672764
Mimosa savokaea	DuPuy M173 (P)	ERS13672766
Mimosa sp.	Koenen 188 (G, K)	ERS13672765
Mimosa suffruticosa	Labat 3549 (P)	ERS13672763
Mimosa uncinata	Decary 14429 (P)	ERS13672778
Mimosa vilersii	Labat 3020 (P)	ERS13672757
Mimosa volubilis	Koenen 407 (G, K)	ERS13672771
Mimosa waterlotii	Rakotomalale 52 (G)	ERS13672774

Table S11. GenBank accession numbers of Parkia clade samples included in the metachronogram.

**Species**	**GenBank accession code**
Anadenanthera peregrina	AF521814
Parkia bahiae	KY046204
Parkia biglobosa	KY046215
Parkia decussata	KY118189
Parkia discolor	KY045957
Parkia leiophylla	KY046207
Parkia madagascariensis	KY046208
Parkia multijuga	KY046209
Parkia nitida	KY046210
Parkia panurensis	KY046211
Parkia pendula	KY046083
Parkia platycephala	KY045958
Parkia sumatrana	KY046214
Parkia ulei	KY046045
Parkia velutina	KY046084
Vachellia bidwillii	MK923636
Vachellia clarksoniana	MK923634
Vachellia ditricha	MK923632
Vachellia douglasica	MK923631
Vachellia pachyphloia	MK923630
Vachellia suberosa	MK923629
Vachellia sutherlandii	MK923627
Vachellia valida	MK923623

Table S12. Ancient phylogenetic turnover variation partitioning results obtained with the metachronogram per region. The relative contribution of each predictor variable is shown, as well as the total variance explained by the full model.

pantropics

**Cut-off**	**Full**	**5 Ma**	**10 Ma**	**20 Ma**
**Correlation with taxonomic turnover**	0.70	0.62	0.56	0.35
**Correlation with full phylogenetic turnover**	1.00	0.99	0.95	0.64
**Variation partitioning**				
**Climatic distance**	0.04	0.03	0.03	-
**Interaction**	0.31	0.29	0.26	-
**Spatial distance**	0.64	0.67	0.71	-
**Percentage variance explained**	54.47	51.68	47.01	-

North America

Cut-off	Full	5 Ma	10 Ma	20 Ma
Correlation with taxonomic turnover	0.70	0.61	0.52	0.41
Correlation with full phylogenetic turnover	1.00	0.99	0.96	0.87
**Variation partitioning**				
Climatic distance	0.48	0.50	0.56	0.61
Interaction	0.51	0.49	0.44	0.39
Spatial distance	0.01	0.00	0.00	0.00
Percentage variance explained	29.92	25.78	22.18	16.74

South America

Cut-off	Full	5 Ma	10 Ma	20 Ma
Correlation with taxonomic turnover	0.64	0.53	0.48	0.36
Correlation with full phylogenetic turnover	1.00	0.98	0.96	0.82
**Variation partitioning**				
Climatic distance	0.55	0.62	0.63	0.64
Interaction	0.40	0.34	0.33	0.30
Spatial distance	0.05	0.04	0.04	0.06
Percentage variance explained	21.84	18.79	17.11	12.05

Africa

Cut-off	Full	5 Ma	10 Ma	20 Ma
Correlation with taxonomic turnover	0.78	0.70	0.63	0.53
Correlation with full phylogenetic turnover	1.00	0.99	0.97	0.90
**Variation partitioning**				
Climatic distance	0.63	0.66	0.69	0.71
Interaction	0.30	0.28	0.26	0.25
Spatial distance	0.06	0.05	0.04	0.03
Percentage variance explained	28.10	27.41	26.62	23.61

Asia

Cut-off	Full	5 Ma	10 Ma	20 Ma
Correlation with taxonomic turnover	0.86	0.77	0.65	0.29
Correlation with full phylogenetic turnover	1.00	0.99	0.94	0.59
**Variation partitioning**				
Climatic distance	0.20	0.26	0.34	1.00
Interaction	0.39	0.35	0.29	0.00
Spatial distance	0.41	0.39	0.37	0.00
Percentage variance explained	34.58	31.87	27.19	15.79

Australia

Cut-off	Full	5 Ma	10 Ma	20 Ma
Correlation with taxonomic turnover	0.77	0.64	0.47	0.12
Correlation with full phylogenetic turnover	1.00	0.96	0.82	0.26
**Variation partitioning**				
Climatic distance	0.47	0.51	0.59	-
Interaction	0.47	0.44	0.38	-
Spatial distance	0.07	0.05	0.03	-
Percentage variance explained	44.67	37.96	25.13	-

Table S13. Ancient phylogenetic turnover variation partitioning results obtained with the genus-level Mimosoid tree per region. The relative contribution of each predictor variable is shown, as well as the total variance explained by the full model.

pantropics

**Cut-off**	**Full**	**5 Ma**	**10 Ma**	**20 Ma**
**Correlation with taxonomic turnover**	0.97	0.96	0.92	0.54
**Correlation with full phylogenetic turnover**	1.00	1.00	0.99	0.69
**Variation partitioning**				
**Climatic distance**	0.41	0.41	0.41	0.54
**Interaction**	0.50	0.50	0.50	0.39
**Spatial distance**	0.08	0.08	0.09	0.07
**Percentage variance explained**	51.98	49.87	46.22	29.58

North America

**Cut-off**	**Full**	**5 Ma**	**10 Ma**	**20 Ma**
**Correlation with taxonomic turnover**	0.88	0.82	0.73	0.54
**Correlation with full phylogenetic turnover**	1.00	0.99	0.96	0.82
**Variation partitioning**				
**Climatic distance**	0.43	0.46	0.50	0.55
**Interaction**	0.53	0.51	0.49	0.45
**Spatial distance**	0.04	0.03	0.02	0.00
**Percentage variance explained**	36.74	33.57	28.74	22.62

South America

**Cut-off**	**Full**	**5 Ma**	**10 Ma**	**20 Ma**
**Correlation with taxonomic turnover**	0.86	0.78	0.60	0.44
**Correlation with full phylogenetic turnover**	1.00	0.99	0.88	0.75
**Variation partitioning**				
**Climatic distance**	0.41	0.48	0.65	0.71
**Interaction**	0.49	0.44	0.30	0.24
**Spatial distance**	0.09	0.08	0.04	0.05
**Percentage variance explained**	31.86	29.72	25.18	18.14

Africa

**Cut-off**	**Full**	**5 Ma**	**10 Ma**	**20 Ma**
**Correlation with taxonomic turnover**	0.91	0.87	0.81	0.57
**Correlation with full phylogenetic turnover**	1.00	1.00	0.98	0.80
**Variation partitioning**				
**Climatic distance**	0.42	0.45	0.50	0.67
**Interaction**	0.46	0.44	0.41	0.29
**Spatial distance**	0.12	0.11	0.09	0.04
**Percentage variance explained**	41.65	41.54	41.34	36.03

Asia

**Cut-off**	**Full**	**5 Ma**	**10 Ma**	**20 Ma**
**Correlation with taxonomic turnover**	0.88	0.83	0.78	0.39
**Correlation with full phylogenetic turnover**	1.00	1.00	0.98	0.62
**Variation partitioning**				
**Climatic distance**	0.09	0.09	0.10	0.46
**Interaction**	0.45	0.45	0.45	0.33
**Spatial distance**	0.47	0.46	0.45	0.21
**Percentage variance explained**	38.63	35.64	32.16	10.93

Australia	Cut-off	Full	5 Ma	10 Ma	20 Ma
	Correlation with taxonomic turnover	0.97	0.95	0.92	0.52
	Correlation with full phylogenetic turnover	1.00	0.95	0.99	0.69
	Variation partitioning				
	Climatic distance	0.34	0.33	0.33	0.41
	Interaction	0.54	0.54	0.54	0.45
	Spatial distance	0.12	0.12	0.13	0.13
	Percentage variance explained	53.17	50.85	46.89	21.73

Table S14. PBD_{dev} variation partitioning results obtained with the metachronogram per region. The relative contribution of each predictor variable is shown, as well as the total variance explained by the full model.

Region	pantropics	North America	South America	Africa	Asia	Australia
Correlation with taxonomic turnover	0.40	0.11	0.16	0.26	-0.03	0.24
Correlation with phylogenetic turnover	0.92	0.74	0.83	0.77	0.43	0.77
Variation partitioning						
Climatic distance	-	0.78	0.78	0.83	0.82	0.66
Interaction	-	0.22	0.20	0.15	0.04	0.30
Spatial distance	-	0.00	0.02	0.03	0.14	0.04
Percentage variance explained	-	12.06	10.07	15.90	6.97	15.97

Table S15. PBD_{dev} variation partitioning results obtained with the genus-level Mimosoid tree per region. The relative contribution of each predictor variable is shown, as well as the total variance explained by the full model.

Region	pantropics	North America	South America	Africa	Asia	Australia
Correlation with taxonomic turnover	0.31	0.10	0.19	0.30	0.01	0.02
Correlation with phylogenetic turnover	0.77	0.54	0.65	0.66	0.45	0.26
Variation partitioning						
Climatic distance	0.13	0.97	0.81	0.67	0.57	0.55
Interaction	0.32	0.03	0.18	0.29	0.29	0.25
Spatial distance	0.55	0.00	0.01	0.03	0.13	0.20
Percentage variance explained	25.32	9.01	9.66	19.60	3.88	3.54

Table S16. Details of the concatenated alignments showing alignment lengths, phylogenetic informativeness, and fractions of missing data.

Alignment	Type	Sites	Alignment patterns	Gaps
All genes with paralogs	NT	1544271	1099898	0.38
All genes with paralogs	AA	514757	429244	0.38
All genes without paralogs	NT	1091682	945847	0.116
All genes without paralogs	AA	363894	348473	0.116
Single-copy genes	NT	944871	824713	0.119
Single-copy genes	AA	314957	302861	0.119
Chloroplast	NT	65409	36662	0.488

Table S17. Robinson-Foulds distances between various species trees. Abbreviations are as follows 'AS' = ASTRAL-3; 'RA' = RAxML; '997' = all genes without paralogs, '997p'= all genes with paralogs, 'SC' = single-copy genes; 'NT' = nucleotide alignment; 'AA' = amino acid alignment.

**Robinson-Foulds distances**	AS-SC	AS-997	AS-997p	RA-SC-NT	RA-997-NT	RA-997p-NT	RA-SC-AA	RA-997-AA	RA-997p-AA	Phy-loBayes
AS-997	14									
AS-997p	10	12								
RA-SC-NT	98	98	100							
RA-997-NT	98	102	100	28						
RA-997p-NT	86	86	88	20	34					
RA-SC-AA	94	92	94	30	50	34				
RA-997-AA	90	90	92	38	44	34	16			
RA-997p-AA	90	90	92	30	46	26	8	12		
PhyloBayes	83	83	83	65	63	63	63	65	63	
Chloroplast	288	290	288	306	306	300	300	302	300	265

Table S18. Number of times taxa were identified as rogue taxa across 54 RogueNaRok analyses.

**Taxon**	**Times identified as rogue**
Zygia inaequalis	12
Zygia longifolia	8
Archidendron quocense	7
Archidendron lucidum	7
Inga cinnamomea	7
Leucochloron limae	6
Zygia ramiflora	5
Paraserianthes lophantha	3
Abarema langsdorfii	2
Hydrochorea corymbosa 1	2
Hydrochorea corymbosa 2	2
Albizia obbiadensis	1
Albizia viridis	1
Archidendron ellipticum subsp. *ellipticum*	1
Archidendron triplinervium	1
Caesalpinia cassioides	1
Hydrochorea obliquifoliolata	1
Hydrochorea rhombifolia	1
Denisophytum madagascariense	1
Hydrochorea gonggrijpii	1
Hydrochorea marginata	1
Inga heterophylla	1
Inga setosa	1
Inga stipularis	1
Samanea saman	1
Wallaceodendron celebicum	1

Table S19. Numbers of accepted taxa, accepted species, and synonyms in the taxonomic checklist, and numbers of records, taxa, and species in the occurrence dataset. See Appendix 1 (Data S1) for details about the assembly and contents of the checklist and occurrence dataset, and Appendix 2 (Data S1) for the full taxonomic checklist. Note that the number of species listed for *Abarema* includes five poorly-known taxa unlikely to belong to *Abarema*; see Appendix 1 for details.

	**Taxonomic checklist**			**Occurrence dataset**		
**Genus**	**Accepted taxa**	**Accepted species**	**Synonyms**	**Occurrences**	**Taxa**	**Species**
Abarema	7	7	13	407	5	5
Acacia	1098	1093	64	178367	1074	1068
Acaciella	25	15	107	4521	23	14
Adenanthera	15	13	13	628	11	10
Adenopodia	7	7	18	364	7	7
Afrocalliandra	2	2	3	51	2	2
Alantsilodendron	9	9	8	145	9	9
Albizia	119	87	213	8706	106	86
Amblygonocarpus	1	1	6	82	1	1
Anadenanthera	6	2	38	4360	6	2
Anonychium	1	1	8	116	1	1
Archidendron	120	99	388	4170	97	87
Archidendropsis	16	11	15	166	16	11
Aubrevillea	2	2	2	112	2	2
Blanchetiodendron	1	1	4	154	1	1
Boliviadendron	1	1	1	3	1	1
Calliandra	206	149	470	16053	188	137
Calliandropsis	1	1	2	68	1	1
Calpocalyx	11	11	10	755	11	11
Cedrelinga	1	1	3	249	1	1
Chidlowia	1	1	0	137	1	1
Chloroleucon	17	11	68	1802	17	11
Cojoba	19	14	191	2527	17	12
Cylicodiscus	1	1	1	90	1	1
Desmanthus	27	23	107	3375	25	23
Dichrostachys	33	13	59	1050	14	13
Ebenopsis	3	3	15	258	3	3
Entada	52	40	127	4415	44	37
Enterolobium	8	8	3	2768	8	8
Faidherbia	1	1	8	624	1	1
Falcataria	3	3	11	433	3	3
Fillaeopsis	1	1	0	84	1	1
Gagnebina	10	8	19	168	10	8
Gretheria	2	2	7	281	2	2
Gwilymia	7	7	12	273	7	7
Havardia	3	3	19	1002	3	3
Heliodendron	3	3	8	323	3	3
Hesperalbizia	1	1	7	465	1	1

Hydrochorea	10	10	48	1476	8	8
Indopiptadenia	1	1	1	25	1	1
Inga	287	275	609	39332	279	266
Jupunba	52	37	164	3246	51	37
Kanaloa	1	1	0	1	1	1
Lachesiodendron	1	1	10	662	1	1
Lemurodendron	1	1	0	5	1	1
Leucaena	33	26	67	2172	25	23
Leucochloron	4	4	7	252	4	4
Lysiloma	8	8	45	5577	8	8
Macrosamanea	14	12	33	642	13	11
Mariosousa	13	13	30	1566	13	13
Marlimorimia	6	6	24	958	5	5
Mezcala	1	1	1	16	1	1
Microlobius	3	1	13	253	2	1
Mimosa	832	589	621	36653	737	530
Mimozyganthus	1	1	1	60	1	1
Naiadendron	1	1	1	31	1	1
Neltuma	68	44	163	5523	51	36
Neptunia	17	11	50	2220	13	10
Newtonia	17	15	24	595	17	15
Osodendron	5	3	37	286	4	3
Painteria	2	2	6	59	2	2
Parapiptadenia	6	6	8	1409	6	6
Pararchidendron	5	1	13	506	4	1
Parasenegalia	11	11	42	268	10	10
Paraserianthes	3	1	17	228	2	1
Parkia	44	38	68	3323	39	33
Pentaclethra	3	3	4	1135	3	3
Piptadenia	32	32	11	2096	25	25
Piptadeniastrum	1	1	1	332	1	1
Piptadeniopsis	1	1	0	12	1	1
Pithecellobium	20	18	309	5346	20	18
Pityrocarpa	9	7	17	1686	8	7
Plathymenia	1	1	5	1517	1	1
Prosopidastrum	5	5	9	80	5	5
Prosopis	7	3	19	49	4	3
Pseudalbizzia	26	17	101	3394	23	17
Pseudoprosopis	9	7	9	353	8	7
Pseudosamanea	3	3	13	424	3	3
Pseudosenegalia	2	2	4	11	2	2
Punjuba	6	6	16	184	5	5
Ricoa	1	1	9	80	1	1
Robrichia	3	3	6	557	3	3
Samanea	3	3	20	839	3	3

Sanjappa	1	1	1	1	1	1
Schleinitzia	4	4	13	71	3	3
Senegalia	256	221	480	20636	214	190
Serianthes	27	18	19	168	21	17
Sphinga	3	3	15	563	3	3
Strombocarpa	12	10	31	722	11	9
Stryphnodendron	31	29	29	2178	20	18
Sympetalandra	5	5	4	74	5	5
Tetrapleura	2	2	2	314	2	2
Thailentadopsis	3	3	24	23	3	3
Vachellia	233	169	556	24434	200	146
Viguieranthus	24	18	11	334	19	18
Wallaceodendron	1	1	4	33	1	1
Xerocladia	1	1	2	34	1	1
Xylia	11	9	13	488	10	9
Zapoteca	35	23	116	4031	31	20
Zygia	66	58	384	5238	61	56
**Total**	4135	3464	6408	424333	3746	3232

Table S20. Phylogenetic turnover variation partitioning results obtained with the metachronogram per region. The relative contribution of each predictor variable is shown, as well as the total variance explained by the full model.

	**pantropics**	**North America**	**South America**	**Africa**	**Asia**	**Australia**
**Climatic distance**	0.04	0.48	0.55	0.63	0.20	0.47
**Interaction**	0.31	0.51	0.40	0.30	0.39	0.47
**Spatial distance**	0.64	0.01	0.05	0.06	0.41	0.07
**Percentage variance explained**	54.47	29.92	21.84	28.10	34.58	44.67

**Precipitation**	0.75	0.85	0.80	1.00	0.59	0.47
**Interaction**	-0.01	0.11	0.08	0.00	-0.00	0.14
**Temperature**	0.26	0.04	0.12	0.00	0.41	0.39
**Percentage variance explained**	3.00	9.10	6.69	15.00	2.31	16.20

**Precipitation**	0.60	0.83	0.61	0.93	0.04	0.51
**Interaction**	0.04	0.08	0.31	0.07	0.17	0.19
**Precipitation seasonality**	0.36	0.09	0.08	0.00	0.79	0.29
**Percentage variance explained**	3.48	9.58	6.41	15.02	6.37	13.93

**Precipitation**	0.05	0.28	0.38	0.40	0.00	0.19
**Interaction**	0.73	0.66	0.60	0.56	0.23	0.73
**Dry season length**	0.22	0.06	0.02	0.04	0.77	0.08
**Percentage variance explained**	2.84	9.31	6.04	15.66	5.96	10.68

**Precipitation**	0.77	0.90	0.91	0.81	0.40	0.49
**Interaction**	-0.07	-0.04	-0.04	0.16	0.02	0.20
**SD of cloud cover**	0.29	0.14	0.14	0.03	0.58	0.31
**Percentage variance explained**	3.14	10.13	6.84	15.51	3.24	14.19

Table S21. Phylogenetic turnover variation partitioning results obtained with the genus-level Mimosoid tree per region. The relative contribution of each predictor variable is shown, as well as the total variance explained by the full model.

	pantropics	North America	South America	Africa	Asia	Australia
**Climatic distance**	0.11	0.43	0.41	0.42	0.09	0.41
**Interaction**	0.33	0.53	0.49	0.46	0.45	0.50
**Spatial distance**	0.56	0.04	0.09	0.12	0.47	0.08
**Percentage variance explained**	51.98	36.74	31.86	41.65	38.63	53.41

**Precipitation**	0.72	0.83	0.75	0.99	0.61	0.54
**Interaction**	0.01	0.07	0.07	-0.00	0.08	0.16
**Temperature**	0.27	0.09	0.19	0.01	0.31	0.30
**Percentage variance explained**	5.45	10.88	7.03	18.13	1.57	16.90

**Precipitation**	0.64	0.89	0.77	0.92	0.06	0.41
**Interaction**	0.04	0.06	0.18	0.07	0.23	0.27
**Precipitation seasonality**	0.33	0.05	0.05	0.00	0.71	0.32
**Percentage variance explained**	5.89	10.34	6.06	18.07	3.76	17.51

**Precipitation**	0.13	0.32	0.33	0.44	-0.00	0.05
**Interaction**	0.80	0.54	0.62	0.56	0.26	0.78
**Dry season length**	0.07	0.14	0.05	0.00	0.74	0.17
**Percentage variance explained**	4.25	11.50	6.02	18.00	4.10	14.32

**Precipitation**	0.84	0.89	0.93	0.73	0.39	0.38
**Interaction**	-0.04	-0.05	-0.04	0.21	-0.02	0.29
**SD of cloud cover**	0.20	0.16	0.11	0.06	0.63	0.34
**Percentage variance explained**	4.94	11.70	6.43	19.14	2.91	17.87

Table S22. The influence of biomes on phylogenetic turnover, tested using variation partitioning with the metachronogram per region. Three biome classification schemes are used (see methods for details). The relative contribution of each predictor variable is shown, as well as the total variance explained by the full model.

	**pantropics**	**North America**	**South America**	**Africa**	**Asia**	**Australia**
**Olson et al. (*98*)**						
**Climatic/spatial distance**	0.92	0.86	0.81	0.79	0.84	0.66
**Interaction**	0.08	0.14	0.18	0.20	0.13	0.34
**Biomes**	0.00	0.00	0.01	0.01	0.03	0.01
**Percentage variance explained**	53.47	31.07	23.84	29.08	38.51	42.34
**Higgins et al. (*99*)**						
**Climatic/spatial distance**	0.89	0.96	0.84	0.95	0.94	0.74
**Interaction**	0.11	0.04	0.15	0.05	0.06	0.25
**Biomes**	0.00	0.00	0.00	0.00	0.00	0.01
**Percentage variance explained**	53.25	31.84	23.50	28.92	35.31	42.32
**Rainforest, savanna, succulent**						
**Climatic/spatial distance**	0.94	0.66	0.63	0.68	0.94	0.99
**Interaction**	0.94	0.34	0.33	0.32	0.05	0.01
**Biomes**	0.05	0.00	0.03	0.00	0.01	0.00

Table S23. The influence of biomes on phylogenetic turnover, tested using variation partitioning with the genus-level Mimosoid tree per region. Three biome classification schemes are used (see methods for details). The relative contribution of each predictor variable is shown, as well as the total variance explained by the full model.

	**pantropics**	**North America**	**South America**	**Africa**	**Asia**	**Australia**
**Olson et al. (*98*)**						
**Climatic/spatial distance**	0.91	0.84	0.87	0.77	0.76	0.64
**Interaction**	0.09	0.16	0.12	0.22	0.16	0.35
**Biomes**	0.00	0.00	0.01	0.01	0.08	0.00
**Percentage variance explained**	51.89	38.31	34.32	42.37	42.85	52.31
**Higgins et al. (*99*)**						
**Climatic/spatial distance**	0.88	0.90	0.90	0.92	0.93	0.71
**Interaction**	0.12	0.09	0.10	0.08	0.07	0.28
**Biomes**	0.00	0.00	0.00	0.00	0.00	0.01
**Percentage variance explained**	51.73	38.95	33.38	42.01	40.14	52.76
**Rainforest, savanna, succulent**						
**Climatic/spatial distance**	0.96	0.72	0.66	0.63	0.92	1.00
**Interaction**	0.96	0.28	0.31	0.36	0.07	0.00
**Biomes**	0.04	0.00	0.03	0.01	0.01	0.00
**Percentage variance explained**	51.64	38.84	34.41	42.40	40.38	52.32

Table S24. Fractions of trans-oceanic dispersal events per splitting event in the phylogeny through time, measured using six different models and two definitions of trans-oceanic dispersal, as well as the log likelihood and AIC weight of each model.

Time period	DEC+j_7_regions	DEC+j_3_regions	DEC_7_regions	DEC_3_regions	BAY_7_regions	BAY_3_regions
0-5	0.02	0.00	0.02	0.00	0.02	0.00
5-10	0.02	0.01	0.02	0.01	0.02	0.01
10-15	0.03	0.02	0.04	0.02	0.03	0.02
15-20	0.03	0.02	0.03	0.02	0.02	0.02
20-25	0.09	0.06	0.09	0.05	0.09	0.06
25-30	0.05	0.03	0.05	0.05	0.03	0.00
30-35	0.09	0.05	0.14	0.09	0.05	0.05
35-40	0.00	0.00	0.14	0.14	0.00	0.00
**LnL**	-1594.07	-1594.07	-1613.45	-1613.45	-2272.26	-2272.26
**AICc_weight**	0.9999999896	0.9999999896	0.0000000104	0.0000000104	0.0	0.0

Time period	BAY+j_7_regions	BAY+j_3_regions	DIVA_7_re-gions	DIVA_3_re-gions	DIVA+j_7_re-gions	DIVA+j_3_re-gions	AIC weighted average
0-5	0.02	0.00	0.02	0.00	0.02	0.00	0.01
5-10	0.02	0.01	0.02	0.01	0.02	0.01	0.02
10-15	0.03	0.02	0.04	0.03	0.03	0.02	0.03
15-20	0.02	0.02	0.03	0.03	0.03	0.02	0.02
20-25	0.09	0.06	0.11	0.06	0.11	0.07	0.07
25-30	0.03	0.00	0.11	0.05	0.08	0.03	0.04
30-35	0.05	0.05	0.14	0.09	0.09	0.05	0.07
35-40	0.00	0.00	0.00	0.00	0.00	0.00	0.00
**LnL**	-2243.43	-2243.43	-1680.34	-1680.34	-1651.30	-1651.30	
**AICc_weight**	0.0	0.0	0.0	0.0	0.0	0.0	

5. Supplementary figures

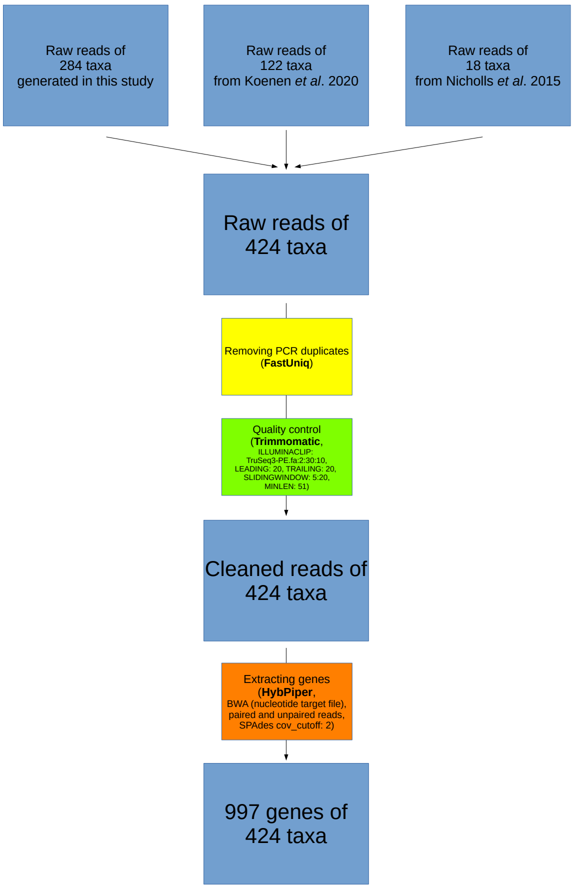

Figure S1. Overview of the data cleaning and target assembly workflow.

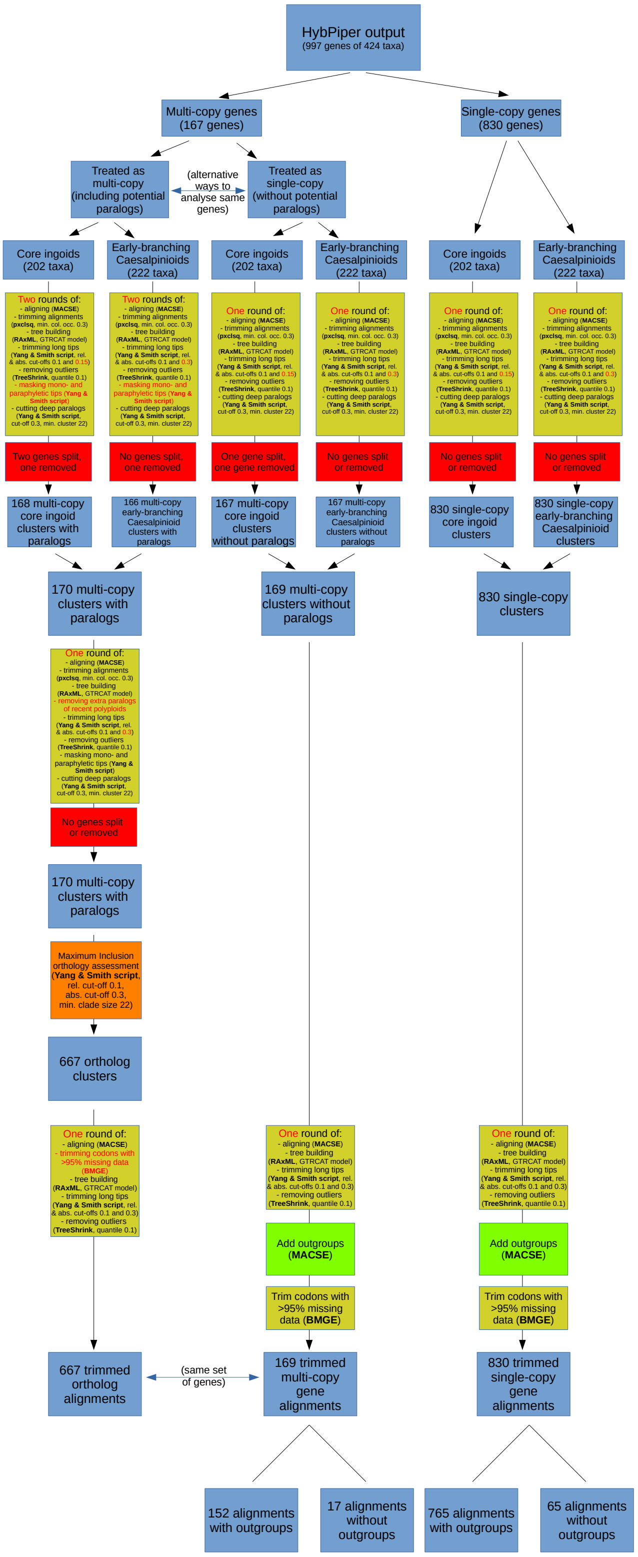

Figure S2. Overview and results of the orthology assessment workflow.

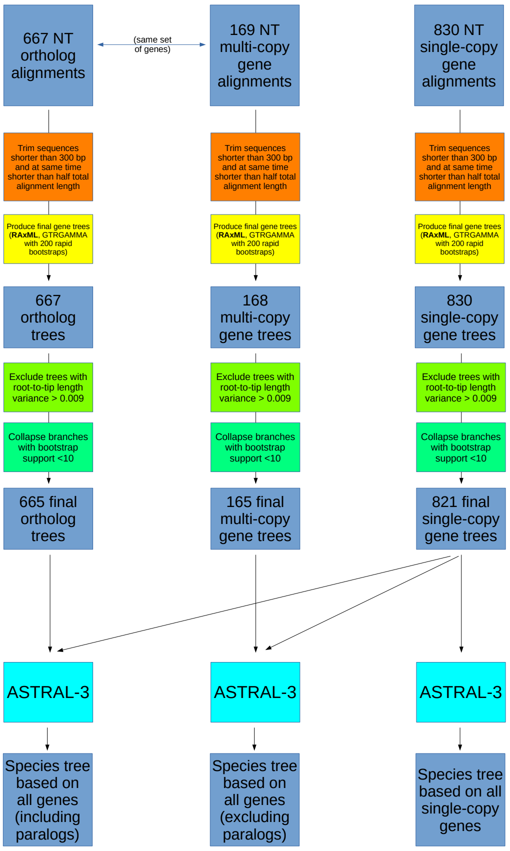

Figure S3. Overview of species tree inference workflow with ASTRAL-3.

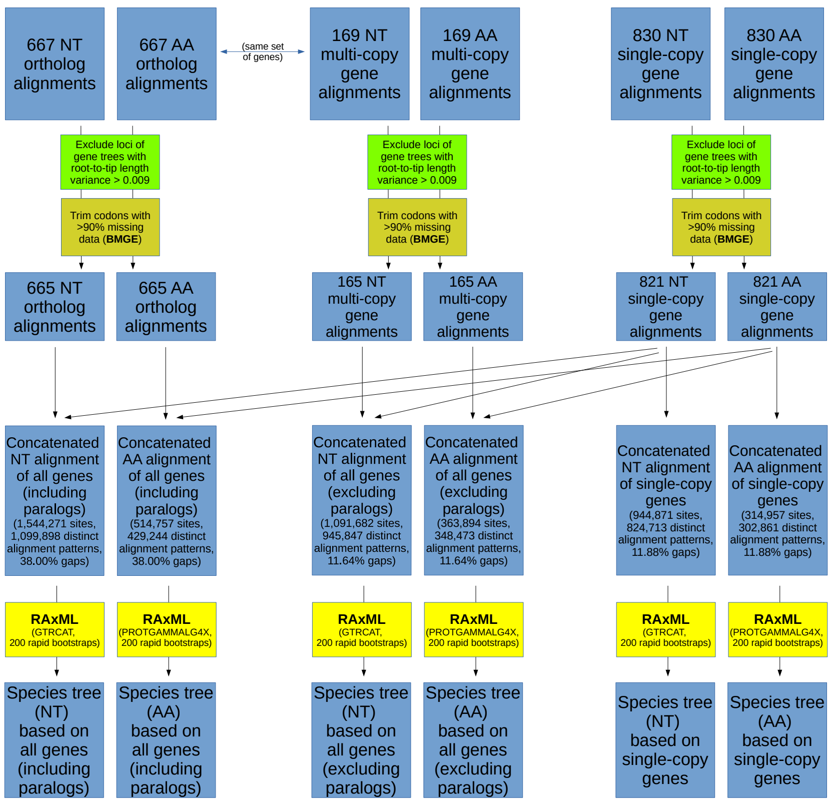

Figure S4. Overview of species tree inference workflow with RAxML.

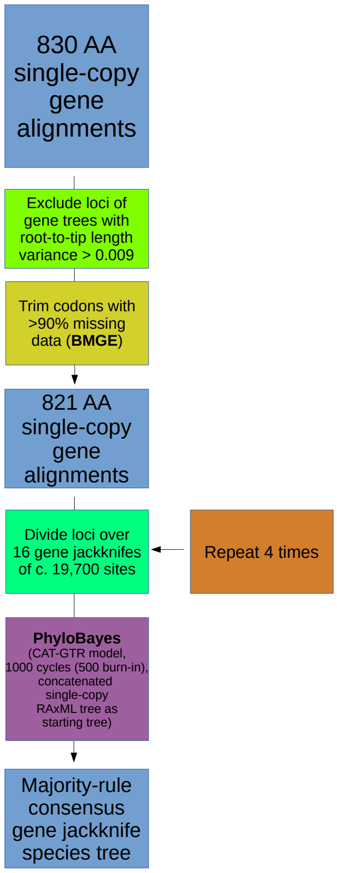

Figure S5. Overview of species tree inference workflow with PhyloBayes.

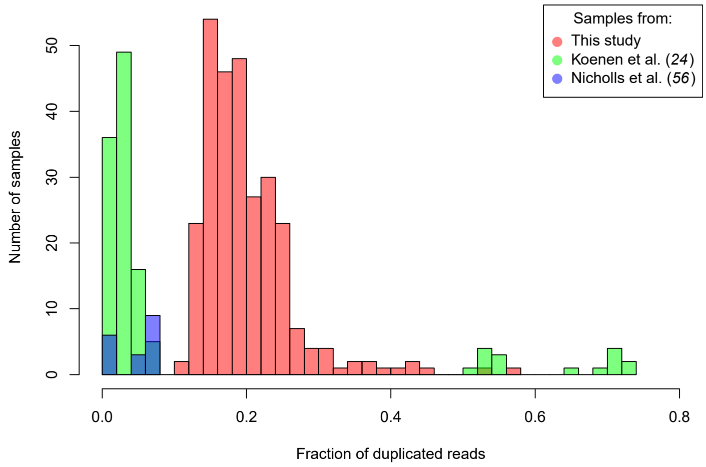

Figure S6 (left). Fractions of duplicated reads per sample.

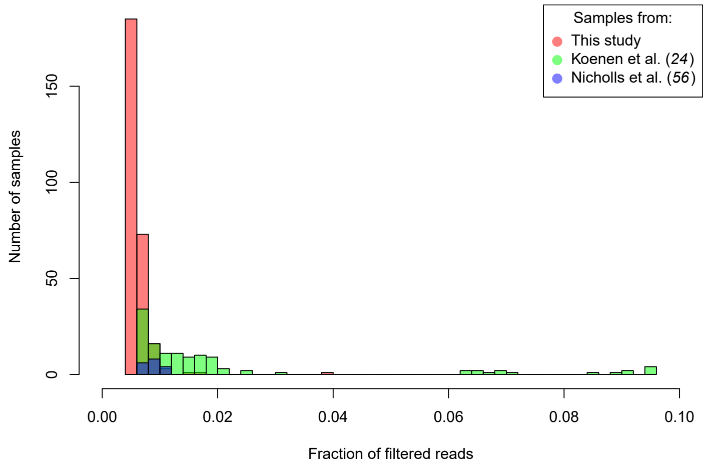

Figure S7 (left). Fractions of filtered reads per sample.

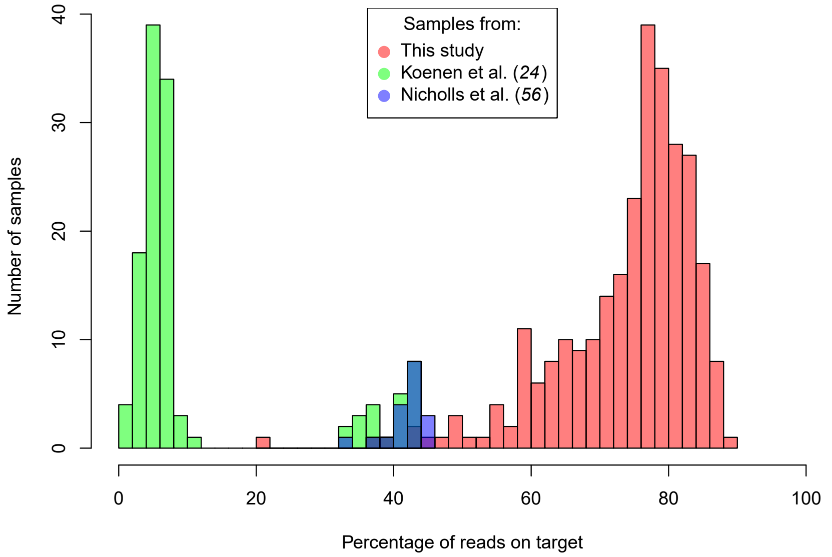

Figure S8 (left). Percentages of reads on target per sample.

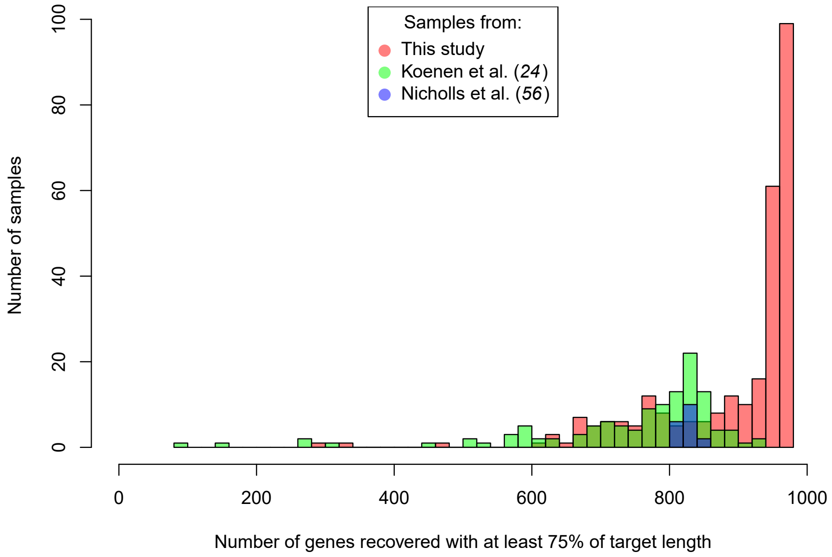

Figure S9 (left). Numbers of genes recovered with at least 75% of the target length per sample.

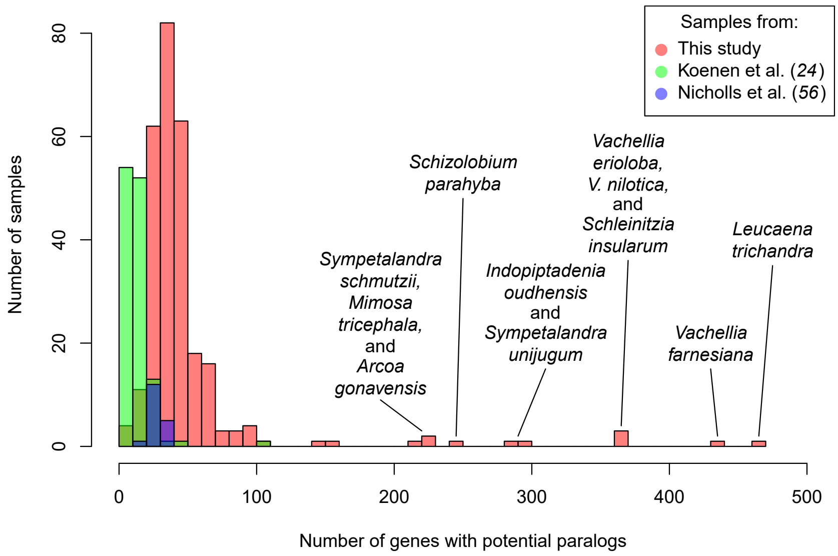

Figure S10 (left). Numbers of genes with potential paralogs per sample. The eleven samples with the most potential paralogs are labelled.

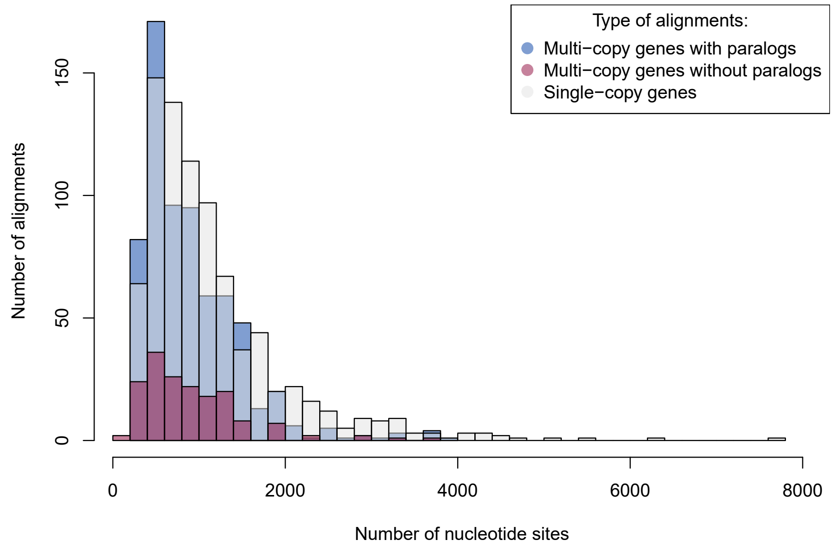

Figure S11 (left). Numbers of nucleotide sites per alignment.

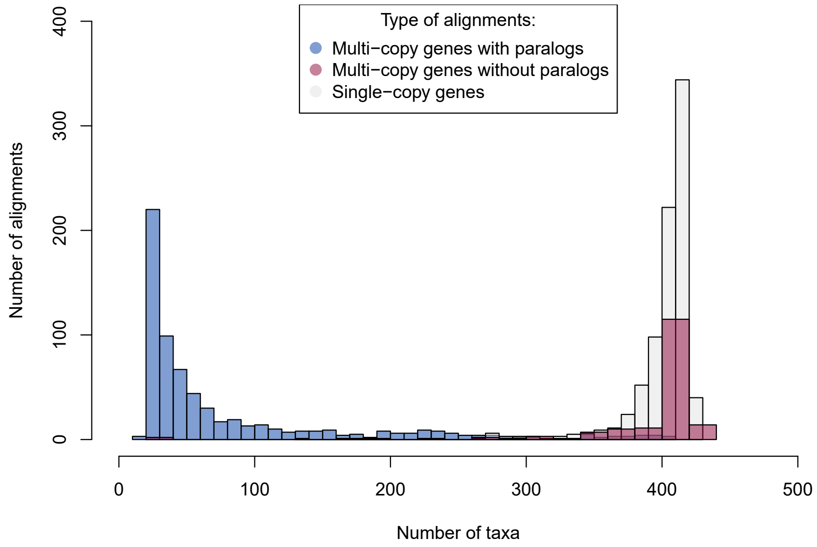

Figure S12 (left). Numbers of taxa per alignment.

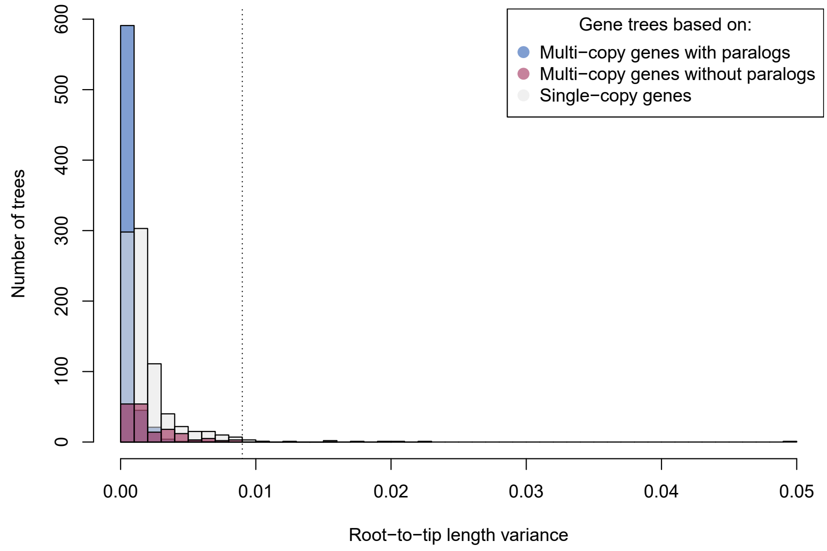

Figure S13 (left). Root-to-tip length variances per gene tree. The cut-off of 0.009 is indicated with a dashed vertical line.

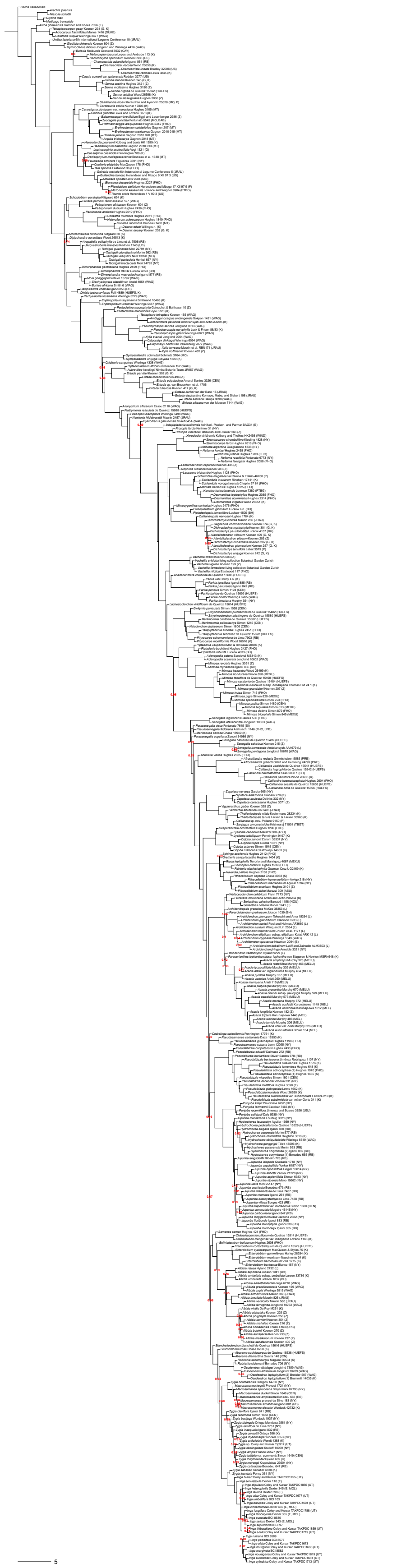

Figure S14. Phylogeny of Caesalpinioideae. ASTRAL species tree based on the 821 single-copy gene trees. Local posterior probability support values are only shown for nodes with a local posterior probability < 1. Branch lengths are expressed in coalescent units. Terminal branches were assigned an arbitrary uniform length for visual clarity.

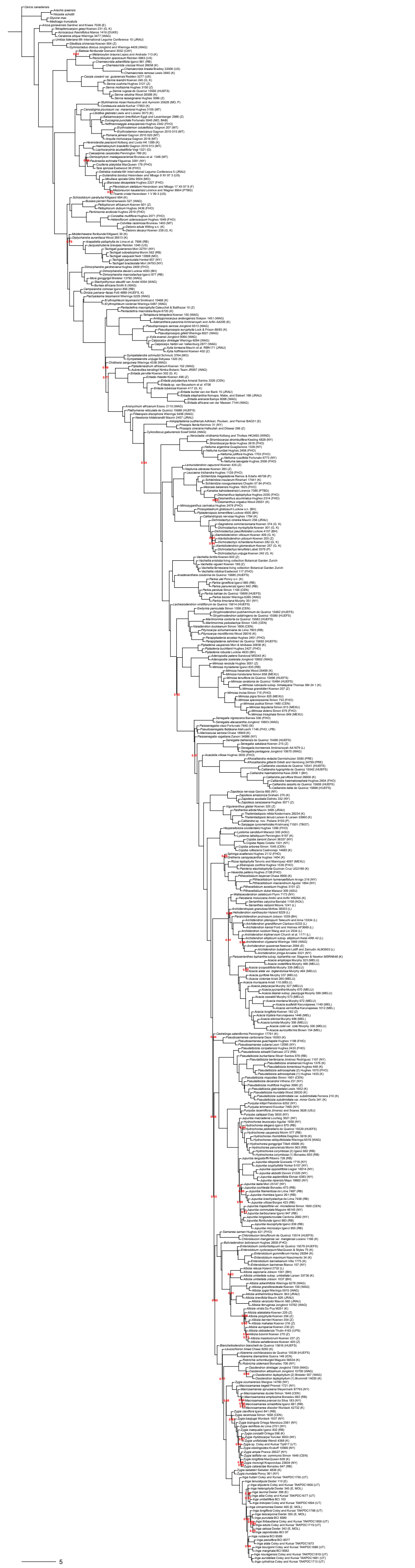

Figure S15. Phylogeny of Caesalpinioideae. ASTRAL species tree based on the 821 single-copy gene trees and the 165 multi-copy gene trees without orthology assessment. Local posterior probability support values are only shown for nodes with a local posterior probability < 1. Branch lengths are expressed in coalescent units. Terminal branches were assigned an arbitrary uniform length for visual clarity.

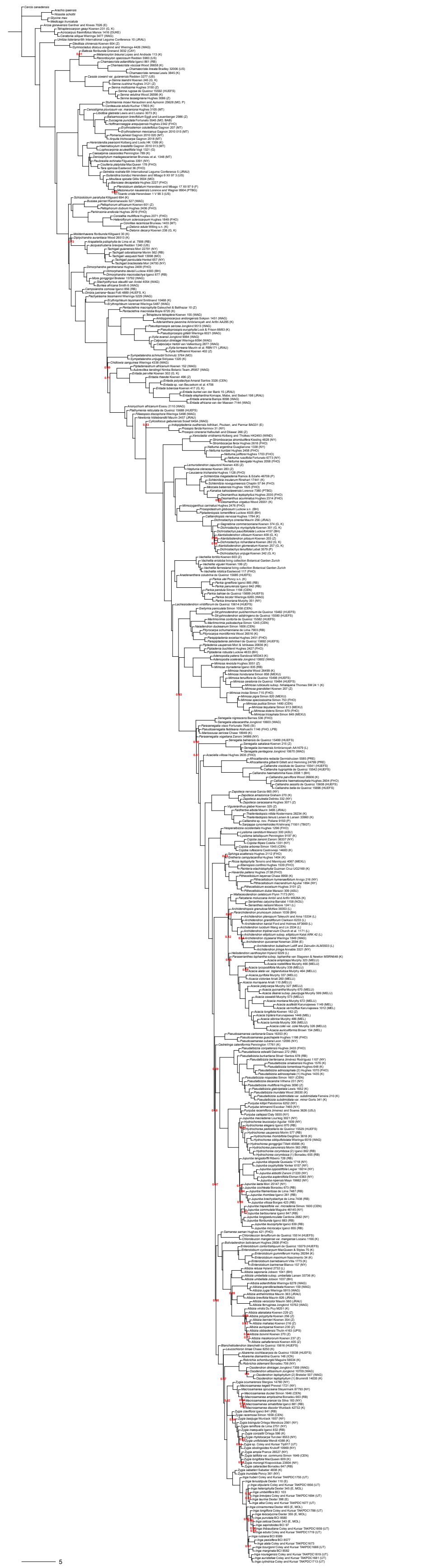

Figure S16. Phylogeny of Caesalpinioideae. ASTRAL species tree based on the 821 single-copy gene trees and the 665 ortholog trees resulting from orthology assessment. Local posterior probability support values are only shown for nodes with a local posterior probability < 1. Branch lengths are expressed in coalescent units. Terminal branches were assigned an arbitrary uniform length for visual clarity.

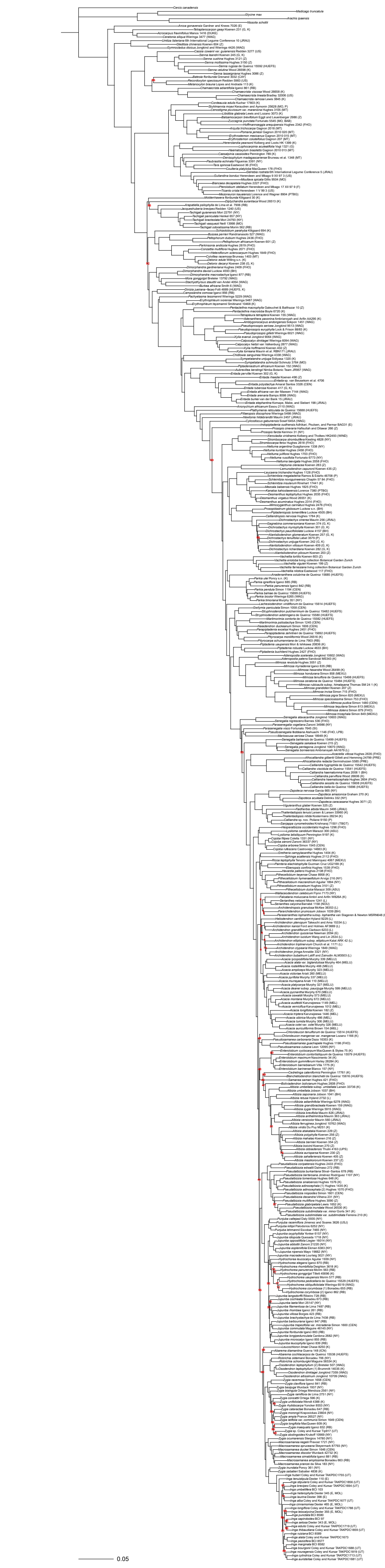

Figure S17. Phylogeny of Caesalpinioideae. RAxML species tree based on the nucleotide single-copy genes alignment. Bootstrap support values are only shown for nodes with < 100% bootstrap support.

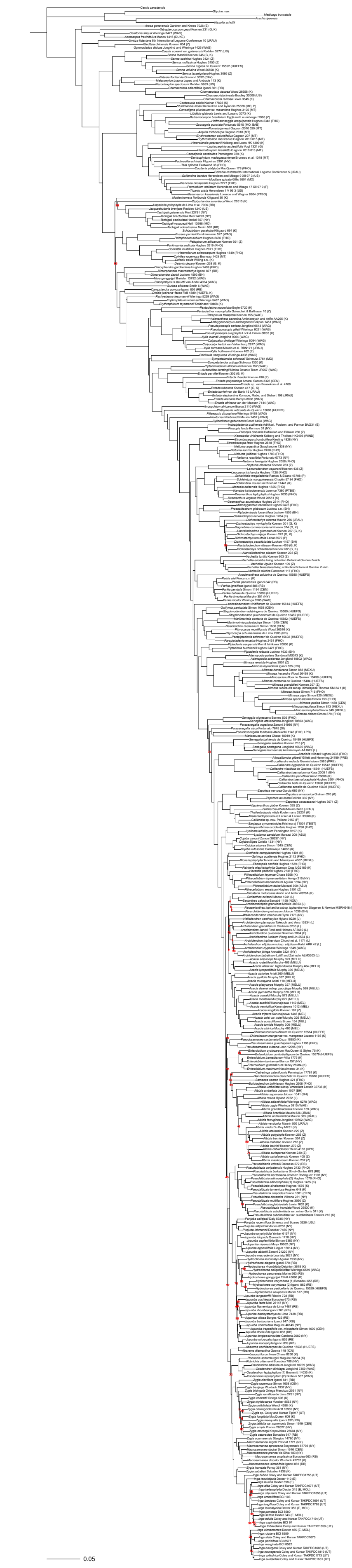

Figure S18. Phylogeny of Caesalpinioideae. RAxML species tree based on the nucleotide alignment of all genes without orthology assessment. Bootstrap support values are only shown for nodes with < 100% bootstrap support.

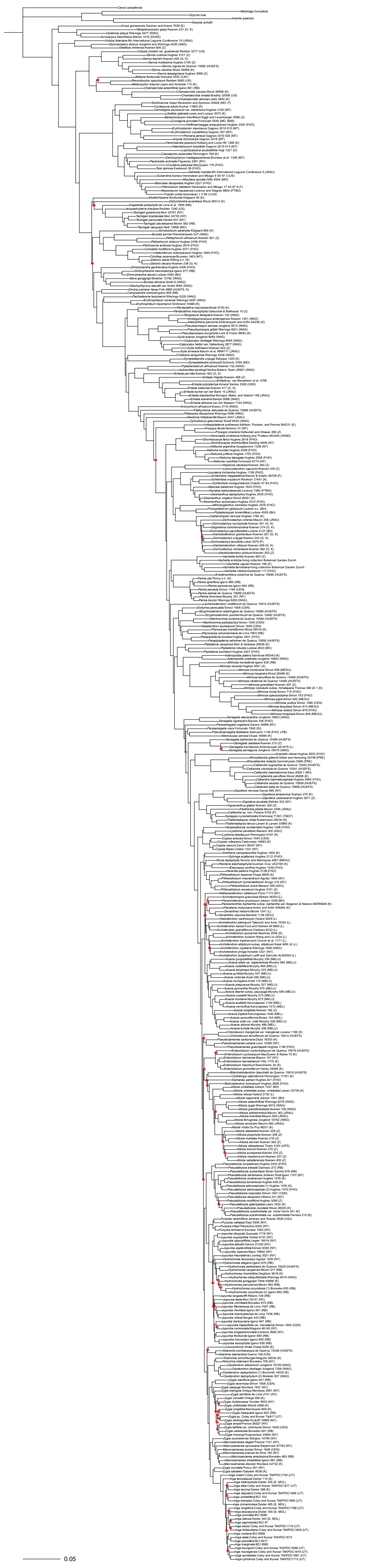

Figure S19. Phylogeny of Caesalpinioideae. RAxML species tree based on the nucleotide alignment of all genes with orthology assessment. Bootstrap support values are only shown for nodes with < 100% bootstrap support.

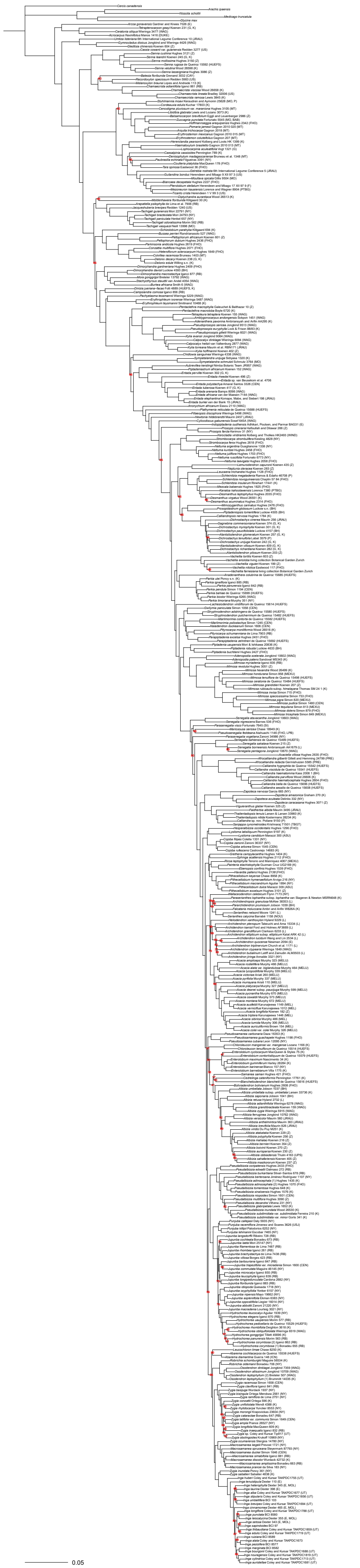

Figure S20. Phylogeny of Caesalpinioideae. RAxML species tree based on the amino acid single-copy genes alignment. Bootstrap support values are only shown for nodes with < 100% bootstrap support.

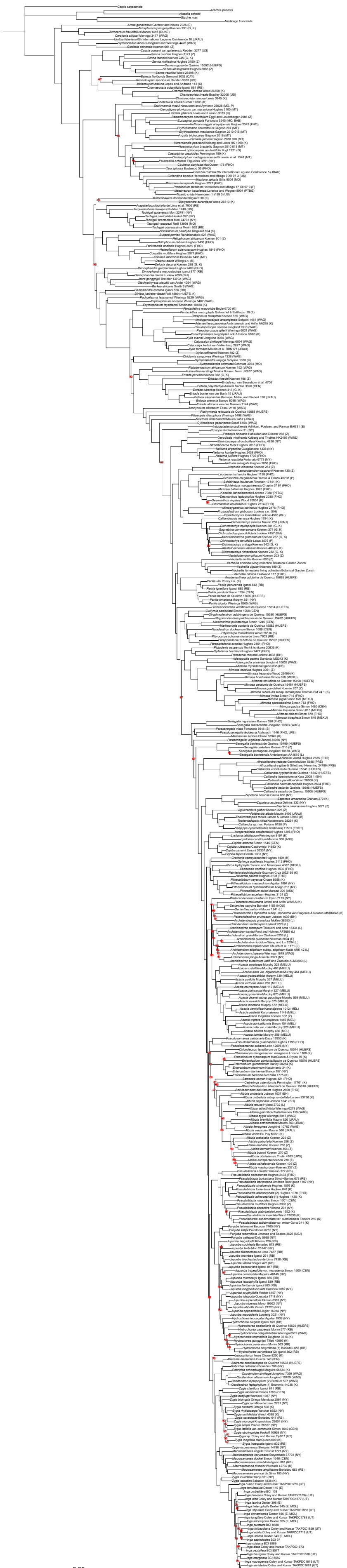

Figure S21. Phylogeny of Caesalpinioideae. RAxML species tree based on the amino acid alignment of all genes without orthology assessment. Bootstrap support values are only shown for nodes with < 100% bootstrap support.

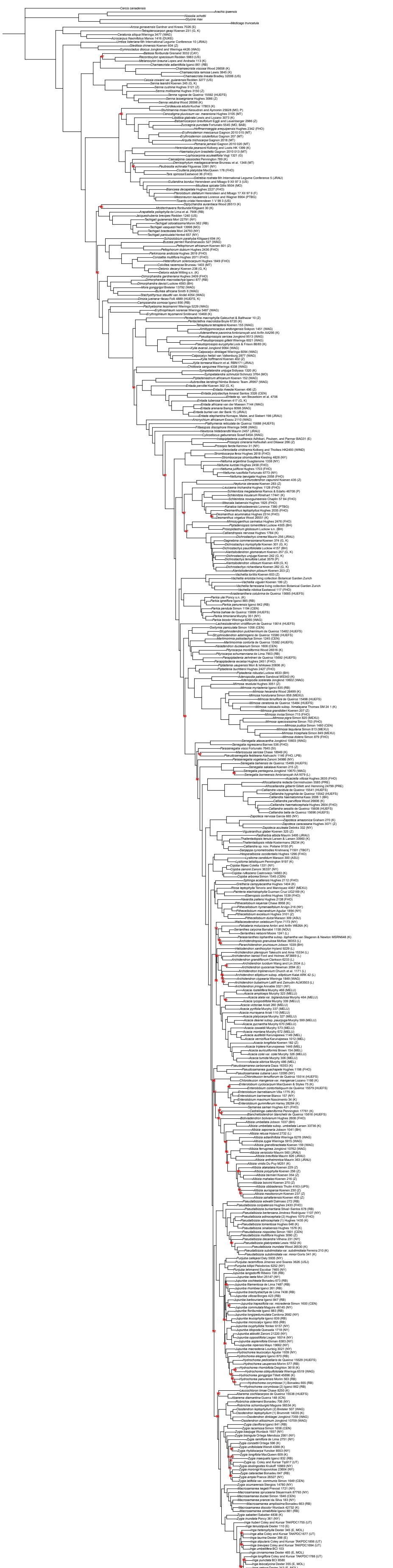

Figure S22. Phylogeny of Caesalpinioideae. RAxML species tree based on the amino acid alignment of all genes with orthology assessment. Bootstrap support values are only shown for nodes with < 100% bootstrap support.

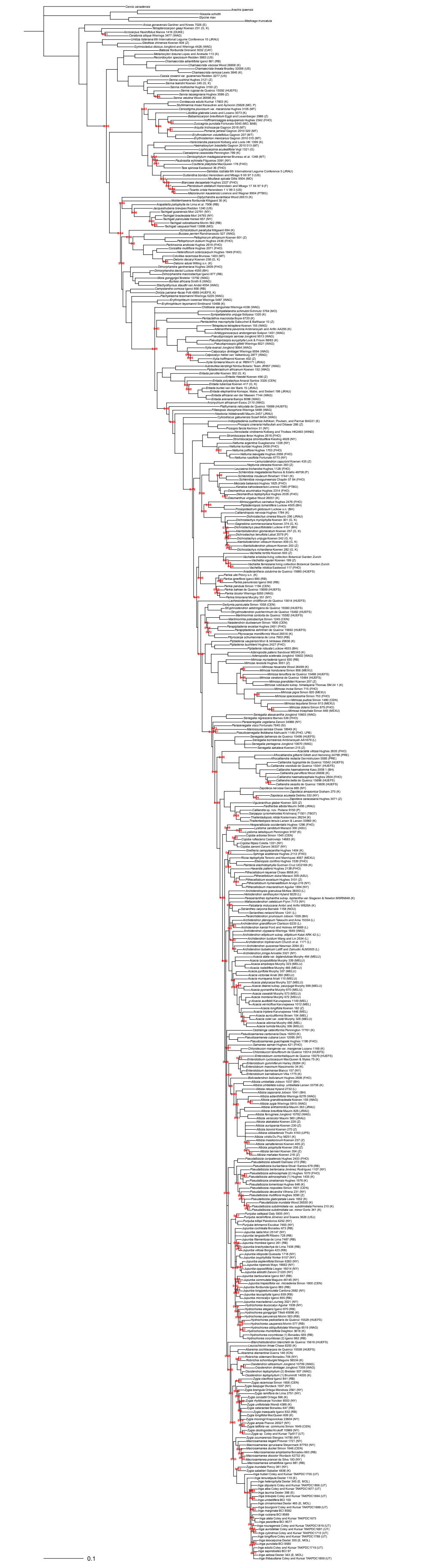

Figure S23. Phylogeny of Caesalpinioideae. PhyloBayes species tree. Posterior probability support values are only shown for nodes with a posterior probability < 1.

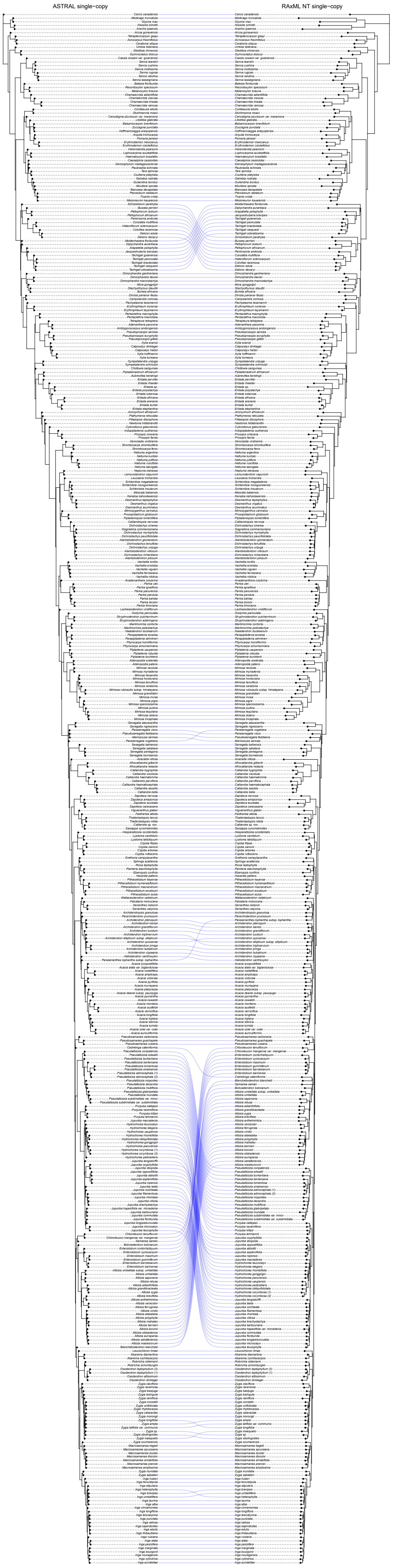

Figure S24. Tanglegram comparing the ASTRAL single-copy genes phylogeny (Figure S14) with the RAxML nucleotide single-copy genes phylogeny (Figure S17).

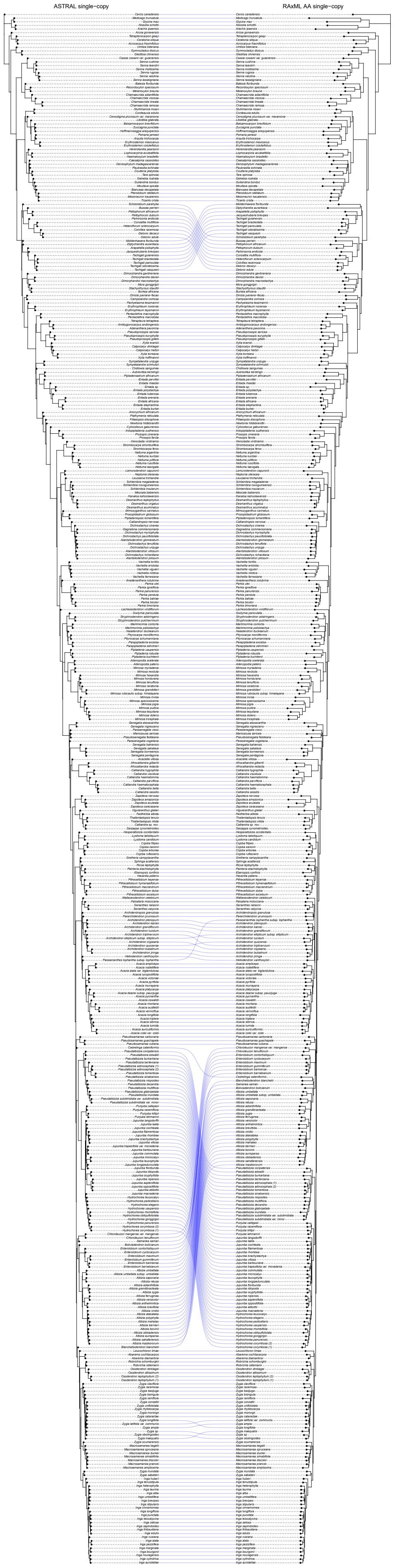

Figure S25. Tanglegram comparing the ASTRAL single-copy genes phylogeny (Figure S14) with the RAxML amino acid single-copy genes phylogeny (Figure S20).

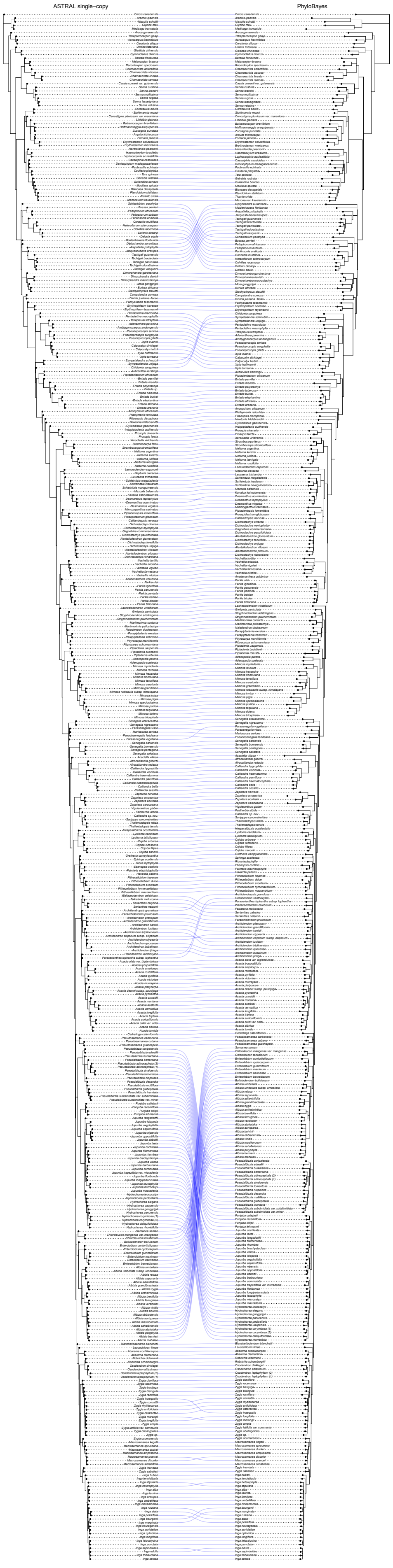

Figure S26. Tanglegram comparing the ASTRAL single-copy genes phylogeny (Figure S14) with the PhyloBayes phylogeny (Figure S23).

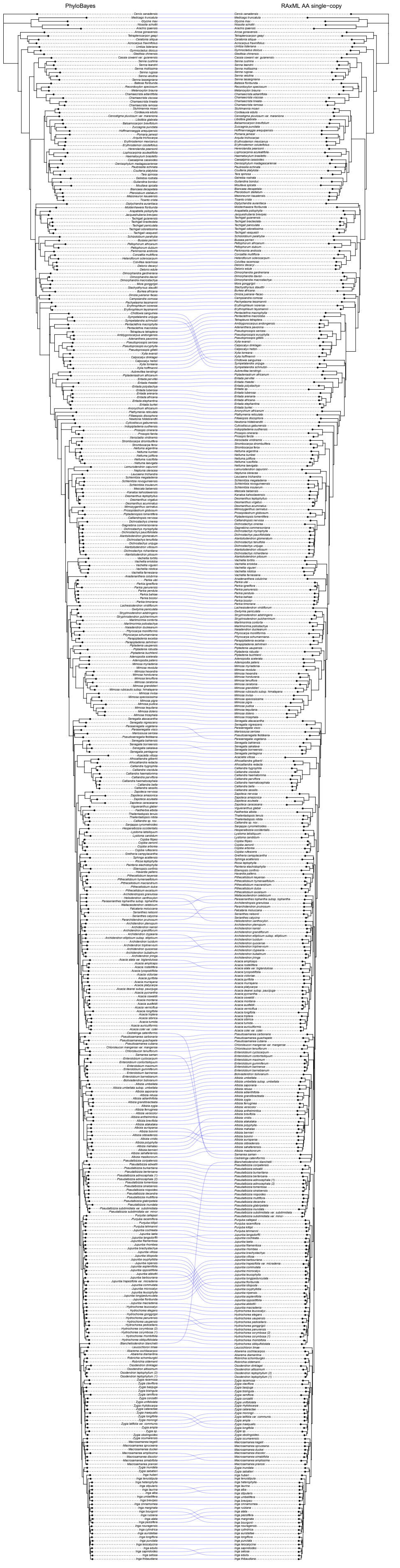

Figure S27. Tanglegram comparing the PhyloBayes phylogeny (Figure S23) with the RAxML amino acid single-copy genes phylogeny (Figure S20).

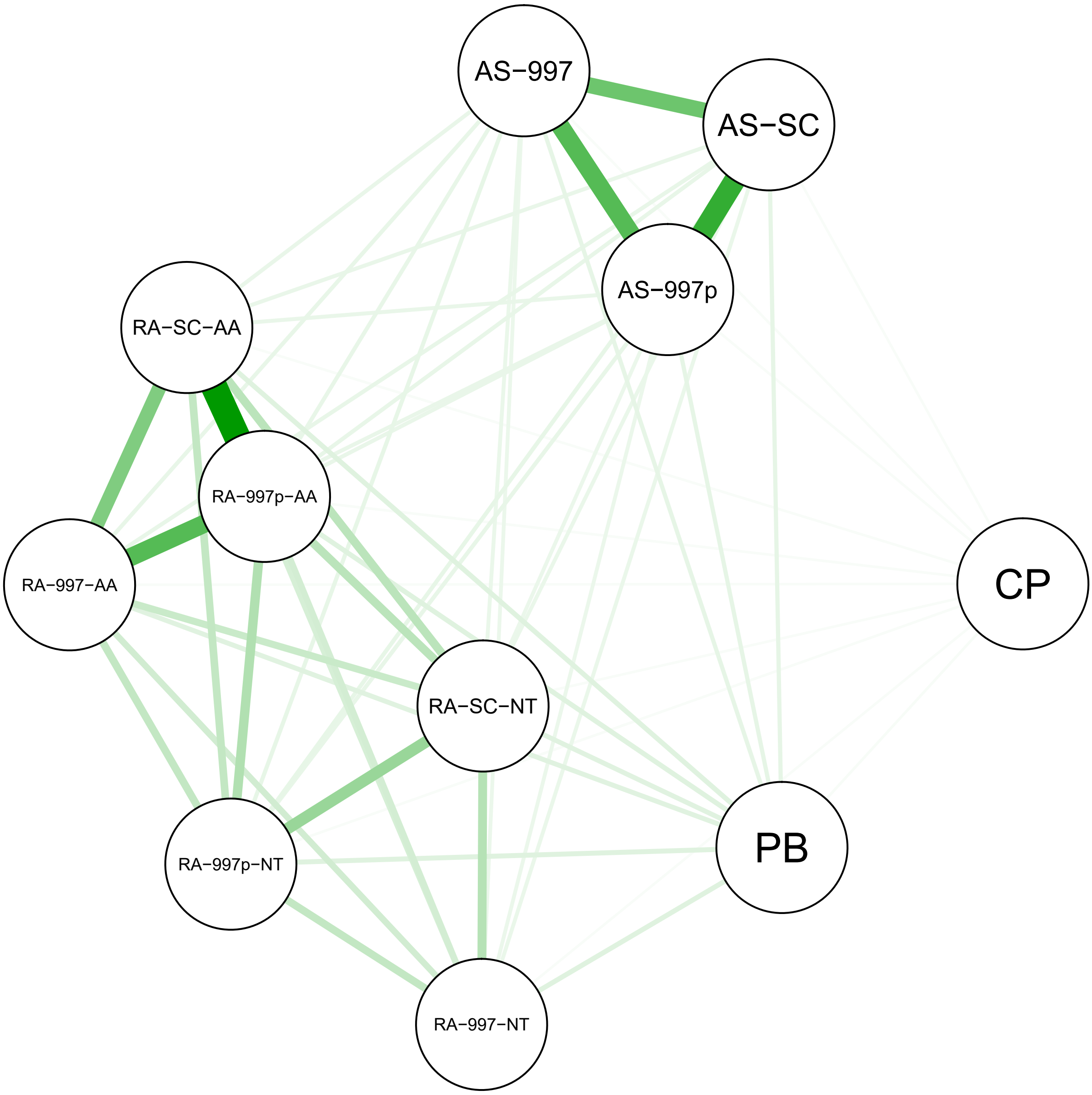

Figure S28. Levels of topological congruence between phylogenies generated in different ways, estimated as Robinson-Foulds (RF) distances between species trees. Exact values are in Table S17. Thickness of the connecting lines reflects RF distance. Abbreviations are as follows 'AS' = ASTRAL-3; 'RA' = RAxML; '997' = all genes without paralogs, '997p'= all genes with paralogs, 'SC' = single-copy genes; 'NT' = nucleotide alignment; 'AA' = amino acid alignment; 'PB' = PhyloBayes phylogeny; 'CP' = chloroplast phylogeny.

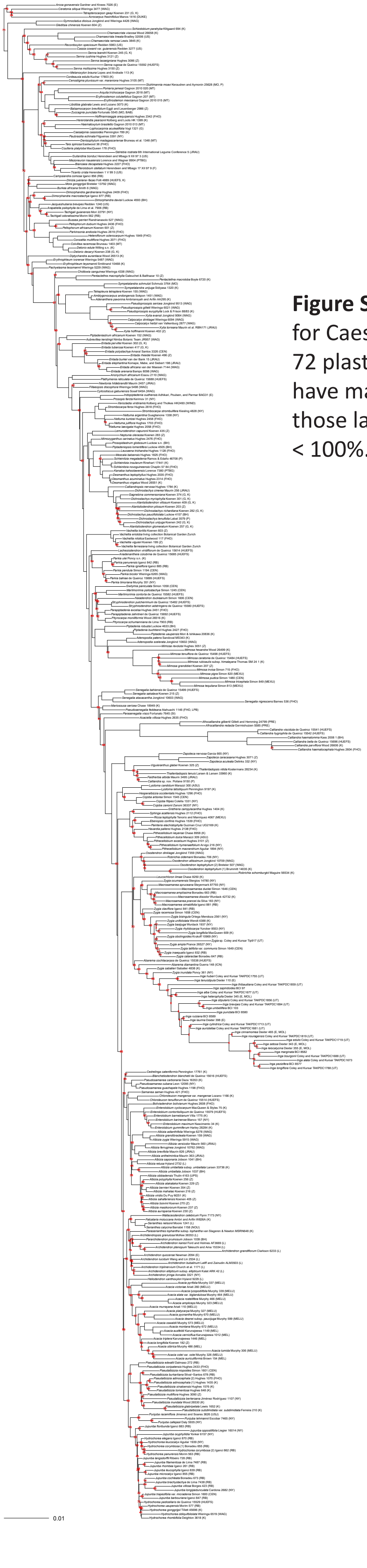

Figure S29 (left). Chloroplast gene tree for Caesalpinioideae based on analysis of 72 plastid genes using RAxML. All nodes have maximal bootstrap support except those labelled with actual support values < 100%.

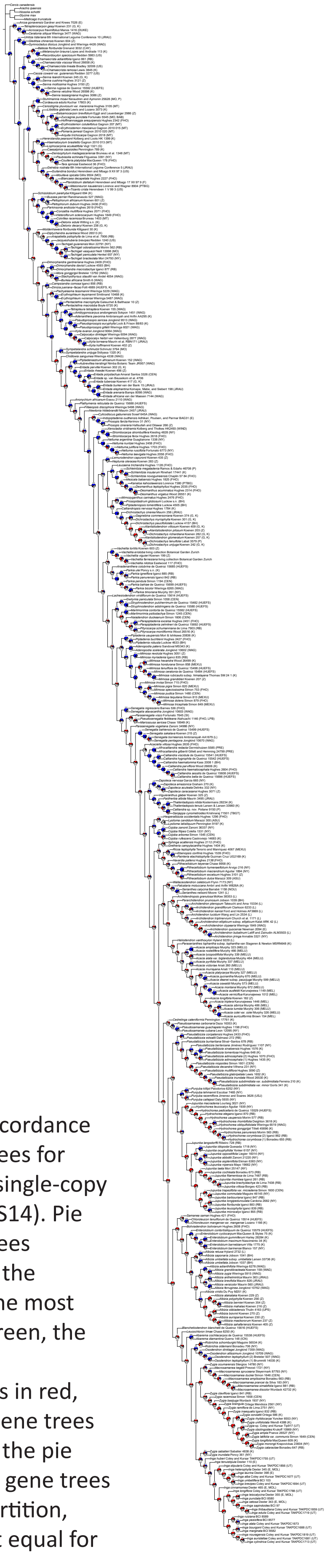

Figure S30 (right). Conflict and concordance among the 821 single-copy gene trees for each bipartition mapped onto the single-copy genes ASTRAL species tree (Figure S14). Pie charts show the fraction of gene trees supporting that bipartition in blue, the fraction of gene trees supporting the most likely alternative configuration in green, the fraction of gene trees supporting additional conflicting configurations in red, and the fraction of uninformative gene trees in grey. Numbers above and below the pie charts indicate the total number of gene trees supporting and conflicting the bipartition, respectively. Branch lengths are set equal for easier visualisation.

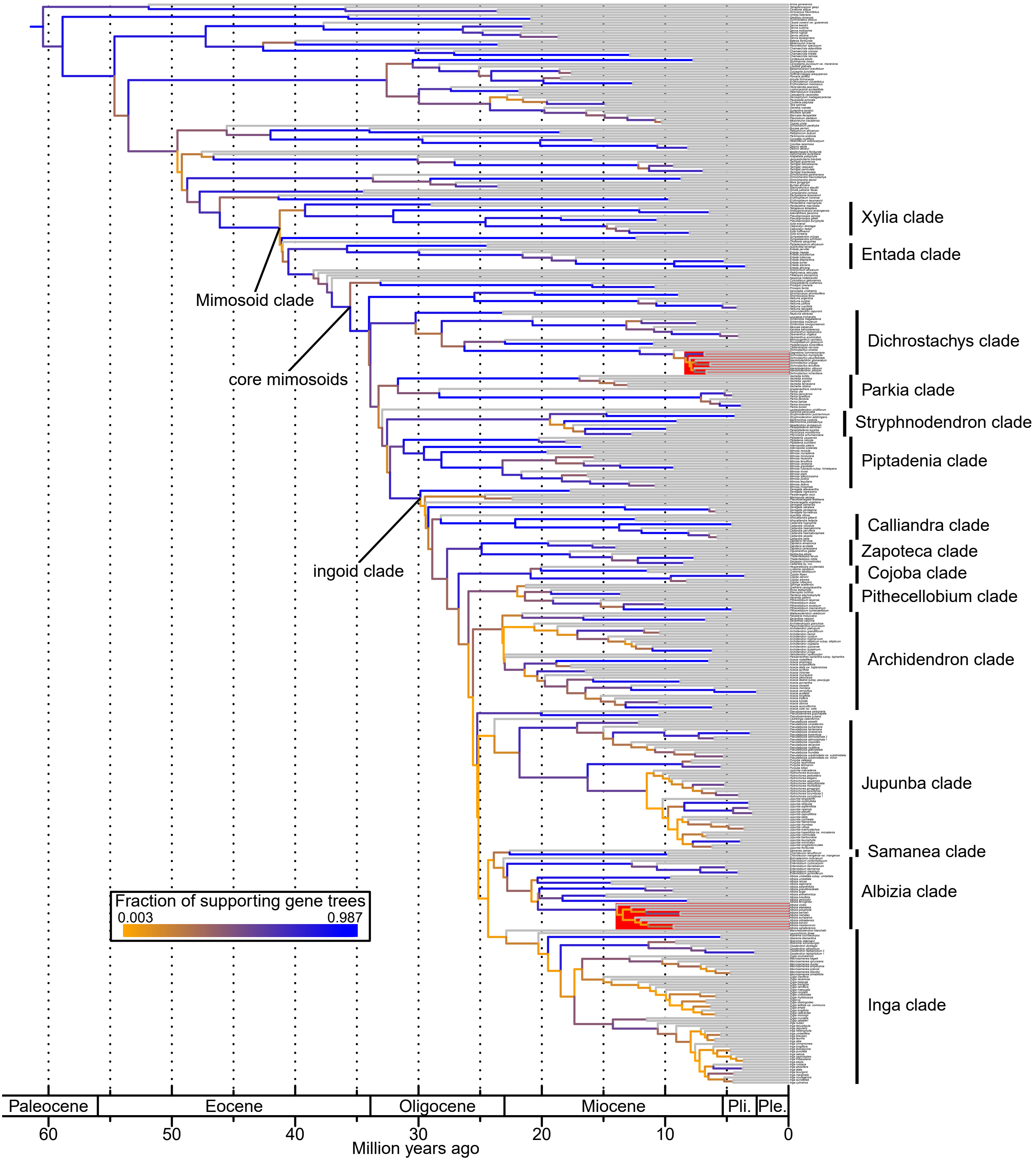

Figure S31. Gene tree incongruence mapped onto the time-calibrated version of the phylogenomic backbone of Caesalpinioideae. Each branch is coloured to reflect the ratio of total supporting versus total conflicting gene trees as determined by PhyParts. Clades named by Koenen et al. (*24*) are labelled. Two recent radiations in Madagascar, one in the Dichrostachys clade and one in *Albizia*, are highlighted.

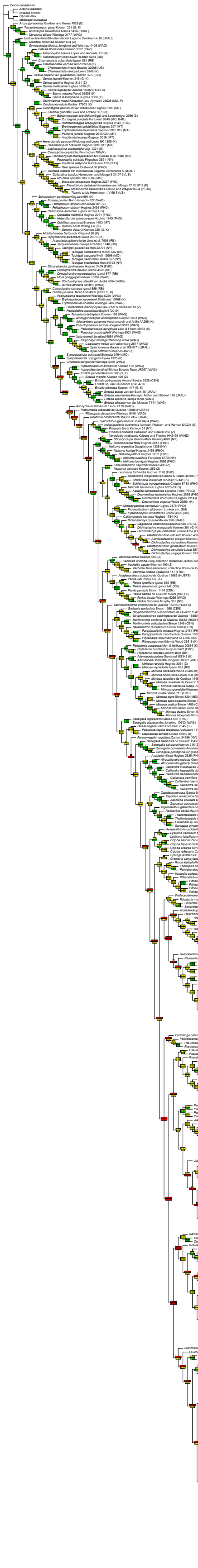

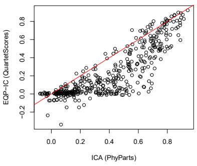

Figure S32 (left). Internode certainty values based on the 821 single-copy gene trees mapped onto the single-copy genes ASTRAL species tree (Figure S14). For each node, the upper number shows the quartet-based Extended Quadripartition Internode Certainty (EQP-IC) score calculated with QuartetScores, and the lower number shows the bipartition-based Internode Certainty All score calculated with PhyParts, both rounded down to two digits. Boxes are coloured based on unrounded values: green for values ≥ 0.5, yellow for values ≥ 0 and < 0.5, and red for values < 0. Branch lengths are set equal for easier visualisation. Inset depicts a correlation plot between the two measures.

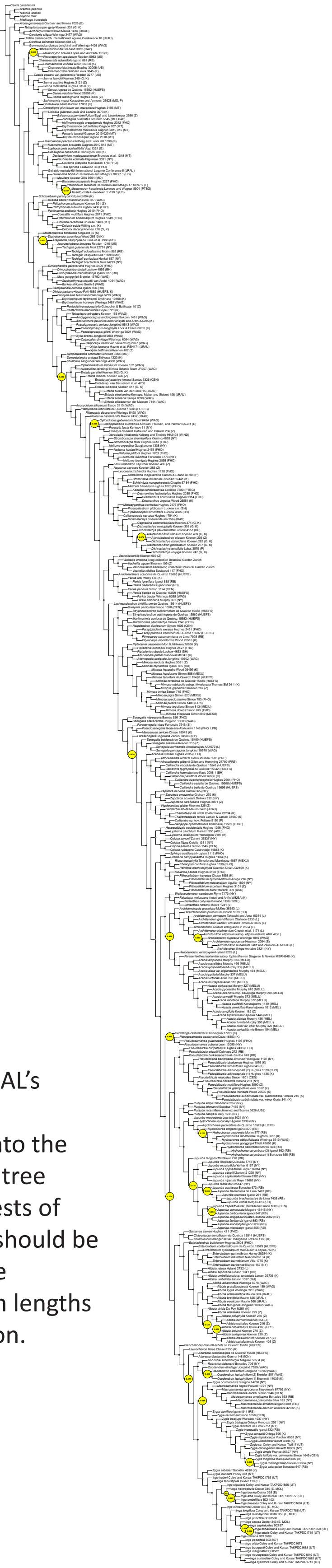

Figure S33 (right). Results of ASTRAL's polytomy test based on the 821 single-copy gene trees mapped onto the single-copy genes ASTRAL species tree (Figure S14). Node numbers are tests of the null hypothesis that a branch should be replaced by a polytomy. Only node numbers > 0.05 are shown. Branch lengths are set equal for easier visualisation.

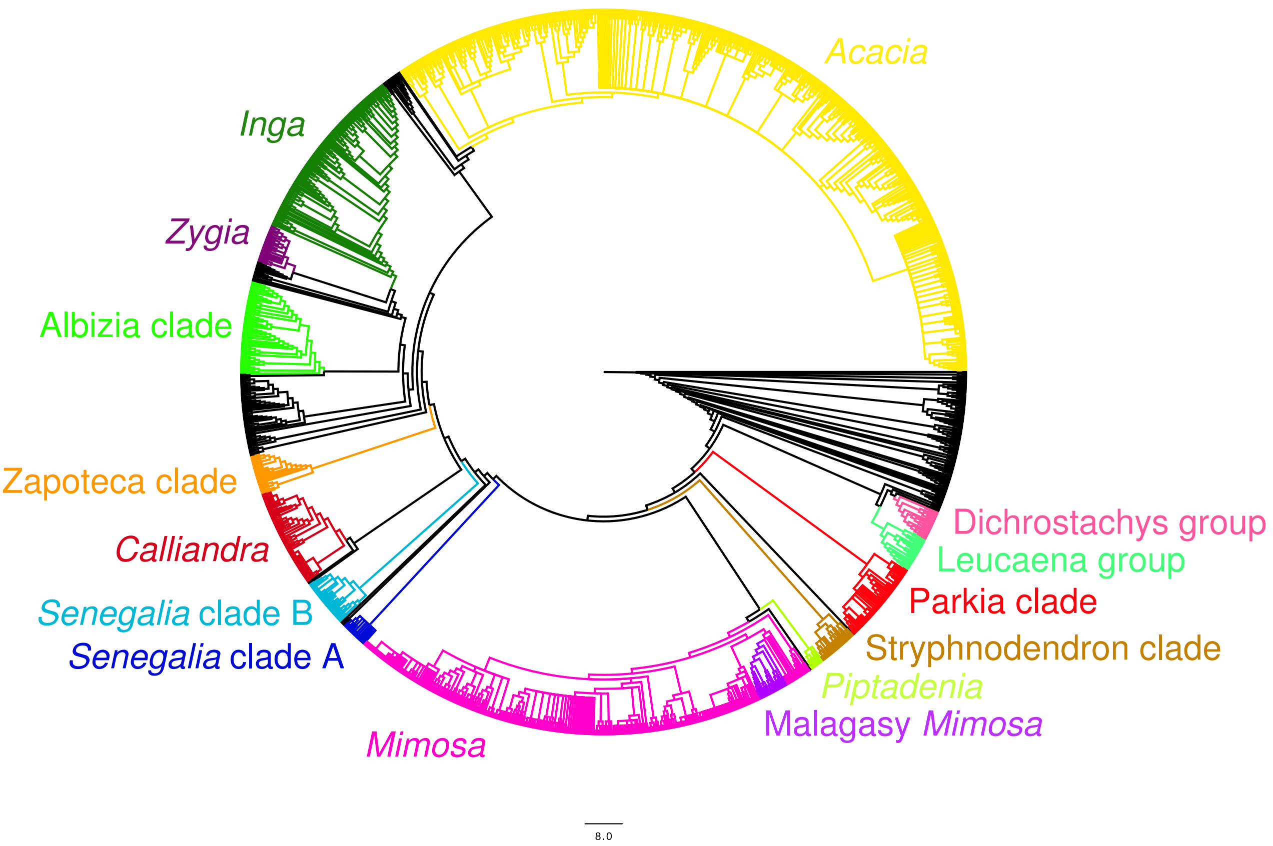

Figure S34. Metachronogram with the names and locations of all subtrees (coloured branches) that were grafted onto the phylogenomic backbone (black branches).

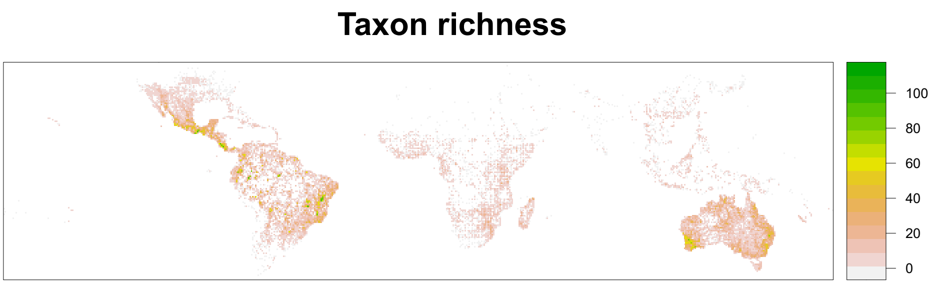

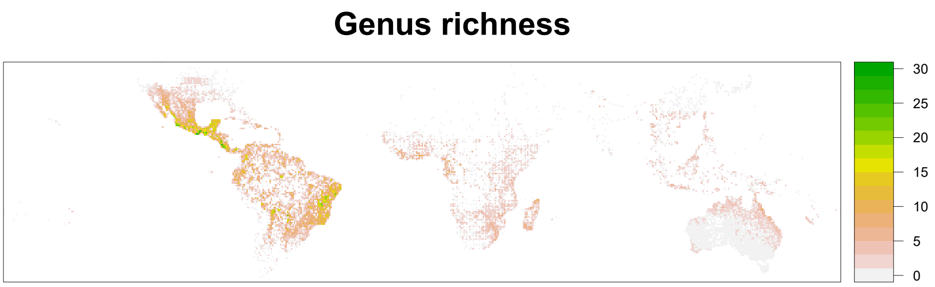

Figure S35. Mimosoid taxon and genus richness per half degree latitude/longitude grid cell.

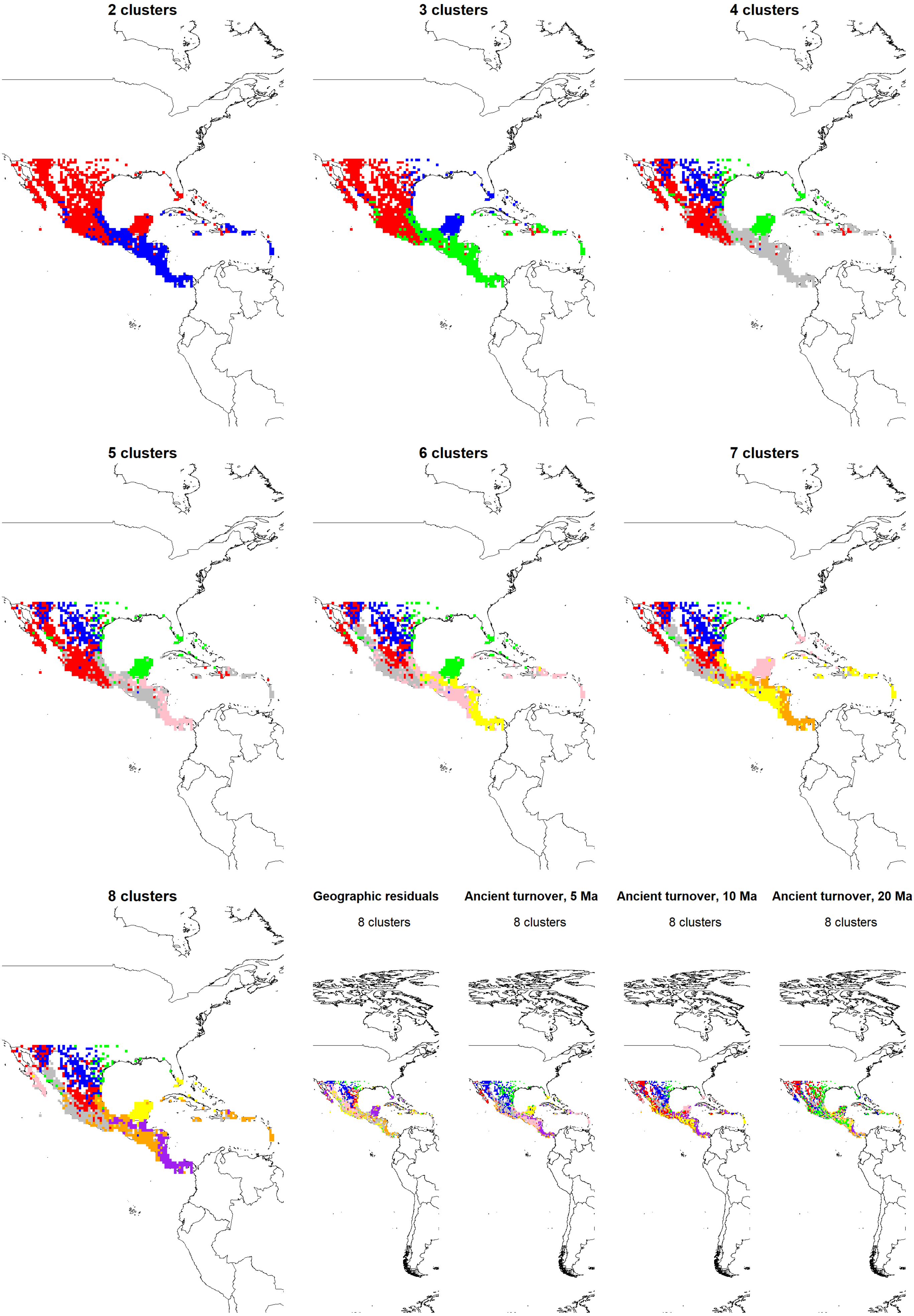

Figure S36. Phyloregionalization of North America using the metachronogram. Subfigures show clustering results with two to eight phyloregions, as well as the results of phyloregionalization analyses using the geographic residuals of phylogenetic turnover, and ancient phylogenetic turnover with a cut-off of 5, 10, and 20 million years.

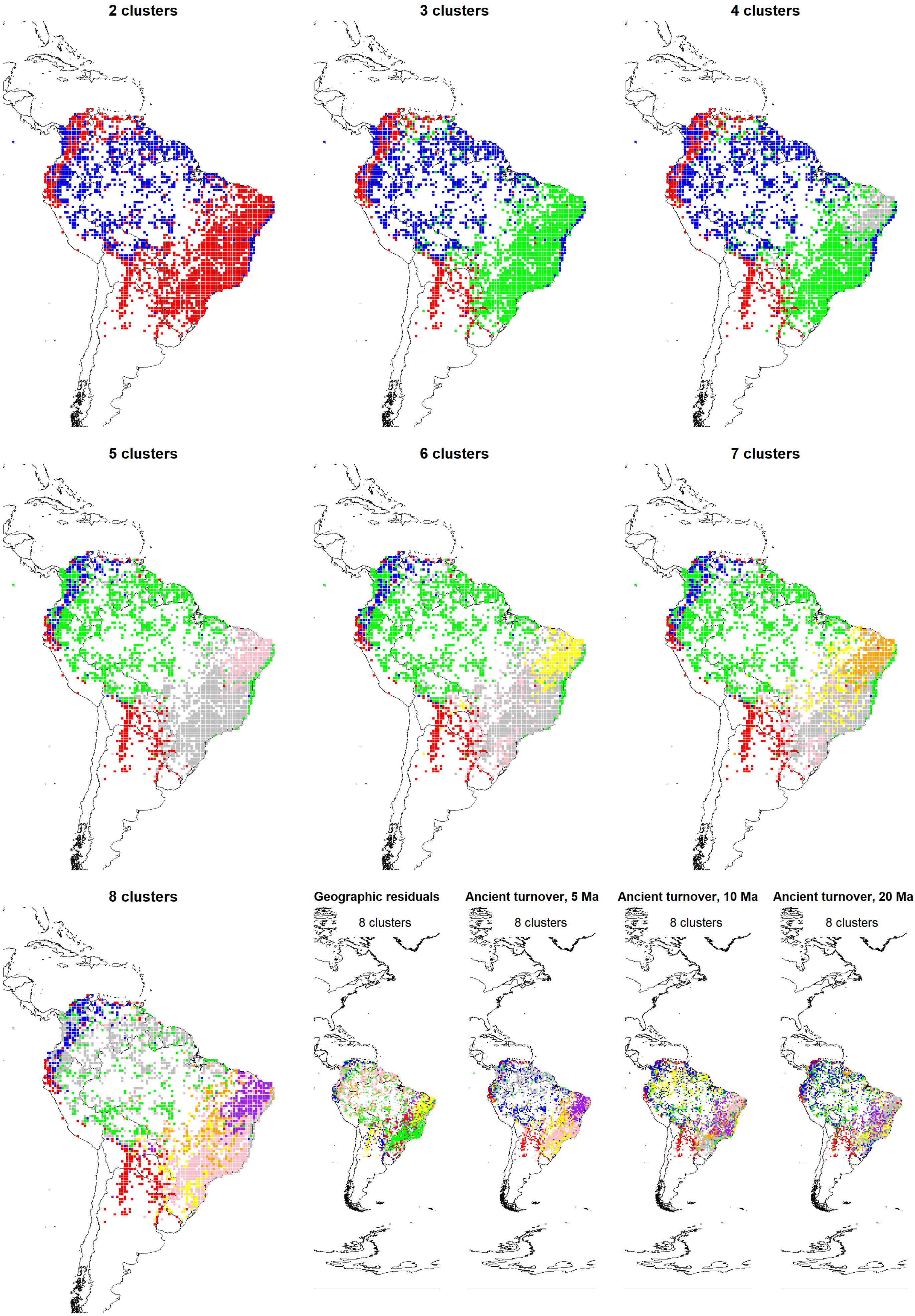

Figure S37. Phyloregionalization of South America using the metachronogram. Subfigures show clustering results with two to eight phyloregions, as well as the results of phyloregionalization analyses using the geographic residuals of phylogenetic turnover, and ancient phylogenetic turnover with a cut-off of 5, 10, and 20 million years.

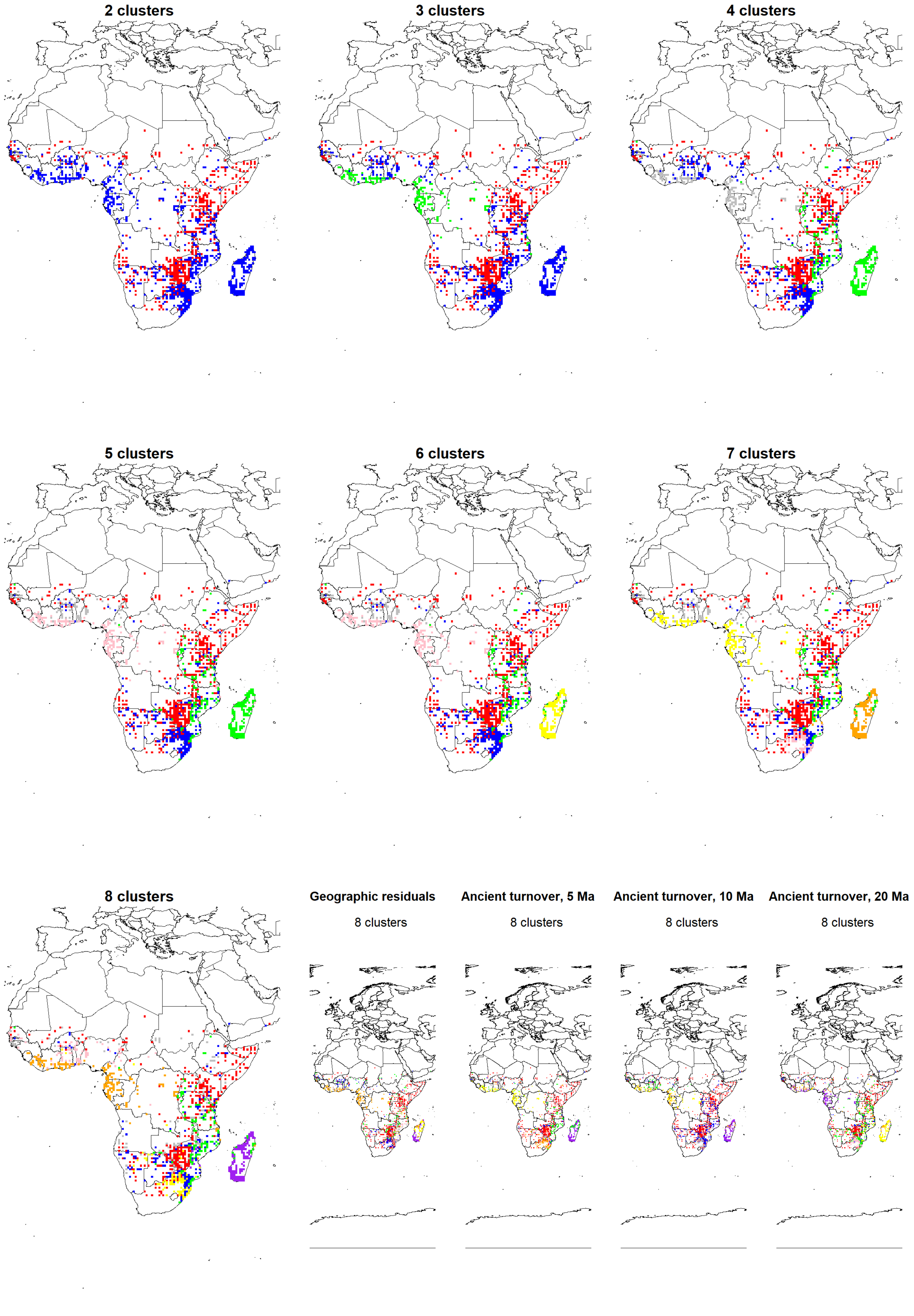

Figure S38. Phyloregionalization of Africa using the metachronogram. Subfigures show clustering results with two to eight phyloregions, as well as the results of phyloregionalization analyses using the geographic residuals of phylogenetic turnover, and ancient phylogenetic turnover with a cut-off of 5, 10, and 20 million years.

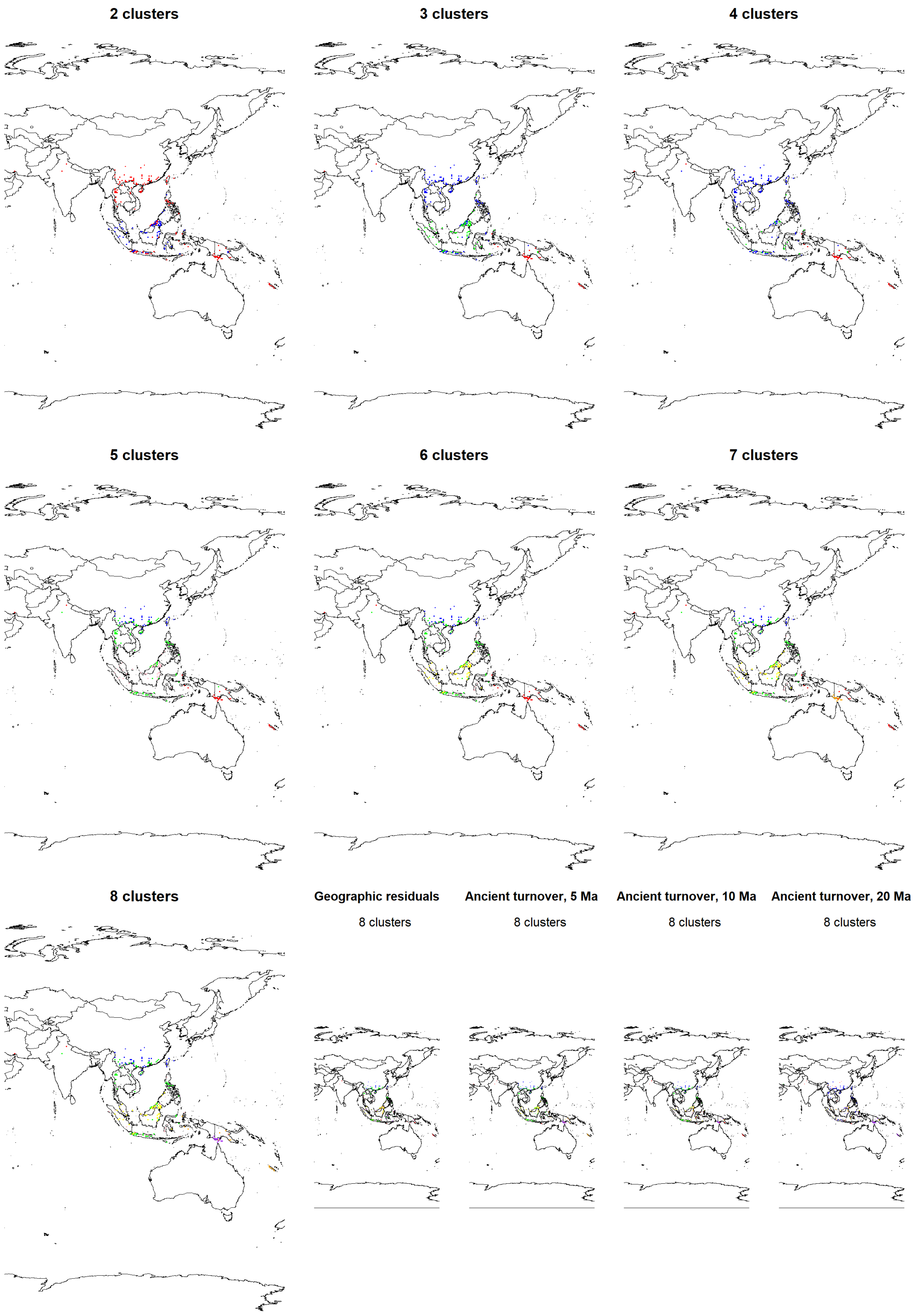

Figure S39. Phyloregionalization of Asia using the metachronogram. Subfigures show clustering results with two to eight phyloregions, as well as the results of phyloregionalization analyses using the geographic residuals of phylogenetic turnover, and ancient phylogenetic turnover with a cut-off of 5, 10, and 20 million years.

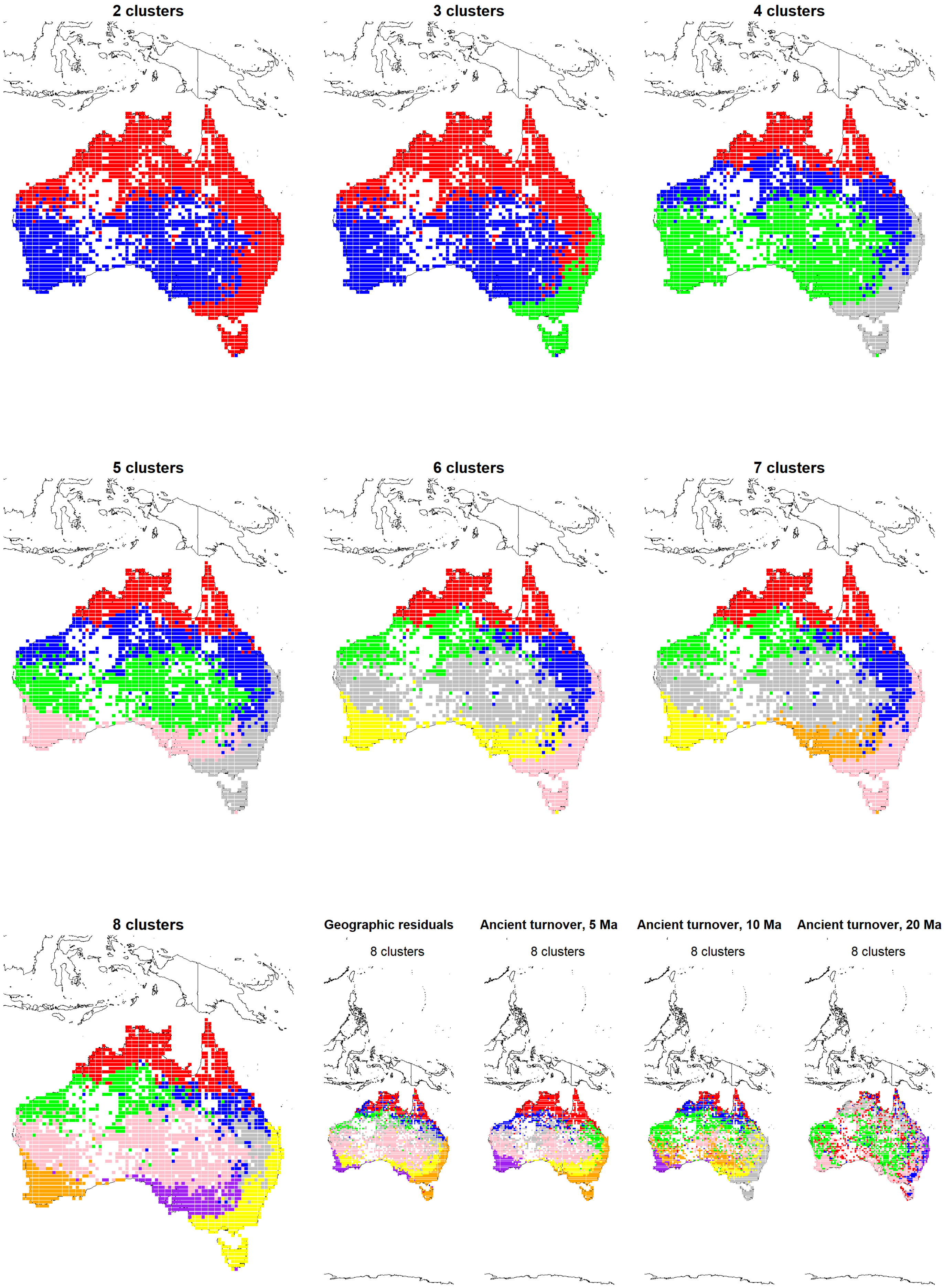

Figure S40. Phyloregionalization of Australia using the metachronogram. Subfigures show clustering results with two to eight phyloregions, as well as the results of phyloregionalization analyses using the geographic residuals of phylogenetic turnover, and ancient phylogenetic turnover with a cut-off of 5, 10, and 20 million years.

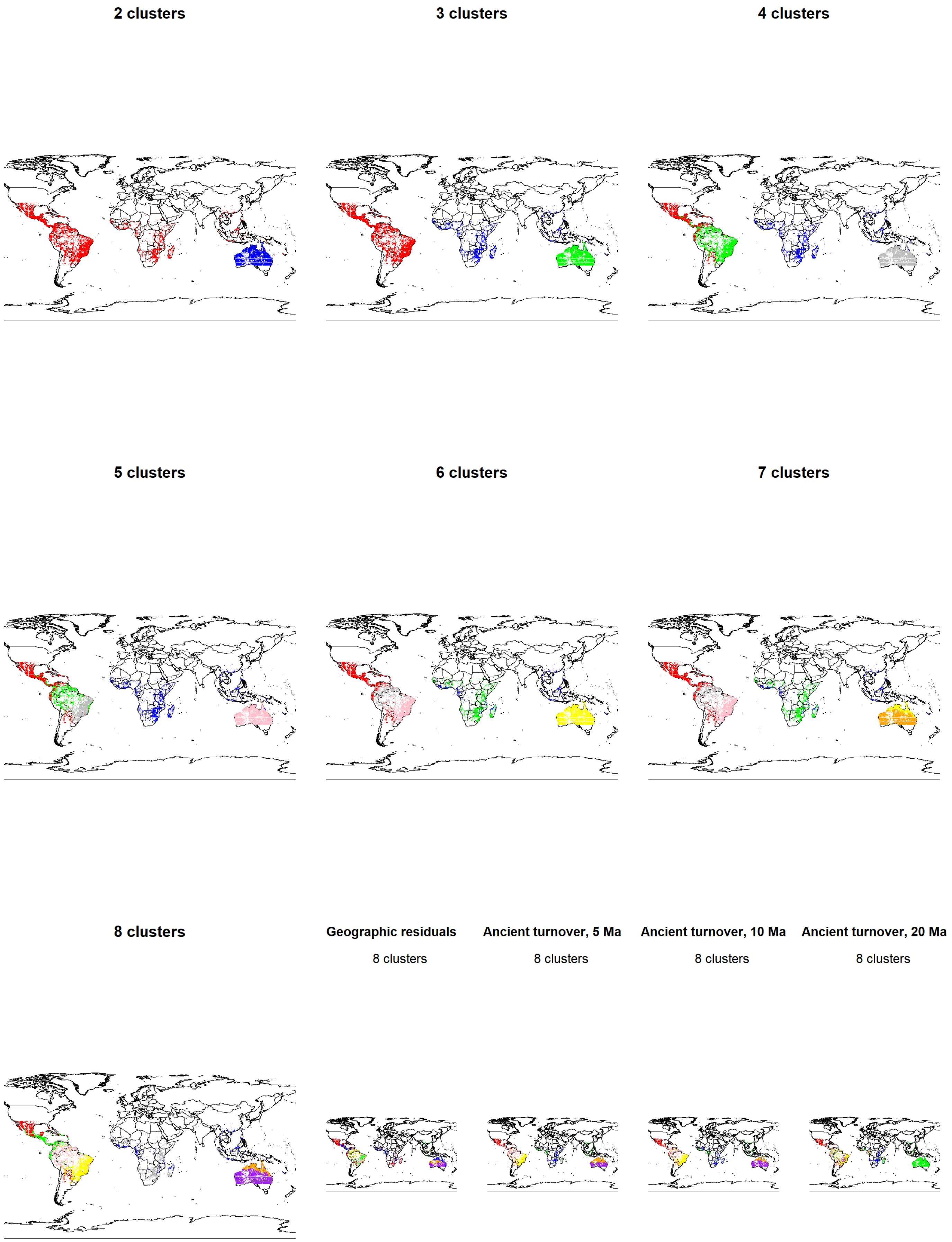

Figure S41. Phyloregionalization of the global tropics using the metachronogram. Subfigures show clustering results with two to eight phyloregions, as well as the results of phyloregionalization analyses using the geographic residuals of phylogenetic turnover, and ancient phylogenetic turnover with a cut-off of 5, 10, and 20 million years.

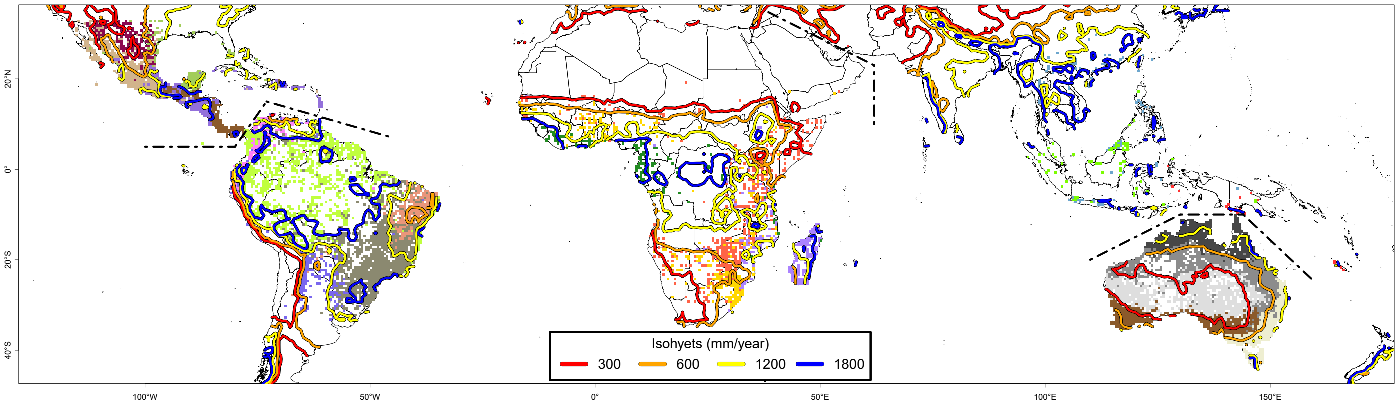

Figure S42. Phyloregionalization per continent using the metachronogram, showing global distribution of isohyets. Caption otherwise as for Figure 3.

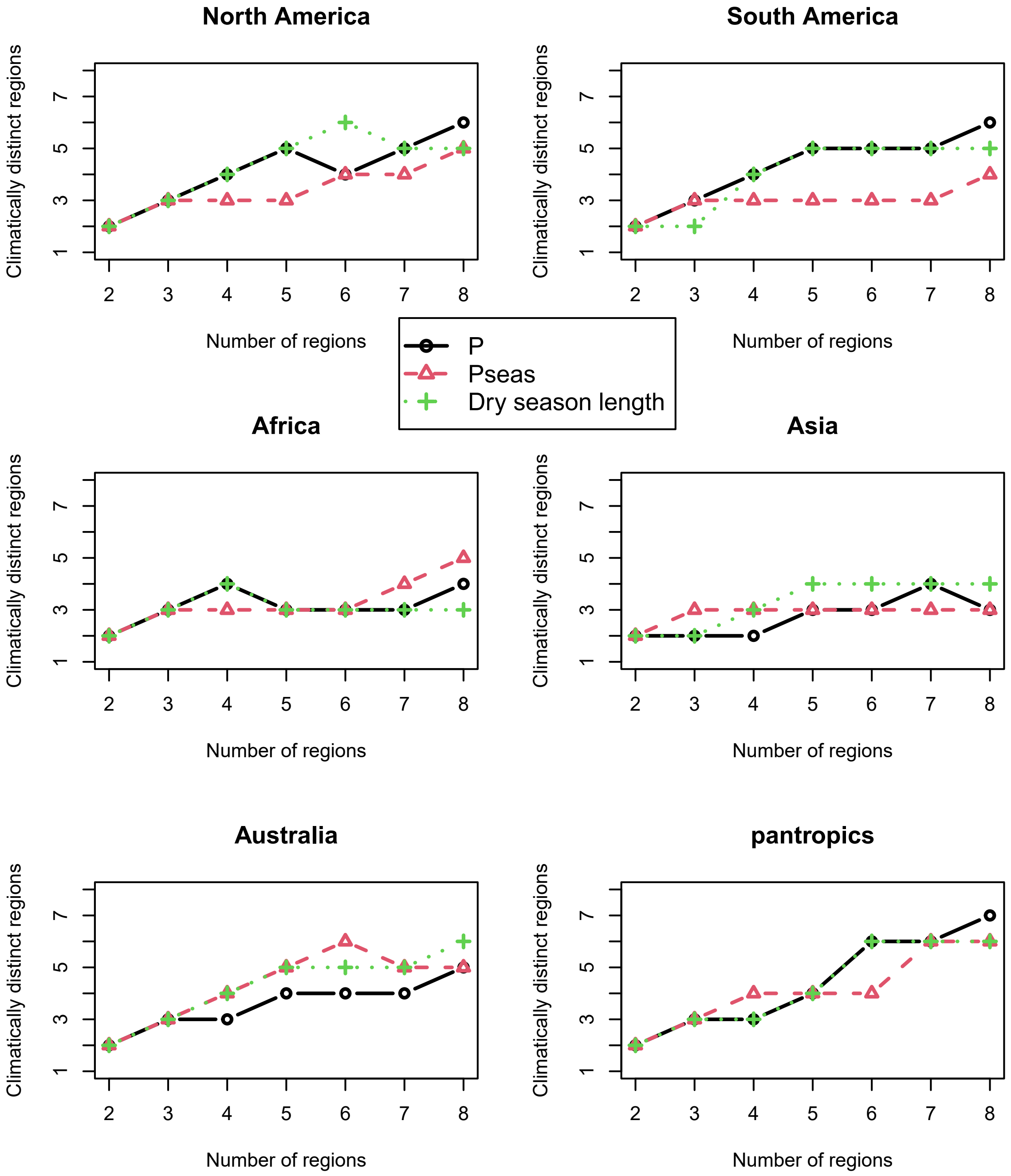

Figure S43. Climatic distinctiveness of phyloregions. Each subplot shows, for phyloregionalization analyses with two to eight phyloregions performed using the metachronogram, how many of the resulting phyloregions have statistically significant different climatic values from the other regions based on mean annual precipitation (P), precipitation seasonality (Pseas), and dry season length.

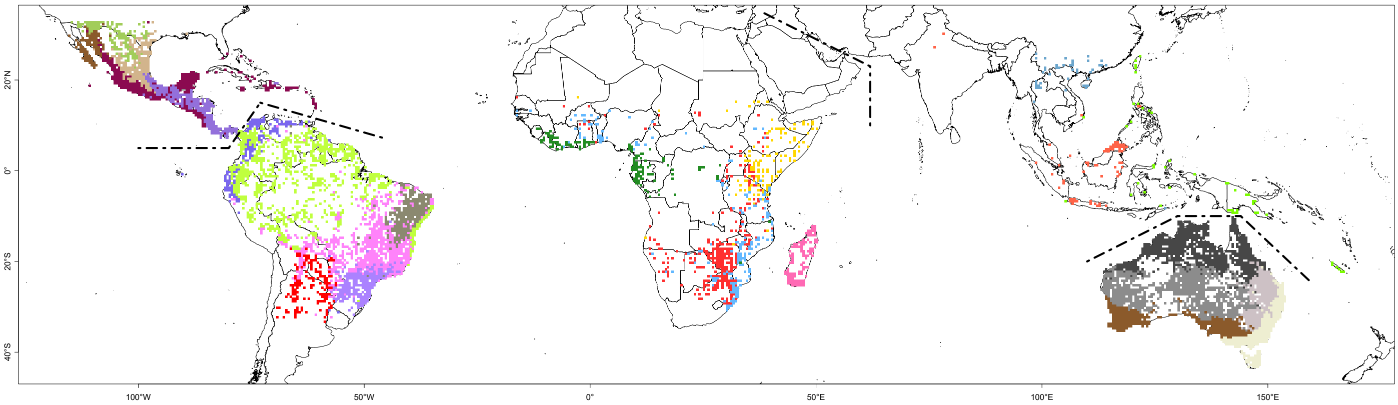

Figure S44. Phyloregionalization per continent using the genus-level Mimosoid phylogeny (rather than the metachronogram). Caption otherwise as for Figure 3.

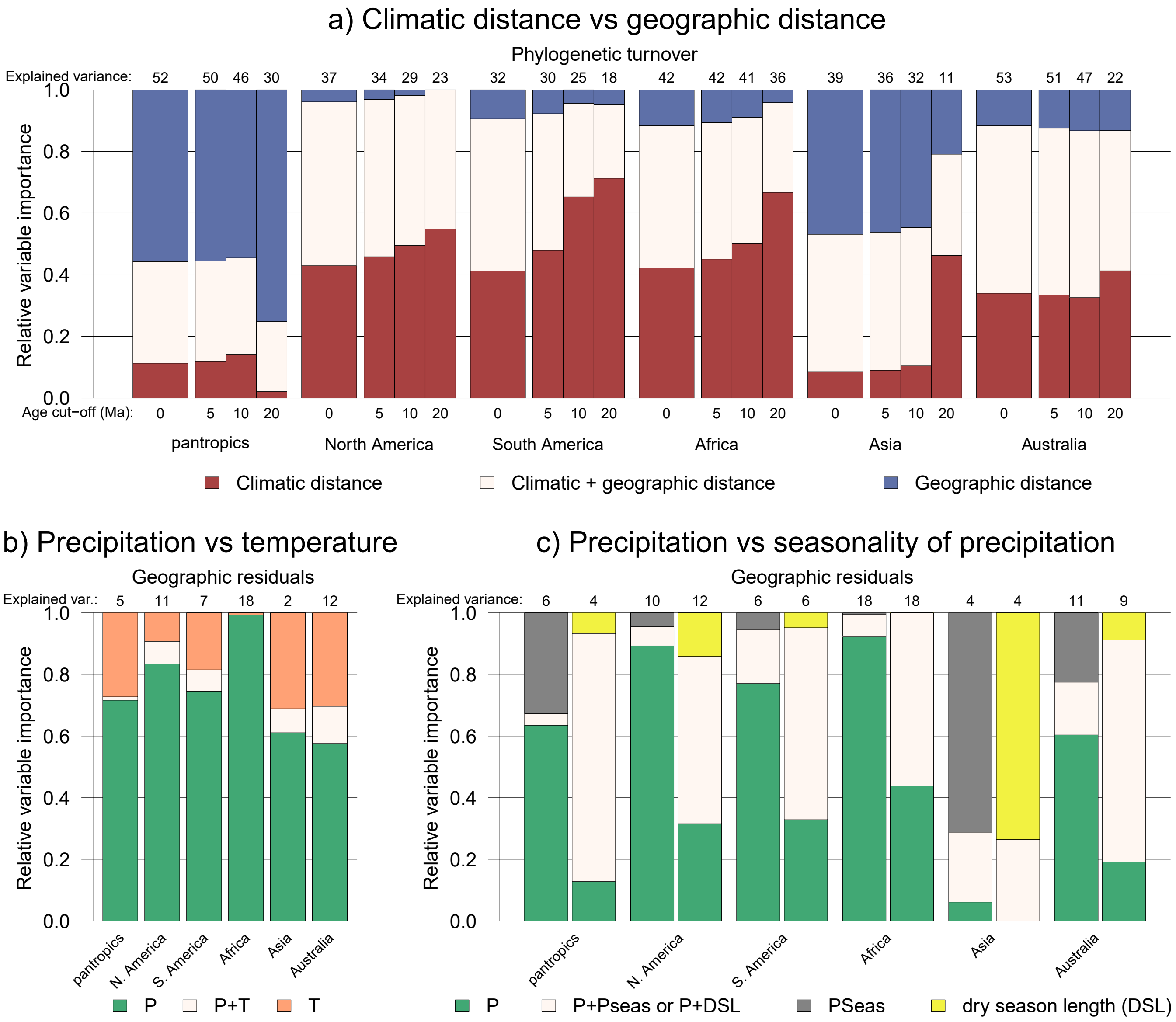

Figure S45. Variation partitioning results obtained using the genus-level Mimosoid phylogeny (rather than the metachronogram). See caption Figure 2 for explanation.

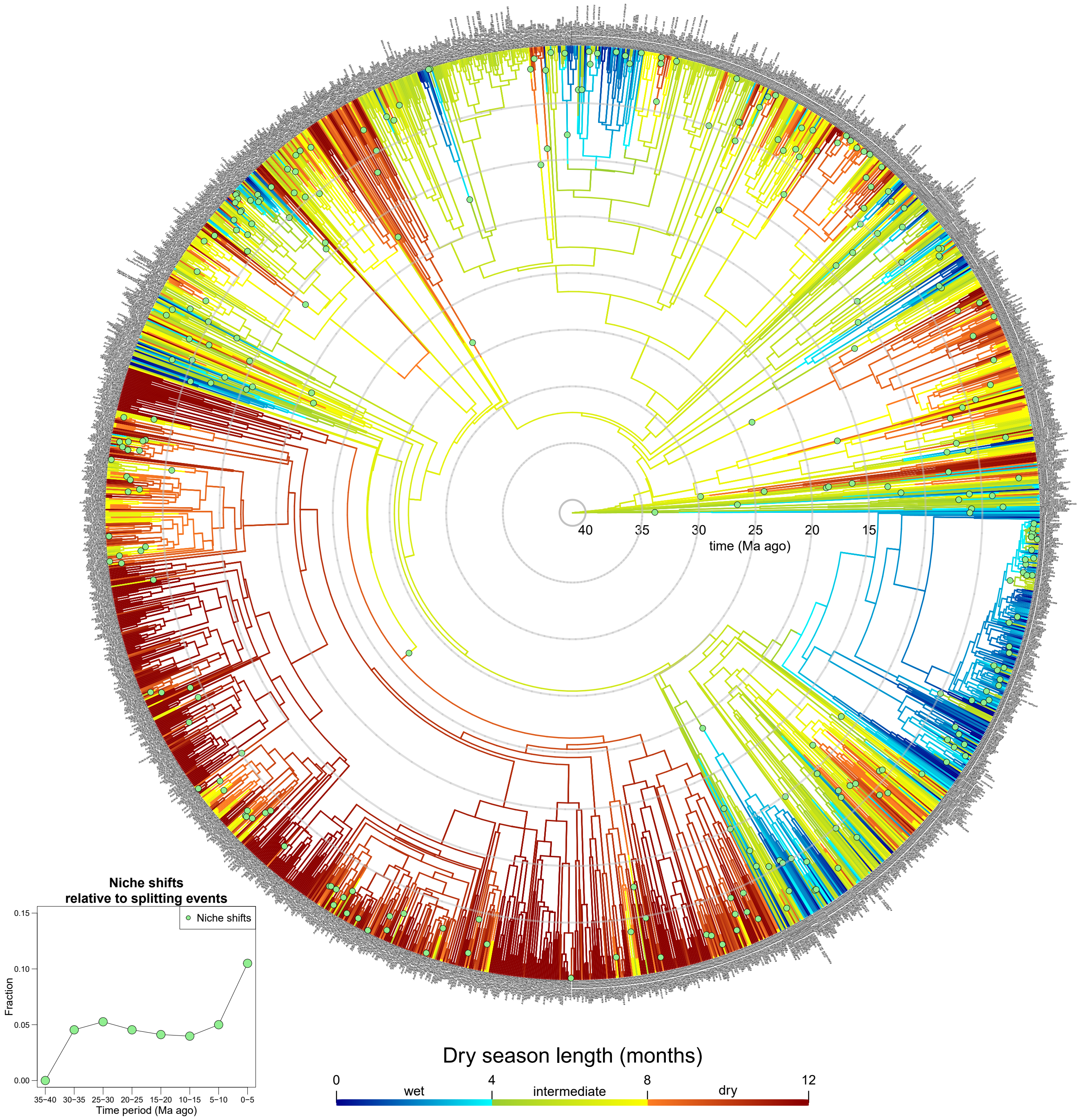

Figure S46. Optimisation of dry season length across the Mimosoid phylogeny. Inset shows the fraction of dry season length niche shifts per speciation event through time. See caption Figure 1 for explanation.

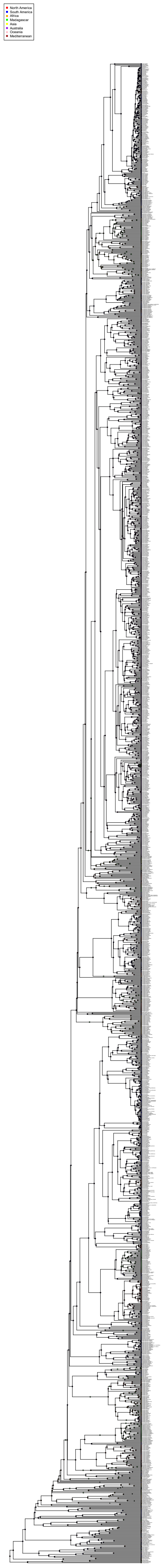

Figure S47. Ancestral range estimation of Caesalpinioideae, performed using BioGeoBEARS with the best-fitting model (i.e., DEC+J). Trans-oceanic dispersal events in the Mimosoid clade, based on a model with seven regions, are indicated with numbered green circles.

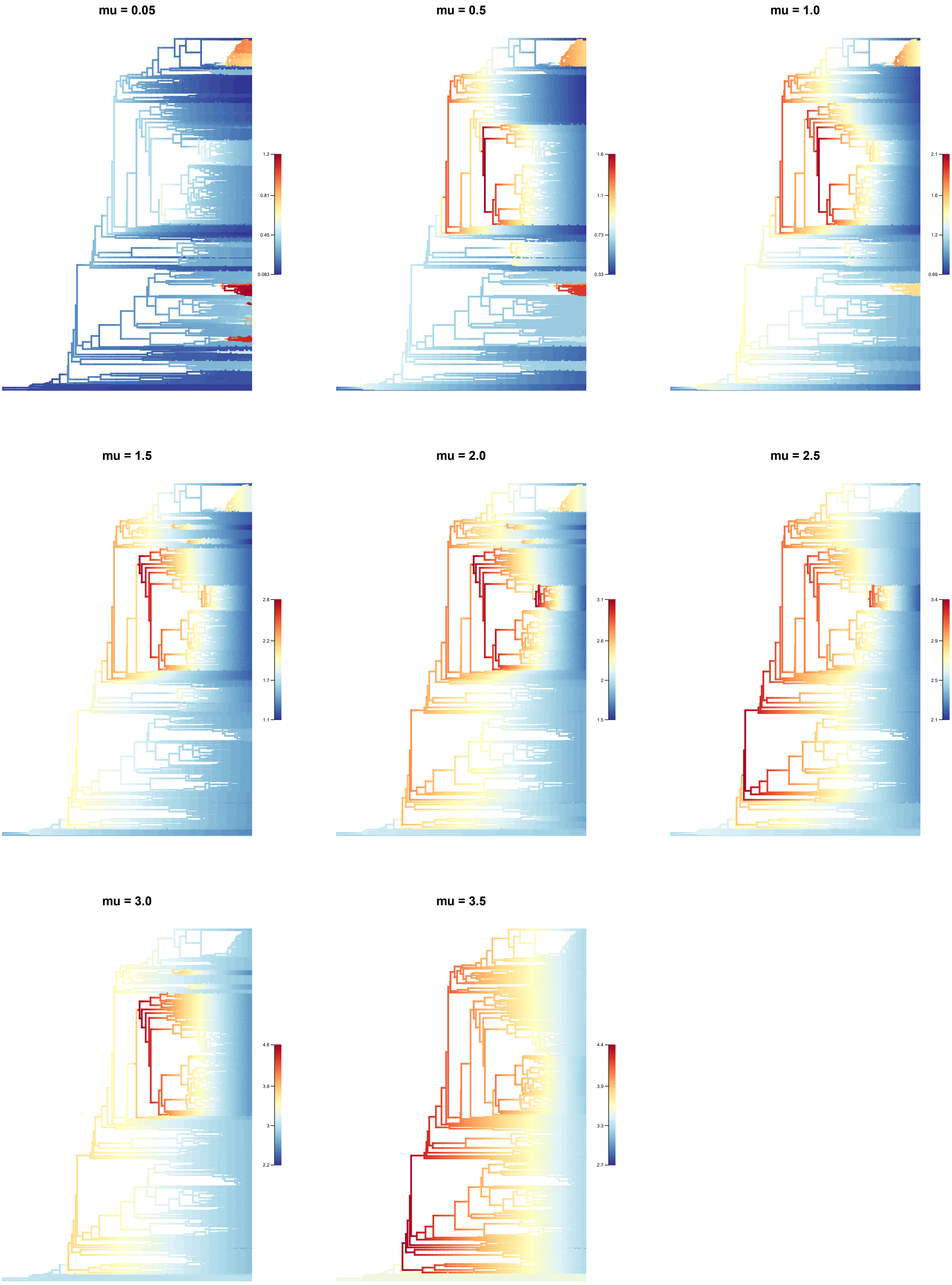

Figure S48. Speciation rates estimated across the Caesalpinioideae metachronogram under eight scenarios with different fixed extinction rates. Extinction rates are shown above each subfigure, while speciation rates are indicated by branch colours.

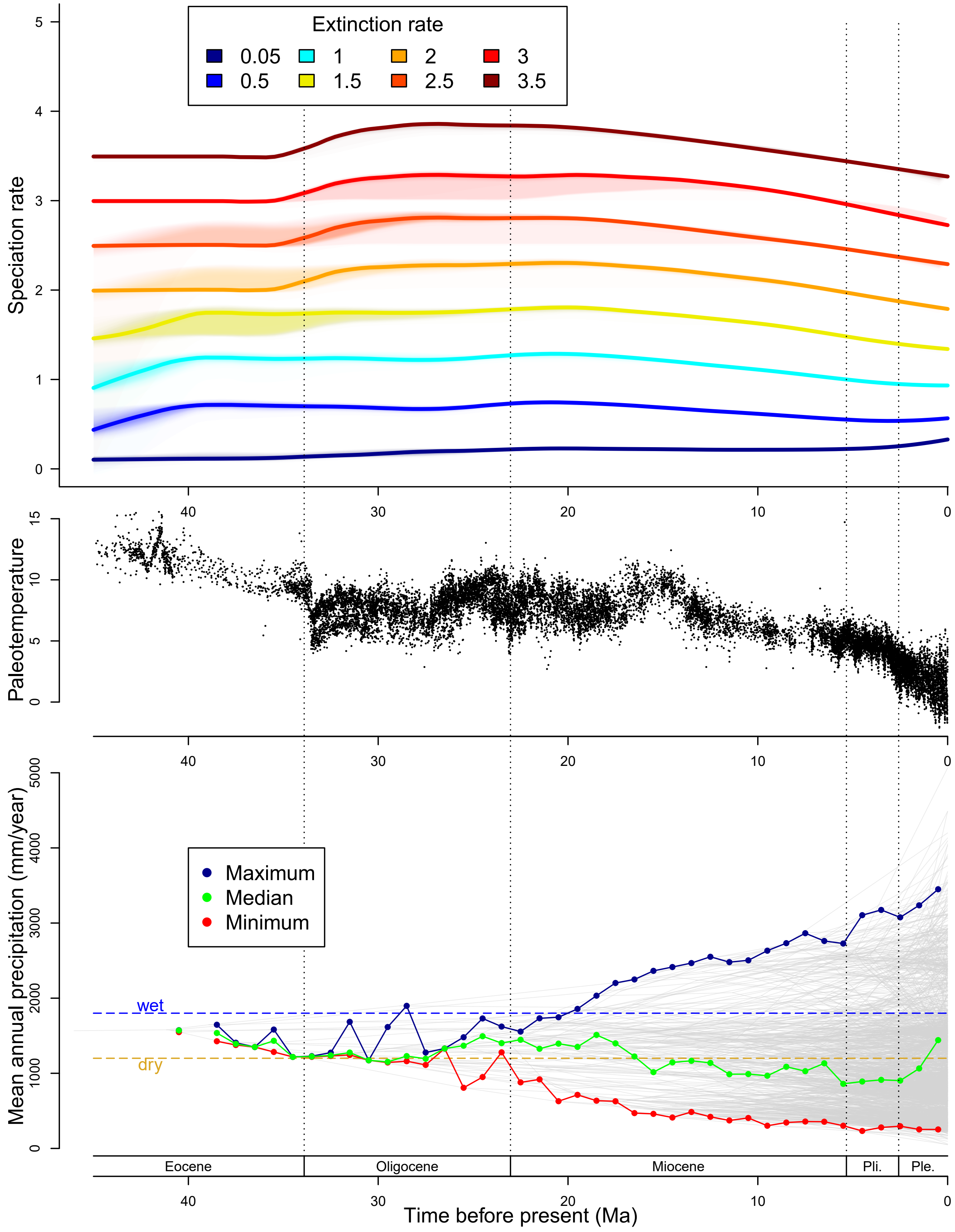

Figure S49. Top: Speciation rates in the Mimosoid clade through time, estimated under different extinction rate scenarios using BAMM. Middle: Paleotemperature inferred from delta O18 measurements, using data from Zachos et al. (*179*). Bottom: Phenogram of mean annual precipitation in the Mimosoid clade through time. Coloured lines with dots show the median, wettest, and driest reconstructed rainfall niche of all nodes in the phylogeny per time bin of one million years.

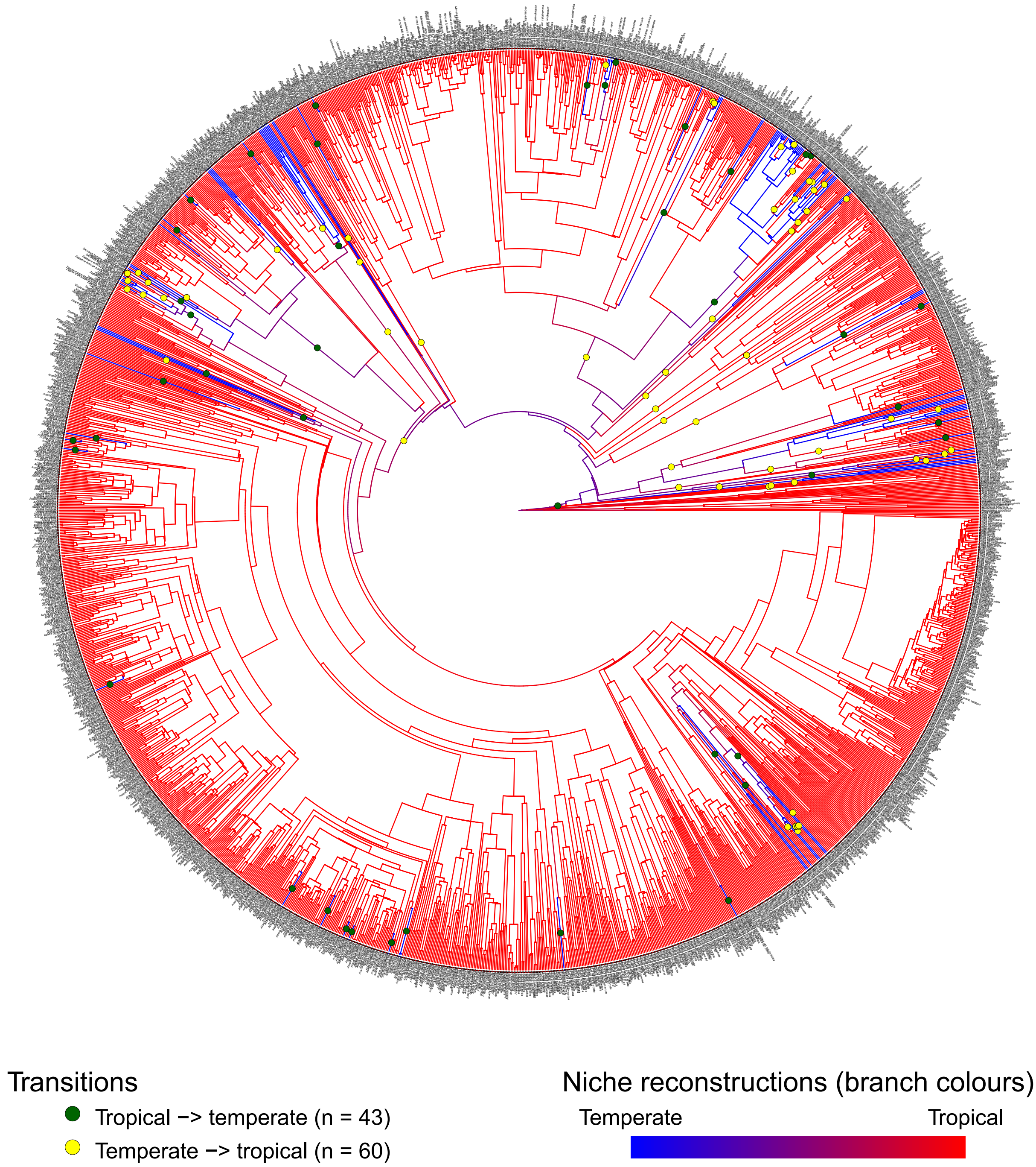

Figure S50. Optimisation of tropical and temperate niches across the Mimosoid phylogeny. Ancestral niches were estimated using a complete metachronogram for Caesalpinioideae, including non-Mimosoid Caesalpinioideae taxa, but only the Mimosoid clade is shown here.

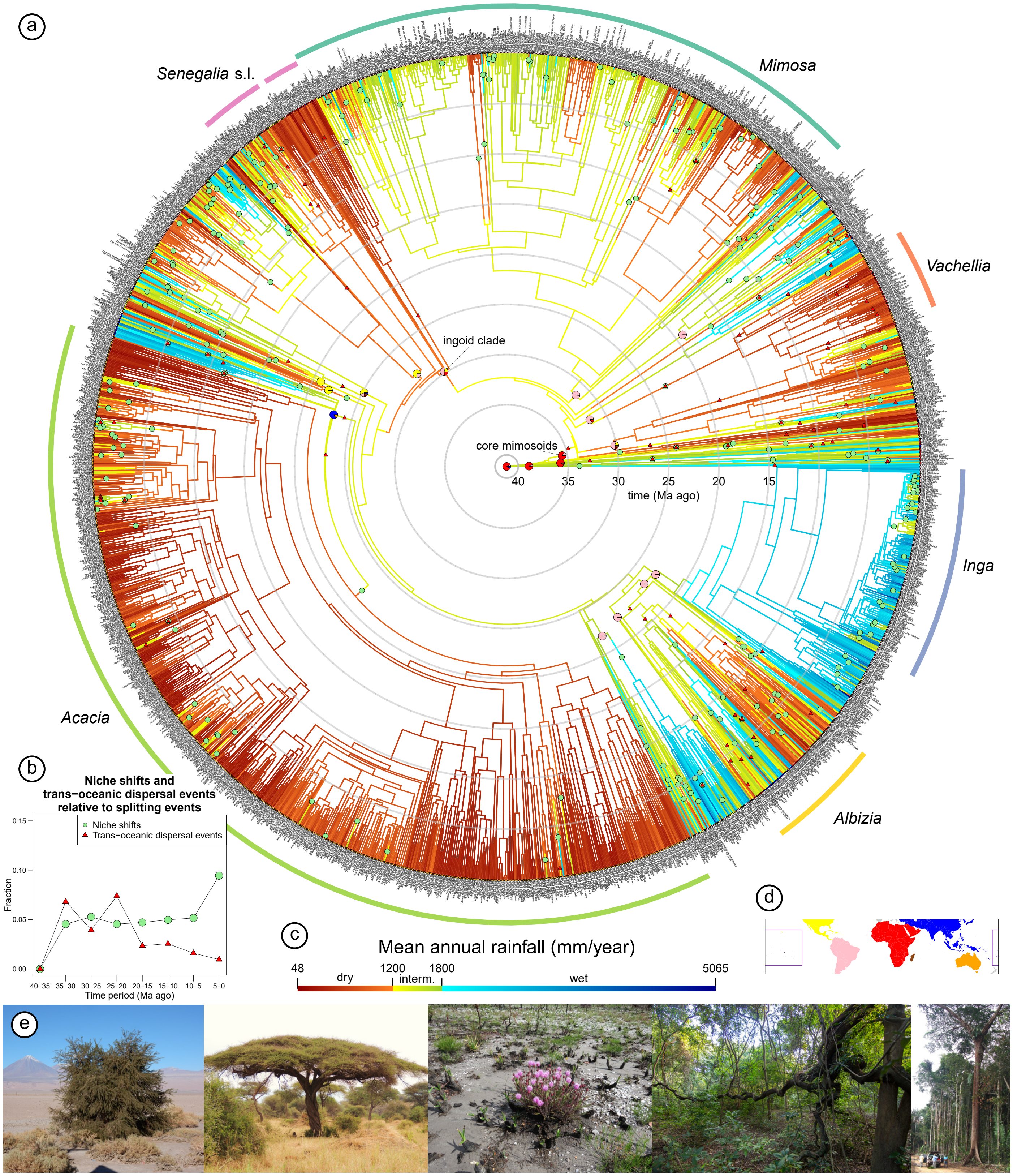

Figure S51. Higher resolution version of Figure 1. See Figure 1 for caption.

REFERENCES AND NOTES

1. A. von Humboldt, A. Bonpland, *Essai sur la Géographie des Plantes; Accompagné d'un Table Physique des Régions Équinoxales* (Levrault, Schoell et Compagnie, Libraires, 1805).

2. G.-L. Buffon, *Histoire Naturelle, Générale et Particuliére* (Imprimerie Royale, 1763), vol. 10.

3. A. P. de Candolle, Essai Élémentaire de Géographie Botanique, in *Dictionnaire des Sciences Naturelles XVIII* (1820).

4. E. J. M. Koenen, J. J. Clarkson, T. D. Pennington, L. W. Chatrou, Recently evolved diversity and convergent radiations of rainforest mahoganies (Meliaceae) shed new light on the origins of rainforest hyperdiversity. *New Phytol.* **207**, 327–339 (2015).

5. R. A. Segovia, R. T. Pennington, T. R. Baker, F. C. de Souza, D. M. Neves, C. C. Davis, J. J. Armesto, A. T. Olivera-Filho, K. G. Dexter, Freezing and water availability structure the evolutionary diversity of trees across the Americas. *Sci. Adv.* **6**, eaaz5373 (2020).

6. W. D. Kissling, W. L. Eiserhardt, W. J. Baker, F. Borchsenius, T. L. P. Couvreur, H. Balslev, J.-C. Svenning, Cenozoic imprints on the phylogenetic structure of palm species assemblages worldwide. *Proc. Natl. Acad. Sci. U.S.A.* **109**, 7379–7384 (2012).

7. H. Qian, N. G. Swenson, J. Zhang, Phylogenetic beta diversity of angiosperms in North America. *Glob. Ecol. Biogeogr.* **22**, 1152–1161 (2013).

8. W. L. Eiserhardt, J.-C. Svenning, W. J. Baker, T. L. P. Couvreur, H. Balslev, Dispersal and niche evolution jointly shape the geographic turnover of phylogenetic clades across continents. *Sci. Rep.* **3**, 1164 (2013).

9. M. R. Carvalho, C. A. Jaramillo, F. de la Parra, D. Caballero-Rodríguez, F. Herrera, S. L. Wing, B. L. Turner, C. D'Apolito, M. Romero-Báez, P. Narváez, C. Martínez, M. Gutierrez, C. Labandeira, G. Bayona, M. J. Rueda, M. Paez-Reyes, D. Cárdenas, Á. Duque, J. L. Crowley, C. Santos, D. Silvestro, Extinction at the end-Cretaceous and the origin of modern Neotropical rainforests. *Science* **372**, 63–68 (2021).

10. Y. Xing, R. E. Onstein, R. J. Carter, T. Stadler, H. P. Linder, Fossils and a large molecular phylogeny show that the evolution of species richness, generic diversity, and turnover rates are disconnected. *Evolution* **68**, 2821–2832 (2014).

11. M. J. Donoghue, A phylogenetic perspective on the distribution of plant diversity. *Proc. Natl. Acad. Sci. U.S.A.* **105**, 11549–11555 (2008).

12. C. H. Graham, P. V. A. Fine, Phylogenetic beta diversity: Linking ecological and evolutionary processes across space in time. *Ecol. Lett.* **11**, 1265–1277 (2008).

13. C. König, P. Weigelt, H. Kreft, Dissecting global turnover in vascular plants. *Glob. Ecol. Biogeogr.* **26**, 228–242 (2017).

14. F. Mazel, R. O. Wüest, J.-P. Lessard, J. Renaud, G. F. Ficetola, S. Lavergne, W. Thuiller, Global patterns of β-diversity along the phylogenetic time-scale: The role of climate and plate tectonics. *Glob. Ecol. Biogeogr.* **26**, 1211–1221 (2017).

15. D. M. Neves, A. J. Kerkhoff, S. Echeverría-Londoño, C. Merow, N. Morueta-holme, R. K. Peet, B. Sandel, J.-C. Svenning, S. K. Wiser, B. J. Enquist, The adaptive challenge of extreme conditions shapes evolutionary diversity of plant assemblages at continental scales. *Proc. Natl. Acad. Sci. U.S.A.* **118**, e2021132118 (2021).

16. A. J. Kerkhoff, P. E. Moriarty, M. D. Weiser, The latitudinal species richness gradient in New World woody angiosperms is consistent with the tropical conservatism hypothesis. *Proc. Natl. Acad. Sci. U.S.A.* **111**, 8125–8130 (2014).

17. D. M. Neves, K. G. Dexter, T. R. Baker, F. Coelho de Souza, A. T. Oliveira-Filho, L. P. Queiroz, H. C. Lima, M. F. Simon, G. P. Lewis, R. A. Segovia, L. Arroyo, C. Reynel, J. L. Marcelo-Peña, I. Huamantupa-Chuquimaco, D. Villarroel, G. A. Parada, A. Daza, R. Linares-Palomino, L. V. Ferreira, R. P. Salomão, G. S. Siqueira, M. T. Nascimento, C. N. Fraga, R. T. Pennington, Evolutionary diversity in tropical tree communities peaks at intermediate precipitation. *Sci. Rep.* **10**, 1188 (2020).

18. T. F. Rangel, N. R. Edwards, P. B. Holden, J. A. F. Diniz-Filho, W. D. Gosling, M. T. P. Coelho, F. A. S. Cassemiro, C. Rahbek, R. K. Colwell, Modeling the ecology and evolution of biodiversity: Biogeographical cradles, museums, and graves. *Science* **361**, eaar5452 (2018).

19. F. P. Peixoto, F. Villalobos, A. S. Melo, J. A. F. Diniz-Filho, R. Loyola, T. F. Rangel, M. V. Cianciaruso, Geographical patterns of phylogenetic beta-diversity components in terrestrial mammals. *Glob. Ecol. Biogeogr.* **26**, 573–583 (2017).

20. J. J. Ringelberg, N. E. Zimmermann, A. Weeks, M. Lavin, C. E. Hughes, Biomes as evolutionary arenas: Convergence and conservatism in the trans-continental succulent biome. *Glob. Ecol. Biogeogr.* **29**, 1100–1113 (2020).

21. M. D. Crisp, M. T. K. Arroyo, L. G. Cook, M. A. Gandolfo, G. J. Jordan, M. S. McGlone, P. H. Weston, M. Westoby, P. Wilf, H. P. Linder, Phylogenetic biome conservatism on a global scale. *Nature* **458**, 754–756 (2009).

22. A. Skeels, M. Cardillo, Environmental niche conservatism explains the accumulation of species richness in Mediterranean-hotspot plant genera. *Evolution* **71**, 582–594 (2017).

23. H. Kreft, W. Jetz, Global patterns and determinants of vascular plant diversity. *Proc. Natl. Acad. Sci. U.S.A.* **104**, 5925–5930 (2007).

24. E. J. M. Koenen, C. A. Kidner, É. R. de Souza, M. F. Simon, J. R. V. Iganci, J. A. Nicholls, G. K. Brown, L. P. de Queiroz, M. A. Luckow, G. P. Lewis, R. T. Pennington, C. E. Hughes, Hybrid capture of 964 nuclear genes resolves evolutionary relationships in the mimosoid legumes and reveals the polytomous origins of a large pantropical radiation. *Am. J. Bot.* **107**, 1710–1735 (2020).

25. G. P. Lewis, B. Schrire, B. Mackinder, M. Lock, *Legumes of the World* (Royal Botanic Gardens, 2005).

26. J. J. Ringelberg, E. J. M. Koenen, J. R. Iganci, L. P. De Queiroz, D. J. Murphy, M. Gaudeul, A. Bruneau, M. Luckow, G. P. Lewis, C. E. Hughes, Phylogenomic analysis of 997 nuclear genes reveals the need for extensive generic re-delimitation in Caesalpinioideae (Leguminosae). *PhytoKeys* **205**, 3–58 (2022).

27. M. Lavin, B. P. Schrire, G. P. Lewis, R. T. Pennington, A. Delgado-Salinas, M. Thulin, C. E. Hughes, A. B. Matos, M. F. Wojciechowski, Metacommunity process rather than continental tectonic history better explains geographically structured phylogenies in legumes. *Philos. Trans. R. Soc. Lond. B Biol. Sci.* **359**, 1509–1522 (2004).

28. G. G. Simpson, Mammals and land bridges. *J. Wash. Acad. Sci.* **30**, 137–163 (1940).

29. B. H. Daru, M. van der Bank, T. J. Davies, Unravelling the evolutionary origins of biogeographic assemblages. *Divers. Distrib.* **24**, 313–324 (2018).

30. C. Penone, B. G. Weinstein, C. H. Graham, T. M. Brooks, C. Rondinini, S. B. Hedges, A. D. Davidson, G. C. Costa, Global mammal beta diversity shows parallel assemblage structure in similar but isolated environments. *Proc. Biol. Sci.* **283**, 20161028 (2016).

31. B. Saladin, W. Thuiller, C. H. Graham, S. Lavergne, L. Maiorano, N. Salamin, N. E. Zimmermann, Environment and evolutionary history shape phylogenetic turnover in European tetrapods. *Nat. Commun.* **10**, 249 (2019).

32. G. R. Moncrieff, W. J. Bond, S. I. Higgins, Revising the biome concept for understanding and predicting global change impacts. *J. Biogeogr.* **43**, 863–873 (2016).

33. A. T. Oliveira-Filho, D. Cardoso, B. D. Schrire, G. P. Lewis, R. T. Pennington, T. J. Brummer, J. Rotella, M. Lavin, Stability structures tropical woody plant diversity more than seasonality: Insights into the ecology of high legume-succulent-plant biodiversity. *South African J. Bot.* **89**, 42–57 (2013).

34. M. Pagel, Inferring the historical patterns of biological evolution. *Nature* **401**, 877–884 (1999).

35. R. T. Pennington, M. Lavin, The contrasting nature of woody plant species in different neotropical forest biomes reflects differences in ecological stability. *New Phytol.* **210**, 25–37 (2016).

36. A.-P. Gorel, O. J. Hardy, G. Dauby, K. G. Dexter, R. A. Segovia, K. Steppe, A. Fayolle, Climatic niche lability but growth form conservatism in the African woody flora. *Ecol. Lett.* **25**, 1164–1176 (2022).

37. M. J. Donoghue, M. J. Sanderson, Confluence, synnovation, and depauperons in plant diversification. *New Phytol.* **207**, 260–274 (2015).

38. C. H. Graham, D. Storch, A. Machac, Phylogenetic scale in ecology and evolution. *Glob. Ecol. Biogeogr.* **27**, 175–187 (2018).

39. M. J. Donoghue, E. J. Edwards, Biome shifts and niche evolution in plants. *Annu. Rev. Ecol. Evol. Syst.* **45**, 547–572 (2014).

40. K. J. Feeley, J. T. Stroud, Where on Earth are the "tropics"? *Front. Biogeogr.* **10**, e38649 (2018).

41. A. E. Zanne, D. C. Tank, W. K. Cornwell, D. J. Mcglinn, B. C. O. Meara, A. T. Moles, P. B. Reich, D. L. Royer, D. E. Soltis, P. S. Soltis, N. G. Swenson, L. Warman, F. Hemmings, M. R. Leishman, J. Oleksyn, J. M. Beaulieu, Three keys to the radiation of angiosperms into freezing environments. *Nature* **506**, 89–92 (2014).

42. O. Hagen, A. Skeels, R. Onstein, W. Jetz, L. Pellissier, Earth history events shaped the evolution of uneven biodiversity across tropical moist forests. *Proc. Natl. Acad. Sci. U.S.A.* **118**, e2026347118 (2021).

43. M. J. Donoghue, E. J. Edwards, Model clades are vital for comparative biology, and ascertainment bias is not a problem in practice: A response to Beaulieu and O'Meara (2018). *Am. J. Bot.* **106**, 327–330 (2019).

44. T. Westerhold, N. Marwan, A. J. Drury, D. Liebrand, C. Agnini, E. Anagnostou, J. S. K. Barnet, S. M. Bohaty, D. De Vleeschouwer, F. Florindo, T. Frederichs, D. A. Hodell, A. E. Holbourn, D. Kroon, V. Lauretano, K. Littler, L. J. Lourens, M. Lyle, H. Pälike, U. Röhl, J. Tian, R. H. Wilkens, P. A. Wilson, J. C. Zachos, An astronomically dated record of Earth's climate and its predictability over the last 66 million years. *Science* **369**, 1383–1387 (2020).

45. M. Arakaki, P.-A. Christin, R. Nyffeler, A. Lendel, U. Eggli, R. M. Ogburn, E. Spriggs, M. J. Moore, E. J. Edwards, Contemporaneous and recent radiations of the world's major succulent plant lineages. *Proc. Natl. Acad. Sci. U.S.A.* **108**, 8379–8384 (2011).

46. P. S. Herendeen, in *Advances in Legume Systematics part 4: The Fossil Record*, P. S. Herendeen, D. L. Dilcher, Eds. (Royal Botanic Gardens, 1992), pp. 85–160.

47. S. Magallón-Puebla, S. R. S. Cevallos-Ferriz, Fossil legume fruits from Tertiary strata of Puebla, Mexico. *Can. J. Bot.* **72**, 1027–1038 (1994).

48. L. Calvillo-Canadell, S. R. S. Cevallos-Ferriz, Diverse assemblage of Eocene and Oligocene leguminosae from Mexico. *Int. J. Plant Sci.* **166**, 671–692 (2005).

49. R. E. Ricklefs, A. E. Schwarzbach, S. S. Renner, Rate of lineage origin explains the diversity anomaly in the world's mangrove vegetation. *Am. Nat.* **168**, 805–810 (2006).

50. P. Sanchez-Martinez, J. Martínez-Vilalta, K. G. Dexter, R. A. Segovia, M. Mencuccini, Adaptation and coordinated evolution of plant hydraulic traits. *Ecol. Lett.* **23**, 1599–1610 (2020).

51. R Core Team, R: A language and environment for statistical computing (R Foundation for Statistical Computing, 2022); www.R-project.org/.

52. V. A. Funk, R. J. Bayer, S. Keeley, R. Chan, L. Watson, B. Gemeinholzer, E. E. Schilling, J. L. Panero, B. G. Baldwin, N. Garcia-Jacas, A. Susanna, R. K. Jansen, I. Friis, H. Balslev, Everywhere but Antarctica: Using a supertree to understand the diversity and distribution of the Compositae. *Biol. Skr.* **55**, 343–373 (2005).

53. V. A. Funk, C. D. Specht, Meta-trees: Grafting for a global perspective. *Proc. Biol. Soc. Wash.* **120**, 232–240 (2007).

54. E. L. Spriggs, P. A. Christin, E. J. Edwards, C_4 photosynthesis promoted species diversification during the Miocene grassland expansion. *PLOS ONE* **9**, e97722 (2014).

55. N. Azani, M. Babineau, C. Donovan Bailey, H. Banks, A. R. Barbosa, R. B. Pinto, J. S. Boatwright, L. M. Borges, G. K. Brown, A. Bruneau, E. Candido, D. Cardoso, K.-F. Chung, R. P. Clark, Adilva de S. Conceição, M. Crisp, P. Cubas, A. Delgado-Salinas, K. G. Dexter, J. J. Doyle, J. Duminil, A. N. Egan, M. de la Estrella, M. J. Falcão, D. A. Filatov, A. P. Fortuna-Perez, R. H. Fortunato, E. Gagnon, P. Gasson, J. G. Rando, A. M. Goulart de Azevedo Tozzi, B. Gunn, D. Harris, E. Haston, J. A. Hawkins, P.

S. Herendeen, C. E. Hughes, J. R. V. Iganci, F. Javadi, S. A. Kanu, S. Kazempour-Osaloo, G. C. Kite, B. B. Klitgaard, F. J. Kochanovski, E. J. M. Koenen, L. Kovar, M. Lavin, M. le Roux, G. P. Lewis, H. C. de Lima, M. C. López-Roberts, B. Mackinder, V. H. Maia, V. Malécot, V. F. Mansano, B. Marazzi, S. Mattapha, J. T. Miller, C. Mitsuyuki, T. Moura, D. J. Murphy, M. Nageswara-Rao, B. Nevado, D. Neves, D. I. Ojeda, R. T. Pennington, D. E. Prado, G. Prenner, L. P. de Queiroz, G. Ramos, Fabiana L. Ranzato Filardi, P. G. Ribeiro, M. de Lourdes Rico-Arce, M. J. Sanderson, J. Santos-Silva, W. M. B. São-Mateus, M. J. S. Silva, M. F. Simon, C. Sinou, C. Snak, É. R. de Souza, J. Sprent, K. P. Steele, J. E. Steier, R. Steeves, C. H. Stirton, S. Tagane, B. M. Torke, H. Toyama, D. T. da Cruz, M. Vatanparast, J. J. Wieringa, M. Wink, M. F. Wojciechowski, T. Yahara, T. Yi, E. Zimmerman, A new subfamily classification of the Leguminosae based on a taxonomically comprehensive phylogeny: The Legume Phylogeny Working Group (LPWG). *Taxon* **66**, 44–77 (2017).

56. J. A. Nicholls, R. T. Pennington, E. J. M. Koenen, C. E. Hughes, J. Hearn, L. Bunnefeld, K. G. Dexter, G. N. Stone, C. A. Kidner, Using targeted enrichment of nuclear genes to increase phylogenetic resolution in the neotropical rain forest genus *Inga* (Leguminosae: Mimosoideae). *Front. Plant Sci.* **6**, 710 (2015).

57. E. Gagnon, A. Bruneau, C. E. Hughes, L. de Queiroz, G. P. Lewis, A new generic system for the pantropical *Caesalpinia* group (Leguminosae). *PhytoKeys* **71**, 1–160 (2016).

58. A. G. de Lima, J. de Paula-souza, J. J. Ringelberg, M. F. Simon, L. P. de Queiroz, L. M. Borges, V. de F. Mansano, V. C. Souza, V. R. Scalon, New segregates from the Neotropical genus *Stryphnodendron* (Leguminosae, Caesalpinioideae, mimosoid clade). *PhytoKeys* **205**, 203–237 (2022).

59. S. F. Altschul, W. Gish, W. Miller, E. W. Myers, D. J. Lipman, Basic local alignment search tool. *J. Mol. Biol.* **215**, 403–410 (1990).

60. W. J. Kent, BLAT—The BLAST-like alignment tool. *Genome Res.* **12**, 656–664 (2002).

61. S. Mirarab, Species tree estimation using ASTRAL: Practical considerations. arXiv 1904.03826 (2019)

62. C. Zhang, M. Rabiee, E. Sayyari, S. Mirarab, ASTRAL-III: Polynomial time species tree reconstruction from partially resolved gene trees. *BMC Bioinformatics* **19**, 153 (2018).

63. S. A. Smith, M. J. Moore, J. W. Brown, Y. Yang, Analysis of phylogenomic datasets reveals conflict, concordance, and gene duplications with examples from animals and plants. *BMC Evol. Biol.* **15**, 150 (2015).

64. X. Zhou, S. Lutteropp, L. Czech, A. Stamatakis, M. Von Looz, A. Rokas, Quartet-based computations of Internode Certainty provide robust measures of phylogenetic incongruence. *Syst. Biol.* **69**, 308–324 (2020).

65. D. F. Robinson, L. R. Foulds, Comparison of phylogenetic trees. *Math. Biosci.* **53**, 131–147 (1981).

66. K. P. Schliep, phangorn: Phylogenetic analysis in R. *Bioinformatics* **27**, 592–593 (2011).

67. L. J. Revell, phytools: An R package for phylogenetic comparative biology (and other things). *Methods Ecol. Evol.* **3**, 217–223 (2012).

68. A. J. Aberer, D. Krompass, A. Stamatakis, Pruning rogue taxa improves phylogenetic accuracy: An efficient algorithm and webservice. *Syst. Biol.* **62**, 162–166 (2013).

69. M. A. Suchard, P. Lemey, G. Baele, D. L. Ayres, A. J. Drummond, A. Rambaut, Bayesian phylogenetic and phylodynamic data integration using BEAST 1.10. *Virus Evol.* **4**, vey016 (2018).

70. A. J. Drummond, A. Rambaut, BEAST: Bayesian evolutionary analysis by sampling trees. *BMC Evol. Biol.* **7**, 214 (2007).

71. J. W. Brown, S. A. Smith, The past sure is tense: On interpreting phylogenetic divergence time estimates. *Syst. Biol.* **67**, 340–353 (2018).

72. E. J. M. Koenen, D. I. Ojeda, F. T. Bakker, J. J. Wieringa, C. Kidner, O. J. Hardy, R. T. Pennington, P. S. Herendeen, A. Bruneau, C. E. Hughes, The origin of the Legumes is a complex paleopolyploid phylogenomic tangle closely associated with the Cretaceous–Paleogene (K–Pg) mass extinction event. *Syst. Biol.* **70**, 508–526 (2021).

73. S. A. Smith, J. W. Brown, J. F. Walker, So many genes, so little time: A practical approach to divergence-time estimation in the genomic era. *PLOS ONE* **13**, e0197433 (2018).

74. E. J. M. Koenen, D. I. Ojeda, R. Steeves, J. Migliore, F. T. Bakker, J. J. Wieringa, C. Kidner, O. J. Hardy, R. T. Pennington, A. Bruneau, C. E. Hughes, Large-scale genomic sequence data resolve the deepest divergences in the legume phylogeny and support a near-simultaneous evolutionary origin of all six subfamilies. *New Phytol.* **225**, 1355–1369 (2020).

75. A. Bruneau, M. Mercure, G. P. Lewis, P. S. Herendeen, Phylogenetic patterns and diversification in the caesalpinioid legumes. *Botany* **86**, 697–718 (2008).

76. M. F. Simon, R. Grether, L. P. de Queiroz, C. Skema, R. T. Pennington, C. E. Hughes, Recent assembly of the Cerrado, a neotropical plant diversity hotspot, by in situ evolution of adaptations to fire. *Proc. Natl. Acad. Sci. U.S.A.* **106**, 20359–20364 (2009).

77. C. E. González-Orozco, S. W. Laffan, N. Knerr, J. T. Miller, A biogeographical regionalization of Australian *Acacia* species. *J. Biogeogr.* **40**, 2156–2166 (2013).

78. E. Gagnon, J. J. Ringelberg, A. Bruneau, G. P. Lewis, C. E. Hughes, Global Succulent Biome phylogenetic conservatism across the pantropical Caesalpinia Group (Leguminosae). *New Phytol.* **222**, 1994–2008 (2019).

79. H. ter Steege, N. C. A. Pitman, D. Sabatier, C. Baraloto, R. P. Salomão, J. E. Guevara, O. L. Phillips, C. V. Castilho, W. E. Magnusson, J.-F. Molino, A. Monteagudo, P. N. Vargas, J. C. Montero, T. R. Feldpausch, E. N. H. Coronado, T. J. Killeen, B. Mostacedo, R. Vasquez, R. L. Assis, J. Terborgh, F. Wittmann, A. Andrade, W. F. Laurance, S. G. W. Laurance, B. S. Marimon, B.-H. Marimon Jr, I. C. G. Vieira, I. L. Amaral, R. Brienen, H. Castellanos, D. C. López, J. F. Duivenvoorden, H. F. Mogollón, F. D. de Almeida Matos, N. Dávila, R. García-Villacorta, P. R. S. Diaz, F. Costa, T. Emilio, C. Levis, J. Schietti, P. Souza, A. Alonso, F. Dallmeier, A. J. D. Montoya, M. T. F. Piedade, A. Araujo-Murakami, L. Arroyo, R. Gribel, P. V. A. Fine, C. A. Peres, M. Toledo, G. A. Aymard C, T. R. Baker, C. Cerón, J. Engel, T. W. Henkel, P. Maas, P. Petronelli, J. Stropp, C. E. Zartman, D. Daly, D. Neill, M. Silveira, M. R. Paredes, J. Chave, D. de Andrade Lima Filho, P. M. Jørgensen, A. Fuentes, J. Schöngart, F. C. Valverde, A. D. Fiore, E. M. Jimenez, M. C. P. Mora, J. F. Phillips, G. Rivas, T. R. van Andel, P. von Hildebrand, B. Hoffman, E. L. Zent, Y. Malhi, A. Prieto, A. Rudas, A. R. Ruschell, N. Silva, V. Vos, S. Zent, A. A. Oliveira, A. C. Schutz, T. Gonzales, M. T. Nascimento, H. Ramirez-Angulo, R. Sierra, M. Tirado, M. N. U. Medina, G. van der Heijden, C. I. A. Vela, E. V. Torre, C. Vriesendorp, O. Wang, K.

R. Young, C. Baider, H. Balslev, C. Ferreira, I. Mesones, A. Torres-Lezama, L. E. U. Giraldo, R. Zagt, M. N. Alexiades, L. Hernandez, I. Huamantupa-Chuquimaco, W. Milliken, W. P. Cuenca, D. Pauletto, E. V. Sandoval, L. V. Gamarra, K. G. Dexter, K. Feeley, G. Lopez-Gonzalez, M. R. Silman, Hyperdominance in the Amazonian tree flora. *Science* **342**, 1243092 (2013).

80. D. Cardoso, T. Särkinen, S. Alexander, A. M. Amorim, V. Bittrich, M. Celis, D. C. Daly, P. Fiaschi, V. A. Funk, L. L. Giacomin, R. Goldenberg, G. Heiden, J. Iganci, C. L. Kelloff, S. Knapp, H. Cavalcante de Lima, A. F. P. Machado, R. M. dos Santos, R. Mello-Silva, F. A. Michelangeli, J. Mitchell, P. Moonlight, P. L. R. de Moraes, S. A. Mori, T. S. Nunes, T. D. Pennington, J. R. Pirani, G. T. Prance, L. P. de Queiroz, A. Rapini, R. Riina, C. A. V. Rincon, N. Roque, G. Shimizu, M. Sobral, J. R. Stehmann, W. D. Stevens, C. M. Taylor, M. Trovó, C. van den Berg, H. van der Werff, P. L. Viana, C. E. Zartman, R. C. Forzza, Amazon plant diversity revealed by a taxonomically verified species list. *Proc. Natl. Acad. Sci. U.S.A.* **114**, 10695–10700 (2017).

81. DRYFLOR; K. Banda-R, A. Delgado-Salinas, K. G. Dexter, R. Linares-Palomino, A. Oliveira-Filho, D. Prado, M. Pullan, C. Quintana, R. Riina, G. M. Rodríguez, J. Weintritt, P. Acevedo-Rodríguez, J. Adarve, E. Álvarez, B. Aranguren, G. A. Julián Camilo Arteaga, A. Castaño, Á. C. Natalia Ceballos-Mago, H. Cuadros, D. Freddy, W. Devia, H. Dueñas, L. Fajaro, Á. Fernández, M. Á. Fernández, J. Franklin, E. H. Freid, L. A. Galetti, R. Gonto, R. González M, R. Graveson, E. H. Helmer, Á. Idárrago, R. Lopéz, H. Marcano-Vega, O. G. Martínez, H. M. Maturo, M. McDonald, K. McLaren, O. Melo, F. Mijares, V. Mogni, D. Molina, N. D. P. Moreno, J. M. Nassar, D. M. Neves, L. J. Oakley, M. Oatham, A. R. Olvera-Luna, F. F. Pezzini, O. J. R. Dominguez, M. E. Ríos, O. Rivera, N. Rodríguez, A. Rojas, T. E. Särkinen, R. Sánchez, M. Smith, C. Vargas, B. Villanueva, R. T. Pennington, Plant diversity patterns in neotropical dry forests and their conservation implications. *Science* **353**, 1383–1387 (2016).

82. A. Fayolle, M. D. Swaine, J. Aleman, A. F. Azihou, D. Bauman, M. te Beest, E. N. Chidumayo, J. P. G. M. Cromsigt, H. Dessard, M. Finckh, F. M. P. Gonçalves, J. F. Gillet, A. Gorel, A. Hick, R. Holdo, B. Kirunda, G. Mahy, I. McNicol, C. M. Ryan, R. Revermann, A. Plumptre, R. Pritchard, P. Nieto-Quintano, C. B. Schmitt, J. Seghieri, A. Swemmer, H. Talila, E. Woollen, A sharp floristic discontinuity revealed by the biogeographic regionalization of African savannas. *J. Biogeogr.* **46**, 454–465 (2019).

83. A. Baselga, Partitioning the turnover and nestedness components of beta diversity. *Glob. Ecol. Biogeogr.* **19**, 134–143 (2010).

84. F. Leprieur, C. Albouy, J. de Bortoli, P. F. Cowman, D. R. Bellwood, D. Mouillot, Quantifying phylogenetic beta diversity: Distinguishing between 'true' turnover of lineages and phylogenetic diversity gradients. *PLOS ONE* **7**, (2012).

85. G. G. Simpson, Mammals and the nature of continents. *Am. J. Sci.* **241**, 1–31 (1943).

86. H. Kreft, W. Jetz, A framework for delineating biogeographical regions based on species distributions. *J. Biogeogr.* **37**, 2029–2053 (2010).

87. A. Castro-Insua, C. Gómez-Rodríguez, A. Baselga, Dissimilarity measures affected by richness differences yield biased delimitations of biogeographic realms. *Nat. Commun.* **9**, 5085 (2018).

88. A. Baselga, D. Orme, S. Villeger, J. de Bortoli, F. Leprieur, *betapart: Partitioning Beta Diversity into Turnover and Nestedness Components* (2018); R package version 1.5.1.

89. D. Nychka, R. Furrer, J. Paige, S. Sain, *fields: Tools for Spatial Data* doi: 10.5065/D6W957CT (2017); R package version 10.3; https://github.com/NCAR/Fields.

90. M. C. Fitzpatrick, K. Mokany, G. Manion, M. Lisk, S. Ferrier, D. Nieto-Lugilde, *gdm: Generalized Dissimilarity Modeling* (2020); R package version 1.4.

91. D. N. Karger, O. Conrad, J. Böhner, T. Kawohl, H. Kreft, R. W. Soria-Auza, N. E. Zimmermann, H. P. Linder, M. Kessler, Climatologies at high resolution for the earth's land surface areas. *Sci. Data.* **4**, 170122 (2017).

92. A. M. Wilson, W. Jetz, Remotely sensed high-resolution global cloud dynamics for predicting ecosystem and biodiversity distributions. *PLOS Biol.* **14**, e1002415 (2016).

93. R. J. Hijmans, *raster: Geographic Data Analysis and Modeling* (2018); R package version 2.8–4; https://CRAN.R-project.org/package=raster.

94. D. F. Rosauer, S. Ferrier, K. J. Williams, G. Manion, J. S. Keogh, S. W. Laffan, Phylogenetic generalised dissimilarity modelling: A new approach to analysing and predicting spatial turnover in the phylogenetic composition of communities. *Ecography* **37**, 21–32 (2014).

95. S. Ferrier, G. Manion, J. Elith, K. Richardson, Using generalized dissimilarity modelling to analyse and predict patterns of beta diversity in regional biodiversity assessment. *Divers. Distrib.* **13**, 252–264 (2007).

96. D. L. Warren, M. Cardillo, D. F. Rosauer, D. I. Bolnick, Mistaking geography for biology: Inferring processes from species distributions. *Trends Ecol. Evol.* **29**, 572–580 (2014).

97. I. R. McFadden, M. T. P. Coelho, R. O. Wüest, F. A. S. Cassemiro, N. E. Zimmermann, L. Pellissier, T. F. Rangel, C. H. Graham, Global hotspots of recent and ancestral turnover in birds. Research Square 10.21203/rs.3.rs-131370/v2 (2020).

98. D. M. Olson, E. Dinerstein, E. D. Wikramanayake, N. D. Burgess, G. V. N. Powell, E. C. Underwood, J. A. D'amico, I. Itoua, H. E. Strand, J. C. Morrison, C. J. Loucks, T. F. Allnutt, T. H. Ricketts, Y. Kura, J. F. Lamoreux, W. W. Wettengel, P. Hedao, K. R. Kassem, Terrestrial ecoregions of the World: A new map of life on Earth. *Bioscience* **51**, 933–938 (2001).

99. S. I. Higgins, R. Buitenwerf, G. R. Moncrieff, Defining functional biomes and monitoring their change globally. *Glob. Chang. Biol.* **22**, 3583–3593 (2016).

100. R. T. Pennington, C. E. R. Lehmann, L. M. Rowland, Tropical savannas and dry forests. *Curr. Biol.* **28**, R541–R545 (2018).

101. K. G. Dexter, R. T. Pennington, A. T. Oliveira-Filho, M. L. Bueno, P. L. Silva de Miranda, D. M. Neves, Inserting tropical dry forests into the discussion on biome transitions in the tropics. *Front. Ecol. Evol.* **6**, 104 (2018).

102. R. T. Corlett, R. B. Primack, *Tropical Rain Forests* (Wiley, 2011).

103. C. E. R. Lehmann, D. M. Griffith, K. J. Simpson, T. M. Anderson, S. Archibald, D. J. Beerling, W. J. Bond, E. Denton, E. J. Edwards, E. J. Forrestel, D. L. Fox, D. Georges, W. A. Hoffmann, T. A. Kluyver,

L. Mucina, S. Pau, J. Ratnam, N. Salamin, B. Santini, M. D. Smith, E. L. Spriggs, R. Westley, C. J. Still, C. A. E. Strömberg, C. P. Osborne, Functional diversification enabled grassy biomes to fill global climate space. bioRxiv 10.1101/583625 (2019).

104. B. H. Daru, T. L. Elliott, D. S. Park, T. J. Davies, Understanding the processes underpinning patterns of phylogenetic regionalization. *Trends Ecol. Evol.* **32**, 845–860 (2017).

105. B. G. Holt, J.-P. Lessard, M. K. Borregaard, S. A. Fritz, M. B. Araújo, D. Dimitrov, P.-H. Fabre, C. H. Graham, G. R. Graves, K. A. Jønsson, D. Nogués-Bravo, Z. Wang, R. J. Whittaker, J. Fjeldså, C. Rahbek, An update of Wallace's zoogeographic regions of the world. *Science* **339**, 74–78 (2013).

106. D. H. Ogle, J. C. Doll, P. Wheeler, A. Dinno, *FSA: Fisheries Stock Analysis* (2021); R package version 0.9.1; https://github.com/droglenc/FSA.

107. A. Dinno, dunn.test: *Dunn's Test of Multiple Comparisons Using Rank Sums* (2017); R package version 1.3.5; https://CRAN.R-project.org/package=dunn.test.

108. W. L. Eiserhardt, T. L. P. Couvreur, W. J. Baker, Plant phylogeny as a window on the evolution of hyperdiversity in the tropical rainforest biome. *New Phytol.* **214**, 1408–1422 (2017).

109. N. J. Matzke, Probabilistic historical biogeography: New models for founder-event speciation, imperfect detection, and fossils allow improved accuracy and model-testing. *Front. Biogeogr.* **5**, 242–248 (2013).

110. N. J. Matzke, *BioGeoBEARS: BioGeography with Bayesian (and Likelihood) Evolutionary Analysis in R Scripts* (2013); R package version 0.2.1.

111. N. J. Matzke, Model selection in historical biogeography reveals that founder-event speciation is a crucial process in island clades. *Syst. Biol.* **63**, 951–970 (2014).

112. D. L. Rabosky, Automatic detection of key innovations, rate shifts, and diversity-dependence on phylogenetic trees. *PLOS ONE* **9**, e89543 (2014).

113. B. R. Moore, S. Höhna, M. R. May, B. Rannala, J. P. Huelsenbeck, Critically evaluating the theory and performance of Bayesian analysis of macroevolutionary mixtures. *Proc. Natl. Acad. Sci. U.S.A.* **113**, 9569–9574 (2016).

114. A. L. S. Meyer, J. J. Wiens, Estimating diversification rates for higher taxa: BAMM can give problematic estimates of rates and rate shifts. *Evolution* **72**, 39–53 (2018).

115. D. L. Rabosky, J. S. Mitchell, J. Chang, Is BAMM flawed? Theoretical and practical concerns in the analysis of multi-rate diversification models. *Syst. Biol.* **66**, 477–498 (2017).

116. D. L. Rabosky, BAMM at the court of false equivalency: A response to Meyer and Wiens. *Evolution* **72**, 2246–2256 (2018).

117. D. L. Rabosky, Extinction rates should not be estimated from molecular phylogenies. *Evolution* **64**, 1816–1824 (2010).

118. S. Louca, M. W. Pennell, Extant timetrees are consistent with a myriad of diversification histories. *Nature* **580**, 502–505 (2020).

119. D. L. Rabosky, M. Grundler, C. Anderson, P. Title, J. J. Shi, J. W. Brown, H. Huang, J. G. Larson, BAMMtools: An R package for the analysis of evolutionary dynamics on phylogenetic trees. *Methods Ecol. Evol.* **5**, 701–707 (2014).

120. H. Xu, X. Luo, J. Qian, X. Pang, J. Song, G. Qian, J. Chen, S. Chen, FastUniq: A fast *de novo* duplicates removal tool for paired short reads. *PLOS ONE* **7**, e52249 (2012).

121. A. M. Bolger, M. Lohse, B. Usadel, Trimmomatic: A flexible trimmer for Illumina sequence data. *Bioinformatics* **30**, 2114–2120 (2014).

122. M. G. Johnson, E. M. Gardner, Y. Liu, R. Medina, B. Goffinet, A. J. Shaw, N. J. C. Zerega, N. J. Wickett, HybPiper: Extracting coding sequence and introns for phylogenetics from high-throughput sequencing reads using target enrichment. *Appl. Plant Sci.* **4**, 1600016 (2016).

123. H. Li, R. Durbin, Fast and accurate short read alignment with Burrows-Wheeler transform. *Bioinformatics* **25**, 1754–1760 (2009).

124. A. Bankevich, S. Nurk, D. Antipov, A. A. Gurevich, M. Dvorkin, A. S. Kulikov, V. M. Lesin, S. I. Nikolenko, S. Pham, A. D. Prjibelski, A. V. Pyshkin, A. V. Sirotkin, N. Vyahhi, G. Tesler, M. A. Alekseyev, P. A. Pevzner, SPAdes: A new genome assembly algorithm and its applications to single-cell sequencing. *J. Comput. Biol.* **19**, 455–477 (2012).

125. G. S. C. Slater, E. Birney, Automated generation of heuristics for biological sequence comparison. *BMC Bioinformatics* **6**, 31 (2005).

126. Y. Yang, S. A. Smith, Orthology inference in nonmodel organisms using transcriptomes and low-coverage genomes: Improving accuracy and matrix occupancy for phylogenomics. *Mol. Biol. Evol.* **31**, 3081–3092 (2014).

127. V. Ranwez, S. Harispe, F. Delsuc, E. J. P. Douzery, MACSE: Multiple alignment of coding SEquences accounting for frameshifts and stop codons. *PLOS ONE* **6**, e22594 (2011).

128. J. W. Brown, J. F. Walker, S. A. Smith, Phyx: Phylogenetic tools for unix. *Bioinformatics* **33**, 1886–1888 (2017).

129. A. Stamatakis, RAxML version 8: A tool for phylogenetic analysis and post-analysis of large phylogenies. *Bioinformatics* **30**, 1312–1313 (2014).

130. U. Mai, S. Mirarab, TreeShrink: Fast and accurate detection of outlier long branches in collections of phylogenetic trees. *BMC Genomics* **19**, 272 (2018).

131. A. Criscuolo, S. Gribaldo, BMGE (Block Mapping and Gathering with Entropy): A new software for selection of phylogenetic informative regions from multiple sequence alignments. *BMC Evol. Biol.* **10**, 210 (2010).

132. J. J. Doyle, in *Polyploidy and Genome Evolution*, P. S. Soltis, D. E. Soltis, Eds. (Springer-Verlag, 2012), pp. 147–180.

133. R. Govindarajulu, C. E. Hughes, P. J. Alexander, C. Donovan Bailey, The complex evolutionary dynamics of ancient and recent polyploidy in *Leucaena* (Leguminosae; Mimosoideae). *Am. J. Bot.* **98**, 2064–2076 (2011).

134. A. Stamatakis, RAxML-VI-HPC: Maximum likelihood-based phylogenetic analyses with thousands of taxa and mixed models. *Bioinformatics* **22**, 2688–2690 (2006).

135. S. Q. Le, C. C. Dang, O. Gascuel, Modeling protein evolution with several amino acid replacement matrices depending on site rates. *Mol. Biol. Evol.* **29**, 2921–2936 (2012).

136. N. Lartillot, N. Rodrigue, D. Stubbs, J. Richer, PhyloBayes MPI: Phylogenetic reconstruction with infinite mixtures of profiles in a parallel environment. *Syst. Biol.* **62**, 611–615 (2013).

137. N. Lartillot, H. Brinkmann, H. Philippe, Suppression of long-branch attraction artefacts in the animal phylogeny using a site-heterogeneous model. *BMC Evol. Biol.* **7**, S4 (2007).

138. N. Lartillot, H. Philippe, A Bayesian mixture model for across-site heterogeneities in the amino-acid replacement process. *Mol. Biol. Evol.* **21**, 1095–1109 (2004).

139. M. Plummer, N. Best, K. Cowles, K. Vines, CODA: Convergence diagnosis and output analysis for MCMC. *R News* **6**, 7–11 (2006).

140. D. V Dugas, D. Hernandez, E. J. M. Koenen, E. Schwarz, S. Straub, C. E. Hughes, R. K. Jansen, M. Nageswara-Rao, M. Staats, J. T. Trujillo, N. H. Hajrah, N. S. Alharbi, A. L. Al-Malki, J. S. M. Sabir, C. D. Bailey, Mimosoid legume plastome evolution: IR expansion, tandem repeat expansions, and accelerated rate of evolution in *clpP*. *Sci. Rep.* **5**, 16958 (2015).

141. S. Huang, W. Wu, Z. Chen, Q. Zhu, W. L. Ng, Q. Zhou, Characterization of the chloroplast genome of *Erythrophleum fordii* (Fabaceae). *Conserv. Genet. Resour.* **11**, 165–167 (2019).

142. J. P. Huelsenbeck, F. Ronquist, MRBAYES: Bayesian inference of phylogeny. *Bioinformatics* **17**, 754–755 (2001).

143. A. Rambaut, A. J. Drummond, D. Xie, G. Baele, M. A. Suchard, Posterior summarisation in Bayesian phylogenetics using Tracer 1.7. *Syst. Biol.* **67**, 901–904 (2018).

144. B. D. Mishler, N. Knerr, C. E. González-Orozco, A. D. Thornhill, S. W. Laffan, J. T. Miller, Phylogenetic measures of biodiversity and neo- and paleo-endemism in Australian *Acacia*. *Nat. Commun.* **5**, 4473 (2014).

145. É. R. de Souza, G. P. Lewis, F. Forest, A. S. Schnadelbach, C. van den Berg, L. P. de Queiroz, Phylogeny of *Calliandra* (Leguminosae: Mimosoideae) based on nuclear and plastid molecular markers. *Taxon.* **62**, 1200–1219 (2013).

146. C. E. Hughes, C. D. Bailey, S. Krosnick, M. A. Luckow, Relationships among genera of the informal Dichrostachys and Leucaena groups (Mimosoideae) inferred from nuclear ribosomal ITS sequences. *Adv. Legum. Syst.* **10**, 221–238 (2003).

147. S. B. Cannon, M. R. McKain, A. Harkess, M. N. Nelson, S. Dash, M. K. Deyholos, Y. Peng, B. Joyce, C. N. Stewart, M. Rolf, T. Kutchan, X. Tan, C. Chen, Y. Zhang, E. Carpenter, G. K. S. Wong, J. J. Doyle, J. Leebens-Mack, Multiple polyploidy events in the early radiation of nodulating and nonnodulating legumes. *Mol. Biol. Evol.* **32**, 193–210 (2015).

148. M. F. Simon, R. Grether, L. P. de Queiroz, T. E. Särkinen, V. F. Dutra, C. E. Hughes, The evolutionary history of *Mimosa* (Leguminosae): Toward a phylogeny of the sensitive plants. *Am. J. Bot.* **98**, 1201–1221 (2011).

149. T. N. C. Vasconcelos, S. Alcantara, C. O. Andrino, F. Forest, M. Reginato, M. F. Simon, J. R. Pirani, Fast diversification through a mosaic of evolutionary histories characterizes the endemic flora of ancient Neotropical mountains. *Proc. R. Soc. B Biol. Sci.* **287**, 20192933 (2020).

150. M. Morales, R. H. Fortunato, M. F. Simon, A new species of *Mimosa* L. ser. *Bipinnatae* DC. (Leguminosae) from the Cerrado: Taxonomic and phylogenetic insights. *Plan. Theory* **9**, 934 (2020).

151. J. S. Boatwright, O. Maurin, M. van der Bank, Phylogenetic position of Madagascan species of *Acacia s.l.* and new combinations in *Senegalia* and *Vachellia* (Fabaceae, Mimosoideae, Acacieae). *Bot. J. Linn. Soc.* **179**, 288–294 (2015).

152. D. F. Comben, G. A. McCulloch, G. K. Brown, G. H. Walter, Phylogenetic placement and the timing of diversification in Australia's endemic *Vachellia* (Caesalpinioideae, Mimosoid Clade, Fabaceae) species. *Aust. Syst. Bot.* **33**, 103–109 (2020).

153. V. Terra, F. C. P. Garcia, L. P. de Queiroz, M. van der Banke, J. T. Miller, Phylogenetic relationships in *Senegalia* (Leguminosae-Mimosoideae) emphasizing the South American lineages. *Syst. Bot.* **42**, 458–464 (2017).

154. M. F. Simon, J. F. B. Pastore, A. F. Souza, L. M. Borges, V. R. Scalon, P. G. Ribeiro, J. Santos-Silva, V. C. Souza, L. P. Queiroz, Molecular phylogeny of *Stryphnodendron* (Mimosoideae, Leguminosae) and generic delimitations in the Piptadenia Group. *Int. J. Plant Sci.* **177**, 44–59 (2016).

155. V. Terra, J. J. Ringelberg, B. Maslin, E. J. M. Koenen, J. Ebinger, D. Seigler, C. E. Hughes, Dilemmas in generic delimitation of *Senegalia* and allies (Caesalpinioideae, mimosoid clade): How to reconcile phylogenomic evidence with morphology and taxonomy? *PhytoKeys* **205**, 261–278 (2022).

156. L. M. Borges, P. W. Inglis, M. F. Simon, P. G. Ribeiro, L. P. de Queiroz, Misleading fruits: The non-monophyly of *Pseudopiptadenia* and *Pityrocarpa* supports generic re-circumscriptions and a new genus within mimosoid legumes. *PhytoKeys* **205**, 239–259 (2022).

157. J. Ferm, A preliminary phylogeny of *Zapoteca* (Fabaceae: Caesalpinioideae: Mimosoid clade). *Plant Syst. Evol.* **305**, 341–352 (2019).

158. É. R. de Souza, M. V. Krishnaraj, L. P. de Queiroz, *Sanjappa*, a new genus in the tribe Ingeae (Leguminosae: Mimosoideae) from India. *Rheedea.* **26**, 1–12 (2016).

159. J. Ferm, P. Korall, G. P. Lewis, B. Ståhl, Phylogeny of the Neotropical legume genera *Zygia* and *Marmaroxylon* and close relatives. *Taxon.* **68**, 661–672 (2019).

160. G. E. Brewer, J. J. Clarkson, O. Maurin, A. R. Zuntini, V. Barber, S. Bellot, N. Biggs, R. S. Cowan, N. M. J. Davies, S. Dodsworth, S. L. Edwards, W. L. Eiserhardt, N. Epitawalage, S. Frisby, A. Grall, P. J. Kersey, L. Pokorny, I. J. Leitch, F. Forest, W. J. Baker, Factors affecting targeted sequencing of 353 nuclear genes from herbarium specimens spanning the diversity of Angiosperms. *Front. Plant Sci.* **10**, 1102 (2019).

161. M. L. Hart, L. L. Forrest, J. A. Nicholls, C. A. Kidner, Retrieval of hundreds of nuclear loci from herbarium specimens. *Taxon.* **65**, 1081–1092 (2016).

162. F. T. Bakker, A. Antonelli, J. A. Clarke, J. A. Cook, S. V. Edwards, P. G. P. Ericson, S. Faurby, N. Ferrand, M. Gelang, R. G. Gillespie, M. Irestedt, K. Lundin, E. Larsson, P. Matos-Maraví, J. Müller, T. von Proschwitz, G. K. Roderick, A. Schliep, N. Wahlberg, J. Wiedenhoeft, M. Källersjö, The global museum: Natural history collections and the future of evolutionary science and public education. *PeerJ.* **8**, e8225 (2020).

163. X. Jiang, S. V Edwards, L. Liu, The multispecies coalescent model outperforms concatenation across diverse phylogenomic data sets. *Syst. Biol.* **69**, 795–812 (2020).

164. R. P. Clark, K.-W. Jiang, E. Gagnon, Reinstatement of *Ticanto* (Leguminosae-Caesalpinioideae) – the final piece in the *Caesalpinia* group puzzle. *PhytoKeys* **205**, 59–98 (2022).

165. S. A. O'Donnell, J. J. Ringelberg, G. P. Lewis, Re-circumscription of the mimosoid genus *Entada* including new combinations for all species of the phylogenetically nested *Elephantorrhiza* (Leguminosae, Caesalpinioideae, mimosoid clade). *PhytoKeys* **205**, 99–145 (2022).

166. C. E. Hughes, J. J. Ringelberg, G. P. Lewis, S. A. Catalano, Disintegration of the genus *Prosopis* L. (Leguminosae, Caesalpinioideae, mimosoid clade). *PhytoKeys* **205**, 147–189 (2022).

167. C. E. Hughes, J. J. Ringelberg, M. Luckow, J. L. C. Jiménez, *Mezcala* – A new segregate genus of mimosoid legume (Leguminosae, Caesalpinioideae, mimosoid clade) narrowly endemic to the Balsas Depression in Mexico. *PhytoKeys* **205**, 191–201 (2022).

168. I. Tamayo-Cen, B. M. Torke, J. E. López Contreras, G. Carnevali Fernández-Concha, I. Ramírez Morillo, L. L. Can Itza, R. Duno de Stefano, Revisiting the phylogeny and taxonomy of the Pithecellobium clade (Leguminosae, Caesalpinioideae) with new generic circumscriptions. *PhytoKeys* **205**, 279–298 (2022).

169. G. K. Brown, J. Aju, M. J. Bayly, D. J. Murphy, T. G. B. McLay, Phylogeny and classification of the Australasian and Indomalayan mimosoid legumes *Archidendron* and *Archidendropsis* (Leguminosae, subfamily Caesalpinioideae, mimosoid clade). *PhytoKeys.* **205**, 299–333 (2022).

170. E. Demeulenaere, T. Schils, J. G. Burleigh, J. J. Ringelberg, E. J. M. Koenen, S. M. Ickert-Bond, Phylogenomic assessment prompts recognition of the *Serianthes* clade and confirms the monophyly of *Serianthes* and its relationship with *Falcataria* and *Wallaceodendron* in the wider ingoid clade (Leguminosae, Caesalpinioideae). *PhytoKeys* **205**, 335–361 (2022).

171. E. J. M. Koenen, On the taxonomic affinity of *Albizia carbonaria* Britton (Leguminosae, Caesalpinioideae-mimosoid clade). *PhytoKeys* **205**, 363–370 (2022).

172. G. A. Peraza, E. J. M. Koenen, R. Riina, C. E. Hughes, J. J. Ringelberg, G. C. Fernández-Concha, I. M. R. Morillo, L. L. C. Itza, I. Tamayo-Cen, J. H. R. Prado, X. Cornejo, S. Mattapha, R. D. de Stefano, Re-establishment of the genus *Pseudalbizzia* (Leguminosae, Caesalpinioideae, mimosoid clade): The New World species formerly placed in *Albizia*. *PhytoKeys* **205**, 371–400 (2022).

173. M. V. B. Soares, E. J. M. Koenen, J. R. V. Iganci, M. P. Morim, A new generic circumscription of *Hydrochorea* (Leguminosae, Caesalpinioideae, mimosoid clade) with an amphi-Atlantic distribution. *PhytoKeys* **205**, 401–437 (2022).

174. É. R. de Souza, P. G. C. de Almeida, L. Rocha, E. J. M. Koenen, M. A. Burgos, G. P. Lewis, C. E. Hughes, *Boliviadendron*, a new segregate genus of mimosoid legume (Leguminosae, Caesalpinioideae, mimosoid clade) narrowly endemic to the interior Andean valleys of Bolivia. *PhytoKeys* **205**, 439–452 (2022).

175. E. J. M. Koenen, *Osodendron* gen. nov. (Leguminosae, Caesalpinioideae), a new genus of mimosoid legumes of tropical Africa. *PhytoKeys* **205**, 453–470 (2022).

176. C. Meyer, P. Weigelt, H. Kreft, Multidimensional biases, gaps and uncertainties in global plant occurrence information. *Ecol. Lett.* **19**, 992–1006 (2016).

177. M. A. Hyde, B. T. Wursten, P. Ballings, M. Coates Palgrave, Flora of Mozambique: Species information: Individual images: *Entada rheedii*. www.mozambiqueflora.com/speciesdata/image-display.php?species_id=126520&image_id=13 [retrieved 31 May 2022].

178. A. South, *rworldxtra: Country Boundaries at High Resolution* (2012); R package version 1.01; https://CRAN.R-project.org/package=rworldxtra.

179. J. C. Zachos, G. R. Dickens, R. E. Zeebe, An early Cenozoic perspective on greenhouse warming and carbon-cycle dynamics. *Nature* **451**, 279–283 (2008).

180. H. Morlon, E. Lewitus, F. L. Condamine, M. Manceau, J. Clavel, J. Drury, RPANDA: An R package for macroevolutionary analyses on phylogenetic trees. *Methods Ecol. Evol.* **7**, 589–597 (2016).

181. D. J. Bertioli, S. B. Cannon, L. Froenicke, G. Huang, A. D. Farmer, E. K. S. Cannon, X. Liu, D. Gao, J. Clevenger, S. Dash, L. Ren, M. C. Moretzsohn, K. Shirasawa, W. Huang, B. Vidigal, B. Abernathy, Y. Chu, C. E. Niederhuth, P. Umale, Ana Cláudia G Araújo, A. Kozik, K. D. Kim, M. D. Burow, R. K. Varshney, X. Wang, X. Zhang, N. Barkley, P. M. Guimarães, S. Isobe, B. Guo, B. Liao, H Thomas Stalker, R. J. Schmitz, B. E. Scheffler, Soraya C M Leal-Bertioli, X. Xun, S. A. Jackson, R. Michelmore, P. Ozias-Akins, The genome sequences of *Arachis duranensis* and *Arachis ipaensis*, the diploid ancestors of cultivated peanut. *Nat. Genet.* **48**, 438–446 (2016).

182. J. S. Stai, A. Yadav, C. Sinou, A. Bruneau, J. J. Doyle, D. Fernández-Baca, S. B. Cannon, *Cercis*: A non-polyploid genomic relic within the generally polyploid legume family. *Front. Plant Sci.* **10**, 345 (2019).

183. J. Schmutz, S. B. Cannon, J. Schlueter, J. Ma, T. Mitros, W. Nelson, D. L. Hyten, Q. Song, J. J. Thelen, J. Cheng, D. Xu, U. Hellsten, G. D. May, Y. Yu, T. Sakurai, T. Umezawa, M. K. Bhattacharyya, D. Sandhu, B. Valliyodan, E. Lindquist, M. Peto, D. Grant, S. Shu, D. Goodstein, K. Barry, M. Futrell-Griggs, B. Abernathy, J. Du, Z. Tian, L. Zhu, N. Gill, T. Joshi, M. Libault, A. Sethuraman, X.-C. Zhang, K. Shinozaki, H. T. Nguyen, R. A. Wing, P. Cregan, J. Specht, J. Grimwood, D. Rokhsar, G. Stacey, R. C. Shoemaker, S. A. Jackson, Genome sequence of the palaeopolyploid soybean. *Nature* **463**, 178–183 (2010).

184. N. D. Young, F. Debellé, G. E. D. Oldroyd, R. Geurts, S. B. Cannon, M. K. Udvardi, V. A. Benedito, K. F. X. Mayer, J. Gouzy, H. Schoof, Y. Van de Peer, S. Proost, D. R. Cook, B. C. Meyers, M. Spannagl, F. Cheung, S. De Mita, V. Krishnakumar, H. Gundlach, S. Zhou, J. Mudge, A. K. Bharti, J. D. Murray, M. A. Naoumkina, B. Rosen, K. A. T. Silverstein, H. Tang, S. Rombauts, P. X. Zhao, P. Zhou, V. Barbe, P. Bardou, M. Bechner, A. Bellec, A. Berger, H. Bergès, S. Bidwell, T. Bisseling, N. Choisne, A. Couloux, R. Denny, S. Deshpande, X. Dai, J. J. Doyle, A.-M. Dudez, A. D. Farmer, S. Fouteau, C. Franken, C. Gibelin, J. Gish, S. Goldstein, A. J. González, P. J. Green, A. Hallab, M. Hartog, A. Hua, S.

J. Humphray, D.-H. Jeong, Y. Jing, A. Jöcker, S. M. Kenton, D.-J. Kim, K. Klee, H. Lai, C. Lang, S. Lin, S. L. Macmil, G. Magdelenat, L. Matthews, J. M. Corrison, E. L. Monaghan, J.-H. Mun, F. Z. Najar, C. Nicholson, C. Noirot, M. O'Bleness, C. R. Paule, J. Poulain, F. Prion, B. Qin, C. Qu, E. F. Retzel, C. Riddle, E. Sallet, S. Samain, N. Samson, I. Sanders, O. Saurat, C. Scarpelli, T. Schiex, B. Segurens, A. J. Severin, D Janine Sherrier, R. Shi, S. Sims, S. R. Singer, S. Sinharoy, L. Sterck, A. Viollet, B.-B. Wang, K. Wang, M. Wang, X. Wang, J. Warfsmann, J. Weissenbach, D. D. White, J. D. White, G. B. Wiley, P. Wincker, Y. Xing, L. Yang, Z. Yao, F. Ying, J. Zhai, L. Zhou, A. Zuber, J. Dénarié, R. A. Dixon, G. D. May, D. C. Schwartz, J. Rogers, F. Quétier, C. D. Town, B. A. Roe, The *Medicago* genome provides insight into the evolution of rhizobial symbioses. *Nature* **480**, 520–524 (2011).

185. M. Griesmann, Y. Chang, X. Liu, Y. Song, G. Haberer, M. B. Crook, B. Billault-Penneteau, D. Lauressergues, J. Keller, L. Imanishi, Y. P. Roswanjaya, W. Kohlen, P. Pujic, K. Battenberg, N. Alloisio, Y. Liang, H. Hilhorst, M. G. Salgado, V. Hocher, H. Gherbi, S. Svistoonoff, J. J. Doyle, S. He, Y. Xu, S. Xu, J. Qu, Q. Gao, X. Fang, Y. Fu, P. Normand, A. M. Berry, L. G. Wall, J.-M. Ané, K. Pawlowski, X. Xu, H. Yang, M. Spannagl, K. F. X. Mayer, Gane Ka-Shu Wong, M. Parniske, P.-M. Delaux, S. Cheng, Phylogenomics reveals multiple losses of nitrogen-fixing root nodule symbiosis. *Science* **361**, eaat1743 (2018).

186. S. L. Wing, F. Herrera, C. A. Jaramillo, C. Gómez-Navarro, P. Wilf, C. C. Labandeira, Late Paleocene fossils from the Cerrejón Formation, Colombia, are the earliest record of Neotropical rainforest. *Proc. Natl. Acad. Sci. U.S.A.* **106**, 18627–18632 (2009).

187. F. Herrera, M. R. Carvalho, S. L. Wing, C. Jaramillo, P. S. Herendeen, Middle to Late Paleocene Leguminosae fruits and leaves from Colombia. *Aust. Syst. Bot.* **32**, 385–408 (2019).

188. H. D. MacGinitie, in *Carnegie Institution of Washington publication 599. Contributions of Paleontology* (Washington D.C., USA, 1953).

189. P. S. Herendeen, F. Herrera, Eocene fossil legume leaves referable to the extant genus *Arcoa* (Caesalpinioideae, Leguminosae). *Int. J. Plant Sci.* **180**, 220–231 (2019).

190. W. L. Crepet, D. L. Dilcher, Investigations of angiosperms from the Eocene of North America: A mimosoid inflorescence. *Am. J. Bot.* **64**, 714–725 (1977).

191. P. Guinet, E. Sabrouty, H. A. Soliman, A. M. Omrah, Études des caractères du pollen des Légumineuses-Mimosoideae des sédiments Tertiaires du Nord Ouest de l'Égypte. *Mém. Travel. Ec. Prat. des Hautes Etudes, Inst. Montpellier.* **17**, 159–171 (1987).

192. J. T. Miller, D. J. Murphy, S. Y. W. Ho, D. J. Cantrill, D. Seigler, Comparative dating of *Acacia*: Combining fossils and multiple phylogenies to infer ages of clades with poor fossil records. *Aust. J. Bot.* **61**, 436–445 (2013).

193. M. Caccavari, V. Barreda, A new calymmate mimosoid polyad from the Miocene of Argentina. *Rev. Palaeobot. Palynol.* **109**, 197–203 (2000).
